# Rapid regio- and multi-coupling reactivity of 2,3-dibromobenzofurans with atom-economic triarylbismuths under palladium catalysis

**DOI:** 10.3762/bjoc.12.195

**Published:** 2016-09-22

**Authors:** Maddali L N Rao, Jalindar B Talode, Venneti N Murty

**Affiliations:** 1 Department of Chemistry, Indian Institute of Technology Kanpur, Kanpur-208016, U.P., India, Tel/ Fax: +91-512-2597532

**Keywords:** bromobenzofuran coupling, cross-coupling, palladium, regio-selective, triarylbismuth

## Abstract

A regio- and chemoselective cross-coupling study using 2,3-dibromobenzofurans and 2,3,5-tribromobenzofuran was achieved with sub-stoichiometric loadings of triarylbismuths as atom-economic reagents under Pd-catalyzed conditions. As part of this study, various 2,3-diaryl- and 2,3,5-triarylbenzofuran products were obtained in high yields, involving one-pot operations and short reaction times.

## Introduction

**Figure 1:**
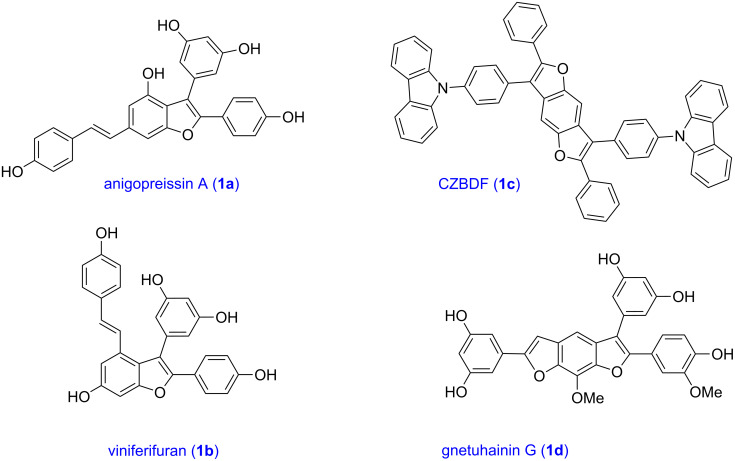
Important benzofuran skeletons.

The benzofuran scaffold is present in various biologically active molecules [1–8], natural products [9–15] and also part of various functional materials [16]. Importantly, 2,3-disubstituted benzofurans are biologically important (Figure 1a–d) and a few reports about their isolation and synthetic methods are available [17–19]. Substituted benzofurans serve as antitumor agents [20], protein tyrosine phosphatase-1B inhibitors [21], antimycobacterial agents [22] and as ambipolar materials (CZBDF, Figure 1c) [16]. To note, synthetic functionalization under transition-metal-catalyzed conditions allows the preparation of multi-substituted benzofurans in a facile manner [23–28]. Langer et al. reported the site-selective Suzuki–Miyaura reaction of 2,3-dibromobenzofuran with arylboronic acids under palladium catalyzed conditions [29–30]. Bach et al. reported site-selective studies involving the Sonogashira, Negishi, Kumada cross-couplings employing 2,3-dibromobenzofuran and 2,3,5-tribromobenzofuran substrates [31–33]. Additionally, Langer et al. reported the synthesis of 2,3-dialkenylbenzofurans and functionalized dibenzofurans with domino “twofold Heck/6π-electrocyclization” of 2,3-di- and 2,3,5-tribromobenzofuran substrates [34].

**Scheme 1:**
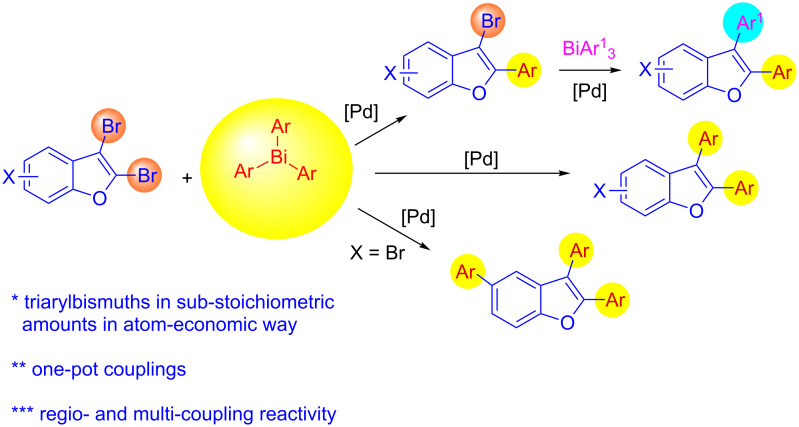
Bis- and tris-couplings.

In this regard, the cross-coupling studies of triarylbismuth reagents in regioselective studies with functionalized bromobenzofurans were not reported so far (Scheme 1) [35]. Given the importance of threefold couplings’ reactivity realized with the sub-stoichiometric loading of triarylbismuths in the cross-coupling reactions [35–42], we report herein, a novel regio- and multi-coupling of bromobenzofurans with triarylbismuth reagents under palladium coupling conditions.

## Results and Discussion

This study was initiated with 2,3-dibromobenzofuran for the investigation of the regio-selective coupling using a triarylbismuth reagent in substoichiometric amounts under Pd-catalyzed conditions (Table 1). A trial reaction was performed with 2,3-dibromobenzofuran (**1.1,** 3.3 equiv) and tri(*p*-anisyl)bismuth (1 equiv) with Pd(OAc)_2_/PPh_3_, Cs_2_CO_3_ (3 equiv) in *N*-methyl-2-pyrrolidone (NMP) at 90 °C for 1 h as protocol conditions [35]. This protocol furnished the preferential cross-coupling at the more electrophilic 2-Br position of 2,3-dibromobenzofuran (**1.1**) [29]. This reaction delivered 2-aryl-3-bromobenzofuran **2.1** in 46% yield (Table 1, entry 1) and the corresponding bis-arylation product involoving both 2- and 3-Br positions was not formed. Under similar conditions but with Cs_2_CO_3_ (4 equiv) as base, the cross-coupling yield was increased to 73% (Table 1, entry 2). A further change in reaction time to 2 h raised the desired yield to 95% (Table 1, entry 3). An additional check with bases K_3_PO_4_ or KOAc did not furnish high yields (Table 1, entries 4 and 5). Investigations using solvents such as *N,N*-dimethylformamide (DMF) and *N,N*-dimethylacetamide (DMA) furnished lowered yields (Table 1, entries 6 and 7) in comparison with NMP solvent. Carrying out the cross-couplings at different temperatures also gave lower yields (Table 1, entries 8 and 9). Additionally, the stoichiometric combination of 3 equiv of 2,3-dibromobenzofuran (**1.1**) and 1 equiv of bismuth reagent gave 86% yield (Table 1, entry 10). A few control reactions without base or palladium catalyst showed inferior or no cross-coupling reactivity (Table 1, entries 11 and 12). This investigation results that the desired regio-selective cross-coupling reactivity could be obtained in excellent yield with Pd(OAc)_2_/4 PPh_3_ (0.1 equiv) Cs_2_CO_3_ (4 equiv) in NMP at 90 °C and 2 h reaction time (Table 1, entry 3) and it was considered as optimized protocol for our further study.

**Table 1:** Screening for mono-arylation.^a^

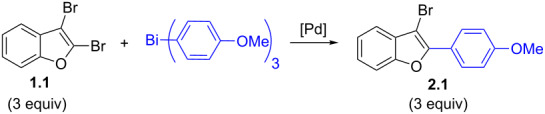					

Entry	Base (equiv)	Solvent	Temp. (°C)	Time (h)	Yield (%) (**2.1**)

1	Cs_2_CO_3_ (3)	NMP	90	1	46
2	Cs_2_CO_3_ (4)	NMP	90	1	73
3	Cs_2_CO_3_ (4)	NMP	90	2	95
4	K_3_PO_4_ (4)	NMP	90	2	79
5	KOAc (4)	NMP	90	2	61
6	Cs_2_CO_3_ (4)	DMF	90	2	71
7	Cs_2_CO_3_ (4)	DMA	90	2	80
8	Cs_2_CO_3_ (4)	NMP	110	2	84
9	Cs_2_CO_3_ (4)	NMP	60	2	47
10	Cs_2_CO_3_ (4)	NMP	90	2	86^b^
11	None	NMP	90	2	05
12	Cs_2_CO_3_ (4)	NMP	90	2	None^c^

^a^Reaction conditions: 2,3-Dibromobenzofuran (**1.1**) 0.825 mmol, 3.3 equiv), Bi(*p*-anisyl)_3_ (0.25 mmol, 1 equiv), Pd(OAc)_2_ (0.025 mmol, 0.1 equiv), PPh_3_ (0.1 mmol, 0.4 equiv), base (0.75–1 mmol, 3–4 equiv), and solvent (3 mL), temp., time. Isolated yields based on three aryl couplings from BiAr_3_. Bianisyl formed in 5–15% yields. ^b^With 2,3-dibromobenzofuran (**1.1**) (0.75 mmol, 3 equiv). ^c^Without Pd-catalyst.

To check the generality of this regio-selective coupling, various 2,3-dibromobenzofurans have been tested with differently functionalized triphenylbismuth reagents under the optimized conditions (Table 2). This study was performed with triphenylbismuth reagents substituted with electronically activating and deactivating groups. The cross-couplings performed with these reagents demonstrated an excellent general reactivity (Table 2, entries 1–12). It was highly satisfying to note that the corresponding products **2.1**–**2.12** were obtained in 79–95% yields. It prompted us to extend our study to other functionalized 2,3-dibromobenzofuran substrates. For example, a few bismuth couplings carried out with 2,3-dibromo-5-nitrobenzofuran (**1.2**) furnished the corresponding 2-aryl-3-bromobenzofurans **2.13**–**2.15** in 76–88% yields (Table 2, entries 13–15**)**. Additionally, we have also planned chemoselective couplings with differently functionalized 2,3-dibromobenzofurans. This study using 2,3-dibromobenzofurans functionalized with 5-chloro, 5,7-dichloro, 7-bromo-5-chloro and 5-bromo groups **1.3**–**1.6** furnished exclusive arylations at C-2 position.

**Table 2:** Cross-couplings of 2,3-dibromobenzofurans with BiAr_3_ reagents.^a^

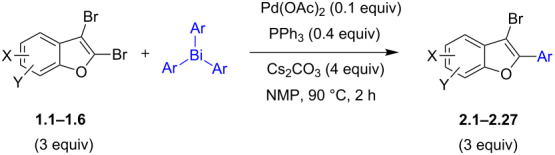					

Entry	2,3-Dibromobenzofurans		2-Aryl-3-bromobenzofurans		Yield (%)

1	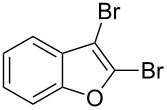	**1.1**	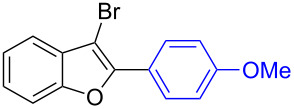	**2.1**	95
2	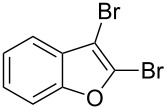	**1.1**	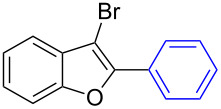	**2.2**	88
3	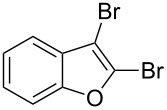	**1.1**	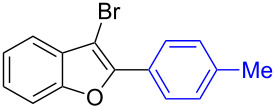	**2.3**	81
4	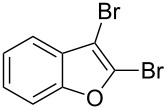	**1.1**	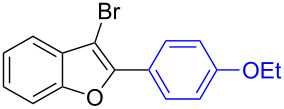	**2.4**	85
5	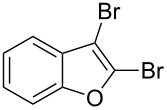	**1.1**	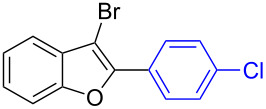	**2.5**	89
6	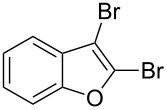	**1.1**	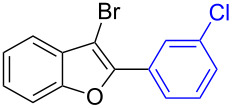	**2.6**	86
7	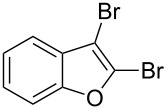	**1.1**	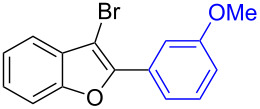	**2.7**	82
8	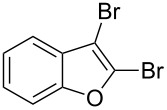	**1.1**	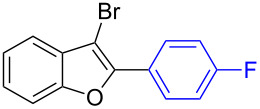	**2.8**	93
9	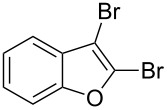	**1.1**	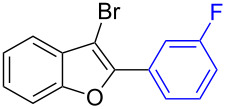	**2.9**	90
10	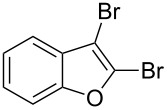	**1.1**	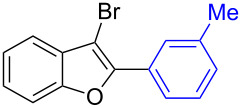	**2.10**	79
11	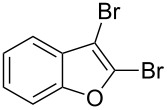	**1.1**	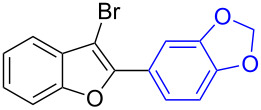	**2.11**	92
12	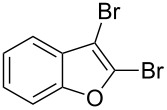	**1.1**	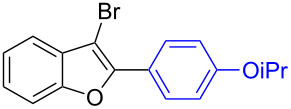	**2.12**	93
13	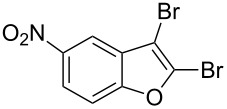	**1.2**	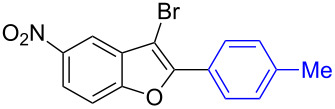	**2.13**	82
14	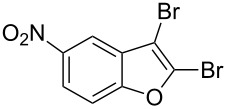	**1.2**	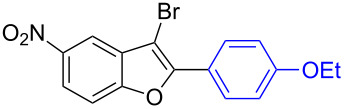	**2.14**	88
15	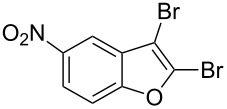	**1.2**	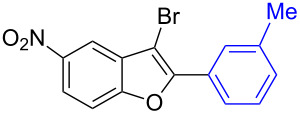	**2.15**	76
16	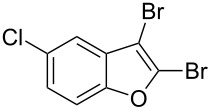	**1.3**	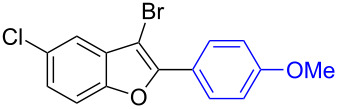	**2.16**	91
17	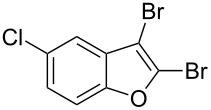	**1.3**	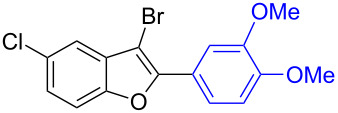	**2.17**	82
18	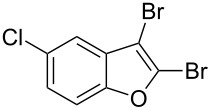	**1.3**	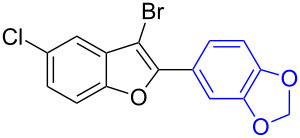	**2.18**	87
19	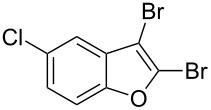	**1.3**	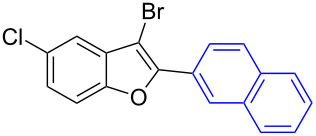	**2.19**	63
20	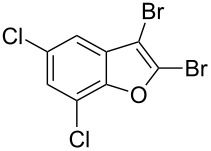	**1.4**	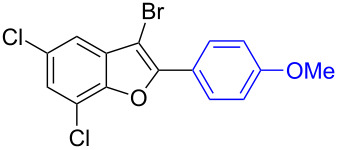	**2.20**	87
21	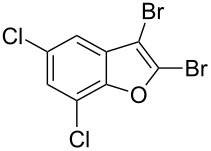	**1.4**	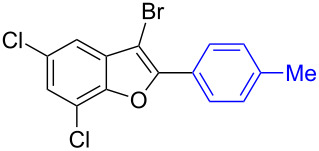	**2.21**	79
22	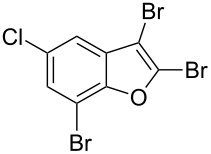	**1.5**	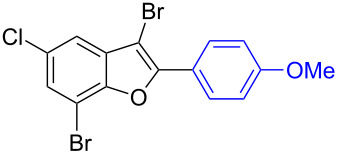	**2.22**	70
23	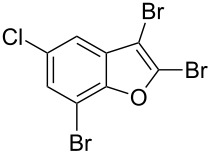	**1.5**	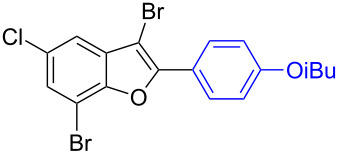	**2.23**	73
24	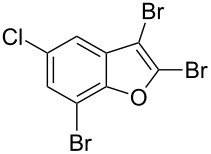	**1.5**	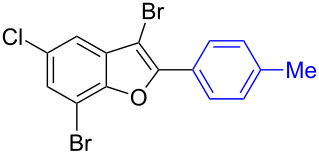	**2.24**	68
25	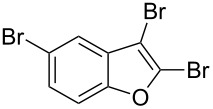	**1.6**	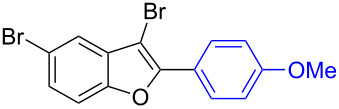	**2.25**	92
26	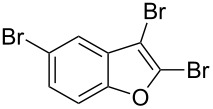	**1.6**	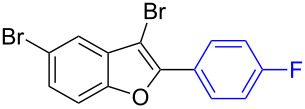	**2.26**	87
27	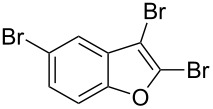	**1.6**	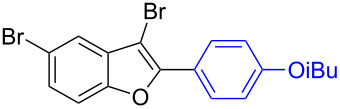	**2.27**	77

^a^Reaction conditions: 2,3-Dibromobenzofurans (0.825 mmol, 3.3 equiv), BiAr_3_ (0.25 mmol, 1 equiv), Cs_2_CO_3_ (1 mmol, 4 equiv), Pd(OAc)_2_ (0.025 mmol, 0.1 equiv), PPh_3_ (0.1 mmol, 0.4 equiv), NMP (3 mL), 90 °C, 2 h. Isolated yields based on three aryl couplings from BiAr_3_. Biaryl from BiAr_3_ formed in minor amounts.

In these cases, the corresponding 2-aryl-3-bromobenzofuran products **2.16**–**2.27** were obtained in high yields (Table 2, entries 16-27). It is to be mentioned that, despite the known facile coupling nature of aryl bromide [42] and its presence as part of the substrate, we obtained high regio-selective couplings with polyhalogenated benzofurans. The corresponding coupling products **2.22**–**2.27** were obtained in high yields (Table 2, entries 22–27). It is to be mentioned at this stage that in comparison to similar cross-couplings carried out with aryl boronic acids [29], the present method with triarylbismuth reagents showed appreciable reactivity with threefold coupling advantage and extended substrate scope. The overall resulted regio- and chemo-selective couplings encouraged us to investigate further arylation studies with triarylbismuth reagents. It is to note that, under these mono-arylation coupling conditions comprising an 1:3 stoichiometric ratio of BiAr_3_:dibromide combination we have not obtained the formation of any bis-arylated product. Hence, it was of interest to explore towards a one-pot bis-coupling method to synthesize 2,3-diarylbenzofurans using 2,3-dibromobenzofuran which involves couplings at both 2-bromo and 3-bromo positions. With this aim, it was investigated with appropriate sub-stoichiometric amounts of triarylbismuth reagents to obtain bis-coupling product (Table 3).

**Table 3:** Screening for bis-arylation.^a^

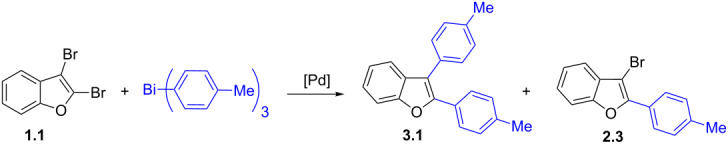				

Entry	Catalyst	Temp. (°C)	Time (h)	Yield (%)^b ^**3.1** (**2.3**)

1	Pd(OAc)_2_/4 PPh_3_	90	2	54 (31)
2	Pd(OAc)_2_/4 PPh_3_	90	4	72 (4)
3	Pd(OAc)_2_/4 PPh_3_	110	2	77 (2)

^a^Reaction conditions: 2,3-Dibromobenzofuran (**1.1**) (0.375 mmol, 1.5 equiv), Bi(*p*-tolyl)_3_ (0.25 mmol, 1 equiv), Pd(OAc)_2_ (0.025 mmol, 0.1 equiv), PPh_3_ (0.1 mmol, 0.4 equiv), Cs_2_CO_3_ (1 mmol, 4 equiv), NMP (3 mL), temp, time. ^b^Isolated yield (**3.1**) based on three aryl couplings from BiAr_3_. Bitolyl formed in 5–15% yields. Isolated yield of **2.3** given in parenthesis.

**Figure 2:**
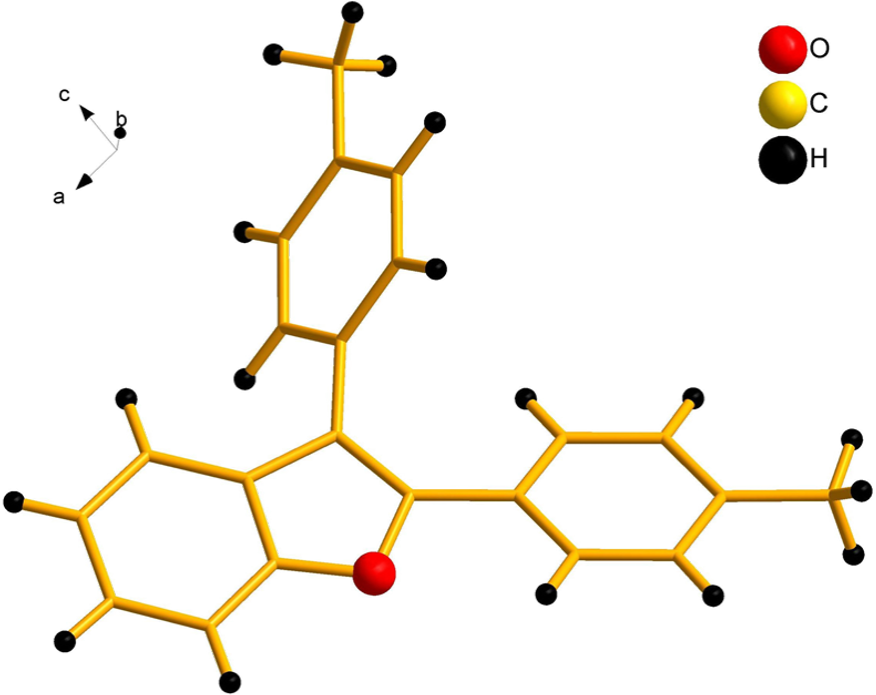
X-ray structure of bis-coupling product **3.1** (CCDC-1425338) [43].

Our initial attempt with mono-arylation catalytic conditions but with stoichiometric amount of bismuth reagent afforded 2,3-diarylbenzofuran **3.1** in 54% yield along with mono-arylated **2.3** in 31% yield (Table 3, entry 1). Importantly, this bis-coupling process was expected to go firstly through the formation of a mono-arylated product followed by its involvement in a second coupling with bismuth reagent. Hence to improve the bis-coupling process, a brief screening was performed under similar conditions but with 4 h reaction time. This gave the desired bis-coupling in 72% yield along with minor amount of mono-arylated product (Table 3, entry 2). It was further increased to 77% at 110 °C in 2 h conditions (Table 3, entry 3) along with mono-arylated product **2.3** in minor amount. Hence, these conditions were adopted as optimized protocol for our bis-coupling study with triarylbismuth reagents (Table 4). Encouragingly, these bis-couplings using the established conditions afforded symmetrically substituted 2,3-diarylbenzofurans **3.1**–**3.7** in 72–85% yields (Table 4, entries 1–7). In these cases, the corresponding mono-arylated products (**2.1**–**2.3**, **2.8**, **2.10**) were also isolated in minor amounts. Evidently, this generalization of bis-coupling reactivity was proved to be operationally simple with high reactivity and yields involving 2 h short reaction time and with sub-stoichiometric loadings of the bismuth reagent. Incidentally, we could also obtain the X-ray structure analysis for diarylbenzofuran **3.1** as given in Figure 2. The ease of formation of symmetrically substituted bis-coupled products inspired us to additionally develop a viable procedure for the synthesis of unsymmetrically substituted bis-arylated benzofurans. To accomplish this, we initially reacted 2-aryl-3-bromobenzofuran **2.1** with bismuth reagent using the palladium protocol conditions established for symmetrical bis-arylations (Table 5).

**Table 4:** Bis-coupling of 2,3-dibromobenzofurans.^a^

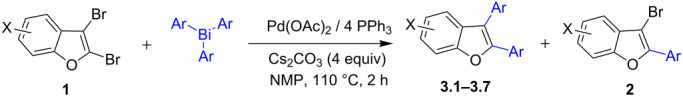			

Entry	2,3-Dibromobenzofurans	Bis- and mono-aryl benzofurans	

1	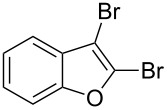 **1.1**	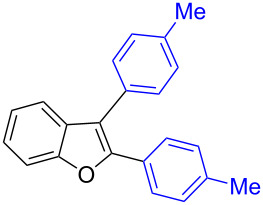 **3.1** (77%)	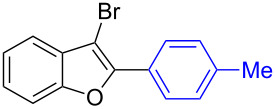 **2.3** (2%)
2	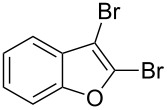 **1.1**	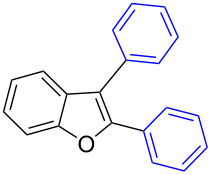 **3.2** (85%)	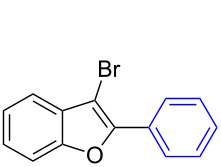 **2.2** (10%)
3	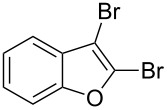 **1.1**	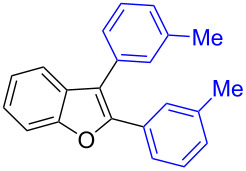 **3.3** (72%)	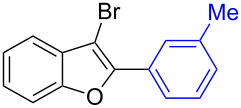 **2.10** (9%)
4	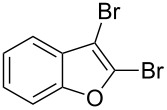 **1.1**	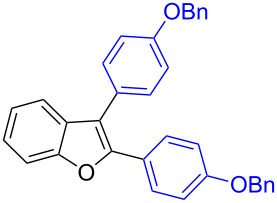 **3.4** (72%)^b^	
5	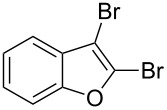 **1.1**	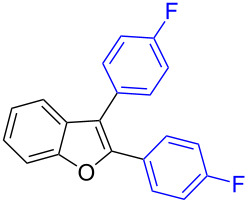 **3.5** (76%)	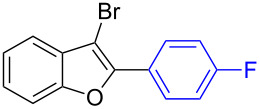 **2.8** (11%)
6	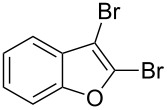 **1.1**	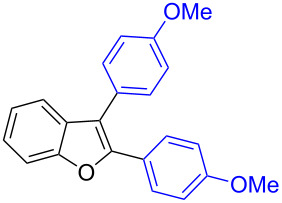 **3.6** (82%)	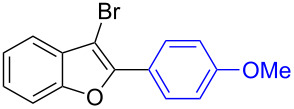 **2.1** (10%)
7	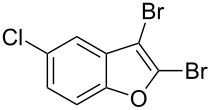 **1.3**	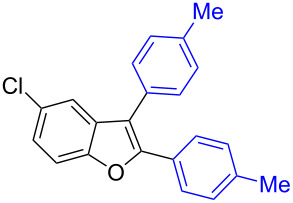 **3.7** (85%)^b^	

^a^Reaction conditions: 2,3-Dibromobenzofurans (0.375 mmol, 1.5 equiv), BiAr_3_ (0.25 mmol, 1 equiv), Pd(OAc)_2_ (0.025 mmol, 0.1 equiv), PPh_3_ (0.1 mmol, 0.4 equiv), Cs_2_CO_3_ (1 mmol, 4 equiv), NMP (3 mL), 110 °C, 2 h. Isolated yields based on three aryl couplings from BiAr_3_. Biaryl from BiAr_3_ formed in minor amounts. ^b^Mono-arylated product **2** not found.

It was carried out at 110 °C for 2 h and this furnished unsymmetrically substituted 2,3-diarylbenzofuran **4.1** in 54% yield along with the recovery of starting 2-aryl-3-bromobenzofuran **2.1** in 42% yield (Table 5, entry 1). As the reaction was found to be incomplete, a further investigation was carried out with 3 h reaction time. In this case, the desired unsymmetrically substituted bis-arylated product **4.1** was increased to 83% yield (Table 5, entry 2). Encouraged with this, a few more diarylations were attempted with different bismuth reagents. These reactions also furnished the corresponding unsymmetrically substituted 2,3-diarylbenzofurans **4.2** and **4.3** in 81% and 73% yields (Table 5, entries 3 and 4) with minor amount of unreacted starting material 2-aryl-3-bromobenzofuran **2.1**.

**Table 5:** Cross-couplings of 2-aryl-3-bromobenzofurans.^a^

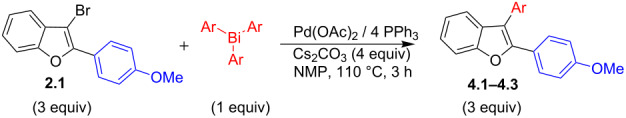				

Entry	BiAr_3_	2,3-Diarylbenzofurans		Yield (%)^b^

1	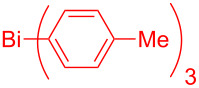	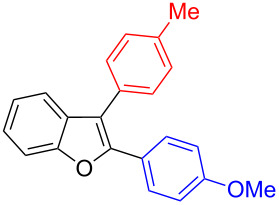	**4.1**	54 (42)^c^
2	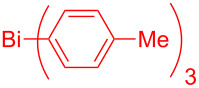	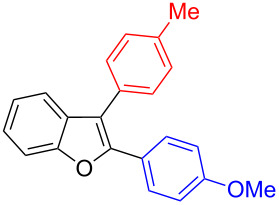	**4.1**	83 (11)
3	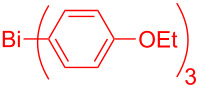	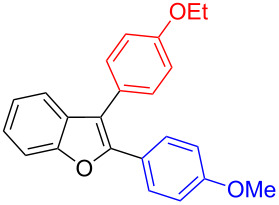	**4.2**	81(12)
4	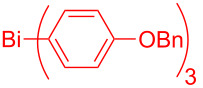	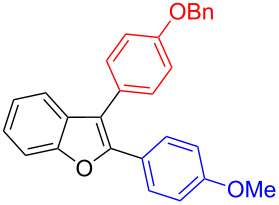	**4.3**	73 (11)

^a^Reaction conditions: 2-Aryl-3-bromobenzofuran (**2.1**, 0.375 mmol, 3 equiv), BiAr_3_ (0.125 mmol, 1 equiv), Pd(OAc)_2_ (0.0125 mmol, 0.1 equiv), PPh_3_ (0.05 mmol, 0.4 equiv), Cs_2_CO_3_ (0.5 mmol, 4 equiv), NMP (3 mL), 110 °C, 3 h. Isolated yields based on three aryl couplings from BiAr_3_. Biaryl from BiAr_3_ formed in minor amounts. ^b^Recovered **2.1** in parenthesis. ^c^Reaction time was 2 h.

The good coupling reactivity of 2-aryl-3-bromobenzofurans to give unsymmetrically substituted 2,3-diarylbenzofurans was impressive. Hence, we were inquisitive to develop a pot-economic protocol to directly access these products minimizing the purification procedure after mono-arylation. Hence, it was performed in a stepwise manner (step 1 and step 2) in a one-pot operation without any intermediate isolation. These results are given in Table 6. To elaborate, firstly we carried out the preparation of 2-aryl-3-bromobenzofuran in step 1 and it was followed by a second arylation at the 3-position as part of step 2. This pot-economic approach afforded mixed 2,3-diarylbenzofurans **4.1**–**4.3** in 68–72% yields (Table 6, entries 1–3). In all these reactions, we have also isolated the mono-aryl product from step 1 (2-aryl-3-bromobenzofuran, **2.1**) in minor amounts. In fact, bis-arylated yields obtained in this one-pot operation are on par with that obtained in Table 5. This reflects the efficient nature of our established pot-economic protocol employing different triarylbismuth reagents. In literature, bis-aryl couplings with arylboronic acids were reported with good reactivity under different heating conditions for mono- and bis-arylations [29–30]. As mentioned before, our couplings employing triarylbismuths reacted on par with good reativity in addition to threefold coupling advantage with sub-stoichiometric loadings.

**Table 6:** Pot-economic synthesis of unsymmetrical 2,3-diarylbenzofurans.^a^

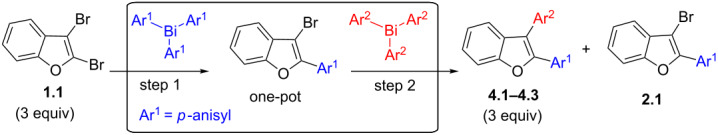				

Entry	BiAr^1^_3_	BiAr^2^_3_	2,3-Diarylbenzofurans	

1	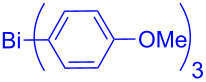	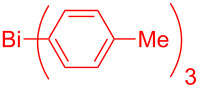	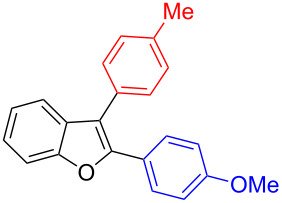 **4.1** (72%)	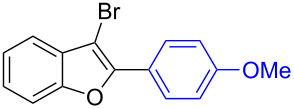 **2.1** (15%)
2	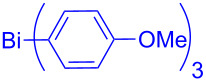	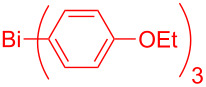	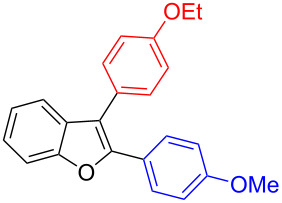 **4.2** (70%)	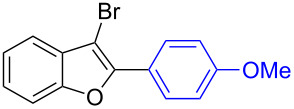 **2.1** (16%)
3	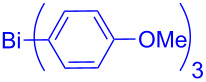	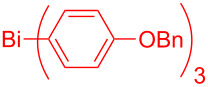	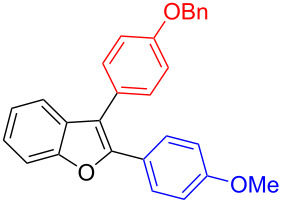 **4.3** (68%)	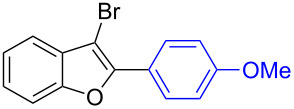 **2.1** (19%)

^a^Reaction conditions for Step 1: 2,3-Dibromobenzofuran (**1.1**, 0.375 mmol, 3 equiv), BiAr^1^_3_ (0.125 mmol, 1 equiv), Cs_2_CO_3_ (0.5 mmol, 4 equiv), Pd(OAc)_2_ (0.0125 mmol, 0.1 equiv), PPh_3_ (0.05 mmol, 0.4 equiv), NMP, 90 °C, 2 h; Reaction conditions for Step 2: BiAr^2^_3_ (0.125 mmol, 1 equiv), Cs_2_CO_3_ (0.25 mmol, 2 equiv), Pd(OAc)_2_ (0.0062 mmol, 0.05 equiv), PPh_3_ (0.025 mmol, 0.2 equiv), NMP, 110 °C, 2 h. Isolated yields based on three aryl couplings from BiAr^1^_3_ and BiAr^2^_3_. Biaryl from BiAr_3_ formed in minor amounts.

Further coupling study was carried out with 2,3,5-tribromobenzofuran (**1.6**) for the plausible threefold arylation under Pd-coupling conditions (Table 7). With some experimentation, it was realized that threefold arylation of 2,3,5-tribromobenzofuran (**1.6**) using triarylbismuth reagent is possible to give 2,3,5-triarylbenzofuran in high yiled after a reaction time of 4 h.

**Table 7:** Tris-coupling of 2,3,5-tribromobenzofuran.^a^

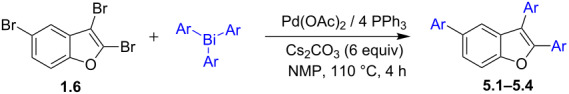				

Entry	BiAr_3_	2,3,5-Triarylbenzofuran		Yield (%)

1	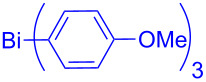	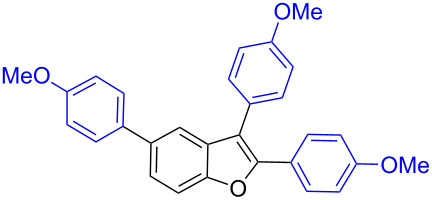	**5.1**	79
2	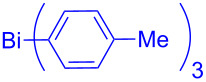	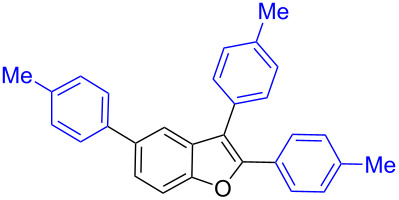	**5.2**	78
3	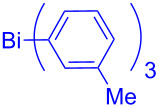	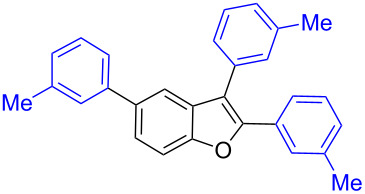	**5.3**	62
4	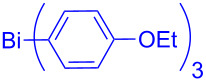	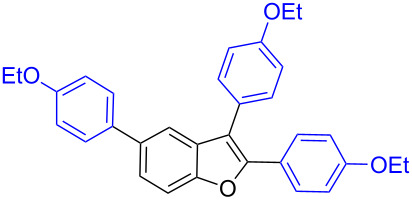	**5.4**	75

^a^Reaction conditions: 2,3,5-Tribromobenzofuran (**1.6**, 0.25 mmol, 1 equiv), BiAr_3_ (0.25 mmol, 1 equiv), Pd(OAc)_2_ (0.025 mmol, 0.1 equiv), PPh_3_ (0.1 mmol, 0.4 equiv), Cs_2_CO_3_ (1.5 mmol, 6 equiv), NMP (3 mL), 110 °C, 4 h. Isolated yields based on three aryl couplings from BiAr_3_. Biaryl from BiAr_3_ formed in minor amounts.

Hence, these couplings were conducted employing different triarylbismuth reagents. These tris-couplings afforded 2,3,5-triarylated benzofurans **5.1**–**5.4** in 62–79% yields (Table 7, entries 1–4). To note, all the three new aryl couplings were obtained in 4 h short duration of time in a one-pot operation and is synthetically advantageous. This reactivity is on par with similar study reported with arylboronic acids [30]. Thus the present method of the preparation of 2,3,5-triarylbenzofurans is expected to serve as a useful protocol to access these skeletons in a facile manner.

## Conclusion

We have established the couplings of 2,3-dibromobenzofurans and 2,3,5-tribromobenzofuran with high yields and faster reactivity using triarylbismuth reagents as atom-economic reagents. The Pd-catalyzed couplings carried out with triarylbismuths in sub-stoichiometric loadings allowed the synthesis of 2-aryl-3-bromobenzofurans and symmetrically/unsymmetrically substituted 2,3-diarylbenzofurans in good to high yields. Additional threefold arylation of 2,3,5-tribromobenzofuran under Pd-catalyzed conditions afforded 2,3,5-triarylbenzofurans in high yields and in short reaction duration. The promising synthetic potential demonstrated in this study is expected to attract easy applications in structural elaborations of medicinally important benzofuran scaffolds.

## Supporting Information

File 1 Experimental procedures, spectroscopic and analytical data of all compounds.

File 2 ^1^H, ^13^C NMR spectra of all compounds.
