# Supplementary material for: Rapid regio- and multi-coupling reactivity of 2,3-dibromobenzofurans with atom-economic triarylbismuths under palladium catalysis
Source: Beilstein J Org Chem. 2016 Sep 22;12:2065–76. doi: 10.3762/bjoc.12.195 (PMC5082443; doi:10.3762/bjoc.12.195)
Supplement: File 1 — Experimental procedures, spectroscopic and analytical data of all compounds. [file Beilstein_J_Org_Chem-12-2065-s001.pdf]

Supporting Information

for

Rapid regio- and multi-coupling reactivity of 2,3-dibromobenzofurans with atom-economic triarylbismuths under palladium catalysis

Maddali L. N. Rao,* Jalindar B. Talode and Venneti N. Murty

Address: Department of Chemistry, Indian Institute of Technology Kanpur, Kanpur-208016, U.P., India, Tel/ Fax: +91-512-2597532

Email: Maddali L. N. Rao - maddali@iitk.ac.in

*Corresponding author

Experimental procedures, spectroscopic and analytical data of all compounds

Contents

1. Experimental procedures for substrate preparation………………………………………………………………. S02
2. Characterization data of compounds (**1.1–1.6**)……………………………………………………………….. S03
3. Experimental procedures for cross-coupling reactions……………………………………………………… S04
4. Characterization data of compounds (**2.1–2.27**, **3.1–3.7**, **4.1–4.3** and **5.1–5.4**)…………………………… S07
5. Crystallographic data for compound **3.1** (CCDC-1425338)………………………………………………… S16
6. References……………………………………………………………………………………………S17

1. Experimental procedures for substrate preparation

1.1 General

The starting materials 2,3-di and 2,3,5-tribromobenzofurans were prepared according to literature procedures [1,2]. Triarylbismuth compounds were made following the known procedures [3]. ^{1}H NMR and ^{13}C NMR spectra were recorded on JEOL–Lambda (500 MHz and 400 MHz) spectrometers. HRMS was measured with Waters ESI-QTOF and CAB155 GCT Premier Analyzer instruments. IR spectra were recorded on a Bruker Vector 22 FT-IR spectrometer. The X-ray diffraction data for **3.1 (**CCDC-1425338) [4] was collected with a Bruker Apex Smart diffractometer and using graphite-monochromated Mo Kα radiation (λ = 0.71073 Å). Its structure was solved by direct methods using SHELXS-97 and refined by full matrix least squares on F^2 using SHELXS-97 [5]. All hydrogen atoms were included in idealized positions and a riding model was used. All non-hydrogen atoms were refined with anisotropic displacement parameters.

1.2 Representative procedure for preparation of starting compounds (1.1–1.6).

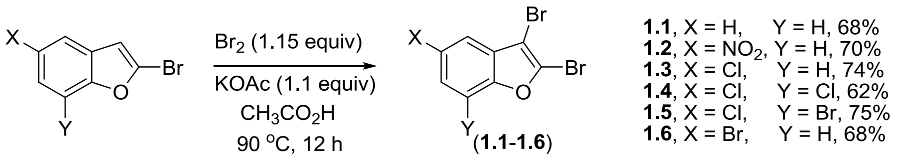

To an oven-dried two-neck round-bottom flask was added 2-bromobenzofuran (20.3 mmol, 1 equiv, 4 g) in acetic acid (20 mL) followed by KOAc (22.3 mmol, 1.1 equiv, 2.19 g). To this, bromine (23.34 mmol, 1.15 equiv, 1.2 mL) in acetic acid (20 mL) was added slowly. The reaction mixture was stirred for 12 h at 90 ℃. It was brought to rt and quenched with 5 % aq. $NaHSO_3$ solution and extracted with diethyl ether. The organic extract was washed with brine, dried over anhydrous $MgSO_4$ and concentrated. The crude mixture was subjected to silica gel column chromatography using hexane as eluent to afford 2,3-dibromobenzofuran **1.1** as colourless liquid (3.8 g, 68%).

2. Characterization data of compounds (1.1–1.6):

2.1. *2,3-Dibromobenzofuran* (**1.1**). [1,2] Colourless liquid (3.8 g, 68%); R_f (Hexane) 0.84; ^{1}H NMR (400 MHz, $CDCl_3$): δ 7.48-7.43 (m, 2H, CH_{ar}), 7.35-7.30 (m, 2H, CH_{ar}) ppm. ^{13}C NMR (125 MHz, $CDCl_3$): δ 154.73, 128.49, 128.13, 125.41, 123.98, 119.27, 111.23, 99.96 ppm. IR (neat, cm^{-1}): 3132, 3088, 1577, 1531, 1431, 1163, 918, 792. HRMS (EI$^+$): [M]$^+$ calcd for $C_8H_4Br_2O$ 273.8629; found 273.8624.

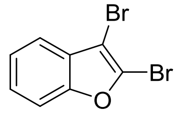

2.2. *2,3-Dibromo-5-nitrobenzofuran* (**1.2**). Yellow solid (1.9 g, 70%), mp 122-124 °C; R_f (Hexane) 0.65; ^{1}H NMR (500 MHz, $CDCl_3$): δ 8.39 (d, J = 2.3 Hz, 1H, CH_{ar}), 8.26 (dd, J = 9.15 Hz, 2.3 Hz, 1H, CH_{ar}), 7.56 (d, J = 9.15 Hz, 1H, CH_{ar}) ppm. ^{13}C NMR (125 MHz, $CDCl_3$): δ 157.19, 144.91, 132.17, 128.76, 121.10, 115.89, 111.93, 100.84 ppm. IR (KBr, cm^{-1}): 3110, 2851, 1541, 1523, 1439, 1349, 1272, 1257, 1072, 998, 820, 729, 695. HRMS (EI$^+$): [M]$^+$ calcd for $C_8H_3Br_2NO_3$ 318.8480; found 318.8482.

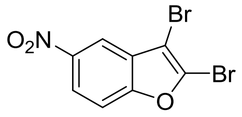

2.3. *2,3-Dibromo-5-chlorobenzofuran* (**1.3**). Colourless solid (1.5 g, 74%), mp 104-106 °C; R_f (Hexane) 0.86; ^{1}H NMR (400 MHz, $CDCl_3$): δ 7.45 (d, J = 1.84 Hz, 1H, CH_{ar}), 7.37 (d, J = 8.68 Hz, 1H, CH_{ar}), 7.28 (dd, J = 8.94 Hz, 2.06 Hz, 1H, CH_{ar}) ppm. ^{13}C NMR (100 MHz, $CDCl_3$): δ 153.09, 130.10, 129.96, 129.40, 125.71, 119.03, 112.34, 99.38 ppm. IR (KBr, cm^{-1}): 3083, 2922, 1587, 1446, 1430, 1254, 1134, 1108, 1002, 798, 706. HRMS (EI$^+$): [M]$^+$ calcd for $C_8H_3Br_2ClO$ 307.8239; found 307.8239.

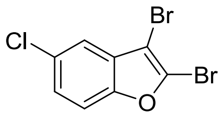

2.4. *2,3-Dibromo-5,7-dichlorobenzofuran* (**1.4**). Colourless solid (1.2 g, 62%), mp 112-114 °C; R_f (Hexane) 0.86; ^{1}H NMR (400 MHz, $CDCl_3$): δ 7.36 (d, J = 2.72 Hz, 1H, CH_{ar}), 7.33 (d, J = 2.32 Hz, 1H, CH_{ar}) ppm. ^{13}C NMR (100 MHz, $CDCl_3$): δ 149.18, 131.15, 130.35, 130.26, 125.68, 117.74, 117.39, 99.98 ppm. IR (KBr, cm^{-1}): 3077, 2924, 1577, 1445, 1398, 1276, 1128, 1082, 936, 849. HRMS (EI$^+$): [M]$^+$ calcd for $C_8H_2Br_2Cl_2O$ 341.7849; found 341.7847.

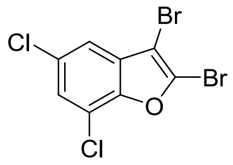

2.5. *2,3,7-Tribromo-5-chlorobenzofuran* (**1.5**). Colourless solid (1.6 g, 75%), mp 140-142 °C; R_f (Hexane) 0.88; ^{1}H NMR (400 MHz, $CDCl_3$): δ 7.48 (d, J = 1.8 Hz, 1H, CH_{ar}), 7.39 (d, J = 1.8 Hz, 1H, CH_{ar}) ppm. ^{13}C NMR (100 MHz, $CDCl_3$): δ 150.58, 131.12, 130.49, 129.96, 128.31, 118.29, 104.26, 100.06 ppm. IR (KBr, cm^{-1}): 3074, 2924, 1570, 1443, 1393, 1125, 1084, 849, 749, 730. HRMS (EI$^+$): [M]$^+$ calcd for $C_8H_2Br_3ClO$ 385.7344; found 385.7345.

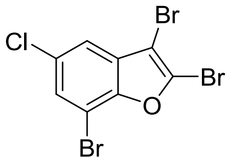

2.6. *2,3,5-Tribromobenzofuran* (**1.6**). [1] Colourless solid (1.29 g, 68%); mp 100-102 °C; R_f (Hexane) 0.85; ^{1}H NMR (400 MHz, $CDCl_3$): δ 7.60 (s, 1H, CH_{ar}), 7.42 (dd, J = 8.92 Hz, J = 1.6 Hz, 1H, CH_{ar}), 7.32 (d, J = 8.68 Hz, 1H, CH_{ar}) ppm. ^{13}C NMR (100 MHz, $CDCl_3$): δ 153.48, 129.99, 129.92, 128.43, 122.07, 117.31, 112.74, 99.19 ppm. IR (KBr, cm^{-1}): 3088, 2922, 1537, 1443, 1428, 1257, 1135, 1113, 998, 800, 682. HRMS (EI^+): $[M]^+$ calcd for $C_8H_3Br_3O$ 351.7734; found 351.7734.

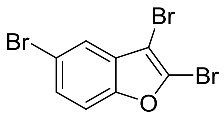

3. Experimental procedures for cross-coupling reactions:

3.1 Representative mono-arylation procedure for Table 1 and 2:

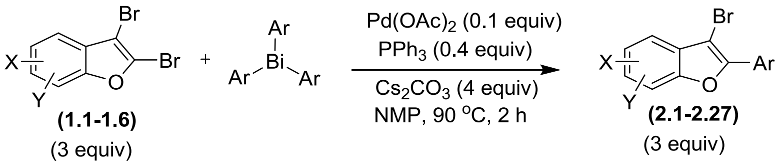

A hot-oven dried Schlenk tube was charged with 2,3-dibromobenzofuran **1.1** (0.825 mmol, 0.228 g) followed by tri-(*p*-anisyl)bismuth (0.25 mmol, 0.133 g), Cs_2CO_3 (1.0 mmol, 0.326 g), $Pd(OAc)_2$ (0.025 mmol, 0.0056 g), PPh_3 (0.1 mmol, 0.0262 g) and NMP (3 mL) solvent under N_2 atmosphere. The reaction mixture was stirred in an oil bath at 90 ^{o}C for 2 h. It was brought to rt and quenched with water and extracted with ethyl acetate. The organic extract was washed with brine, dried over anhydrous $MgSO_4$ and concentrated. The crude mixture was subjected to silica gel column chromatography using hexane as eluent to afford compound **2.1** as colourless solid (0.216 g, 95%).

3.2 Representative bis-arylation procedure for Table 3 and 4:

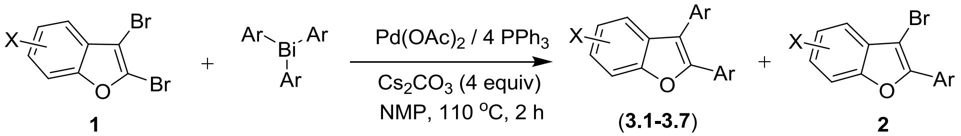

A hot-oven dried Schlenk tube was charged with 2,3-dibromobenzofuran **1.1** (0.375 mmol, 0.104 g) followed by tri-(*p*-tolyl)bismuth (0.25 mmol, 0.12 g), Cs_2CO_3 (1.0 mmol, 0.326 g), $Pd(OAc)_2$ (0.025 mmol, 0.0056 g), PPh_3 (0.1 mmol, 0.0262 g) and NMP (3 mL) solvent under N_2

atmosphere. The reaction mixture was stirred in an oil bath at 110 °C for 2 h. It was subjected to the workup procedure and chromatographic separation as given above for Table 2, to obtain **3.1** as colourless solid (0.086 g, 77%). In this reaction, minor amount of mono-arylated **2.3** was obtained as colourless solid (0.002 g, 2%).

3.3 Cross-coupling procedure for Table 5:

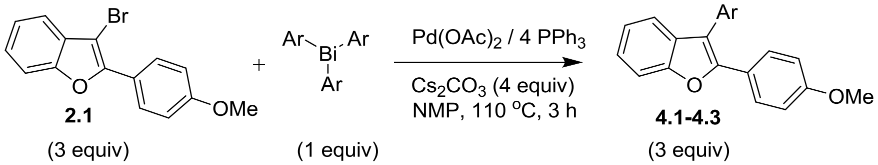

A hot-oven dried Schlenk tube was charged with compound **2.1** (0.375 mmol, 0.114 g) followed by tri-(*p*-tolyl)bismuth (0.125 mmol, 0.061 g), Cs_2CO_3 (0.5 mmol, 0.163 g), $Pd(OAc)_2$ (0.0125 mmol, 0.0028 g), PPh_3 (0.05 mmol, 0.0132 g) and NMP (3 mL) solvent under N_2 atmosphere. The reaction mixture was stirred in an oil bath at 110 °C for 3 h. It was subjected to the workup procedure as given above for Table 2 followed by silica gel chromatographic separation using 2% ethyl acetate in hexane as eluent and product **4.1** was obtained as colourless solid (0.098 g, 83%).

3.4 One-pot cross-coupling procedure for Table 6:

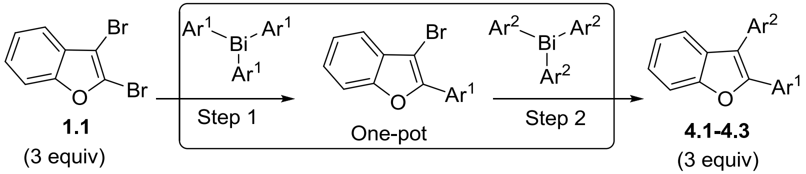

Step 1: A hot-oven dried Schlenk tube was charged with 2,3-dibromobenzofuran **1.1** (0.375 mmol, 0.104 g) followed by tri-(*p*-anisyl)bismuth (0.125 mmol, 0.067 g), Cs_2CO_3 (0.5 mmol, 0.163 g), $Pd(OAc)_2$ (0.0125 mmol, 0.0028 g), PPh_3 (0.05 mmol, 0.0132 g) and NMP (3 mL) solvent under N_2 atmosphere. The reaction mixture was stirred in an oil bath at 90 °C for 2 h. **Step 2:** The reaction mixture was brought to rt and was added tri-(*p*-tolyl)bismuth (0.125 mmol, 0.062 g), Cs_2CO_3 (0.25 mmol, 0.082 g), $Pd(OAc)_2$ (0.0062 mmol, 0.0014 g), PPh_3 (0.025 mmol, 0.0066 g) and NMP (1 mL) solvent under N_2 atmosphere. The combined reaction mixture was stirred again at 110 °C for 2 h. After that, it was subjected

to the workup procedure as given above for Table 2. The crude mixture was subjected to silica gel column chromatography using 2% ethyl acetate in hexane to afford compound **4.1** as colourless solid (0.085 g, 72%).

3.5 Cross-coupling procedure for Table 7:

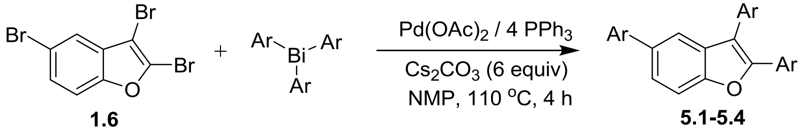

A hot-oven dried Schlenk tube was charged with 2,3,5-tribromobenzofuran **1.6** (0.25 mmol, 0.089 g) followed by tri-(*p*-anisyl)bismuth (0.25 mmol, 0.133 g), Cs_2CO_3 (1.5 mmol, 0.49 g), $Pd(OAc)_2$ (0.025 mmol, 0.0056 g), PPh_3 (0.1 mmol, 0.0262 g) and NMP (3 mL) solvent under N_2 atmosphere. The reaction mixture was stirred in an oil bath at 110 ^{o}C for 4 h. It was subjected to the workup procedure as given above for Table 2 and the crude product mixture was subjected to silica gel column chromatography using 2% ethyl acetate/hexane as eluent to afford compound **5.1** as colourless solid (0.086 g, 79%).

4. Characterization data of compounds (**2.1**–**2.27**, **3.1**–**3.7**, **4.1**–**4.3 and 5.1**–**5.4**).

4.1 Compound **2.1**. [6a] Colourless solid (0.216 g, 95%), mp 68-70 °C; R_f (Hexane) 0.65; ^{1}H NMR (500 MHz, $CDCl_3$): δ = 8.12 (d, *J* = 9.15 Hz, 2H, CH_{ar}), 7.54-7.47 (m, 2H, CH_{ar}), 7.34-7.29 (m, 2H, CH_{ar}), 7.02 (d, *J* = 9.2 Hz, 2H, CH_{ar}), 3.88 (s, 3H, $OC\underline{H}_3$) ppm. ^{13}C NMR (125 MHz, $CDCl_3$): δ = 160.2, 152.9, 150.5, 129.7, 128.3, 125.1, 123.3, 122.2, 119.6, 114.0, 111.1, 92.2, 55.4 ppm. IR (KBr): ṽ = 2977, 1610, 1503, 1451, 1251, 1179, 1048, 740, 654 cm^{-1}. HRMS (EI$^+$): [M]$^+$ calcd for $C_{15}H_{11}BrO_2$ 301.9942; found 301.9947.

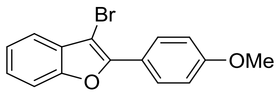

4.2 Compound **2.2**. [6b] Colourless solid (0.18 g, 88%), mp 62-64 °C; R_f (Hexane) 0.75; ^{1}H NMR (500 MHz, $CDCl_3$): δ = 8.20-8.18 (m, 2H, CH_{ar}), 7.58-7.56 (m, 1H, CH_{ar}), 7.53-7.49 (m, 3H, CH_{ar}), 7.45-7.41 (m, 1H, CH_{ar}), 7.39-7.32 (m, 2H, CH_{ar}) ppm. ^{13}C NMR (125 MHz, $CDCl_3$): δ = 153.1, 150.3, 129.6, 129.5, 129.0, 128.6, 126.7, 125.6, 123.5, 119.9, 111.3, 93.8 ppm. IR (KBr): ṽ = 2922, 2853, 1503, 1451, 1205, 1075, 986, 814, 745, 712 cm^{-1}. HRMS (EI$^+$): [M]$^+$ calcd for $C_{14}H_9BrO$ 271.9837; found 271.9835.

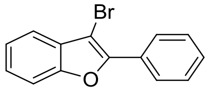

4.3 Compound **2.3**. [6a] Colourless solid (0.175 g, 81%), mp 70-72 °C; R_f (Hexane) 0.72; ^{1}H NMR (500 MHz, $CDCl_3$): δ = 8.07 (d, *J* = 8.2 Hz, 2H, CH_{ar}), 7.56-7.49 (m, 2H, CH_{ar}), 7.36-7.30 (m, 4H, CH_{ar}), 2.42 (s, 3H, CH_3) ppm. ^{13}C NMR (125 MHz, $CDCl_3$): δ = 153.0, 150.6, 139.2, 129.6, 129.3, 126.7, 125.3, 123.4, 119.7, 111.2, 93.1, 21.5 ppm. IR (KBr): ṽ = 2923, 1610, 1452, 1252, 1180, 1073, 985, 742 cm^{-1}. HRMS (EI$^+$): [M]$^+$ calcd for $C_{15}H_{11}BrO$ 285.9993; found 285.9991.

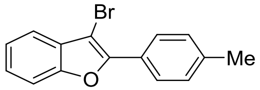

4.4 Compound **2.4**. Colourless solid (0.201 g, 85%), mp 122-124 °C; R_f (Hexane) 0.68; ^{1}H NMR (500 MHz, $CDCl_3$): δ = 8.10 (d, *J* = 8.85 Hz, 2H, CH_{ar}), 7.54-7.47 (m, 2H, CH_{ar}), 7.34-7.29 (m, 2H, CH_{ar}), 7.01 (d, *J* = 8.85 Hz, 2H, CH_{ar}), 4.11 (q, *J* = 6.95 Hz, 2H, $C\underline{H}_2CH_3$), 1.46 (t, *J* = 7.02 Hz, 3H, $CH_2C\underline{H}_3$) ppm. ^{13}C NMR (125 MHz, $CDCl_3$): δ = 159.6, 152.9, 150.5, 129.7, 128.3, 125.1, 123.3, 122.0, 119.5, 114.5, 111.1, 92.1, 63.6, 14.8 ppm. IR (KBr): ṽ = 2977, 2873, 1610, 1503, 1180, 1071, 837, 740 cm^{-1}. HRMS (EI$^+$): [M]$^+$ calcd for $C_{16}H_{13}BrO_2$ 316.0099; found 316.0096.

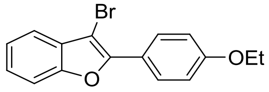

4.5 Compound **2.5**. Colourless solid (0.204 g, 89%), mp 104-106 °C; R_f (Hexane) 0.83; ^{1}H NMR (500 MHz, $CDCl_3$): δ = 8.12 (d, *J* = 8.55 Hz, 2H, CH_{ar}), 7.57-7.49 (m, 2H, CH_{ar}), 7.46 (d, *J* = 8.6 Hz, 2H, CH_{ar}), 7.39-7.31 (m, 2H, CH_{ar}) ppm. ^{13}C NMR (125 MHz, $CDCl_3$): δ = 153.1, 149.2, 134.9, 129.4, 128.9, 128.0, 127.9, 125.9, 123.6, 120.0, 111.3, 94.3 ppm. IR (KBr): ṽ = 1486, 1449, 1250, 1096, 1072, 823, 735 cm^{-1}. HRMS (EI$^+$): [M]$^+$ calcd for $C_{14}H_8BrClO$ 305.9447; found 305.9447.

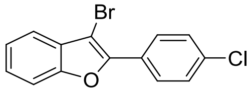

4.6 Compound **2.6**. Colourless solid (0.198 g, 86%); mp 72-74 °C; R_f (Hexane) 0.82; ^{1}H NMR (500 MHz, $CDCl_3$): δ = 8.17 (s, 1H, CH_{ar}), 8.09 (d, J = 7.45 Hz, 1H, CH_{ar}), 7.58-7.50 (m, 2H, CH_{ar}), 7.44-7.32 (m, 4H, CH_{ar}) ppm. ^{13}C NMR (125 MHz, $CDCl_3$): δ = 153.2, 148.7, 134.7, 131.2, 129.9, 129.4, 129.0, 126.5, 126.1, 124.7, 123.7, 120.1, 111.4, 94.9 ppm. IR (KBr): ṽ = 1595, 1452, 1205, 1061, 996, 837, 737 cm^{-1}. HRMS (EI^+): $[M]^+$ calcd for $C_{14}H_8BrClO$ 305.9447; found 305.9441.

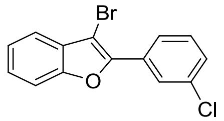

4.7 Compound **2.7**. Pale yellow liquid (0.176 g, 82%); R_f (Hexane) 0.62; ^{1}H NMR (500 MHz, $CDCl_3$): δ = 7.79 (d, J = 7.75 Hz, 1H, CH_{ar}), 7.73-7.72 (m, 1H, CH_{ar}), 7.57 (d, J = 7.45 Hz, 1H, CH_{ar}), 7.52 (d, J = 8.05 Hz, 1H, CH_{ar}), 7.43-7.31 (m, 3H, CH_{ar}), 6.99-6.97 (m, 1H, CH_{ar}), 3.91 (s, 3H, $-OCH_3$) ppm. ^{13}C NMR (125 MHz, $CDCl_3$): δ = 159.6, 153.1, 150.1, 130.7, 129.7, 129.5, 125.7, 123.5, 119.9, 119.2, 115.1, 111.8, 111.3, 94.1, 55.4 ppm. IR (neat): ṽ = 2977, 1610, 1503, 1251, 1072, 1048, 984, 740 cm^{-1}. HRMS (EI^+): $[M]^+$ calcd for $C_{15}H_{11}BrO_2$ 301.9942; found 301.9946.

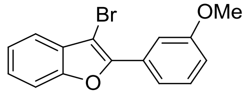

4.8 Compound **2.8**. Colourless solid (0.202 g, 93%), mp 64-66 °C; R_f (Hexane) 0.82; ^{1}H NMR (500 MHz, $CDCl_3$): δ = 8.18-8.15 (m, 2H, CH_{ar}), 7.56 (d, J = 7.45 Hz, 1H, CH_{ar}), 7.50 (d, J = 7.75 Hz, 1H, CH_{ar}), 7.38-7.31 (m, 2H, CH_{ar}), 7.21-7.17 (m, 2H, CH_{ar}) ppm. ^{13}C NMR (125 MHz, $CDCl_3$): δ = 162.9 (d, J_{C-F} = 248.4 Hz), 153.1, 149.5, 129.5, 128.7 (d, J_{C-F} = 8.4 Hz), 125.8, 125.6, 123.6, 119.9, 115.7 (d, J_{C-F} = 21.6 Hz), 111.2, 93.5 ppm. IR (KBr): ṽ = 3069, 1603, 1501, 1450, 1230, 1073, 985, 834, 743 cm^{-1}. HRMS (EI^+): $[M]^+$ calcd for $C_{14}H_8BrFO$ 289.9743; found 289.9742.

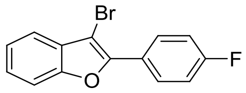

4.9 Compound **2.9**. Colourless solid (0.196 g, 90%), mp 60-62 °C; R_f (Hexane) 0.83; ^{1}H NMR (500 MHz, $CDCl_3$): δ = 7.99 (d, J = 7.0 Hz, 1H, CH_{ar}), 7.91-7.89 (m, 1H, CH_{ar}), 7.57 (d, J = 7.6 Hz, 1H, CH_{ar}), 7.52-7.43 (m, 2H, CH_{ar}), 7.40-7.32 (m, 2H, CH_{ar}), 7.13-7.09 (m, 1H, CH_{ar}) ppm. ^{13}C NMR (125 MHz, $CDCl_3$): δ = 162.8 (d, J_{C-F} = 243.5 Hz), 153.1, 148.9, 131.5, 130.2 (d, J_{C-F} = 8.4 Hz), 129.4, 126.1, 123.7, 122.3, 120.1, 115.9 (d, J_{C-F} = 21.6 Hz), 113.5 (d, J_{C-F} = 24 Hz), 111.4, 94.9 ppm. IR (KBr): ṽ = 2922, 1603, 1501, 1451, 1230, 1072, 985, 832, 744 cm^{-1}. HRMS (EI^+): $[M]^+$ calcd for $C_{14}H_8BrFO$ 289.9743; found 289.9743.

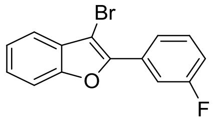

4.10 Compound **2.10**. Pale yellow liquid (0.17 g, 79%); R_f (Hexane) 0.72; ^{1}H NMR (500 MHz, $CDCl_3$): δ = 8.01-7.98 (m, 2H, CH_{ar}), 7.57-7.50 (m, 2H, CH_{ar}), 7.41-7.31 (m, 3H, CH_{ar}), 7.24 (d, J = 7.35 Hz, 1H, CH_{ar}), 2.46 (s, 3H, CH_3) ppm. ^{13}C NMR (125 MHz, $CDCl_3$): δ = 153.1, 150.5, 138.3, 129.9, 129.6, 129.4, 128.5, 127.3, 125.5, 124.0, 123.4, 119.8, 111.2, 93.7, 21.6 ppm. IR (neat): ṽ = 2921, 1503, 1451, 1253, 1076, 986, 814, 747 cm^{-1}. HRMS (EI^+): $[M]^+$ calcd for $C_{15}H_{11}BrO$ 285.9993; found 285.9991.

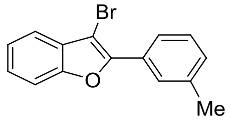

4.11 Compound **2.11**. Colourless solid (0.218 g, 92%), mp 110-112 ^{o}C; R_f (Hexane) 0.5; ^{1}H NMR (500 MHz, $CDCl_3$): δ = 7.72-7.70 (m, 1H, CH_{ar}), 7.66 (d, *J* = 1.85 Hz, 1H, CH_{ar}), 7.54-7.46 (m, 2H, CH_{ar}), 7.35-7.29 (m, 2H, CH_{ar}), 6.93 (d, *J* = 8.25 Hz, 1H, CH_{ar}), 6.04 (s, 2H, $OC\underline{H}_2$) ppm. ^{13}C NMR (125 MHz, $CDCl_3$): δ = 152.9, 150.1, 148.3, 147.9, 129.6, 125.3, 123.5, 123.4, 121.4, 119.7, 111.1, 108.5, 107.1, 101.4, 92.6 ppm. IR (KBr): ṽ = 2907, 1497, 1487, 1251, 1043, 991, 860, 736 cm^{-1}. HRMS (EI^{+}): $[M]^{+}$ calcd for $C_{15}H_9BrO_3$ 315.9735; found 315.9757.

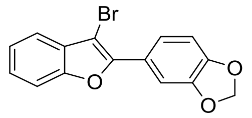

4.12 Compound **2.12**. Colourless solid (0.232 g, 93%), mp 80-82 ^{o}C; R_f (Hexane) 0.62; ^{1}H NMR (500 MHz, $CDCl_3$): δ = 8.09 (d, *J* = 8.2 Hz, 2H, CH_{ar}), 7.54-7.47 (m, 2H, CH_{ar}), 7.34-7.28 (m, 2H, CH_{ar}), 6.99 (d, *J* = 8.55 Hz, 2H, CH_{ar}), 4.66-4.61 (m, 1H, -$C\underline{H}(CH_3)_2$), 1.38 (d, *J* = 6.1 Hz, 6H, -$CH(C\underline{H}_3)_2$) ppm. ^{13}C NMR (125 MHz, $CDCl_3$): δ = 158.6, 152.9, 150.6, 129.7, 128.3, 125.0, 123.3, 121.8, 119.5, 115.7, 111.1, 92.0, 69.9, 22.0 ppm. IR (KBr): ṽ = 2979, 2922, 1611, 1500, 1255, 1245, 1073, 826, 751 cm^{-1}. HRMS (EI): $[M]^{+}$ calcd for $C_{17}H_{15}BrO_2$ 330.0255; found 330.0253.

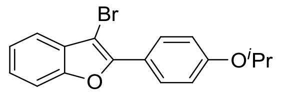

4.13 Compound **2.13**. Yellow solid (0.204 g, 82%), mp 162-164 ^{o}C; R_f (Hexane) 0.45; ^{1}H NMR (500 MHz, $CDCl_3$): δ = 8.45 (d, *J* = 2.15 Hz, 1H, CH_{ar}), 8.25 (dd, *J* = 9.0 Hz, 2.15 Hz, 1H, CH_{ar}), 8.05 (d, *J* = 8.25 Hz, 2H, CH_{ar}), 7.57 (d, *J* = 8.85 Hz, 1H, CH_{ar}), 7.32 (d, *J* = 7.9 Hz, 2H, CH_{ar}), 2.43 (s, 3H, -$C\underline{H}_3$) ppm. ^{13}C NMR (125 MHz, $CDCl_3$): δ = 155.8, 153.9, 144.6, 140.5, 130.4, 129.5, 126.9, 125.5, 121.0, 116.4, 111.7, 93.1, 21.5 ppm. IR (KBr): ṽ = 2922, 2852, 1503, 1451, 1205, 1075, 986, 814, 745 cm^{-1}. HRMS (EI^{+}): $[M]^{+}$ calcd for $C_{15}H_{10}BrNO_3$ 330.9844; found 330.9844.

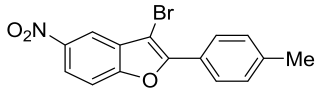

4.14 Compound **2.14**. Yellow solid (0.240 g, 88%), mp 136-138 ^{o}C; R_f (Hexane) 0.42; ^{1}H NMR (500 MHz, $CDCl_3$): δ = 8.43 (d, *J* = 2.3 Hz, 1H, CH_{ar}), 8.23 (dd, *J* = 9.2 Hz, 2.35 Hz, 1H, CH_{ar}), 8.10 (d, *J* = 9.15 Hz, 2H, CH_{ar}), 7.55 (d, *J* = 8.55 Hz, 1H, CH_{ar}), 7.02 (d, *J* = 8.6 Hz, 2H, CH_{ar}), 4.11 (q, *J* = 7.06 Hz, 2H, -$OC\underline{H}_2CH_3$), 1.46 (t, *J* = 7.15 Hz, 3H, -$OCH_2C\underline{H}_3$) ppm. ^{13}C NMR (125 MHz, $CDCl_3$): δ = 160.4, 155.7, 153.9, 144.6, 130.5, 128.6, 120.8, 120.7, 116.1, 114.7, 111.5, 91.9, 63.7, 14.7 ppm. IR (KBr): ṽ = 3099, 2981, 1612, 1518, 1503, 1341, 1253, 1181, 1045, 834, 730 cm^{-1}. HRMS (EI^{+}): $[M]^{+}$ calcd for $C_{16}H_{12}BrNO_4$ 360.9950; found 360.9958.

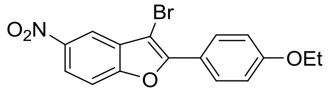

4.15 Compound **2.15**. Yellow solid (0.19 g, 76%), mp 152-154 ^{o}C; R_f (Hexane) 0.45; ^{1}H NMR (500 MHz, $CDCl_3$): δ = 8.47 (d, *J* = 2.15 Hz, 1H, CH_{ar}), 8.27 (dd, *J* = 8.85 Hz, 2.15 Hz, 1H, CH_{ar}), 7.99-7.97 (m, 2H, CH_{ar}), 7.59 (d, *J* = 8.85 Hz, 1H, CH_{ar}), 7.41 (t, *J* = 7.65 Hz, 1H, CH_{ar}), 7.29 (d, *J* = 7.60 Hz, 1H, CH_{ar}), 2.47 (s, 3H, -$C\underline{H}_3$) ppm. ^{13}C NMR (125 MHz, $CDCl_3$): δ = 155.9, 153.8, 144.6, 138.6, 130.9, 130.4, 128.7, 128.2, 127.5, 124.2, 121.2, 116.5, 111.8, 93.7, 21.6 ppm. IR (KBr): ṽ = 2920, 1621, 1525, 1341, 1183, 1063, 813, 748 cm^{-1}. HRMS (EI^{+}): $[M]^{+}$ calcd for $C_{15}H_{10}BrNO_3$ 330.9844; found 330.9845.

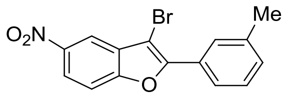

4.16 Compound **2.16**. Colourless solid (0.23 g, 91%), mp 112-114 °C; R_f (Hexane) 0.62; ^{1}H NMR (500 MHz, $CDCl_3$): δ = 8.09 (d, J = 8.85 Hz, 2H, CH_{ar}), 7.49 (d, J = 1.8 Hz, 1H, CH_{ar}), 7.38 (d, J = 8.85 Hz, 1H, CH_{ar}), 7.26 (dd, J = 8.7 Hz, 1.95 Hz, 1H, CH_{ar}), 7.01 (d, J = 8.85 Hz, 2H, CH_{ar}), 3.87 (s, 3H, $OC\underline{H}_3$) ppm. ^{13}C NMR (125 MHz, $CDCl_3$): δ = 160.5, 152.0, 151.3, 131.1, 129.1, 128.4, 125.2, 121.7, 119.2, 114.1, 112.1, 91.2, 55.4 ppm. IR (KBr): ṽ = 2925, 2834, 1609, 1504, 1262, 1182, 1035, 827, 798 cm^{-1}. HRMS (EI^+): $[M]^+$ calcd for $C_{15}H_{10}BrClO_2$ 335.9553; found 335.9556.

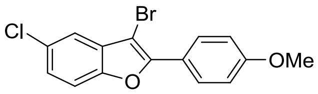

4.17 Compound **2.17**. Colourless solid (0.225 g, 82%), mp 130-132 °C; R_f (Hexane) 0.42; ^{1}H NMR (500 MHz, $CDCl_3$): δ = 7.78-7.75 (m, 1H, CH_{ar}), 7.67-7.65 (m, 2H, CH_{ar}), 7.43-7.41 (m, 1H, CH_{ar}), 7.36 (d, J = 8.55 Hz, 1H, CH_{ar}), 6.98 (d, J = 8.55 Hz, 1H, CH_{ar}), 3.99 (s, 3H, $OC\underline{H}_3$), 3.96 (s, 3H, $OC\underline{H}_3$) ppm. ^{13}C NMR (125 MHz, $CDCl_3$): δ = 151.7, 151.6, 150.1, 148.9, 131.7, 128.1, 122.3, 121.7, 120.2, 116.5, 112.6, 111.0, 109.6, 91.3, 56.0, 56.0 ppm. IR (KBr): ṽ = 2950, 1607, 1508, 1449, 1288, 1272, 1147, 1028, 995, 844, 795 cm^{-1}. HRMS (EI^+): $[M]^+$ calcd for $C_{16}H_{12}BrClO_3$ 365.9658; found 365.9665.

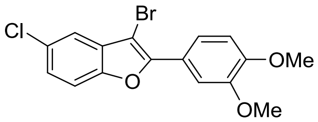

4.18 Compound **2.18**. Colourless solid (0.23 g, 87%), mp 116-118 °C; R_f (Hexane) 0.52; ^{1}H NMR (500 MHz, $CDCl_3$): δ = 7.68 (dd, J = 8.3 Hz, 1.65 Hz, 1H, CH_{ar}), 7.62 (d, J = 1.8 Hz, 1H, CH_{ar}), 7.49 (d, J = 2.15 Hz, 1H, CH_{ar}), 7.38 (d, J = 8.55 Hz, 1H, CH_{ar}), 7.27 (dd, J = 8.55 Hz, 2.1 Hz, 1H, CH_{ar}), 6.93 (d, J = 8.25 Hz, 1H, CH_{ar}), 6.05 (s, 2H, $OC\underline{H}_2$) ppm. ^{13}C NMR (125 MHz, $CDCl_3$): δ = 151.6, 151.2, 148.6, 147.9, 131.1, 129.2, 125.5, 123.0, 121.6, 119.3, 112.2, 108.6, 107.1, 101.5, 91.7 ppm. IR (KBr): ṽ = 2922, 1607, 1498, 1454, 1441, 1256, 1073, 991, 840, 794 cm^{-1}. HRMS (EI^+): $[M]^+$ calcd for $C_{15}H_8BrClO_3$ 349.9345; found 349.9345.

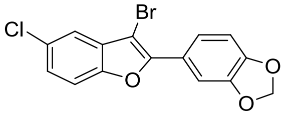

4.19 Compound **2.19**. Colourless solid (0.167 g, 63%), mp 140-142 °C; R_f (Hexane) 0.68; ^{1}H NMR (500 MHz, $CDCl_3$): δ = 8.64 (s, 1H, CH_{ar}), 8.24 (d, J = 8.8 Hz, 1H, CH_{ar}), 7.94-7.92 (m, 2H, CH_{ar}), 7.87-7.85 (m, 1H, CH_{ar}), 7.55-7.53 (m, 3H, CH_{ar}), 7.46-7.43 (m, 1H, CH_{ar}), 7.32-7.30 (m, 1H, CH_{ar}) ppm. ^{13}C NMR (125 MHz, $CDCl_3$): δ = 151.8, 151.6, 133.4, 133.0, 131.1, 129.3, 128.7, 128.3, 127.8, 127.2, 126.7, 126.4, 125.9, 123.6, 119.6, 112.4, 93.2 ppm. IR (KBr): ṽ = 3046, 1508, 1450, 1254, 1064, 995, 854, 795 cm^{-1}. HRMS (EI): $[M]^+$ calcd for $C_{18}H_{10}BrClO$ 355.9604; found 355.9607.

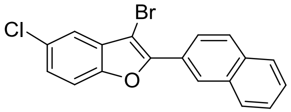

4.20 Compound **2.20**. Colourless solid (0.243 g, 87%), mp 124-126 °C; R_f (Hexane) 0.58; ^{1}H NMR (500 MHz, $CDCl_3$): δ = 8.10 (d, J = 9.2 Hz, 2H, CH_{ar}), 7.37 (d, J = 1.85 Hz, 1H, CH_{ar}), 7.30 (d, J = 1.8 Hz, 1H, CH_{ar}), 7.01 (d, J = 8.85 Hz, 2H, CH_{ar}), 3.88 (s, 3H, OCH_3) ppm. ^{13}C NMR (125 MHz, $CDCl_3$): δ = 160.8, 152.8, 147.3, 132.1, 129.3, 128.6, 125.0, 121.1, 117.9,

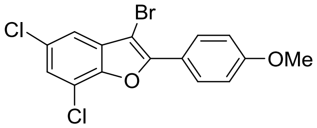

117.1, 114.2, 91.3, 55.4 ppm. IR (KBr): ṽ = 2974, 2923, 1608, 1498, 1257, 1184, 1107, 1075, 985, 750 cm^{-1}. HRMS (EI^+): $[M]^+$ calcd for $C_{15}H_9BrCl_2O_2$ 369.9163; found 369.9160.

4.21 Compound **2.21**. Colourless solid (0.21 g, 79%), mp 122-124 °C; R_f (Hexane) 0.62; ^{1}H NMR (500 MHz, $CDCl_3$): δ = 8.04 (d, *J* = 8.2 Hz, 2H, CH_{ar}), 7.39 (d, *J* = 1.85 Hz, 1H, CH_{ar}), 7.31-7.28 (m, 3H, CH_{ar}), 2.42 (s, 3H, CH_3) ppm. ^{13}C NMR (125 MHz, $CDCl_3$): δ = 152.9, 147.4, 140.1, 131.9, 129.4, 129.3, 126.8, 125.7, 125.3, 118.0, 117.2, 92.2, 21.5 ppm. IR (KBr): ṽ = 2917, 2917, 1607, 1500, 1442, 1262, 1072, 821, 721 cm^{-1}. HRMS (EI): $[M]^+$ calcd for $C_{15}H_9BrCl_2O$ 353.9214; found 353.9219.

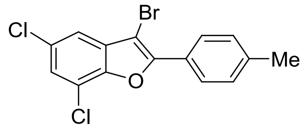

4.22 Compound **2.22**. Colourless solid (0.219 g, 70%), mp 118-120 °C ; R_f (Hexane) 0.58; ^{1}H NMR (500 MHz, $CDCl_3$): δ = 8.12 (d, *J* = 8.9 Hz, 2H, CH_{ar}), 7.46 (d, *J* = 2.0 Hz, 1H, CH_{ar}), 7.43 (d, *J* = 2.0 Hz, 1H, CH_{ar}), 7.02 (d, *J* = 8.9 Hz, 2H, CH_{ar}), 3.88 (s, 3H, OCH_3) ppm. ^{13}C NMR (125 MHz, $CDCl_3$): δ = 160.8, 152.8, 148.7, 131.7, 129.6, 128.6, 127.7, 121.1, 118.5, 114.2, 104.2, 91.4, 55.4 ppm. IR (KBr): ṽ = 3074, 2952, 1606, 1504, 1254, 1176, 1074, 851, 826 cm^{-1}. HRMS (EI^+): $[M]^+$ calcd for $C_{15}H_9Br_2ClO_2$ 413.8658; found 413.8755.

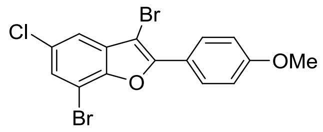

4.23 Compound **2.23**. Colourless solid (0.25 g, 73%), mp 116-118 °C; R_f (Hexane) 0.64; ^{1}H NMR (400 MHz, $CDCl_3$): δ = 8.09 (d, *J* = 9.16 Hz, 2H, CH_{ar}), 7.45 (d, *J* = 1.84 Hz, 1H, CH_{ar}), 7.42 (d, *J* = 1.80 Hz, 1H, CH_{ar}), 7.00 (d, *J* = 9.16 Hz, 2H, CH_{ar}), 3.79 (d, *J* = 6.88 Hz, 2H, CH_2), 2.18-2.07 (m, 1H, -CH), 1.06 (d, *J* = 6.40 Hz, 6H, CH_3) ppm. ^{13}C NMR (125 MHz, $CDCl_3$): δ = 160.5, 152.9, 148.7, 131.7, 129.5, 128.5, 127.6, 120.8, 118.4, 114.7, 104.1, 91.2, 74.5, 28.2, 19.2 ppm. IR (KBr): ṽ = 2954, 2914, 1611, 1502, 1266, 1175, 1033, 831 cm^{-1}. HRMS (EI^+): $[M]^+$ calcd for $C_{18}H_{15}Br_2ClO_2$ 455.9127; found 455.9128.

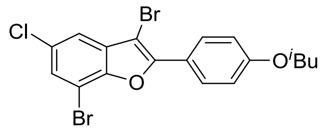

4.24 Compound **2.24**. Colourless solid (0.204 g, 68%), mp 140-142 °C; R_f (Hexane) 0.72; ^{1}H NMR (500 MHz, $CDCl_3$): δ = 8.06 (d, *J* = 8.25 Hz, 2H, CH_{ar}), 7.48 (d, *J* = 2.15 Hz, 1H, CH_{ar}), 7.45 (d, *J* = 1.85 Hz, 1H, CH_{ar}), 7.31 (d, *J* = 7.95 Hz, 2H, CH_{ar}), 2.43 (s, 3H, $-CH_3$) ppm. ^{13}C NMR (125 MHz, $CDCl_3$): δ = 153.0, 148.8, 140.2, 131.6, 129.6, 129.4, 128.0, 126.9, 125.7, 118.7, 104.3, 92.4, 21.6 ppm. IR (KBr): ṽ = 2919, 1591, 1503, 1457, 1220, 1078, 815, 756 cm^{-1}. HRMS (EI^+): $[M]^+$ calcd for $C_{15}H_9Br_2ClO$ 397.8709; found 397.8706.

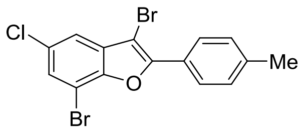

4.25 Compound **2.25**. [1] Colourless solid (0.263 g, 92%), mp 130-132 °C; R_f (Hexane) 0.6; ^{1}H NMR (500 MHz, $CDCl_3$): δ = 8.09 (d, *J* = 8.55 Hz, 2H, CH_{ar}), 7.65 (s, 1H, CH_{ar}), 7.40 (d, *J* = 8.55 Hz, 1H, CH_{ar}), 7.34 (d, *J* = 8.55 Hz, 1H, CH_{ar}), 7.01 (d, *J* =

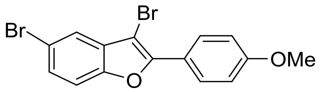

8.55 Hz, 2H, CH_{ar}), 3.88 (s, 3H, OCH_3) ppm. ^{13}C NMR (125 MHz, $CDCl_3$): δ = 160.5, 151.8, 151.7, 131.7, 128.4, 128.0, 122.3, 121.6, 116.5, 114.1, 112.6, 91.0, 55.4 ppm. IR (KBr): ṽ = 2834, 1606, 1503, 1438, 1257, 1177, 1029, 990, 830, 785 cm^{-1}. HRMS (EI^+): $[M]^+$ calcd for $C_{15}H_{10}Br_2O_2$ 379.9048; found 379.9049.

4.26 Compound **2.26**. Colourless solid (0.242 g, 87%), mp 118-120 °C; R_f (Hexane) 0.72; ^{1}H NMR (500 MHz, $CDCl_3$): δ = 8.16-8.13 (m, 2H, CH_{ar}), 7.68 (d, J = 2.15 Hz, 1H, CH_{ar}), 7.46-7.35 (m, 2H, CH_{ar}), 7.21-7.17 (m, 2H, CH_{ar}) ppm. ^{13}C NMR (125 MHz, $CDCl_3$): δ = 163.3 (d, $J_{C\text{-}F}$ = 248.36 Hz), 151.9, 150.8, 131.4, 128.9 (d, $J_{C\text{-}F}$ = 8.4 Hz), 128.6, 125.3, 122.6, 116.7, 115.9 (d, $J_{C\text{-}F}$ = 21.6 Hz), 112.8, 92.4 ppm. IR (KBr): ṽ = 3084, 1606, 1501, 1440, 1241, 1071, 991, 835, 685 cm^{-1}. HRMS (EI^+): $[M]^+$ calcd for $C_{14}H_7Br_2FO$ 367.8848; found 367.8848.

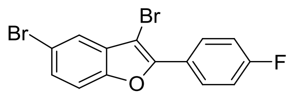

4.27 Compound **2.27**. Colourless solid (0.245 g, 77%), mp 108-110 °C; R_f (Hexane) 0.64; ^{1}H NMR (500 MHz, $CDCl_3$): δ = 8.07 (d, J = 8.85 Hz, 2H, CH_{ar}), 7.64 (d, J = 1.8 Hz, 1H, CH_{ar}), 7.40 (dd, J = 8.55 Hz, 2.15 Hz, 1H, CH_{ar}), 7.33 (d, J = 8.55 Hz, 1H, CH_{ar}), 7.00 (d, J = 9.15 Hz, 2H, CH_{ar}), 3.79 (d, J = 6.7 Hz, 2H, $-OCH_2$), 2.15-2.09 (m, 1H, -CH), 1.05 (d, J = 6.75 Hz, 6H, $-CH_3$) ppm. ^{13}C NMR (125 MHz, $CDCl_3$): δ = 160.2, 151.9, 151.7, 131.7, 128.4, 127.9, 122.2, 121.3, 116.5, 114.6, 112.6, 90.9, 74.5, 28.3, 19.2 ppm. IR (KBr): ṽ = 2956, 2912, 1608, 1502, 1452, 1252, 1174, 1033, 987, 826, 801 cm^{-1}. HRMS (EI^+): $[M]^+$ calcd for $C_{18}H_{16}Br_2O_2$ 421.9517; found 421.9514.

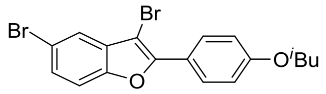

4.28 Compound **3.1**. [6c] Colourless solid (0.086 g, 77%), mp 124-126 °C; R_f (2% EtOAc/Hexane) 0.62; ^{1}H NMR (500 MHz, $CDCl_3$): δ = 7.56 (d, J = 8.25 Hz, 2H, CH_{ar}), 7.53 (d, J = 8.2 Hz, 1H, CH_{ar}), 7.49 (d, J = 7.9 Hz, 1H, CH_{ar}), 7.39 (d, J = 7.65 Hz, 2H, CH_{ar}), 7.32-7.26 (m, 3H, CH_{ar}), 7.22 (t, J = 7.47 Hz, 1H, CH_{ar}), 7.13 (d, J = 7.95 Hz, 2H, CH_{ar}), 2.44 (s, 3H, CH_3), 2.35 (s, 3H, CH_3) ppm. ^{13}C NMR (125 MHz, $CDCl_3$): δ = 153.9, 150.6, 138.3, 137.2, 130.4, 129.9, 129.6, 129.6, 129.1, 127.9, 126.9, 124.4, 122.7, 119.9, 116.7, 111.0, 21.4 ppm. IR (KBr): ṽ = 3033, 2917, 1520, 1499, 1453, 1257, 1067, 964, 816, 752, 516 cm^{-1}. HRMS (EI): $[M]^+$ calcd for $C_{22}H_{18}O$ 298.1358; found 298.1353.

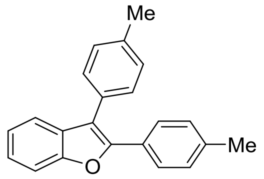

4.29 Compound **3.2**. [6d] Colourless solid (0.086 g, 85%), mp 120-122 °C; R_f (2% EtOAc/Hexane) 0.68; ^{1}H NMR (500 MHz, $CDCl_3$): δ = 7.68-7.66 (m, 2H, CH_{ar}), 7.57 (d, J = 8.2 Hz, 1H, CH_{ar}), 7.53-7.46 (m, 5H, CH_{ar}), 7.43-7.40 (m, 1H, CH_{ar}), 7.35-7.29 (m, 4H, CH_{ar}), 7.26-7.23 (m, 1H, CH_{ar}) ppm. ^{13}C NMR (125 MHz, $CDCl_3$): δ = 154.0, 150.5, 132.8, 130.7, 130.2, 129.8, 129.0, 128.4, 128.3, 127.6, 127.0, 124.7, 122.9, 120.0, 117.5, 111.1 ppm. IR (KBr): ṽ = 2922, 2853, 1502, 1451, 1231, 1205, 1075, 986, 832, 744 cm^{-1}. HRMS (EI): $[M]^+$ calcd for $C_{20}H_{14}O$ 270.1045; found 270.1041.

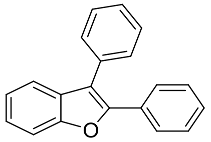

4.30 Compound **3.3**. [6c] Yellow liquid (0.081 g, 72%); R_f (2% EtOAc/Hexane) 0.62; ^{1}H NMR (500 MHz, $CDCl_3$): δ = 7.58-7.55 (m, 2H, CH_{ar}), 7.51 (d, J = 7.95 Hz, 1H, CH_{ar}), 7.42 (d, J = 8.25 Hz, 1H, CH_{ar}), 7.37-7.29 (m, 4H, CH_{ar}), 7.25-7.21 (m, 2H, CH_{ar}), 7.20-7.16 (m, 1H, CH_{ar}), 7.11 (d, J = 7.65 Hz, 1H, CH_{ar}), 2.40 (s, 3H, CH_3), 2.33 (s, 3H, CH_3) ppm. ^{13}C NMR (125 MHz, $CDCl_3$): δ = 153.9, 150.6, 138.5, 138.0, 132.8, 130.6, 130.3, 130.3, 129.1, 128.8, 128.3, 128.2, 127.5, 126.8, 124.5, 124.2, 122.8, 120.1, 117.5, 111.0, 21.5 ppm. IR (neat): ṽ = 3027, 2919, 1597, 1520, 1500, 1452, 1255, 1066, 964, 819, 746 cm^{-1}. HRMS (EI): $[M]^+$ calcd for $C_{22}H_{18}O$ 298.1358; found 298.1342.

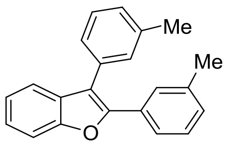

4.31 Compound **3.4**. Colourless solid (0.132 g, 72%), mp 108-110 ^oC; R_f (2% EtOAc/Hexane) 0.58; ^{1}H NMR (500 MHz, $CDCl_3$): δ = 7.62 (d, J = 9.2 Hz, 2H, CH_{ar}), 7.53-7.28 (m, 15H, CH_{ar}), 7.22 (t, J = 7.95 Hz, 1H, CH_{ar}), 7.09 (d, J = 8.55 Hz, 2H, CH_{ar}), 6.93 (d, J = 8.55 Hz, 2H, CH_{ar}), 5.14 (s, 2H, OCH_2), 5.08 (s, 2H, OCH_2) ppm. ^{13}C NMR (125 MHz, $CDCl_3$): δ = 158.8, 158.2, 153.8, 150.4, 136.8, 136.7, 130.9, 130.5, 128.6, 128.4, 128.1, 127.6, 127.5, 126.4, 125.4, 124.2, 123.7, 122.7, 119.7, 115.6, 115.3, 115.2, 114.8, 110.9, 70.1, 70.0 ppm. IR (KBr): ṽ = 2957, 2913, 2871, 1608, 1518, 1501, 1246, 1173, 1032, 838, 747 cm^{-1}. HRMS (ESI): $[M+H]^+$ calcd for $C_{34}H_{27}O_3$ 483.1660; found 483.1668.

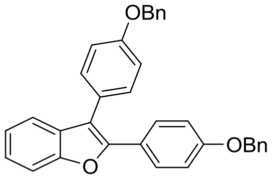

4.32 Compound **3.5**. [6c] Colourless solid (0.087 g, 76%), mp 118-120 ^oC; R_f (Hexane) 0.78; ^{1}H NMR (500 MHz, $CDCl_3$): δ = 7.62-7.59 (m, 2H, CH_{ar}), 7.54 (d, J = 8.25 Hz, 1H, CH_{ar}), 7.46-7.43 (m, 3H, CH_{ar}), 7.34 (t, J = 7.8 Hz, 1H, CH_{ar}), 7.26-7.23 (m, 1H, CH_{ar}), 7.17 (t, J = 8.55 Hz, 2H, CH_{ar}), 7.02 (t, J = 8.7 Hz, 2H, CH_{ar}) ppm. ^{13}C NMR (125 MHz, $CDCl_3$): δ = 162.5 (d, J_{C-F} = 247.16 Hz), 162.4 (d, J_{C-F} = 244.76 Hz), 153.9, 149.8, 131.4 (d, J_{C-F} = 8.4 Hz), 130.0, 128.9 (d, J_{C-F} = 8.4 Hz), 128.5, 126.7, 124.8, 123.1, 119.8, 116.2 (d, J_{C-F} = 21.6 Hz), 115.6 (d, J_{C-F} = 21.6 Hz), 111.1 ppm. IR (KBr): ṽ = 2922, 2853, 1611, 1503, 1451, 1505, 1076, 986, 814, 746 cm^{-1}. HRMS (EI^+): $[M]^+$ calcd for $C_{20}H_{12}F_2O$ 306.0856; found 306.0859.

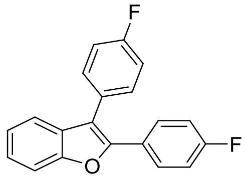

4.33 Compound **3.6**. [6e] Colourless solid (0.102 g, 82%), mp 134-136 ^oC; R_f (2% EtOAc/Hexane) 0.54; ^{1}H NMR (500 MHz, $CDCl_3$): δ = 7.61 (d, J = 8.85 Hz, 2H, CH_{ar}), 7.52 (d, J = 7.95 Hz, 1H, CH_{ar}), 7.47 (d, J = 7.95 Hz, 1H, CH_{ar}), 7.42 (d, J = 8.85 Hz, 2H, CH_{ar}), 7.31-7.20 (m, 2H, CH_{ar}), 7.0 (d, J = 8.55 Hz, 2H, CH_{ar}), 6.85 (d, J = 8.9 Hz, 2H, CH_{ar}), 3.89 (s, 3H, OCH_3), 3.82 (s, 3H, OCH_3) ppm. ^{13}C NMR (125 MHz, $CDCl_3$): δ = 159.6, 159.0, 153.8, 150.5, 130.9, 130.6, 128.4, 125.2, 124.1, 123.5, 122.7, 119.7, 115.6, 114.4, 113.9, 110.9, 55.3, 55.3 ppm. IR (KBr): ṽ = 2966, 2938, 2836, 1607, 1501, 1452, 1248, 1067 cm^{-1}. HRMS (EI): $[M]^+$ calcd for $C_{22}H_{18}O_3$ 330.1256; found 330.1259.

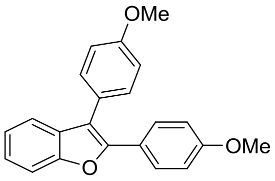

4.34 Compound **3.7**. Colourless solid (0.106 g, 85%), mp 88-90 °C; R_f (2% EtOAc/Hexane) 0.64; ^{1}H NMR (500 MHz, CDCl$_3$): δ = 7.54 (d, *J* = 8.25 Hz, 2H, CH$_{ar}$), 7.45-7.43 (m, 2H, CH$_{ar}$), 7.35 (d, *J* = 7.95 Hz, 2H, CH$_{ar}$), 7.28-7.24 (m, 3H, CH$_{ar}$), 7.13 (d, *J* = 7.9 Hz, 2H, CH$_{ar}$), 2.44 (s, 3H, CH$_3$), 2.35 (s, 3H, CH$_3$) ppm. ^{13}C NMR (125 MHz, CDCl$_3$): δ = 152.2, 152.0, 138.7, 137.6, 131.9, 129.9, 129.5, 129.2, 128.4, 127.5, 126.9, 124.5, 119.5, 116.4, 112.0, 21.4, 21.4 ppm. IR (KBr): ṽ = 1577, 1607, 1500, 1442, 1415, 1403, 1262, 1072, 821, 802, 721 cm^{-1}. HRMS (EI): [M]$^+$ calcd for $C_{22}H_{17}ClO$ 332.0963; found 332.0963.

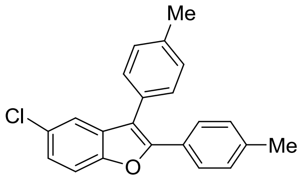

4.35 Compound **4.1**. [6f] Colourless solid (0.098 g, 83%), mp 98-100 °C; R_f (2% EtOAc/Hexane) 0.58; ^{1}H NMR (400 MHz, CDCl$_3$): δ = 7.63 (d, *J* = 9.16 Hz, 2H, CH$_{ar}$), 7.55 (d, *J* = 8.24 Hz, 1H, CH$_{ar}$), 7.50 (dd, *J* = 7.8 Hz, 1.36 Hz, 1H, CH$_{ar}$), 7.42 (d, *J* = 7.8 Hz, 2H, CH$_{ar}$), 7.34-7.21 (m, 4H, CH$_{ar}$), 6.87 (d, *J* = 9.16 Hz, 2H, CH$_{ar}$), 3.83 (s, 3H, OCH$_3$), 2.46 (s, 3H, CH$_3$) ppm. ^{13}C NMR (100 MHz, CDCl$_3$): δ = 159.6, 153.8, 150.5, 137.1, 130.5, 129.9, 129.6, 129.6, 128.4, 124.1, 123.4, 122.7, 119.8, 115.9, 113.9, 110.9, 55.2, 21.3 ppm. IR (KBr): ṽ = 2930, 2835, 1610, 1503, 1452, 1255, 1178, 1031, 832, 745 cm^{-1}. HRMS (ESI): [M+H]$^+$ calcd for $C_{22}H_{19}O_2$ 315.1385; found 315.1388.

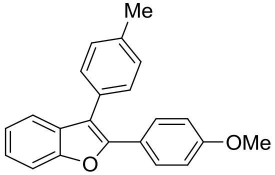

4.36 Compound **4.2**. Colourless solid (0.105 g, 81%), mp 108-110 °C; R_f (2% EtOAc/Hexane) 0.48; ^{1}H NMR (400 MHz, CDCl$_3$): δ = 7.61 (d, *J* = 9.16 Hz, 2H, CH$_{ar}$), 7.52 (d, *J* = 7.8 Hz, 1H, CH$_{ar}$), 7.47 (d, *J* = 7.8 Hz, 1H, CH$_{ar}$), 7.40 (d, *J* = 8.72 Hz, 2H, CH$_{ar}$), 7.31-7.27 (m, 1H, CH$_{ar}$), 7.24-7.20 (m, 1H, CH$_{ar}$), 6.99 (d, *J* = 8.68 Hz, 2H, CH$_{ar}$), 6.85 (d, *J* = 8.72 Hz, 2H, CH$_{ar}$), 4.13-4.08 (q, *J* = 7.03 Hz, 2H, CH$_{ar}$), 3.82 (s, 3H, CH$_{ar}$), 1.48 (t, *J* = 6.88 Hz, 3H, CH$_{ar}$) ppm. ^{13}C NMR (100 MHz, CDCl$_3$): δ = 159.6, 158.4, 153.8, 150.4, 130.9, 130.6, 128.4, 125.0, 124.1, 123.5, 122.7, 119.7, 115.7, 114.9, 113.9, 110.9, 63.4, 55.2, 14.9 ppm. IR (KBr): ṽ = 2970, 2934, 1609, 1594, 1519, 1503, 1249, 1174, 1044, 831 cm^{-1}. HRMS (EI): [M]$^+$ calcd for $C_{23}H_{20}O_3$ 344.1412; found 344.1412.

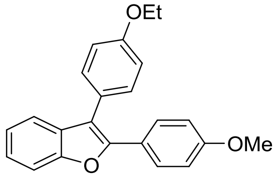

4.37 Compound **4.3**. Colourless solid (0.112 g, 73%), mp 94-96 °C; R_f (2% EtOAc/Hexane) 0.46; ^{1}H NMR (500 MHz, CDCl$_3$): δ = 7.61 (d, *J* = 9.15 Hz, 2H, CH$_{ar}$), 7.53-7.41 (m, 8H, CH$_{ar}$), 7.36 (t, *J* = 7.0 Hz, 1H, CH$_{ar}$), 7.31-7.20 (m, 2H, CH$_{ar}$), 7.08 (d, *J* = 8.55 Hz, 2H, CH$_{ar}$), 6.86 (d, *J* = 8.55 Hz, 2H, CH$_{ar}$), 5.14 (s, 2H, OCH$_2$), 3.82 (s, 3H, OCH$_3$) ppm. ^{13}C NMR (125 MHz, CDCl$_3$): δ = 159.6, 158.3, 153.8, 150.5, 136.9, 130.9, 130.6, 128.6, 128.4, 128.1, 127.6, 125.5, 124.2, 123.5, 122.7, 119.7, 115.6, 115.3, 113.9, 110.9, 70.1, 55.3 ppm. IR (KBr): ṽ = 3034, 2932, 1607, 1516, 1453, 1249, 1238, 1173, 833, 698 cm^{-1}. HRMS (ESI): [M]$^+$ calcd for $C_{28}H_{22}O_3$ 406.1569; found 406.1569.

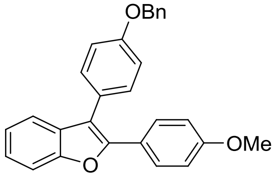

4.38 Compound **5.1**. [6g] Pale yellow solid (0.086 g, 79%), mp 132-134 °C; R_f (2% EtOAc/Hexane) 0.42; ^{1}H NMR (500 MHz, $CDCl_3$): δ = 7.62 (d, J = 8.55 Hz, 2H, CH_{ar}), 7.58-7.52 (m, 4H, CH_{ar}), 7.49-7.47 (m, 1H, CH_{ar}), 7.45 (d, J = 9.15 Hz, 2H, CH_{ar}), 7.02 (d, J = 8.55 Hz, 2H, CH_{ar}), 6.96 (d, J = 8.55 Hz, 2H, CH_{ar}), 6.87 (d, J = 8.55 Hz, 2H, CH_{ar}), 3.89 (s, 3H, OCH_3), 3.85 (s, 3H, OCH_3), 3.83 (s, 3H, OCH_3) ppm. ^{13}C NMR (125 MHz, $CDCl_3$): δ = 159.6, 159.0, 158.8, 153.1, 151.1, 136.2, 134.4, 131.1, 130.9, 128.4, 128.3, 125.1, 123.5, 123.4, 117.7, 115.8, 114.5, 114.1, 113.9, 110.9, 55.3, 55.3 ppm. IR (KBr): ṽ = 2946, 2834, 1611, 1518, 1464, 1249, 1176, 1039, 836, 802 cm^{-1}. HRMS (ESI): $[M+H]^+$ calcd for $C_{29}H_{25}O_4$ 437.1753; found 437.1752.

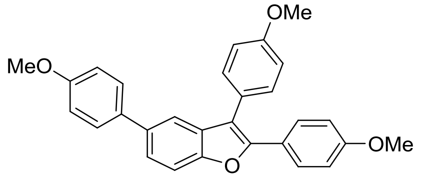

4.39 Compound **5.2**. Colourless solid (0.076 g, 78%), mp 126-128 °C; R_f (2% EtOAc/Hexane) 0.52; ^{1}H NMR (500 MHz, $CDCl_3$): δ = 7.63 (d, J = 1.2 Hz, 1H, CH_{ar}), 7.58-7.51 (m, 4H,CH_{ar}), 7.49 (d, J = 7.95 Hz, 2H, CH_{ar}), 7.41 (d, J = 8.25 Hz, 2H, CH_{ar}), 7.28 (d, J = 7.95 Hz, 2H, CH_{ar}), 7.23 (d, J = 7.9 Hz, 2H, CH_{ar}), 7.13 (d, J = 7.95 Hz, 2H, CH_{ar}), 2.44 (s, 3H, CH_3), 2.39 (s, 3H, CH_3), 2.36 (s, 3H, CH_3) ppm. ^{13}C NMR (125 MHz, $CDCl_3$): δ = 153.4, 151.3, 138.9, 138.3, 137.3, 136.5, 130.9, 129.8, 129.7, 129.7, 129.4, 129.1, 128.0, 127.3, 126.9, 123.9, 118.2, 117.0, 111.0, 21.4, 21.0 ppm. IR (KBr, cm^{-1}): ṽ = 2918, 1606, 1485, 1331, 1124, 1067, 920, 870, 782, 692. HRMS (EI^+): $[M]^+$ calcd for $C_{29}H_{24}O$ 388.1827; found 388.1823.

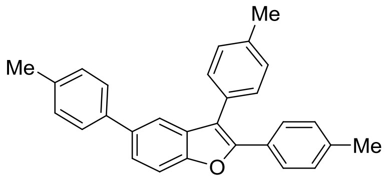

4.40 Compound **5.3**. Colourless solid (0.060 g, 62%), mp 112-114 °C; R_f (2% EtOAc/Hexane) 0.52; ^{1}H NMR (500 MHz, $CDCl_3$): δ = 7.64 (s, 1H, CH_{ar}), 7.61-7.53 (m, 3H, CH_{ar}), 7.42-7.30 (m, 7H, CH_{ar}), 7.25-7.11 (m, 4H, CH_{ar}), 2.42 (s, 3H, CH_3), 2.41 (s, 3H, CH_3), 2.34 (s, 3H, CH_3) ppm. ^{13}C NMR (125 MHz, $CDCl_3$): δ = 153.5, 151.2, 141.7, 138.6, 138.3, 138.1, 136.8, 132.7, 130.8, 130.6, 130.4, 129.1, 128.9, 128.6, 128.4, 128.3, 128.3, 127.6, 127.5, 126.9, 124.6, 124.3, 124.2, 118.5, 117.7, 111.1, 21.5, 21.5 ppm. IR (KBr): ṽ = 3030, 2920, 1605, 1463, 1264, 1065, 781, 701 cm^{-1}. HRMS (EI): $[M]^+$ calcd for $C_{29}H_{24}O$ 388.1827; found 388.1829.

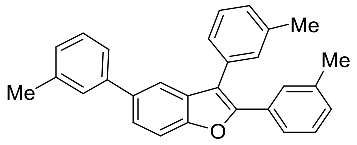

4.41 Compound **5.4**. Colourless solid (0.089 g, 75%), mp 134-136 °C; R_f (2% EtOAc/Hexane) 0.44; ^{1}H NMR (500 MHz, $CDCl_3$): δ = 7.60 (d, J = 8.55 Hz, 2H, CH_{ar}), 7.57 (d, J = 1.85 Hz, 1H, CH_{ar}), 7.54 (d, J = 7.95 Hz, 1H, CH_{ar}), 7.50 (d, J = 8.55 Hz, 2H, CH_{ar}), 7.48-7.46 (m, 1H, CH_{ar}), 7.42 (d, J = 8.55 Hz, 2H, CH_{ar}), 6.99 (d, J = 8.55 Hz, 2H, CH_{ar}), 6.94 (d, J = 8.55 Hz, 2H, CH_{ar}), 6.84 (d, J = 9.15 Hz, 2H, CH_{ar}), 4.13-4.02 (m, 6H, $-OCH_2$), 1.48-1.40 (m, 9H, CH_3) ppm. ^{13}C NMR (125 MHz, $CDCl_3$): δ = 159.0, 158.4, 158.2, 153.1, 151.1, 136.2, 134.2, 131.2, 130.9, 128.4, 125.0, 123.5, 123.3, 117.7, 115.8, 115.0, 114.7, 114.4, 111.0, 63.5, 63.5, 14.9, 14.9, 14.8 ppm. IR

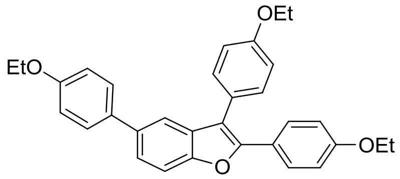

(KBr): ṽ = 2976, 2925, 1608, 1517, 1462, 1247, 1178, 1047, 840, 804 cm^{-1}. HRMS (ESI): $[M+H]^+$ calcd for $C_{32}H_{31}O_4$ 479.2222; found 479.2221.

5. Crystallographic data for compound 3.1 (CCDC-1425338):

	Compound **3.1**
Formula	$C_{22}H_{18}O$
M/g	298.36
Crystal system	Monoclinic
Space group	*C*2/c
a/Å	16.6170(10)
b/Å	7.382(5)
c/Å	26.235(5)
β (°)	99.282(5)
V/Å^3	3176(2)
Z	8
ρ_c/g cm^{-3}	1.248
μ/mm^{-1}	0.075
F(000)	1264.0
Cryst size (mm^3)	0.02 × 0.02 × 0.02
2*θ* range (deg)	8.442 to 50.042
Limiting indices	-19<=h<=19, -8<=k<=8, 25<=l<=31
Reflns collected	10125
Ind reflns	2795 [R(int) = 0.0600]
Completeness to *θ* (%)	99.4
Refinement method	Full-matrix least-squares on F^2
Data/restraints/params	2795/0/210
Goodness-of-fit on F^2	1.068
Final R indices [$I > 2\theta(I)$]	R_1 = 0.0516, wR_2 = 0.1244
R indices (all data)	R_1 = 0.0655, wR_2 = 0.1341
Largest diff. peak and hole(e·Å^{-3})	0.34 and -0.44

6. References:

[1] Bach, T.; Bartels, M. *Synthesis* **2003**, 925-939.

[2] (a) Hung, N. T.; Hussain, M.; Malik, I.; Villinger, A.; Langer, P. *Tetrahedron Lett.* **2010**, *51*, 2420-2422; (b) Hussain, M.; Hung, N. T.; Langer, P. *Tetrahedron Lett.* **2009**, *50*, 3929-3932.

[3] (a) Shimada, S.; Rao, M. L. N. *Topics in Current Chemistry*. **2012**, *311*, 199-228; (b) Suzuki, H.; Matano, Y. Organobismuth chemistry. Eds.; Elsevier, Amsterdam, 2001.

[4] CCDC-1425338 contains all crystallographic details of this publication and is available free of charge at www.ccdc.cam.ac.uk/conts/-retrieving.html or can be ordered from the following address: Cambridge Crystallographic Data Centre, 12 Union Road, GB-Cambridge CB21EZ; Fax: (+44)1223-336-033; or deposit@ ccdc.cam.ac.uk

[5] SAINT+ 6.02ed.; Bruker AXS, Madison, WI, 1999; (b) G. M. Sheldrick, SHELXL-97: Program for Crystal Structure Refinement, University of Gottingen, Gottingen, Germany, 1997

[6] (a) Jacubert, M.; Tikad, A.; Provot, O.; Hamze, A.; Brion, J.-D.; Alami, M. *Eur. J. Org. Chem.* **2010**, 4492-4500; (b) Liang, Y.; Tang, S.; Zhang, X.-D.; Mao, L.-Q.; Xie, Y.-X.; Li, J.-H. *Org. Lett.* **2006**, *8*, 3017-3020; (c) Zeng, W.; Wu, W.; Jiang, H.; Huang, L.; Sun, Y.; Chen, Z.; Li, X. *Chem. Commun.* **2013**, *49*, 6611-6613; (d) Markina, N. A.; Chen, Y.; Larock, R. C. *Tetrahedron* **2013**, *69*, 2701-2713; (e) Liu, G.; Shen, Y.; Zhou, Z.; Lu, X. *Angew. Chem., Int. Ed.* **2013**, *52*, 6033-6037; (f) Guo, L.; Zhang, F.; Hu, W.; Li, L.; Jia, Y. *Chem. Commun.* **2014**, *50*, 3299-3302; (g) Cho, C.-H.; Neuenswander, B.; Lushington, G. H.; Larock, R. C. *J. Comb. Chem.* **2008**, *10*, 941-947.
