# Supplementary material for: Rapid regio- and multi-coupling reactivity of 2,3-dibromobenzofurans with atom-economic triarylbismuths under palladium catalysis
Source: Beilstein J Org Chem. 2016 Sep 22;12:2065–76. doi: 10.3762/bjoc.12.195 (PMC5082443; doi:10.3762/bjoc.12.195)
Supplement: File 2 — 1H, 13C NMR spectra of all compounds. [file Beilstein_J_Org_Chem-12-2065-s002.pdf]

Supporting Information

for

Rapid regio- and multi-coupling reactivity of 2,3-dibromobenzofurans with atom-economic triarylbismuths under palladium catalysis

Maddali L. N. Rao,* Jalindar B. Talode and Venneti N. Murty

Address: Department of Chemistry, Indian Institute of Technology Kanpur, Kanpur-208016, U.P., India, Tel/ Fax: +91-512-2597532

Email: Maddali L. N. Rao - maddali@iitk.ac.in

*Corresponding author

^{1}H, ^{13}C NMR spectra of all compounds

Contents

1. Copies of 1H NMR, 13C NMR spectra of 1.1 to 1.6…………………………………………………………………… S02
2. Copies of 1H NMR, 13C NMR spectra of 2.1 to 2.27 (Table 2)……………………………………………………….S14
3. Copies of 1H NMR, 13C NMR spectra of 3.1 to 3.7 (Table 4)…………………………………..……………………..…S68
4. Copies of 1H NMR, 13C NMR spectra of 4.1 to 4.3 (Tables 5 and 6)……………………….……………………………...S82
5. Copies of 1H NMR, 13C NMR spectra of 5.1 to 5.4 (Table 7)…………………………………………………………S88

1. ^{1}H, ^{13}C Spectra of Compounds (**1.1-1.6**)

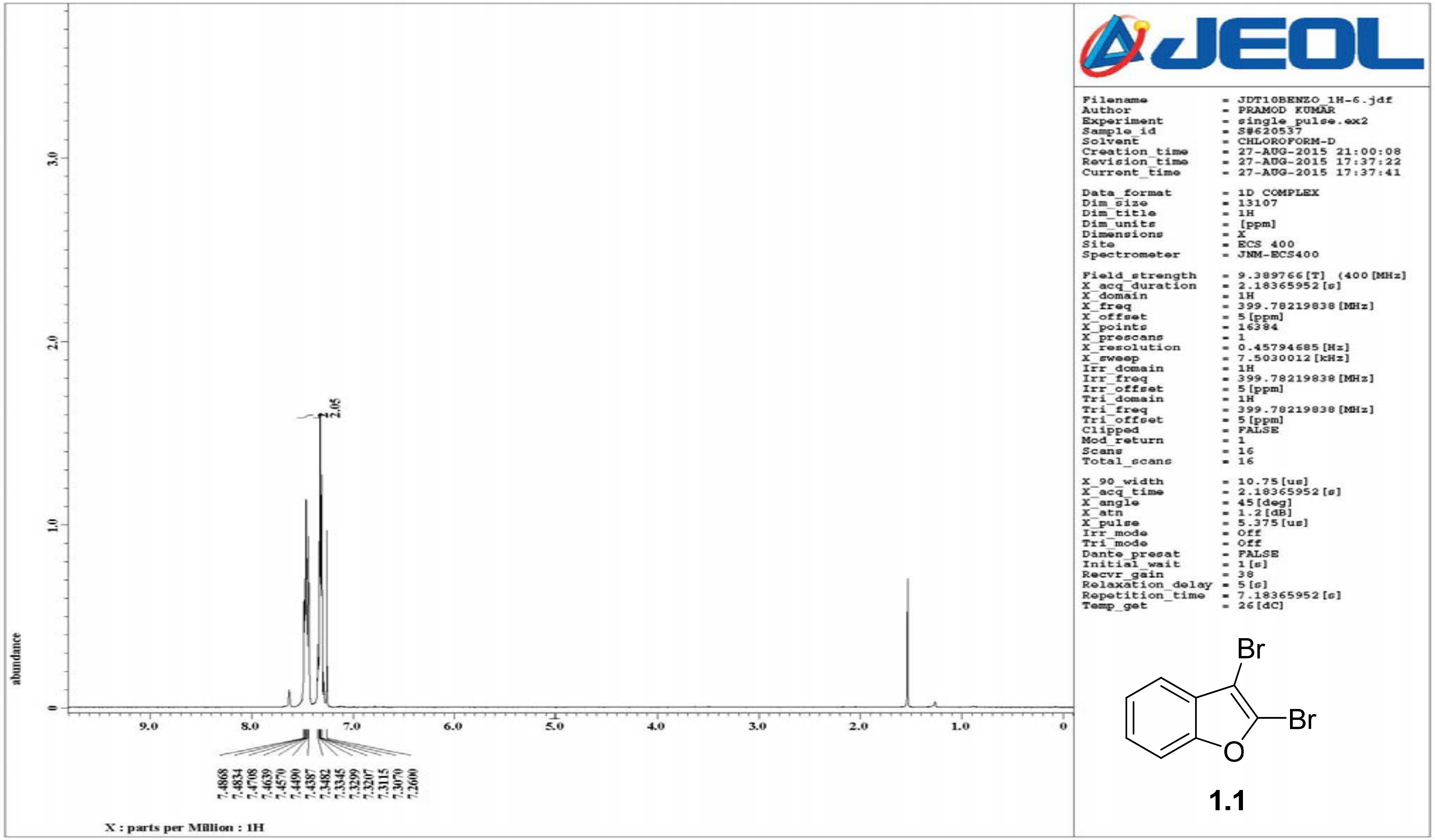

400 MHz ^{1}H NMR spectrum of compound **1.1**

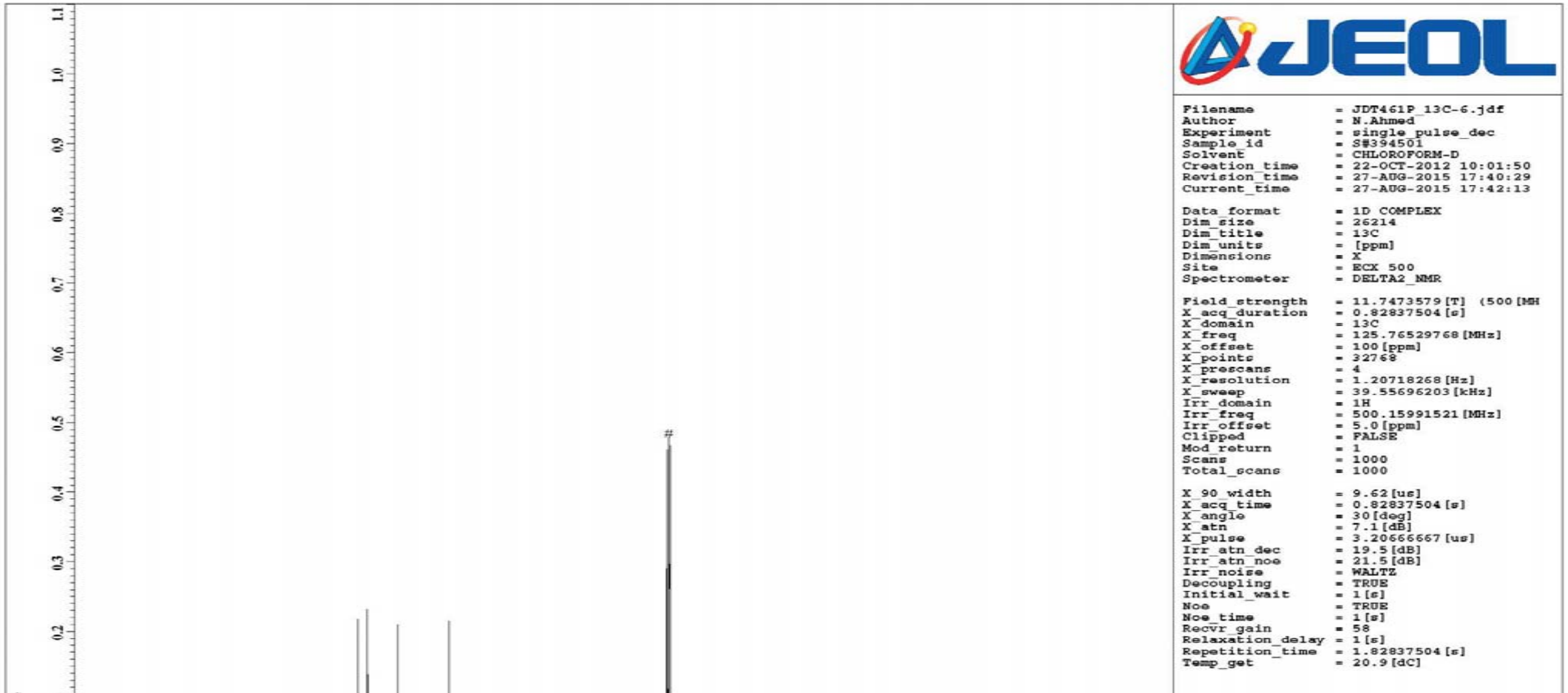

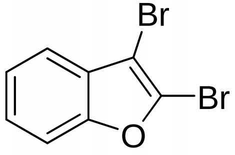

1.1

125 MHz ^{13}C NMR spectrum of compound **1.1**

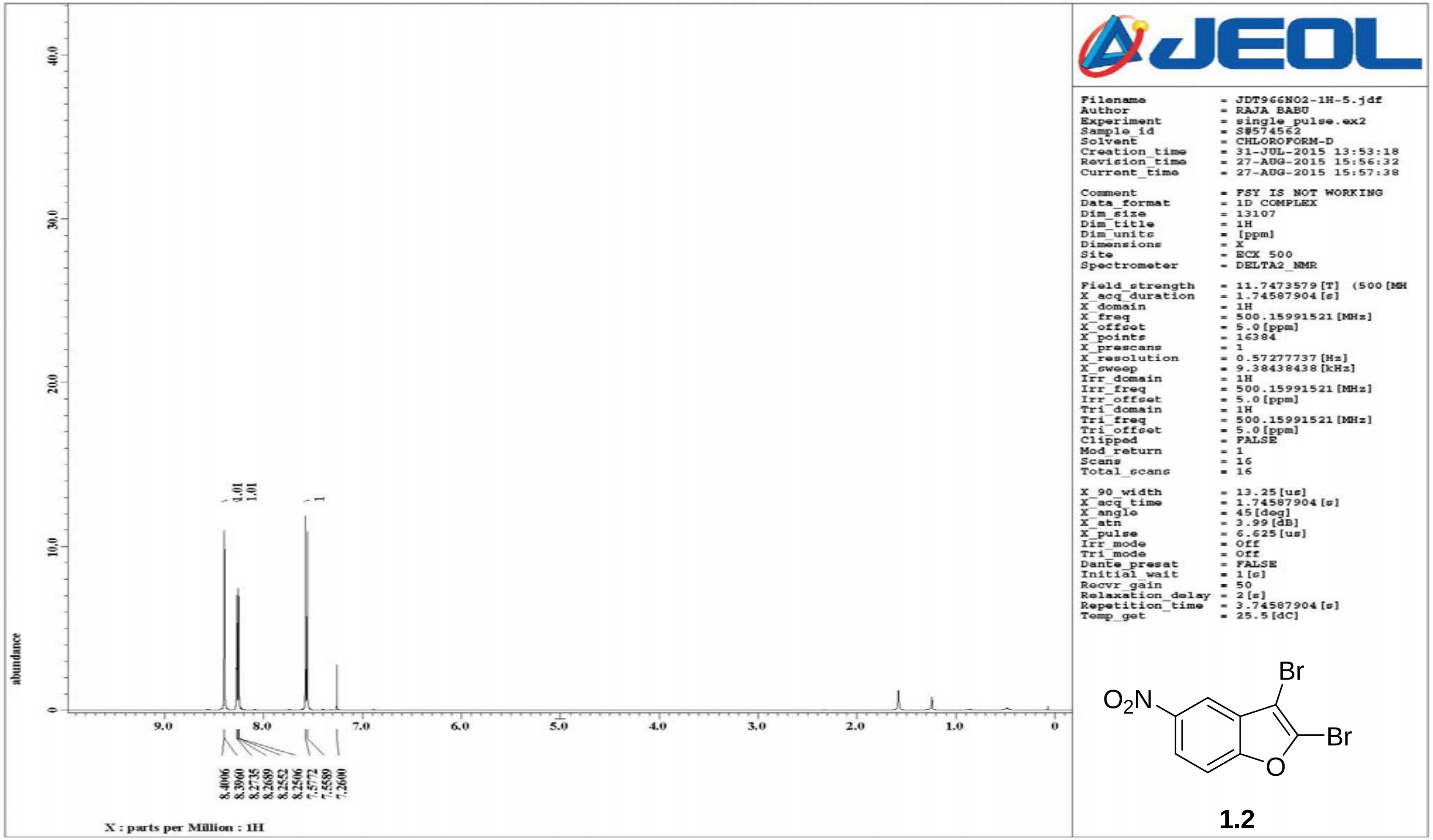

500 MHz ^{1}H NMR spectrum of compound **1.2**

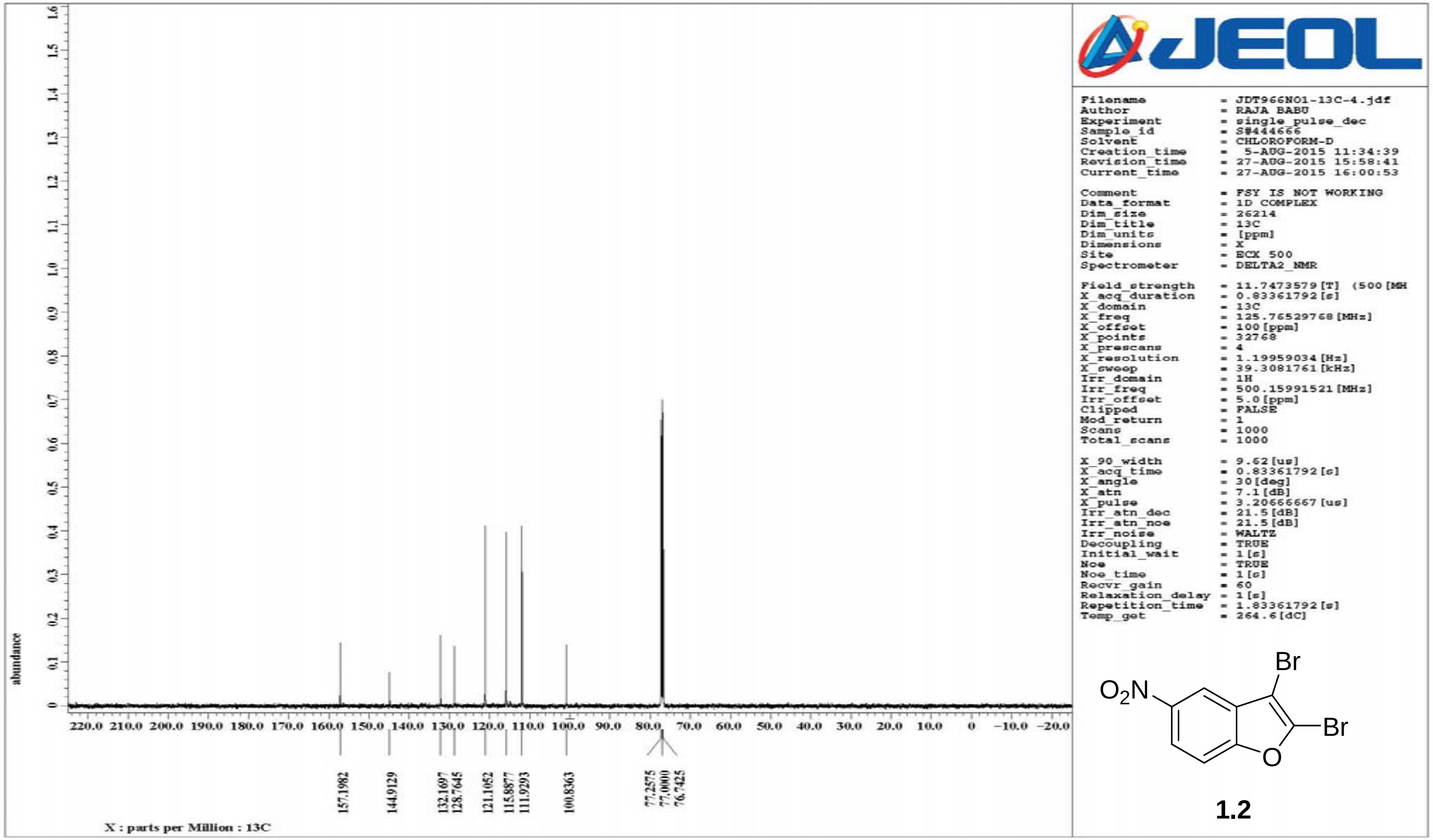

125 MHz ^{13}C NMR spectrum of compound **1.2**

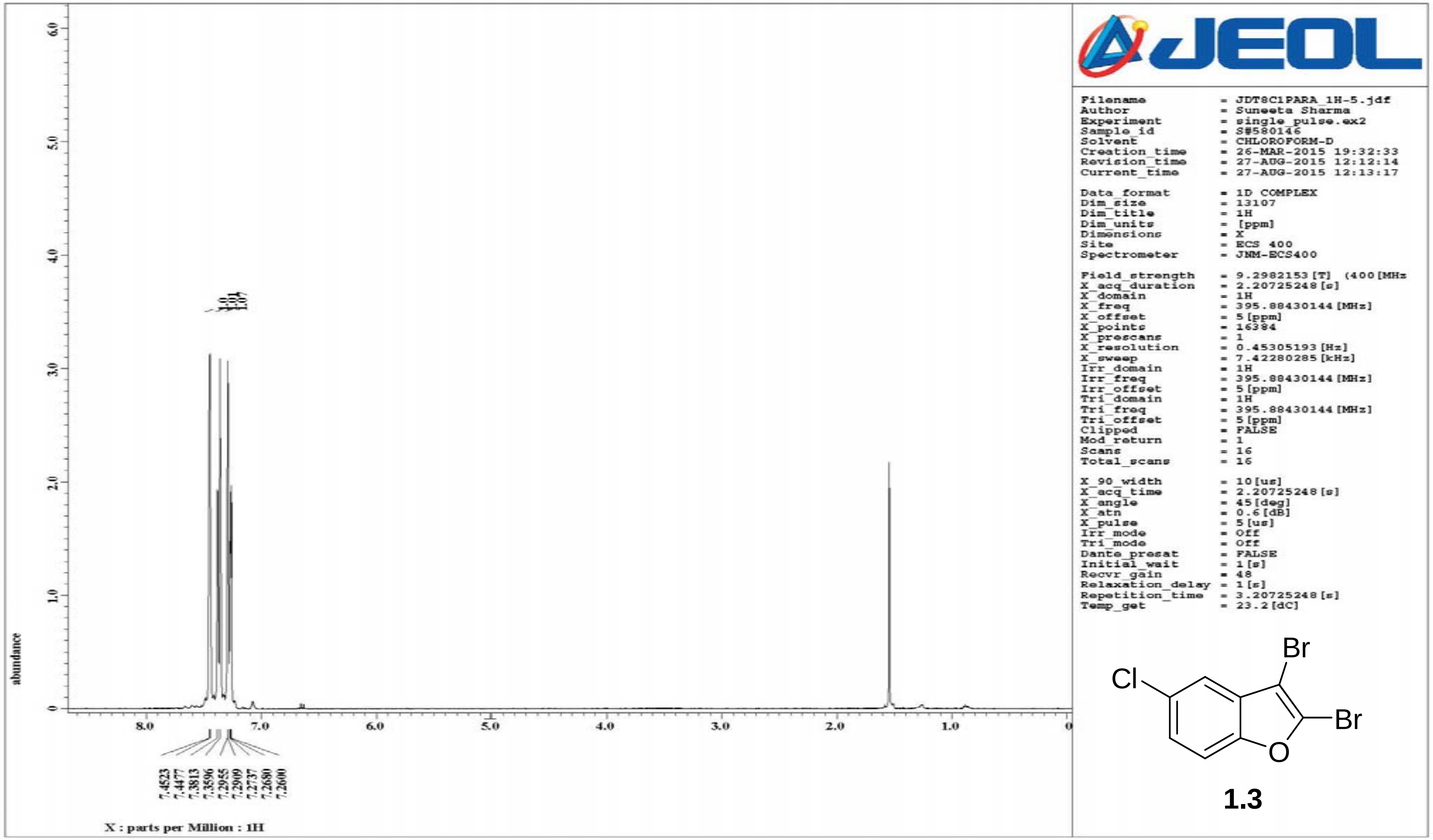

1.3

400 MHz ^{1}H NMR spectrum of compound **1.3**

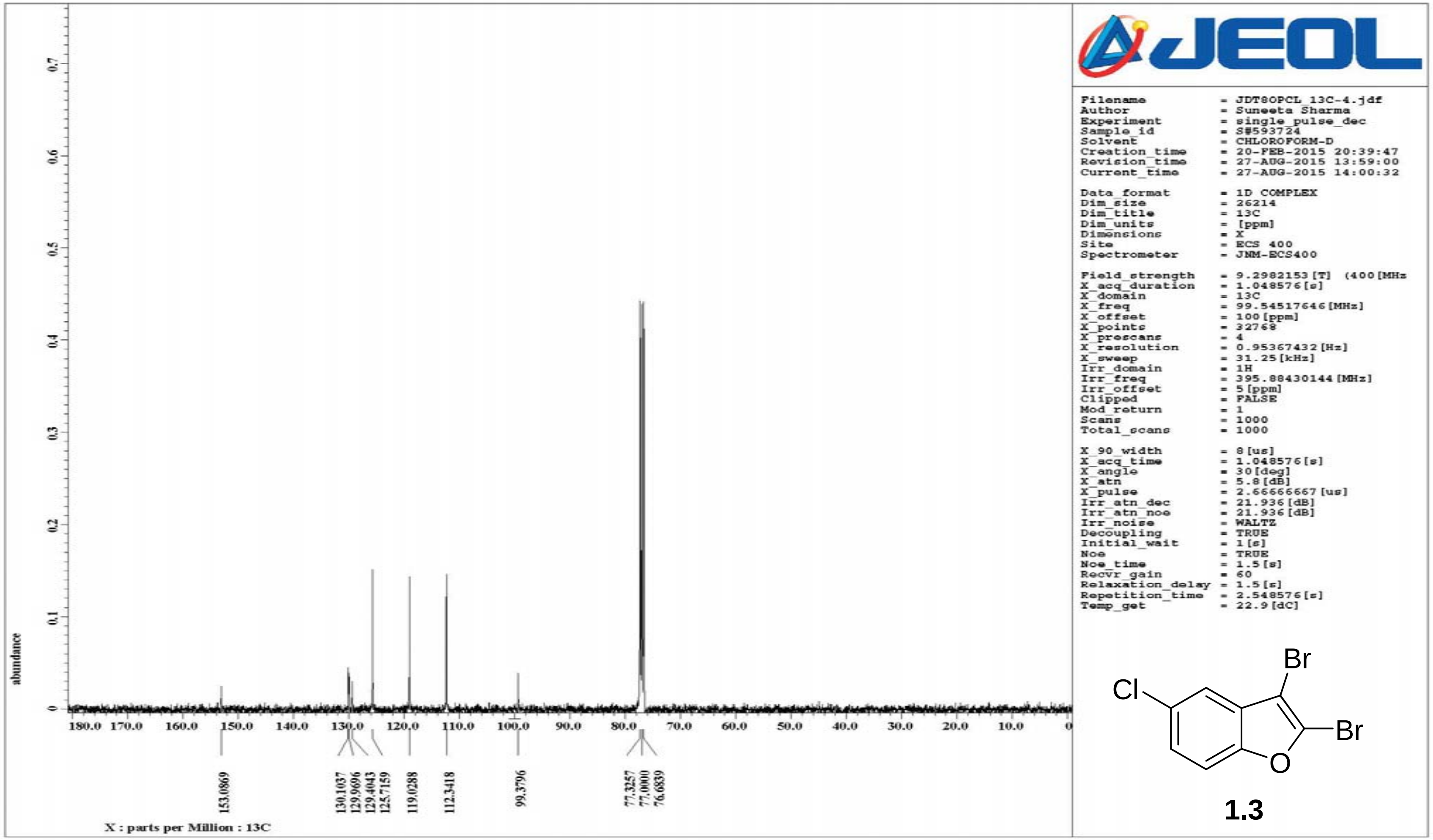

100 MHz ^{13}C NMR spectrum of compound **1.3**

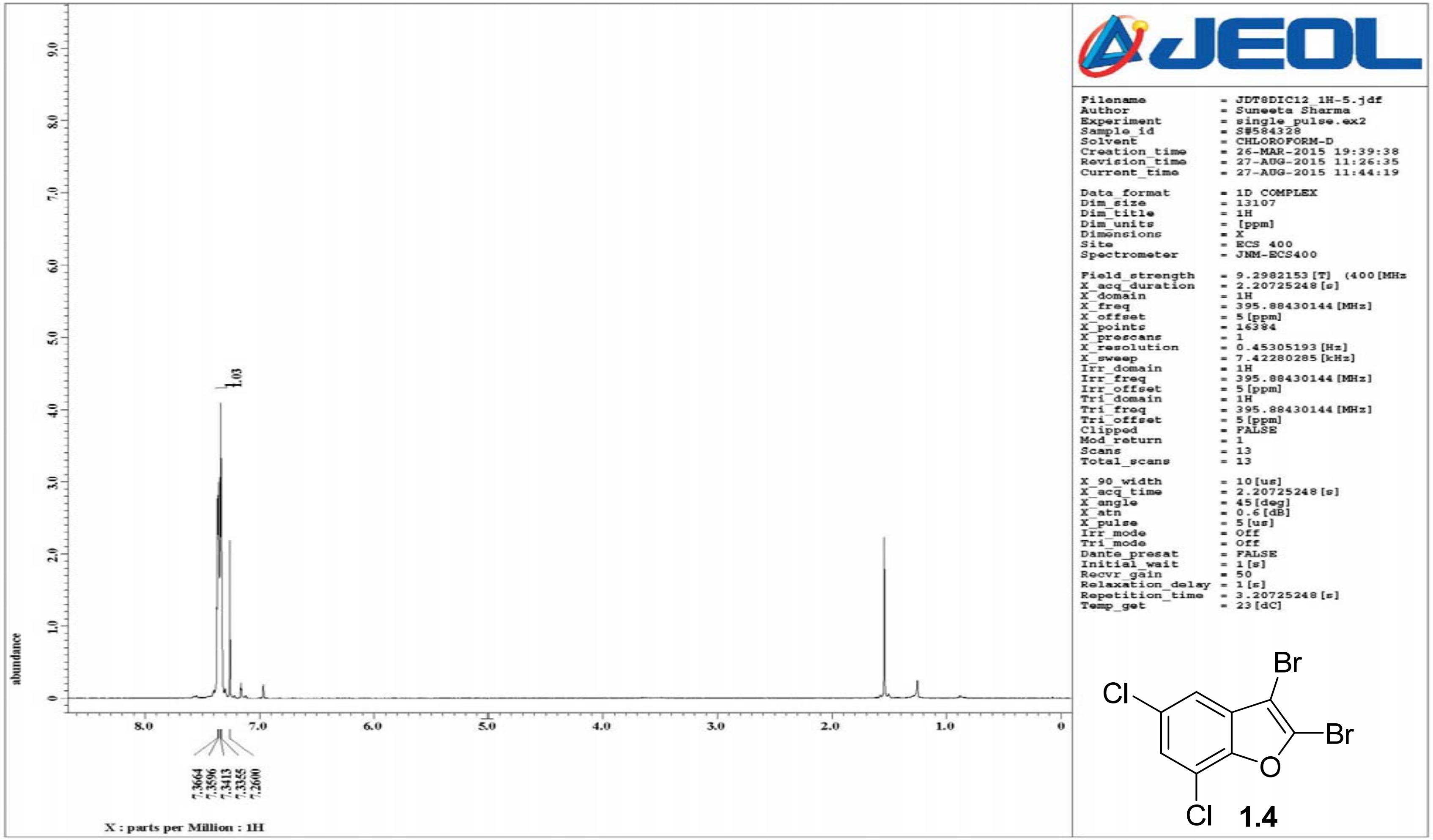

400 MHz ^{1}H NMR spectrum of compound **1.4**

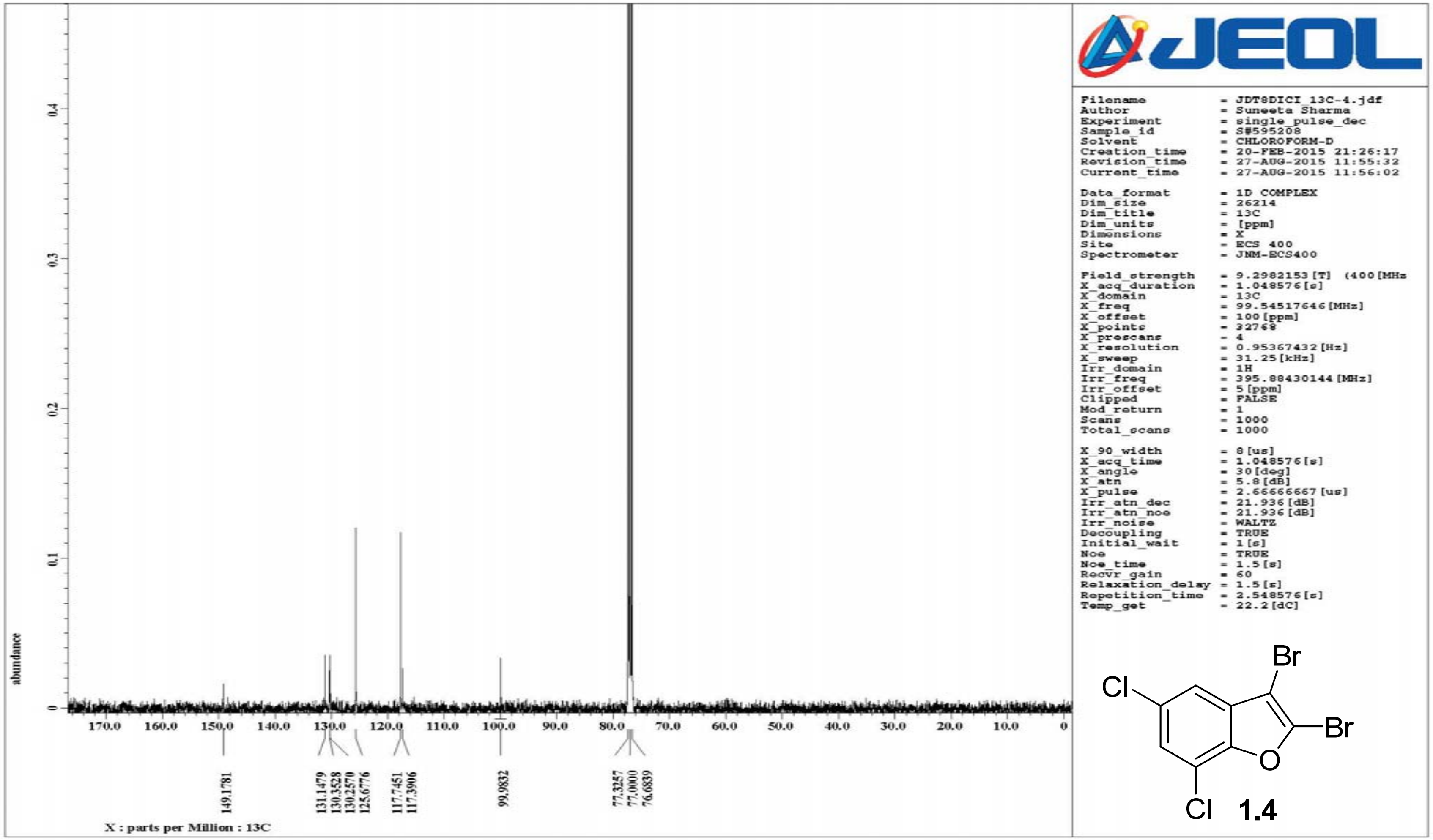

100 MHz ^{13}C NMR spectrum of compound **1.4**

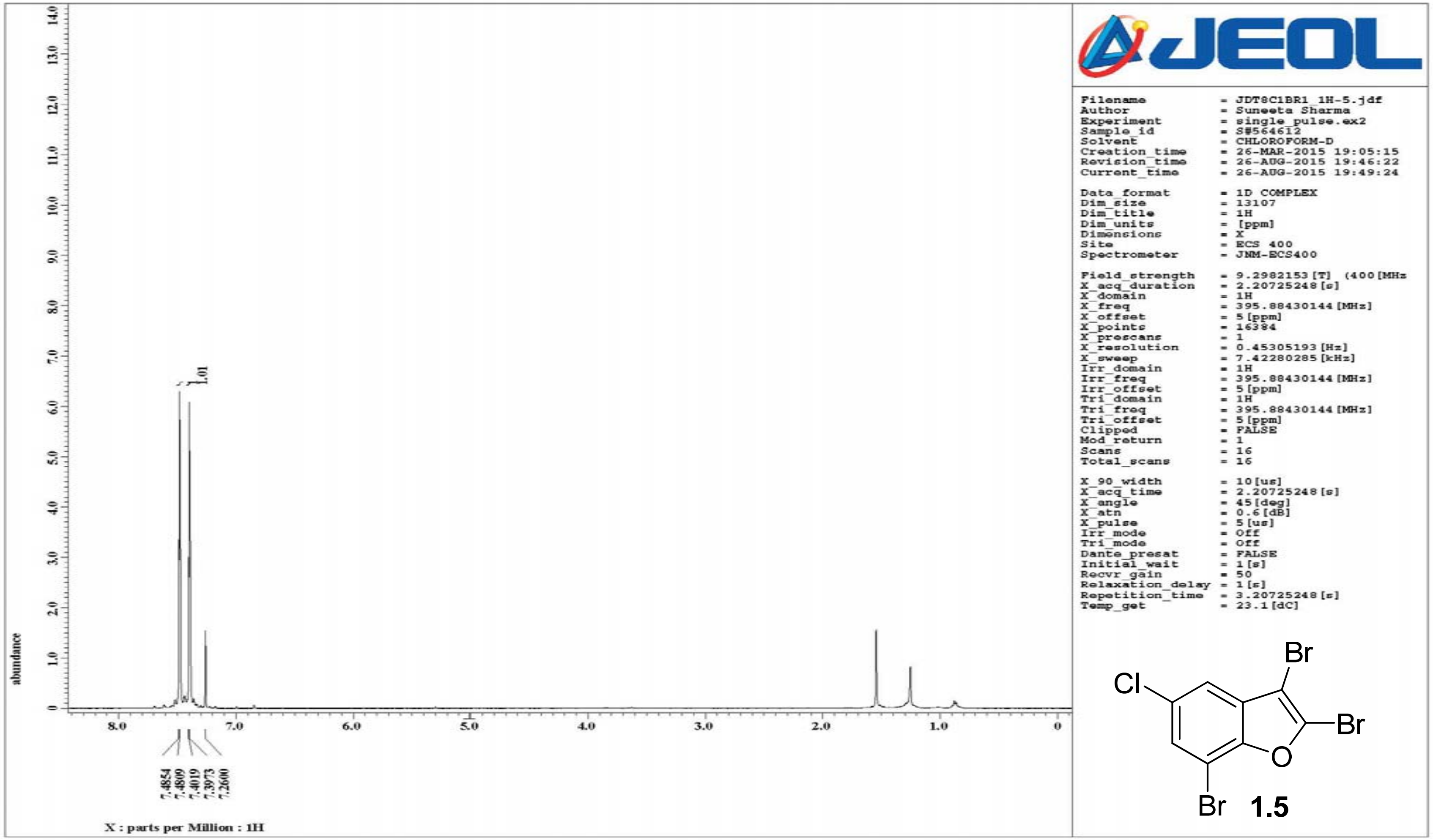

400 MHz ^{1}H NMR spectrum of compound **1.5**

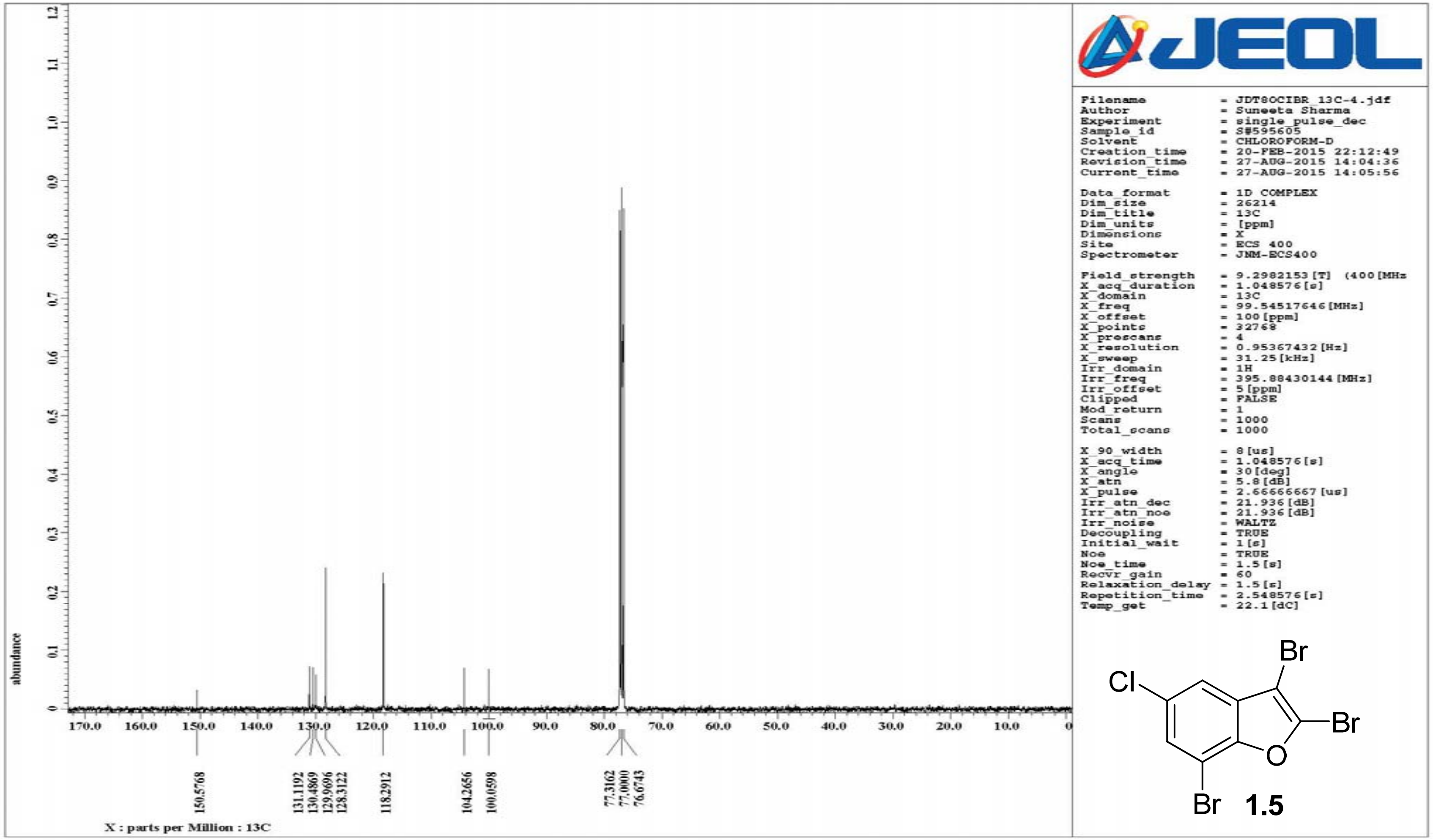

100 MHz ^{13}C NMR spectrum of compound **1.5**

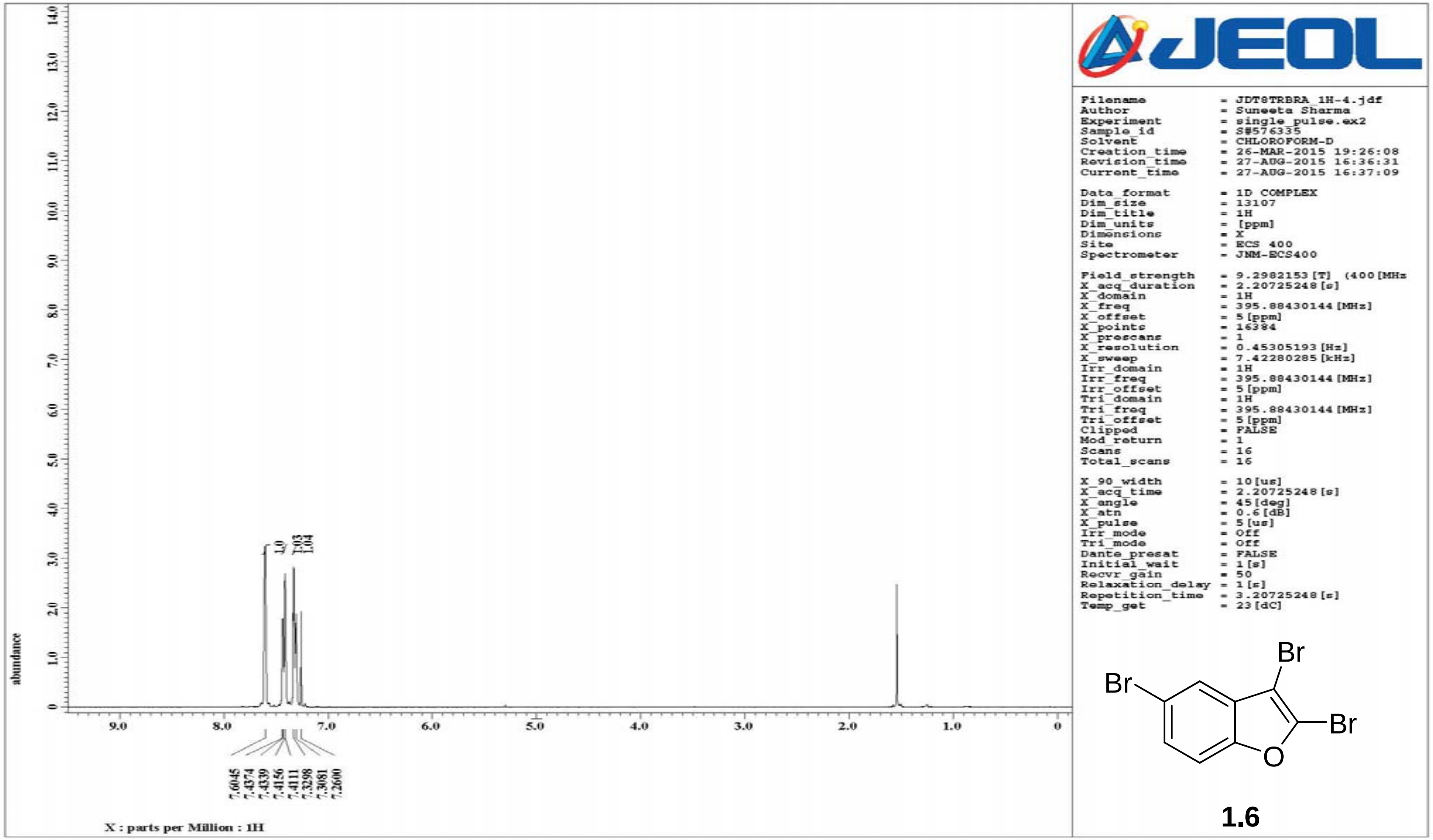

400 MHz ^{1}H NMR spectrum of compound **1.6**

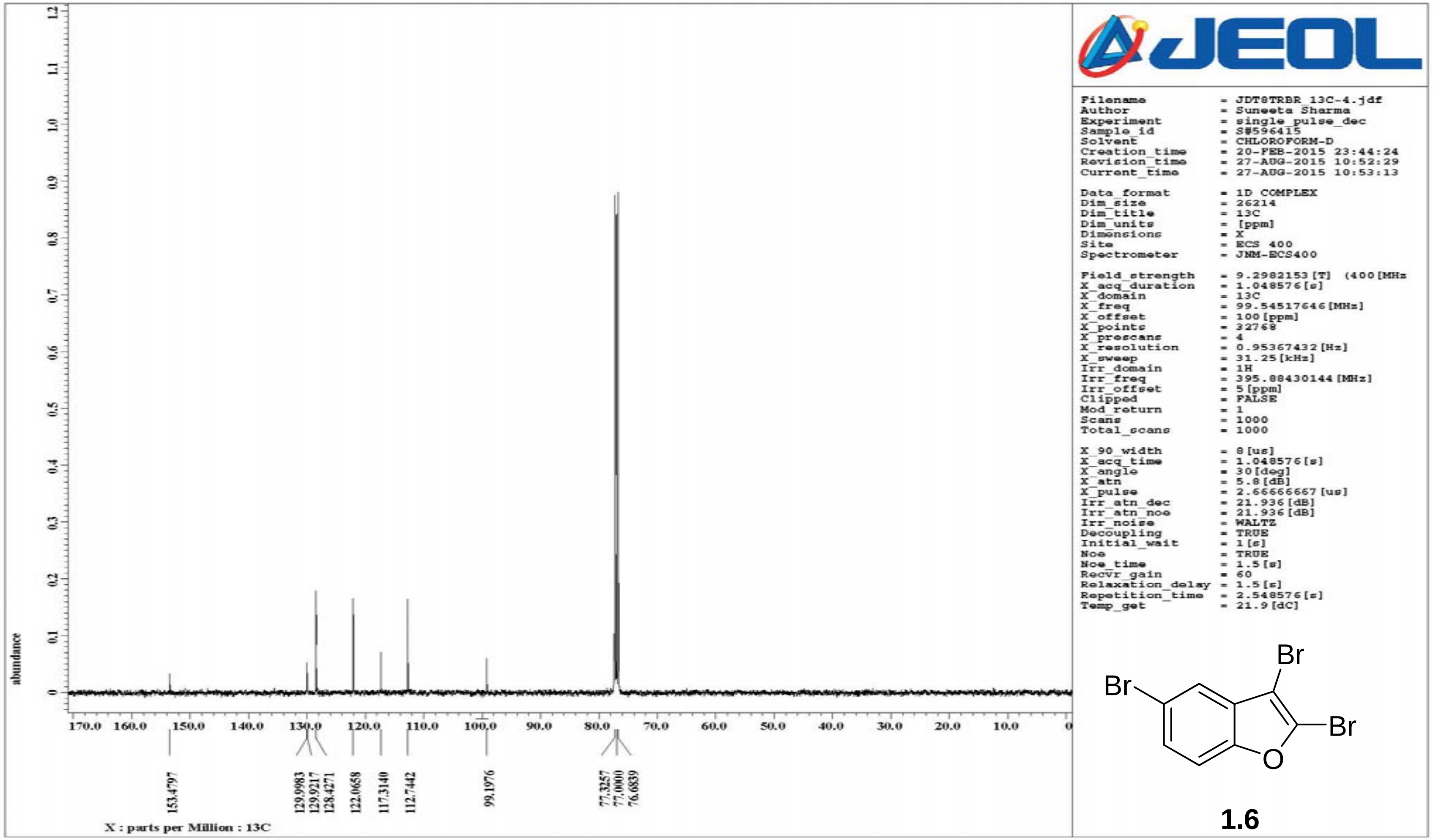

100 MHz ^{13}C NMR spectrum of compound **1.6**

2. ^{1}H, ^{13}C Spectra of Compounds (**2.1-2.27**):

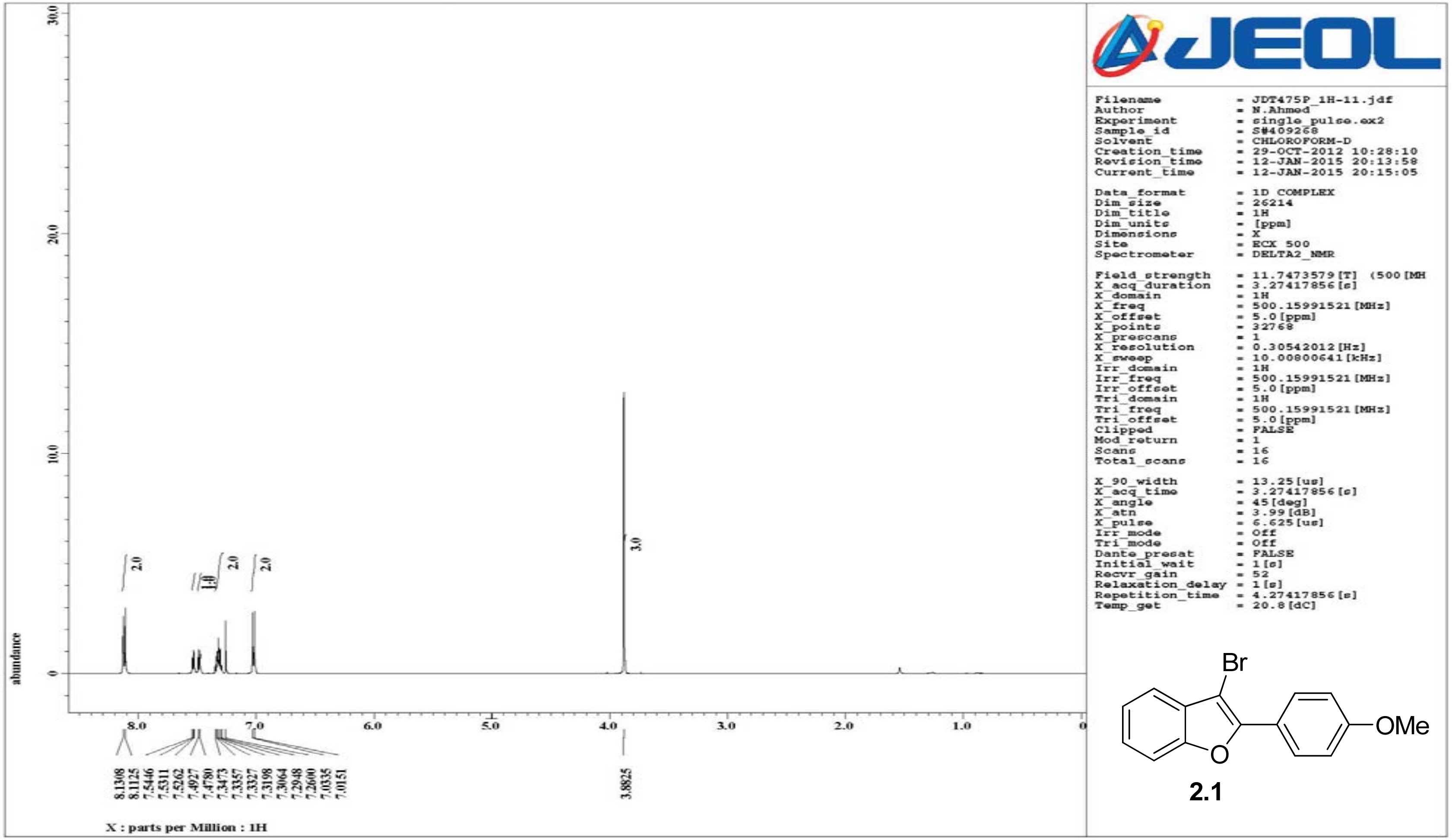

500 MHz ^{1}H NMR spectrum of compound **2.1**

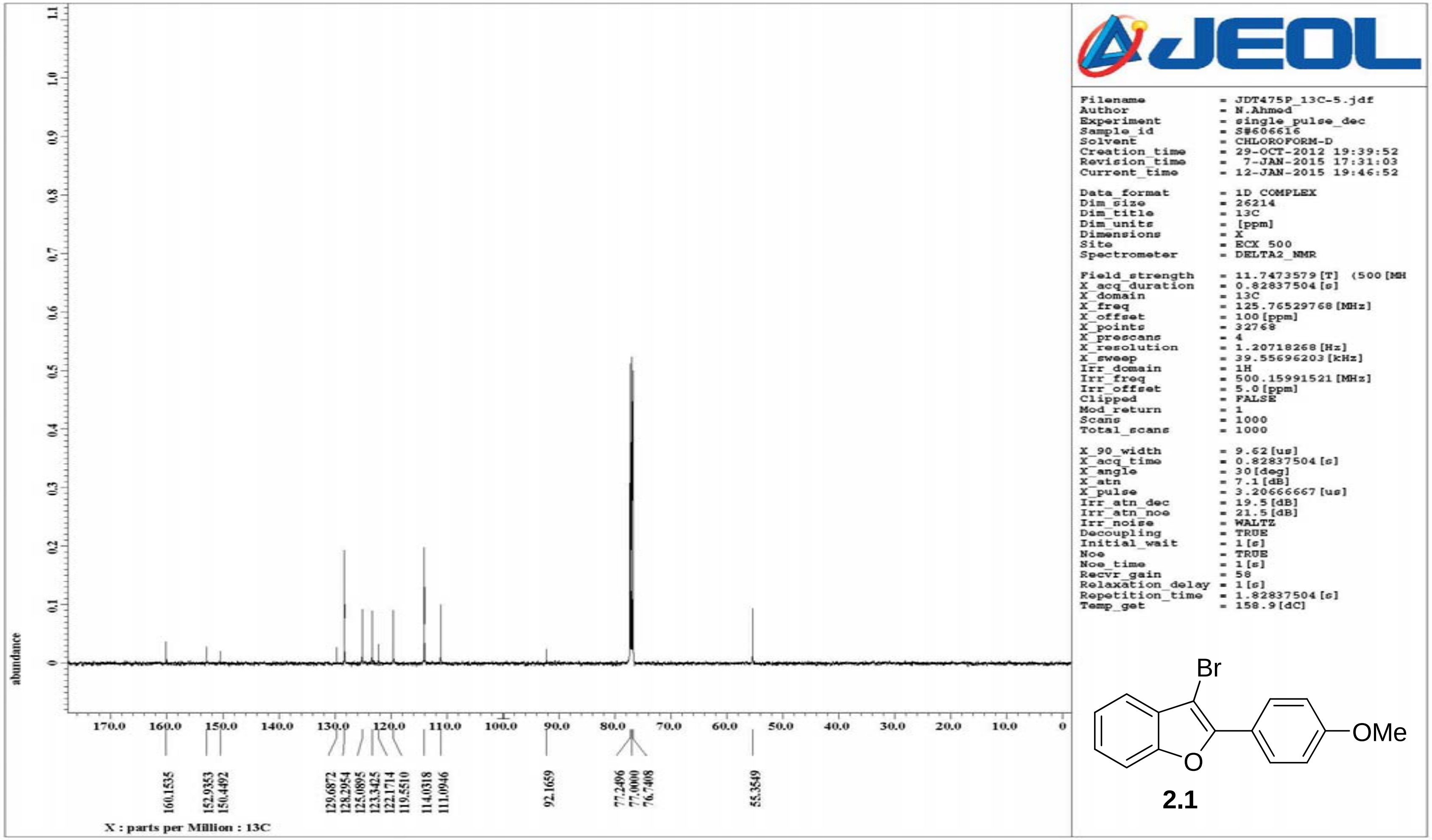

125 MHz ^{13}C NMR spectrum of compound **2.1**

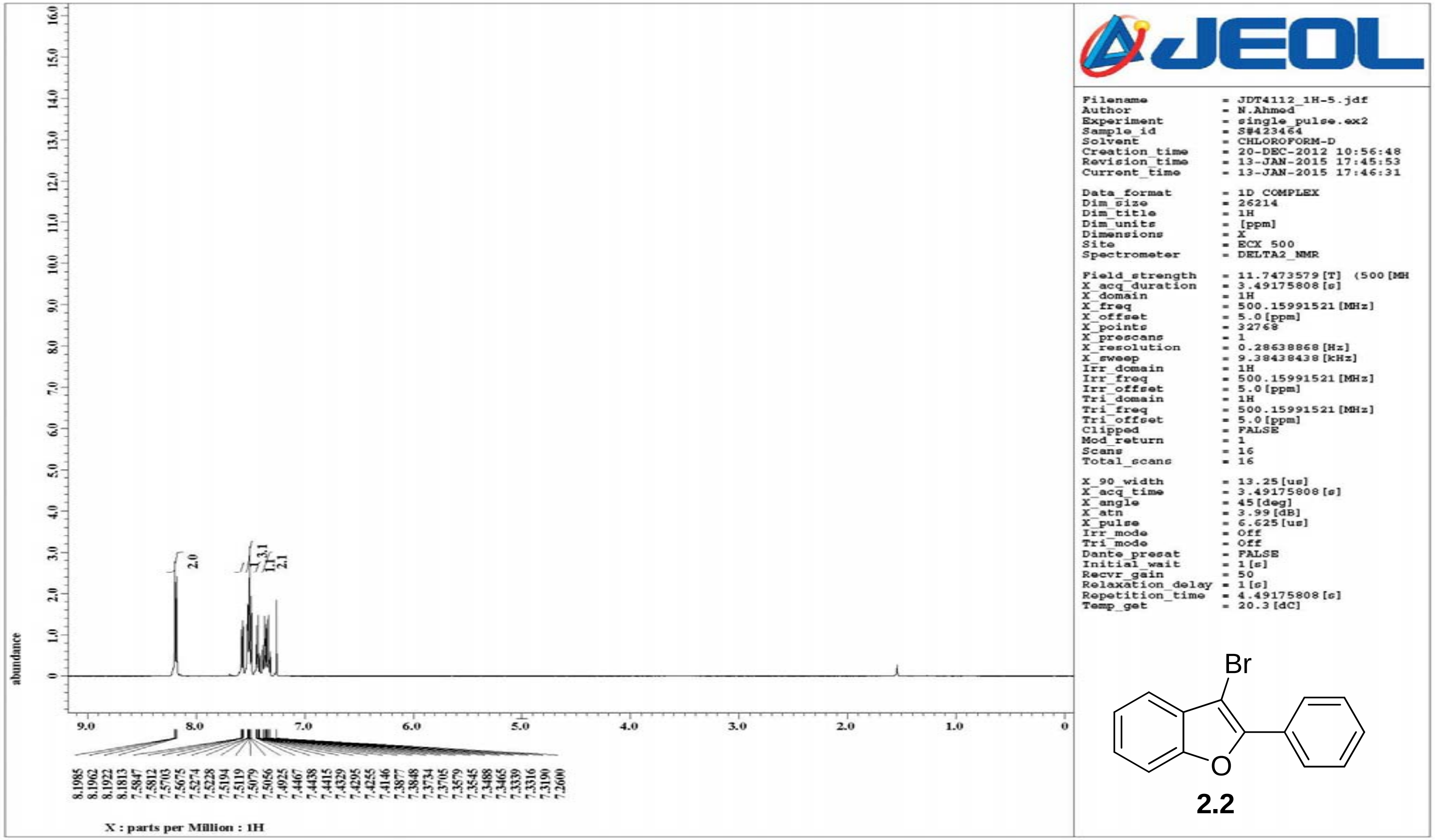

500 MHz ^{1}H NMR spectrum compound **2.2**

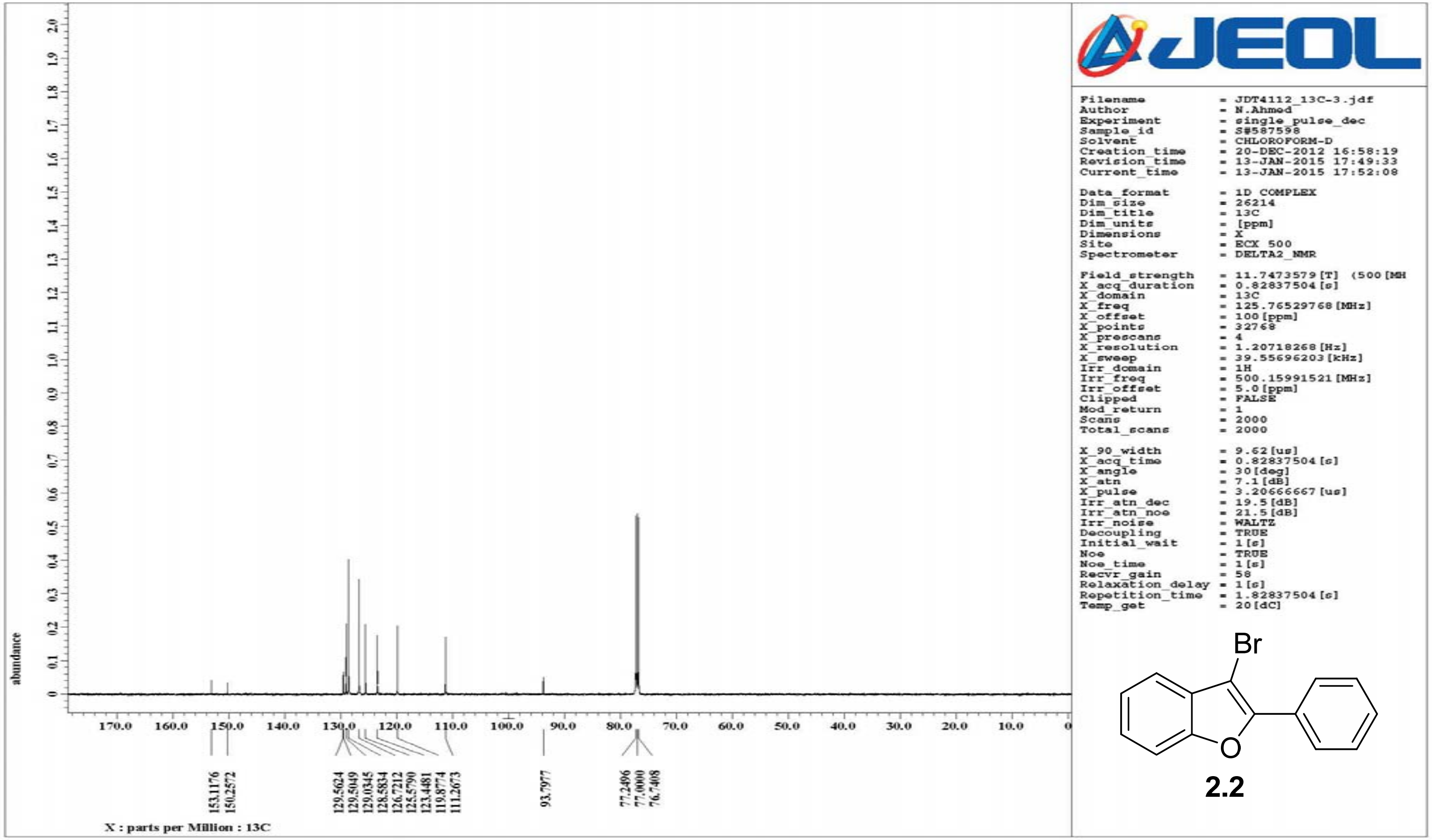

125 MHz ^{13}C NMR spectrum of compound **2.2**

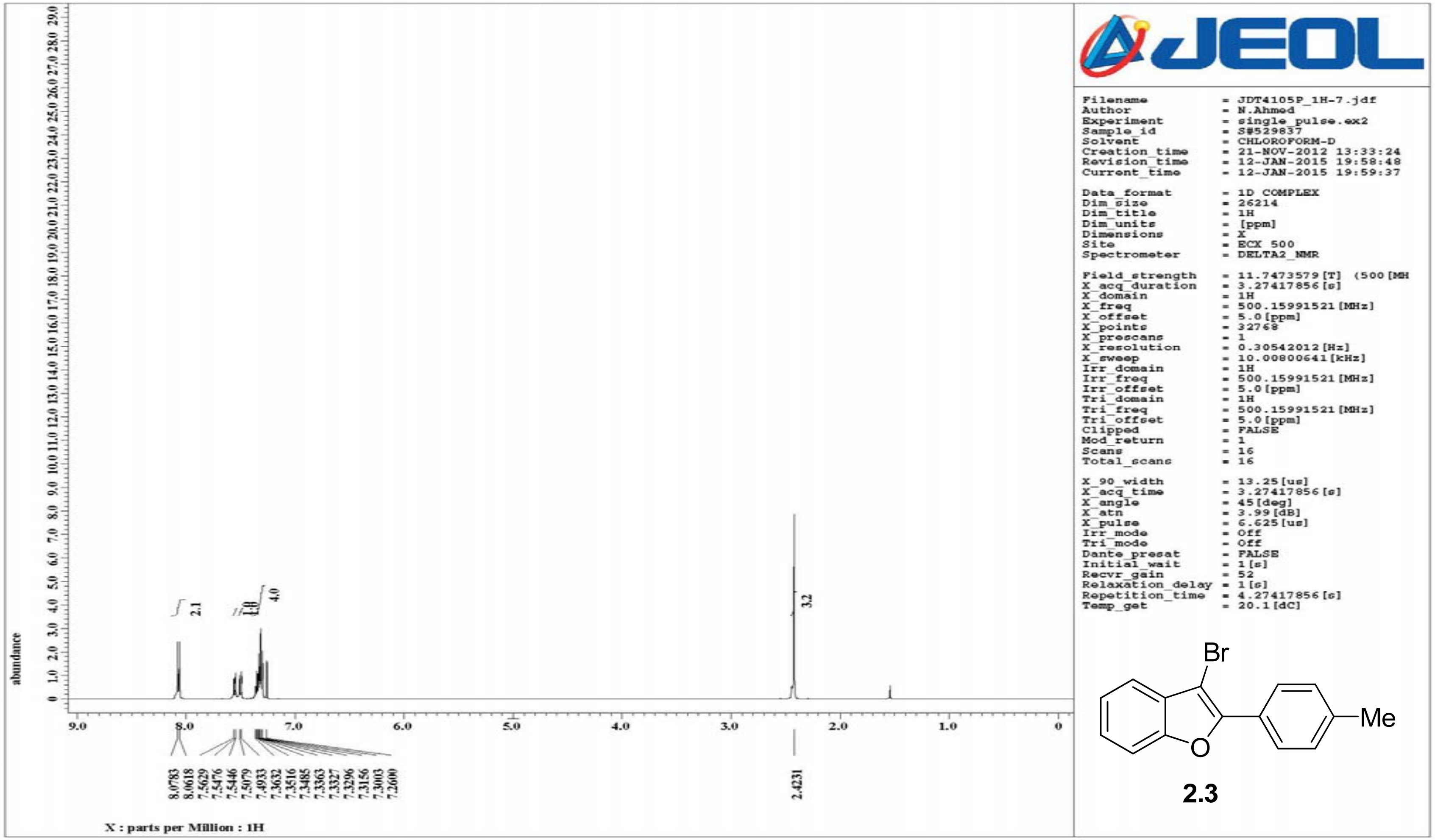

500 MHz ^{1}H NMR spectrum of compound **2.3**

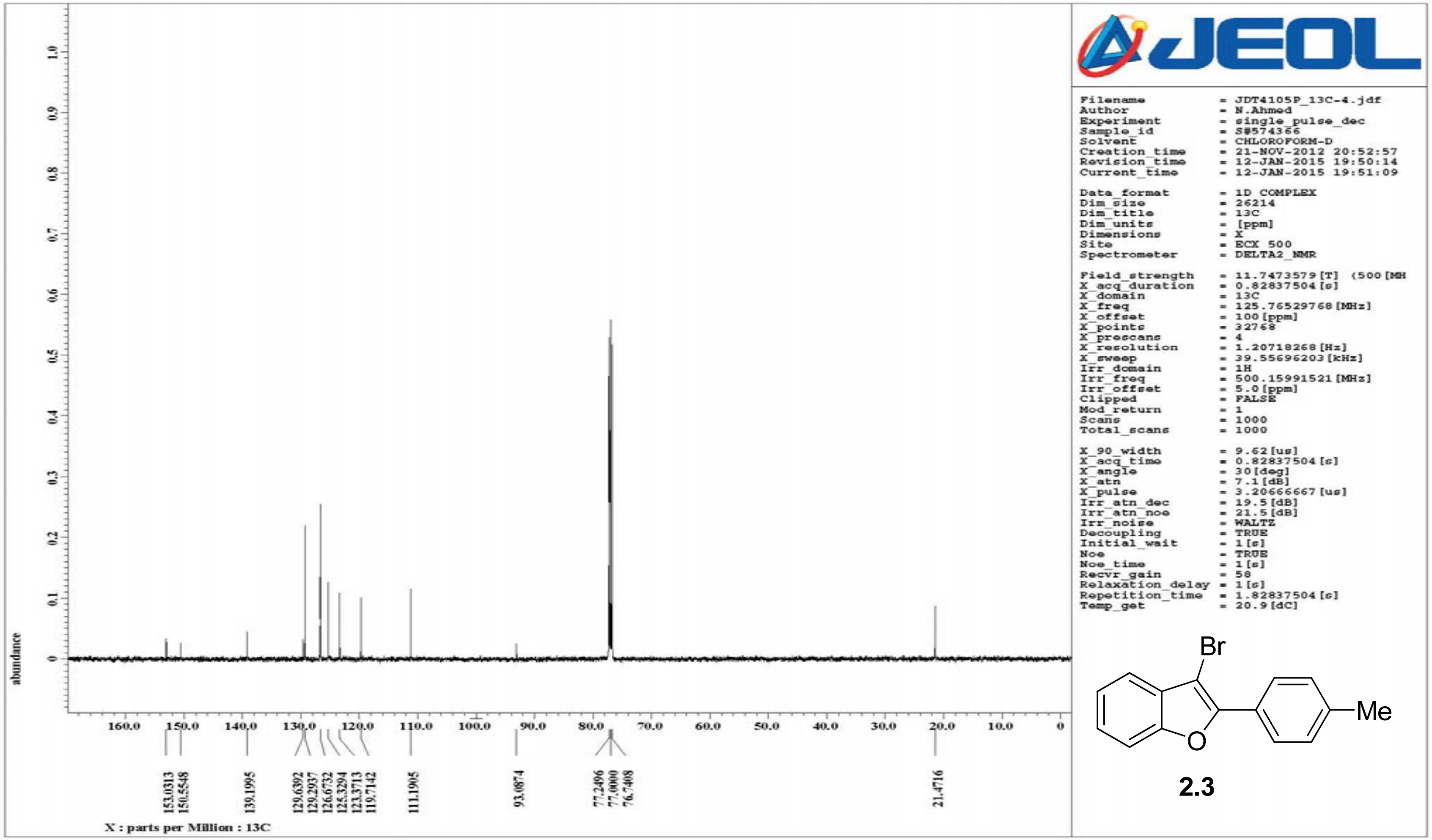

125 MHz ^{13}C NMR spectrum of compound **2.3**

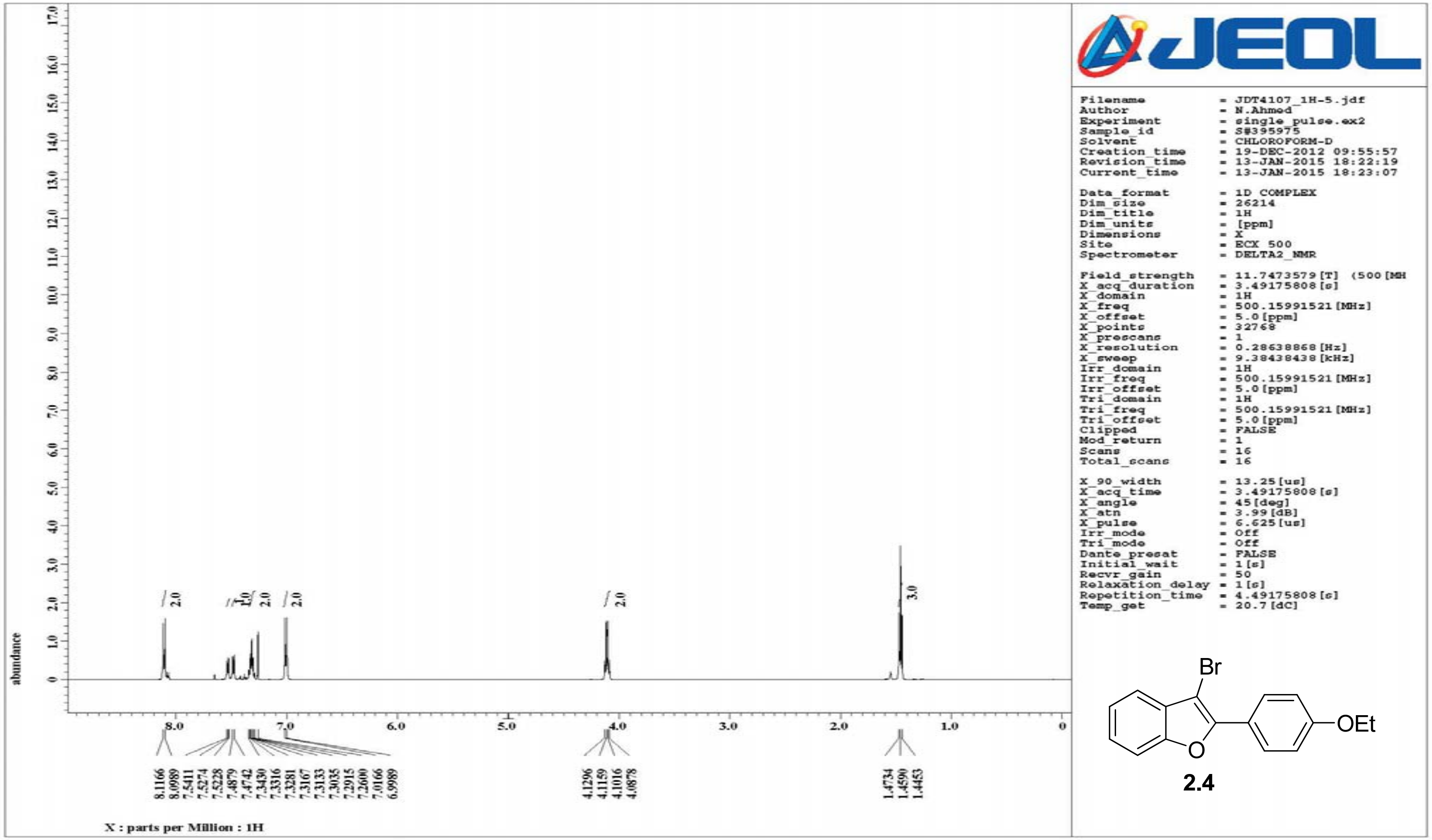

500 MHz ^{1}H NMR spectrum of compound **2.4**

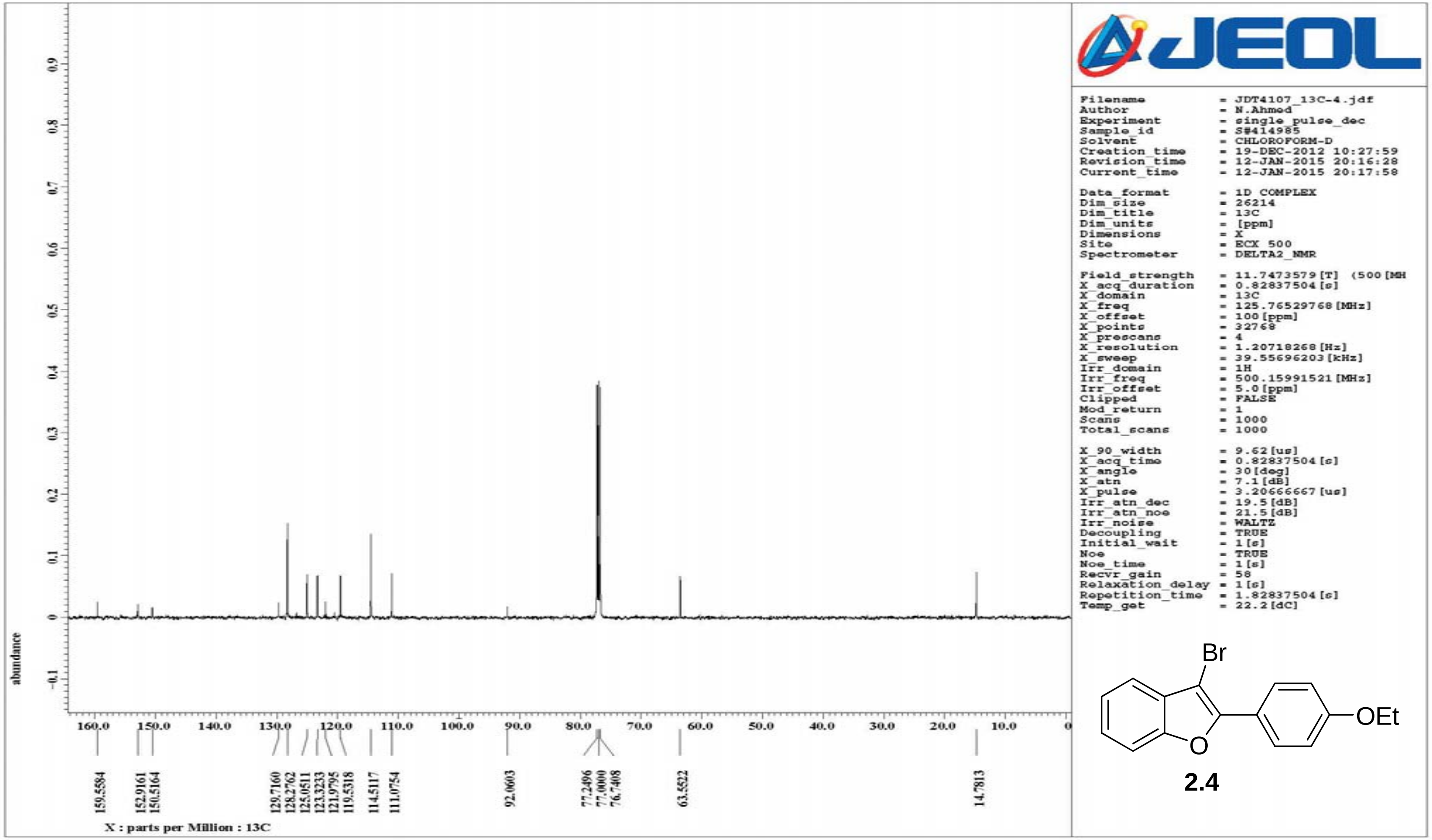

125 MHz ^{13}C NMR spectrum of compound **2.4**

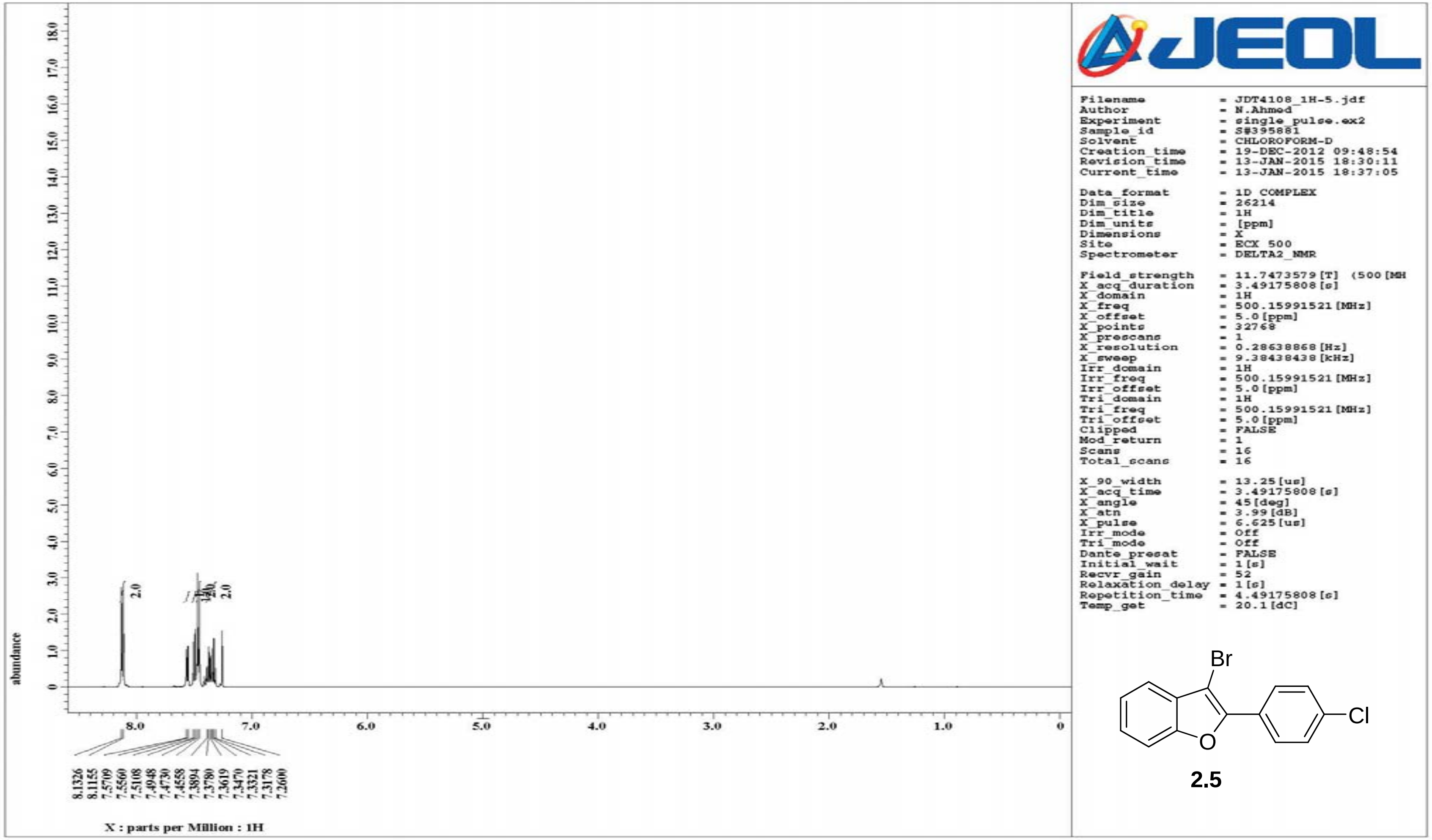

500 MHz ^{1}H NMR spectrum of compound **2.5**

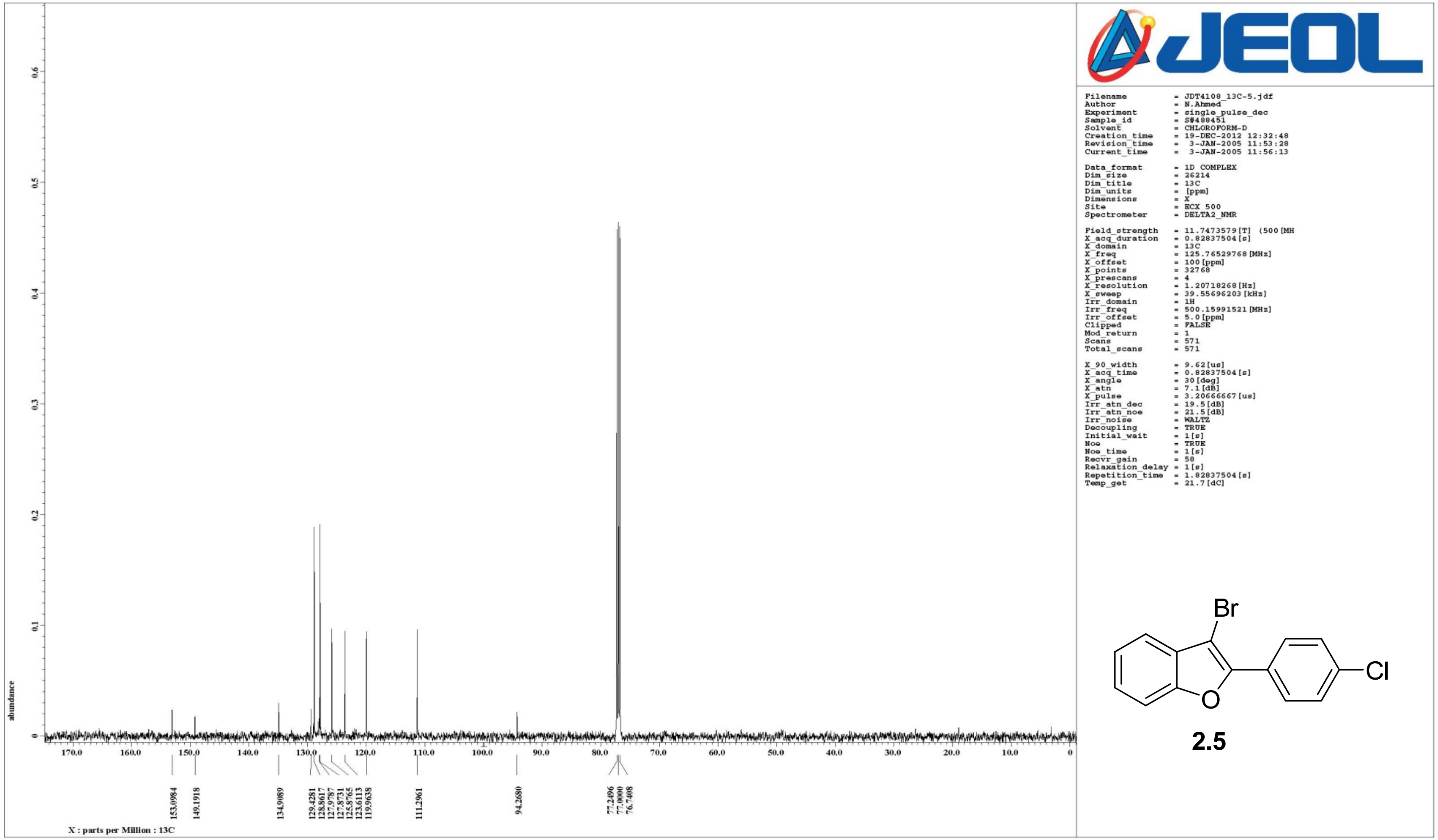

125 MHz ^{13}C NMR spectrum of compound **2.5**

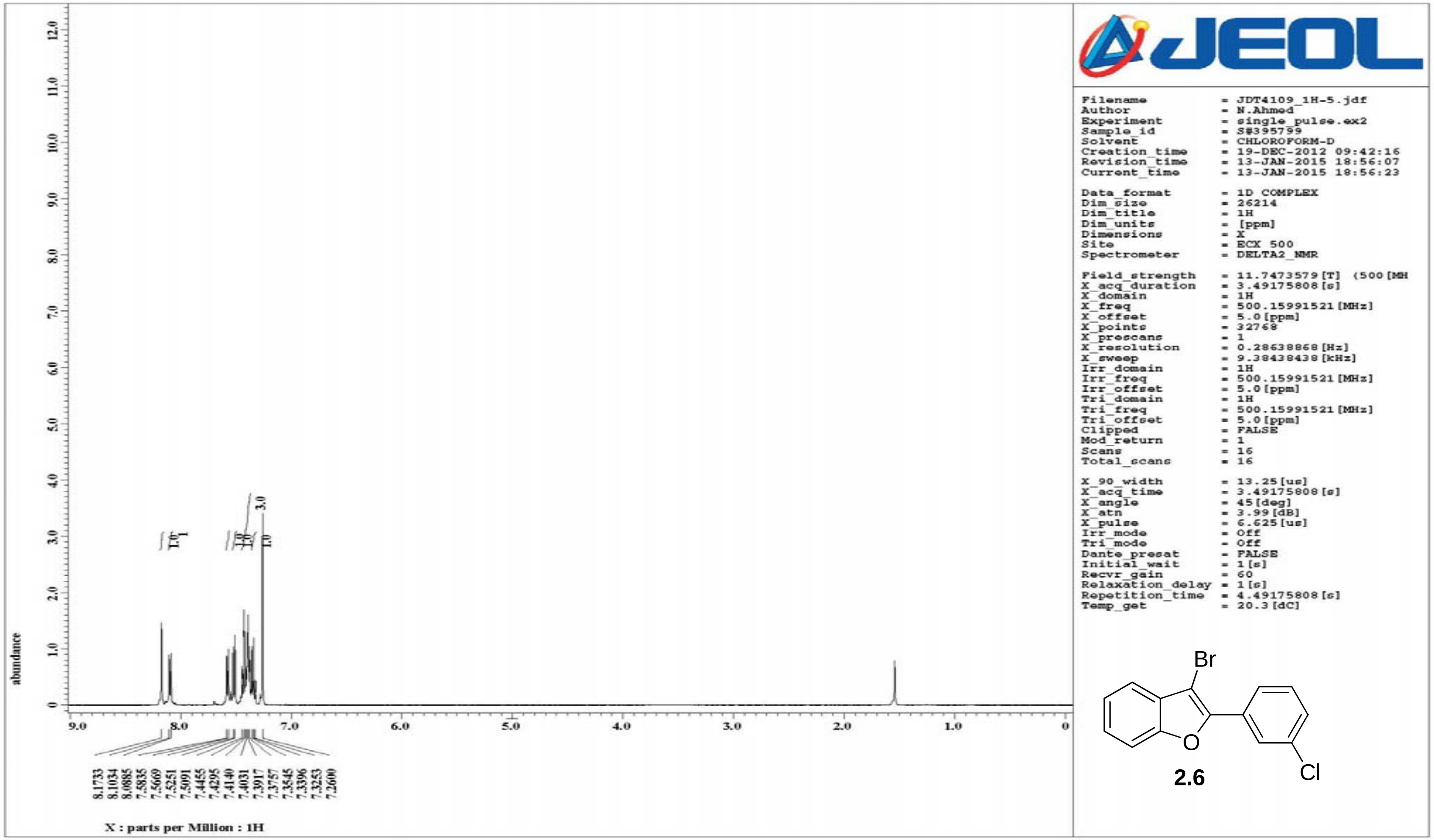

500 MHz ^{1}H NMR spectrum of compound **2.6**

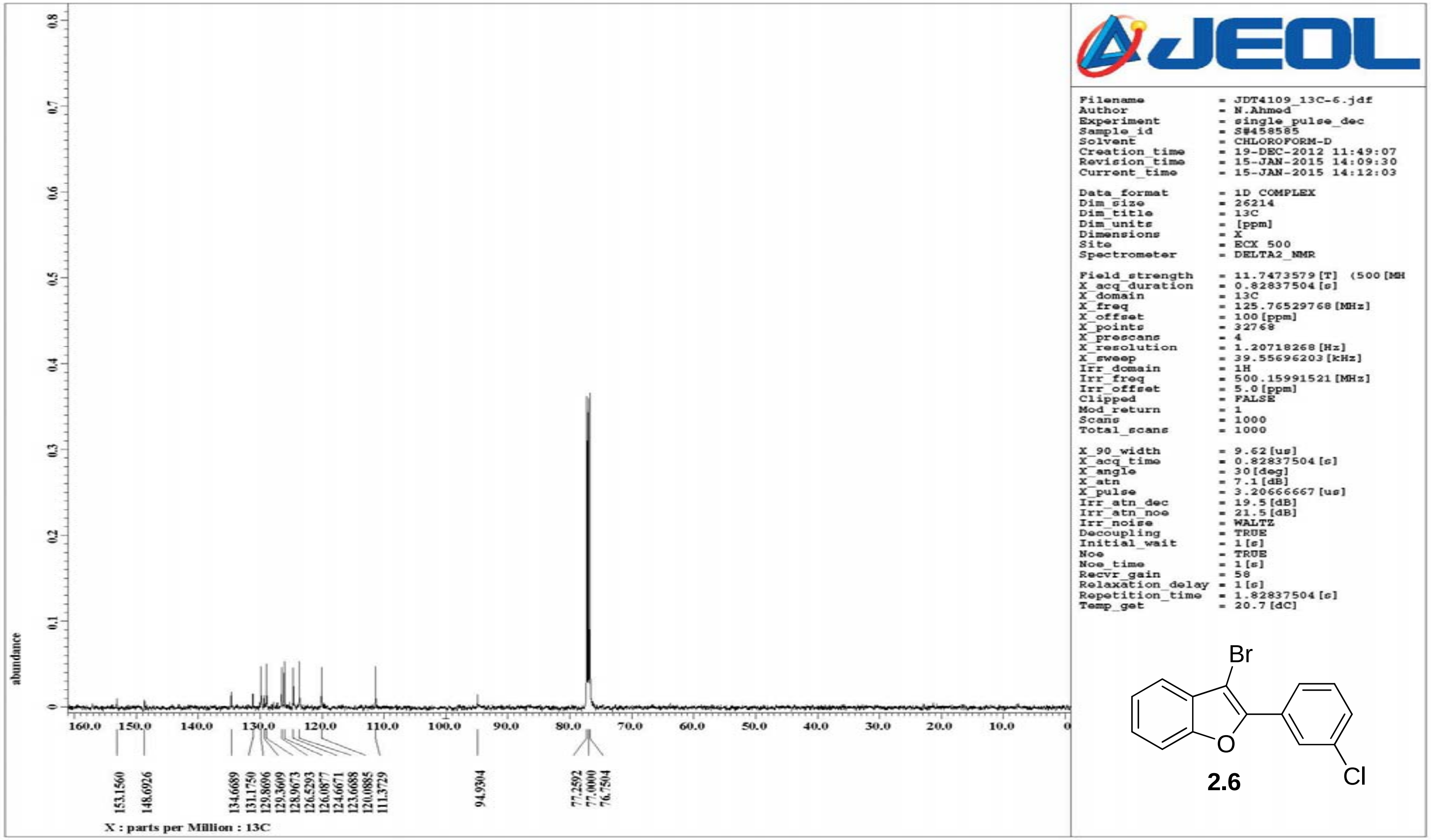

125 MHz ^{13}C NMR spectrum of compound **2.6**

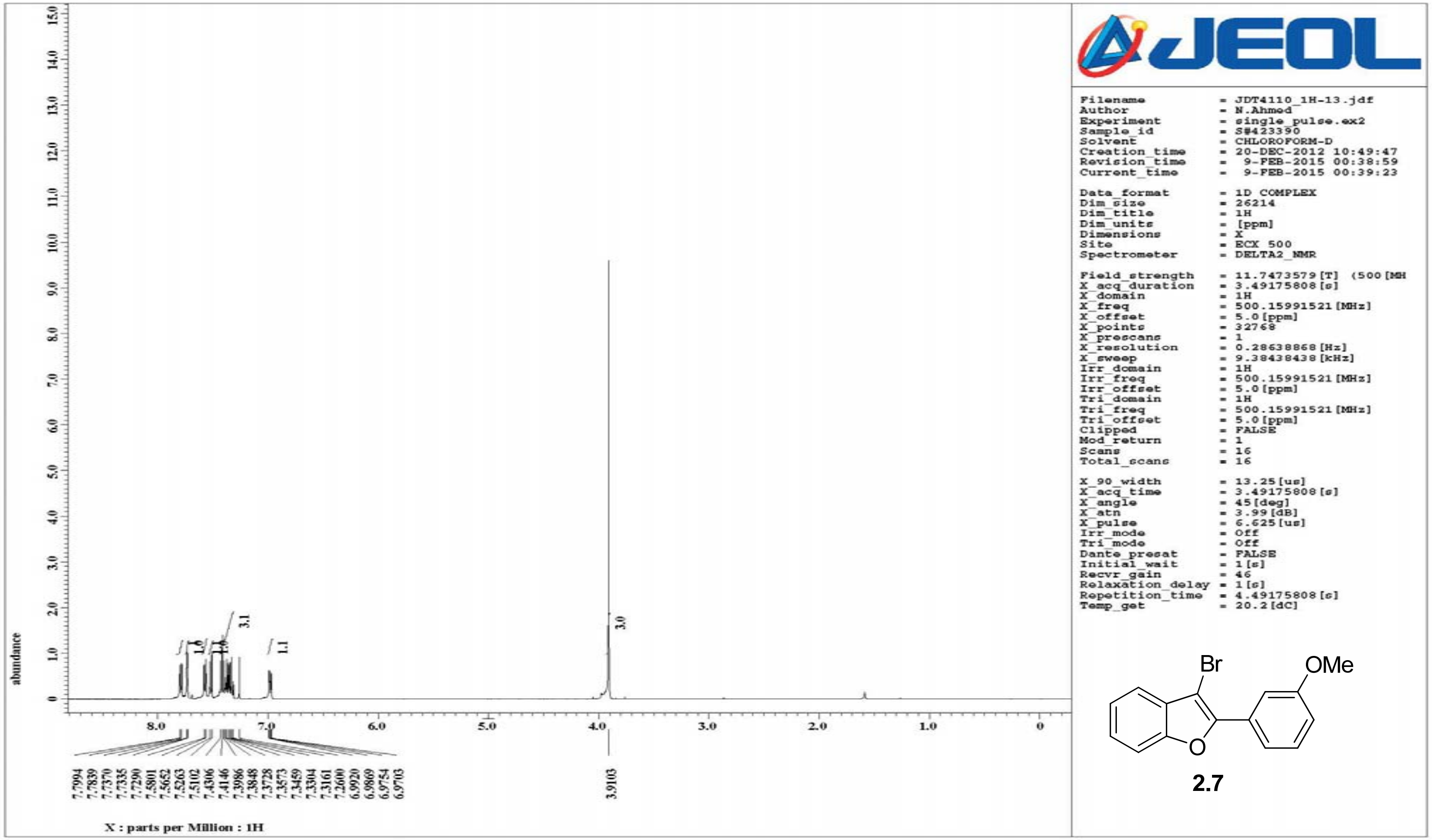

500 MHz ^{1}H NMR spectrum of compound **2.7**

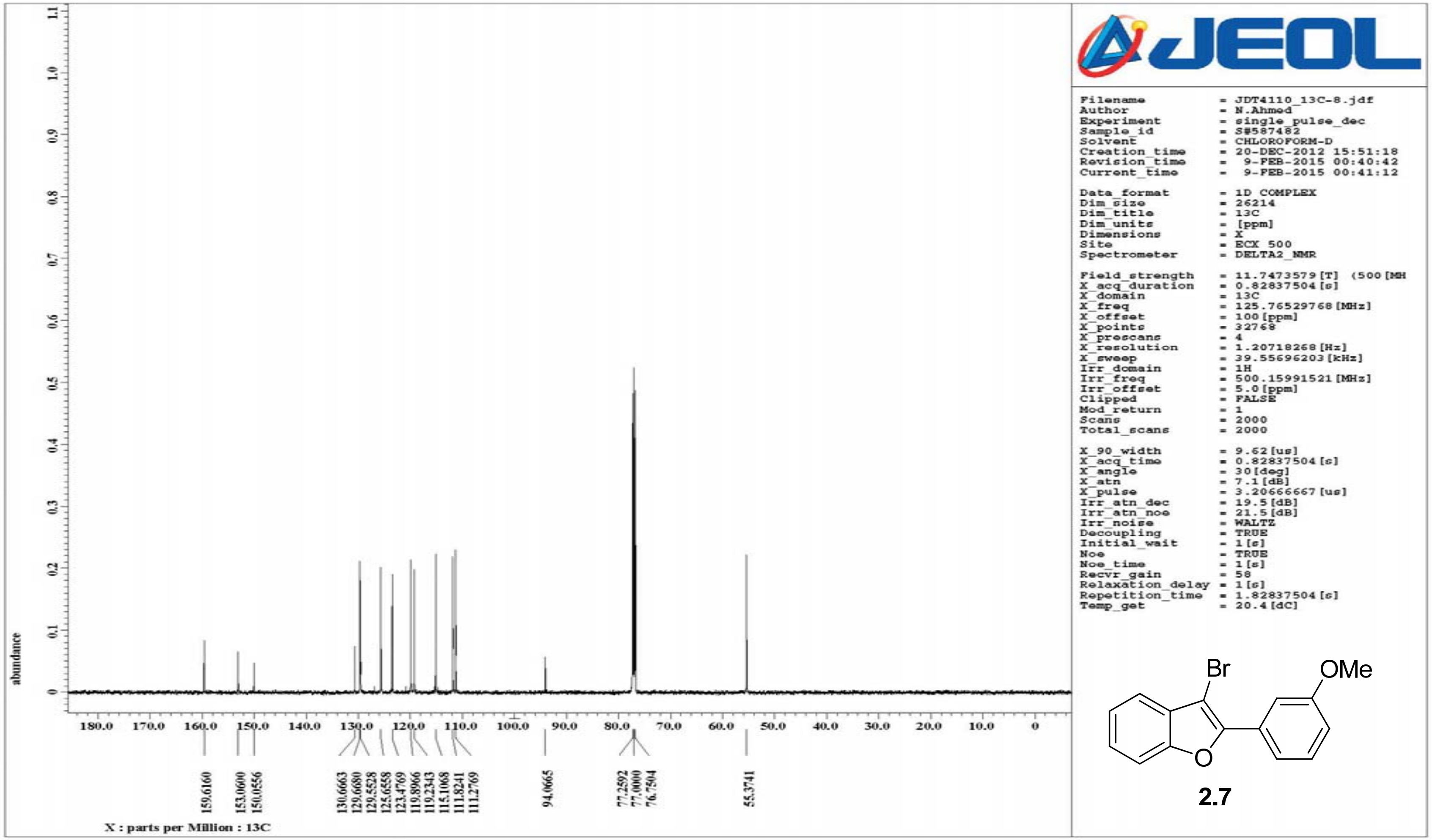

2.7

125 MHz ^{13}C NMR spectrum of compound **2.7**

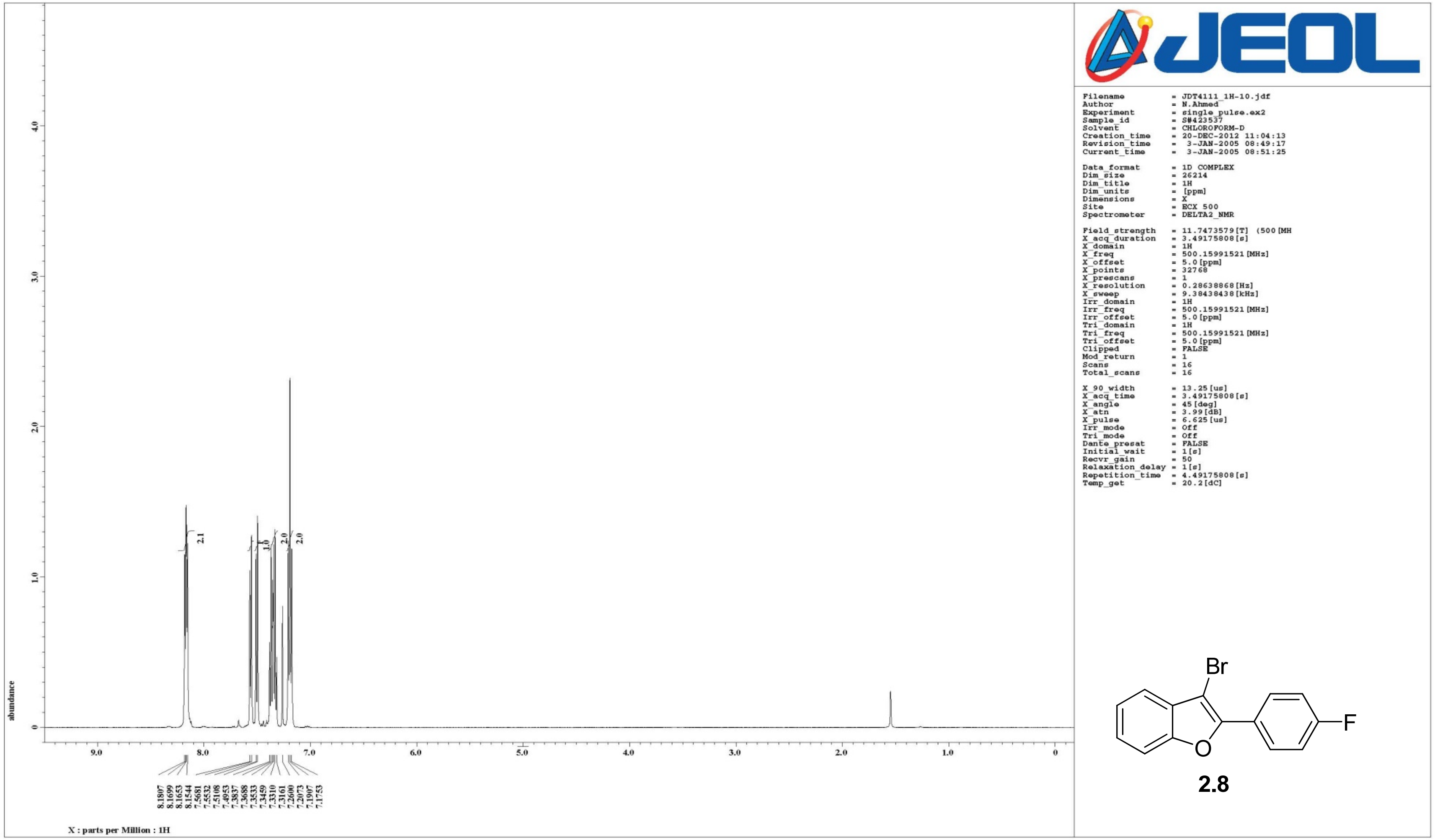

2.8

500 MHz ^{1}H NMR spectrum of compound **2.8**

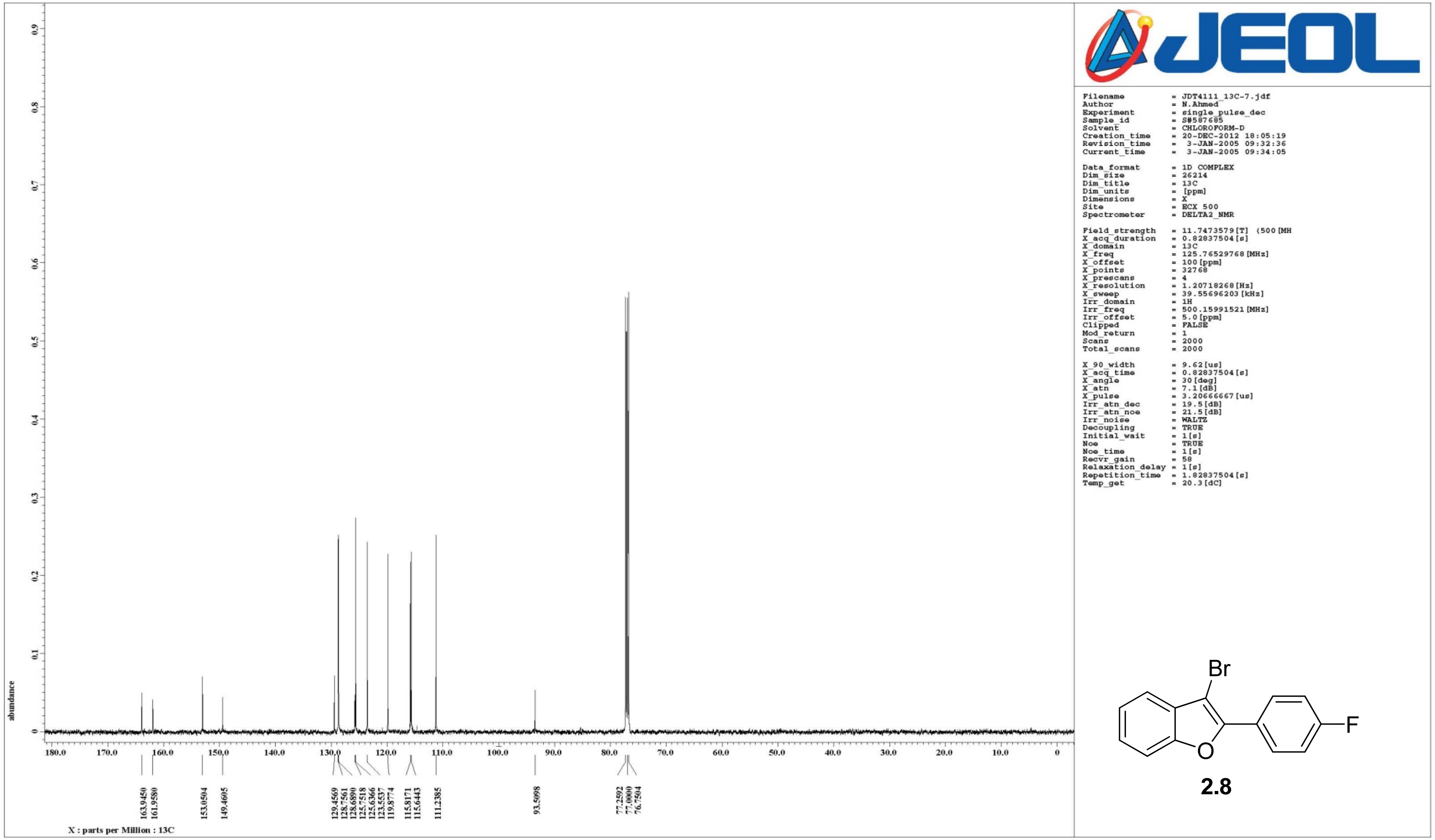

125 MHz ^{13}C NMR spectrum of compound **2.8**

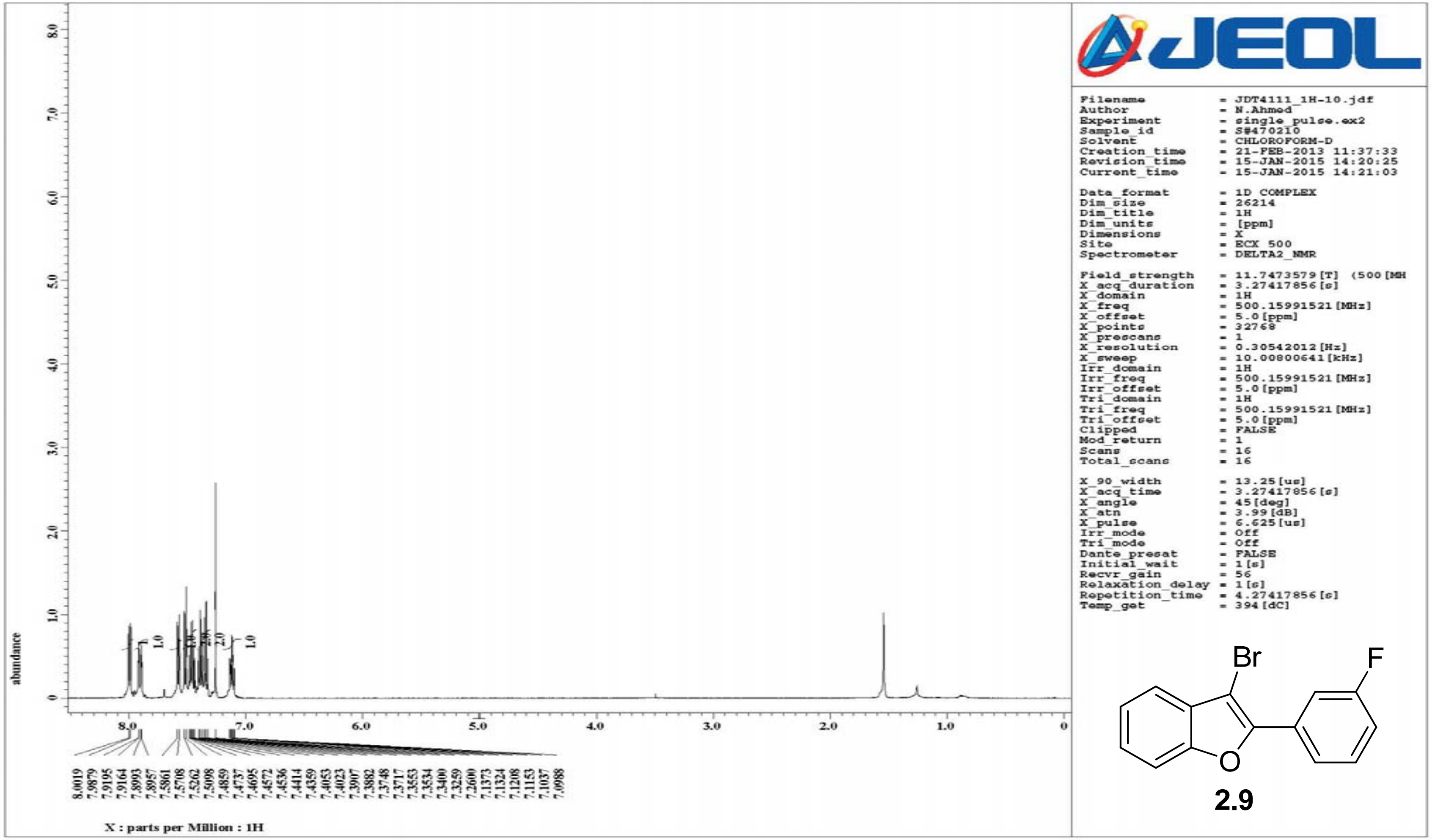

500 MHz ^{1}H NMR spectrum of compound **2.9**

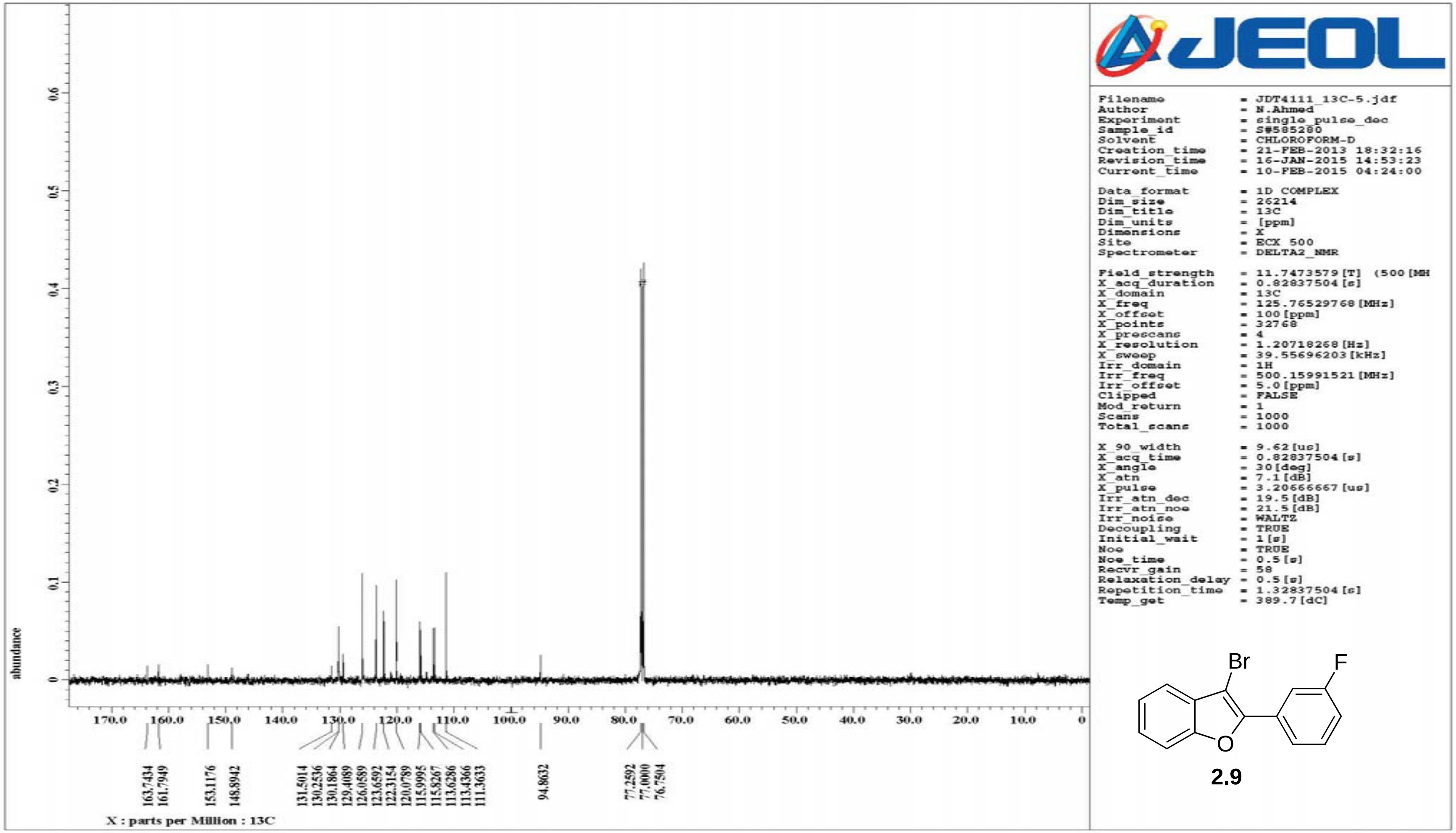

125 MHz ^{13}C NMR spectrum of compound **2.9**

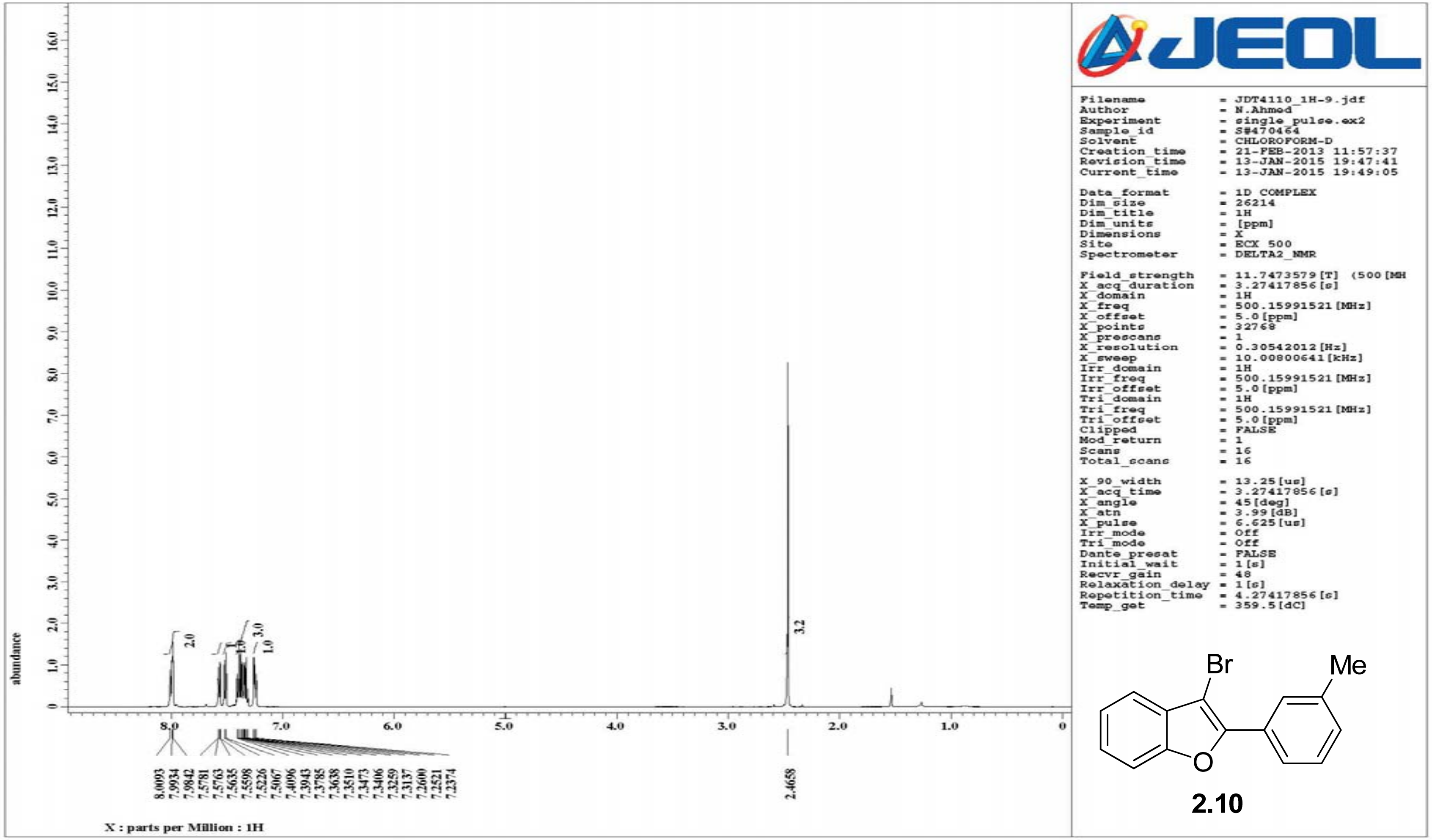

500 MHz ^{1}H NMR spectrum of compound **2.10**

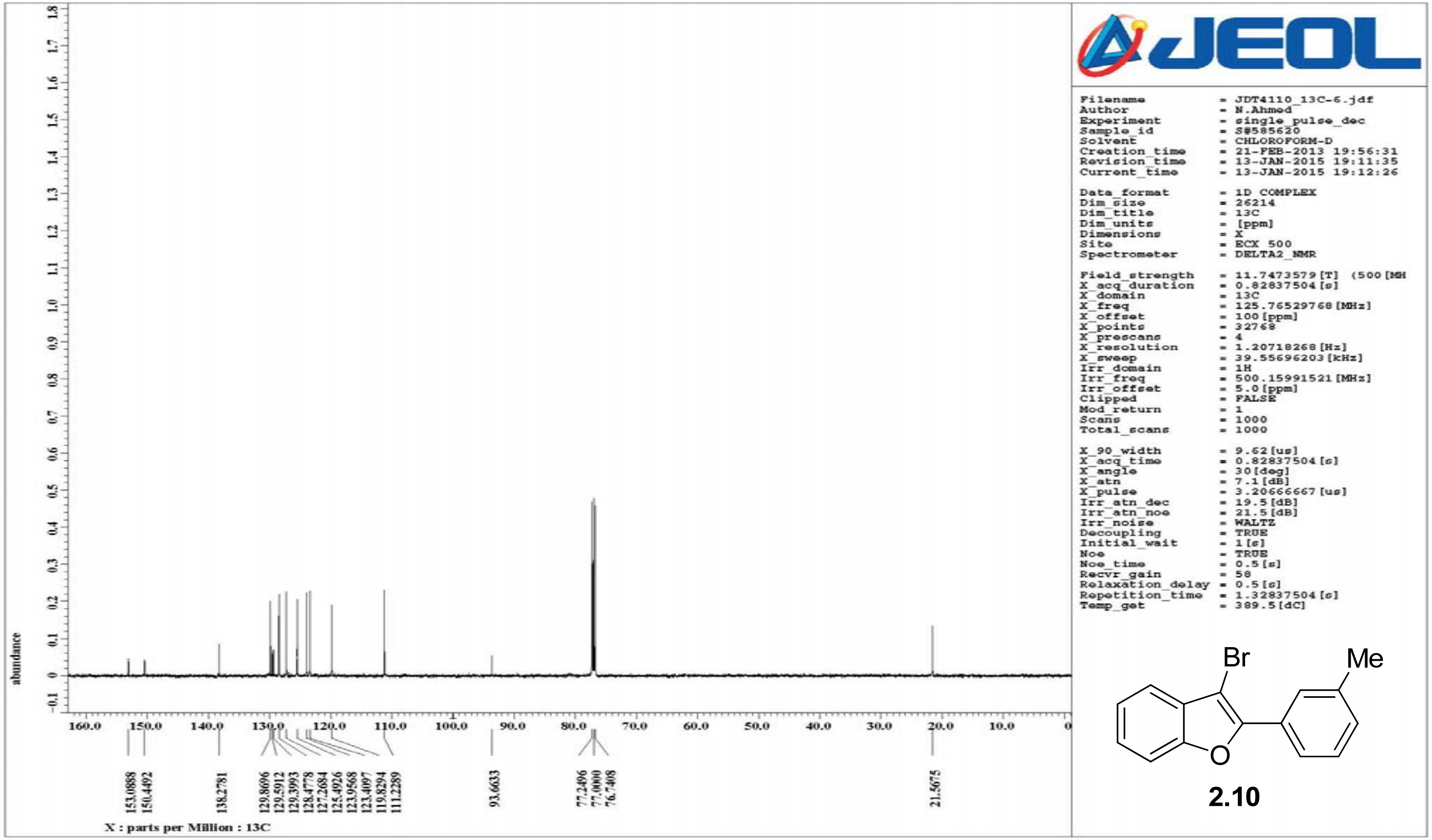

2.10

125 MHz ^{13}C NMR spectrum of compound **2.10**

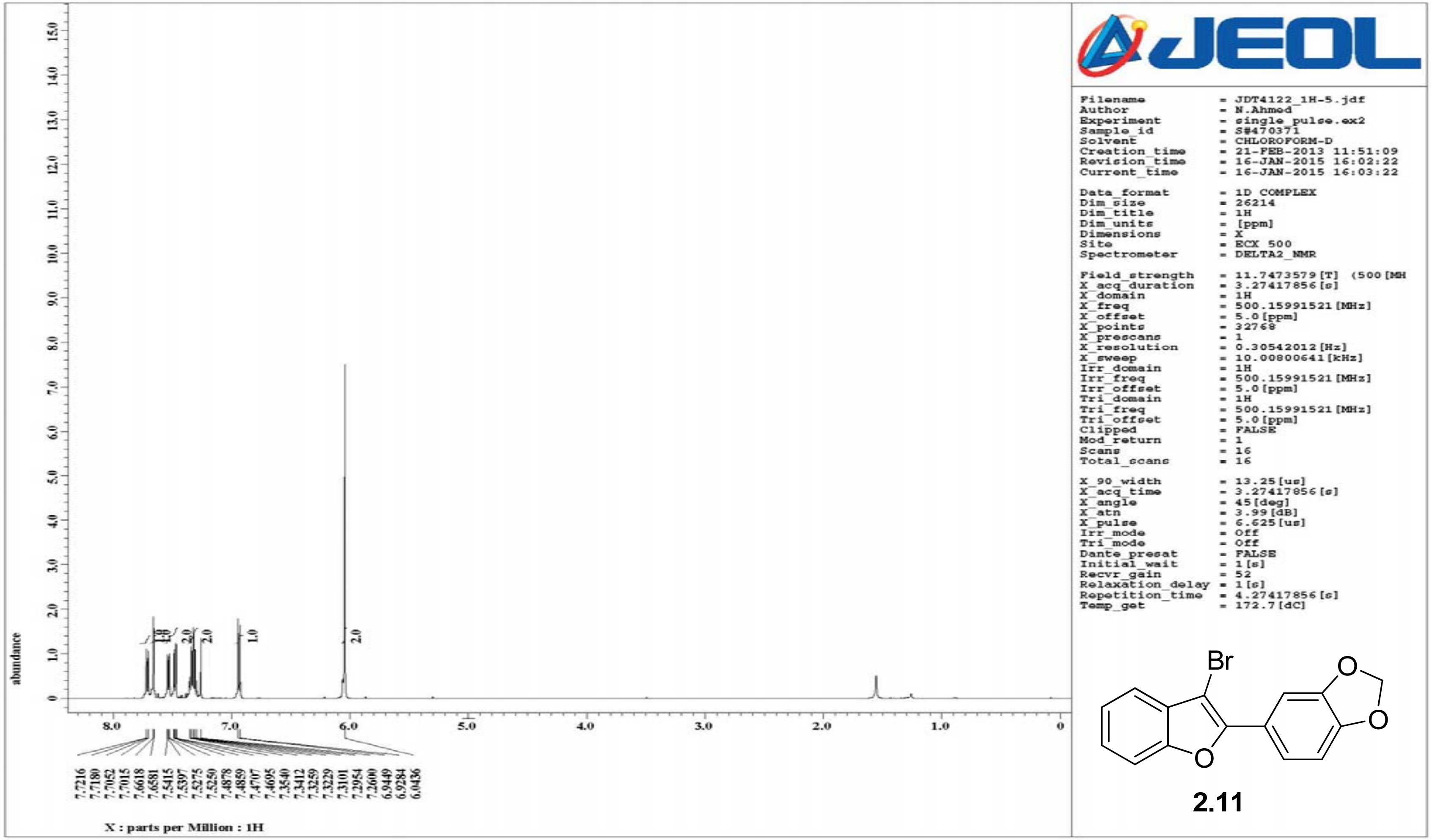

500 MHz ^{1}H NMR spectrum of compound **2.11**

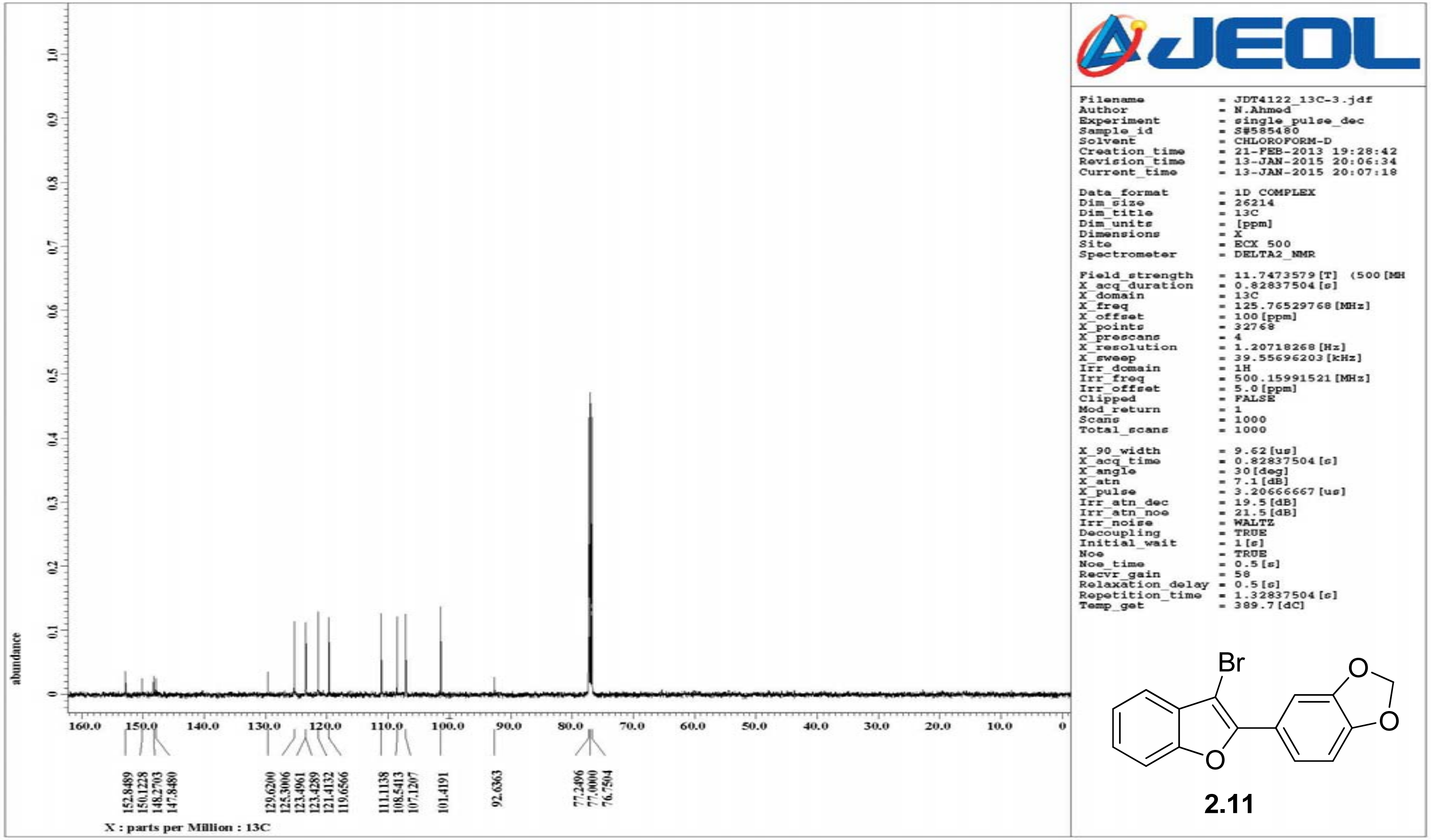

125 MHz ^{13}C NMR spectrum of compound **2.11**

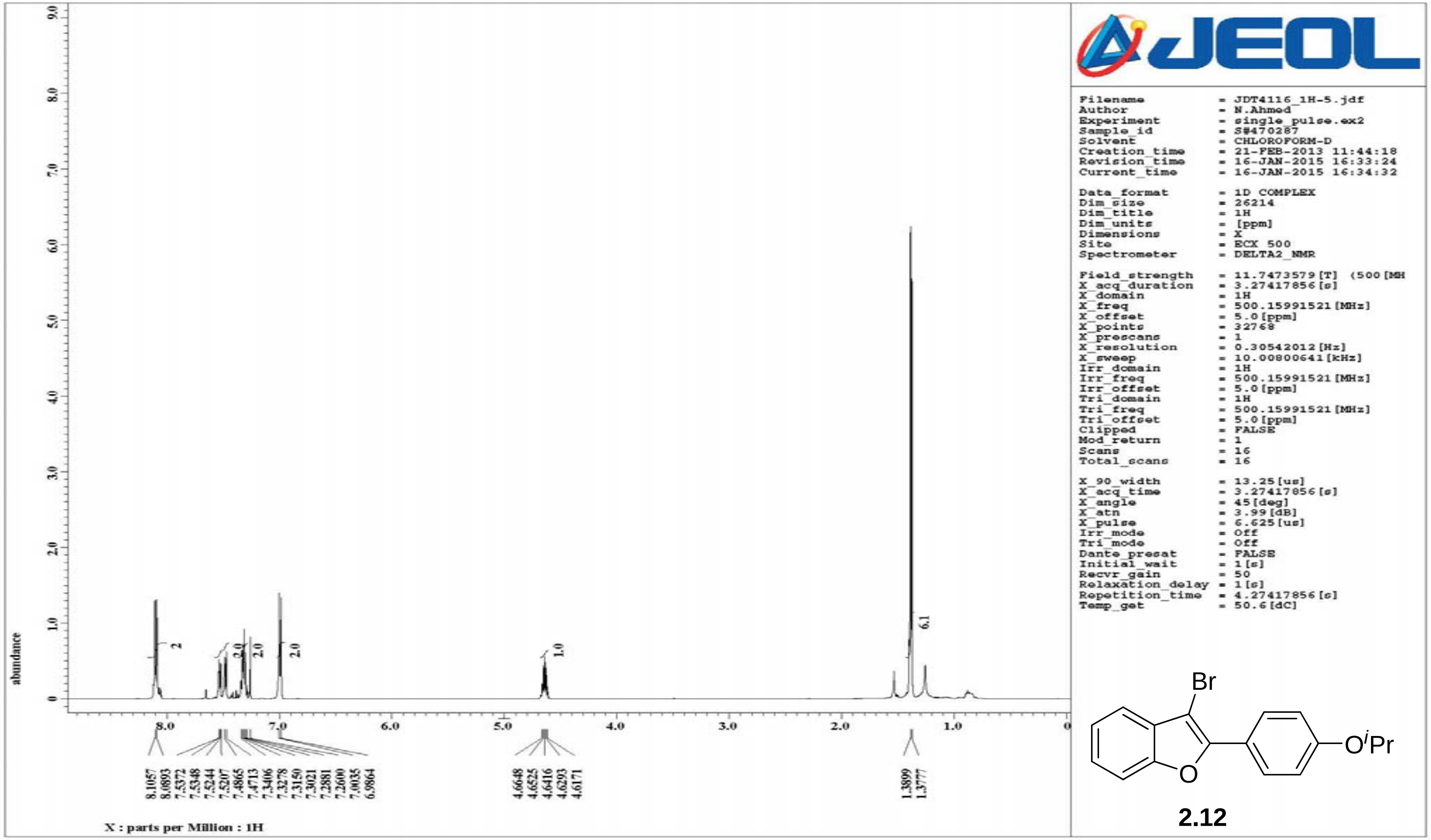

2.12

500 MHz ^{1}H NMR spectrum of compound **2.12**

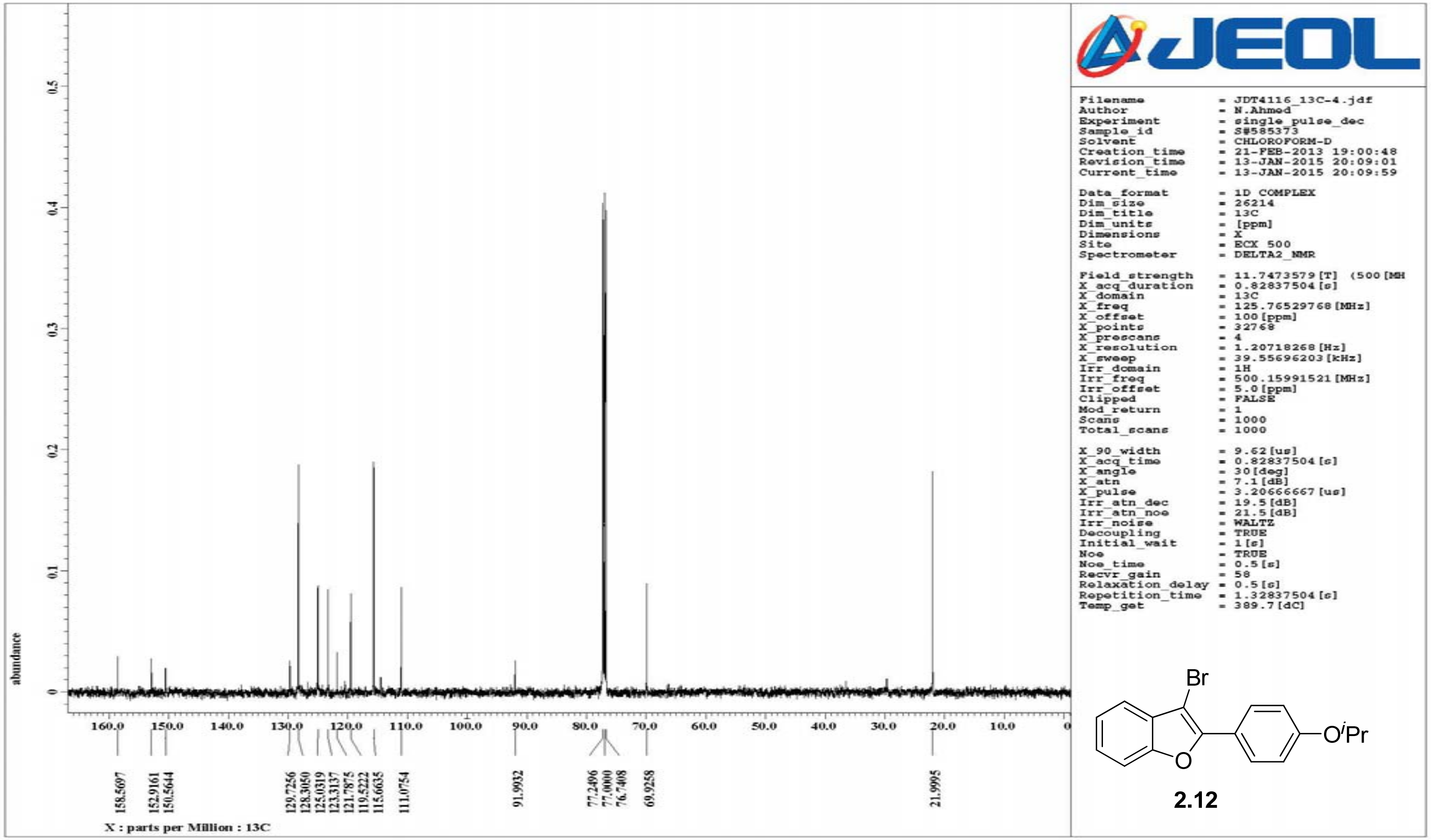

125 MHz ^{13}C NMR spectrum of compound **2.12**

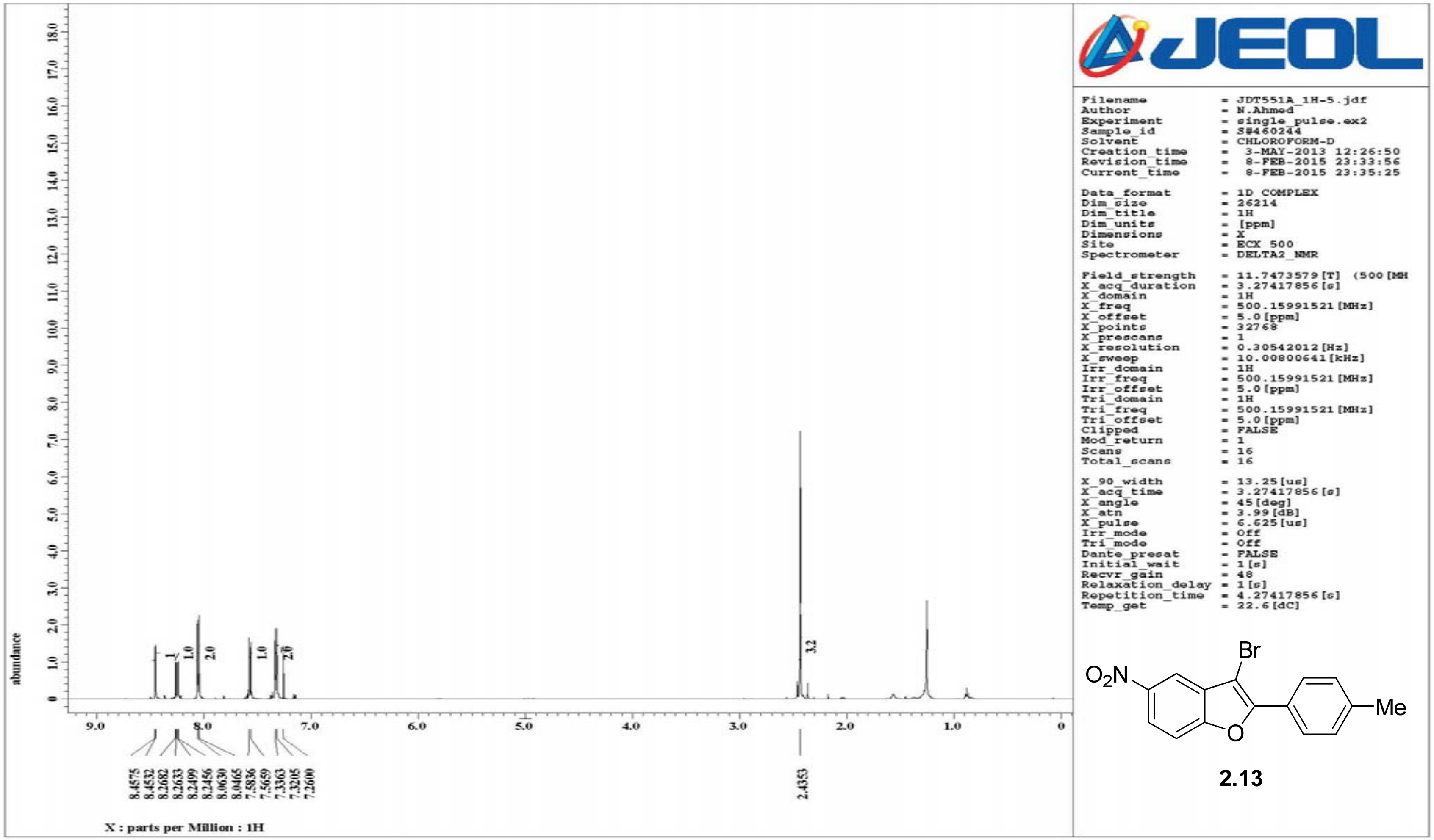

500 MHz ^{1}H NMR spectrum of compound **2.13**

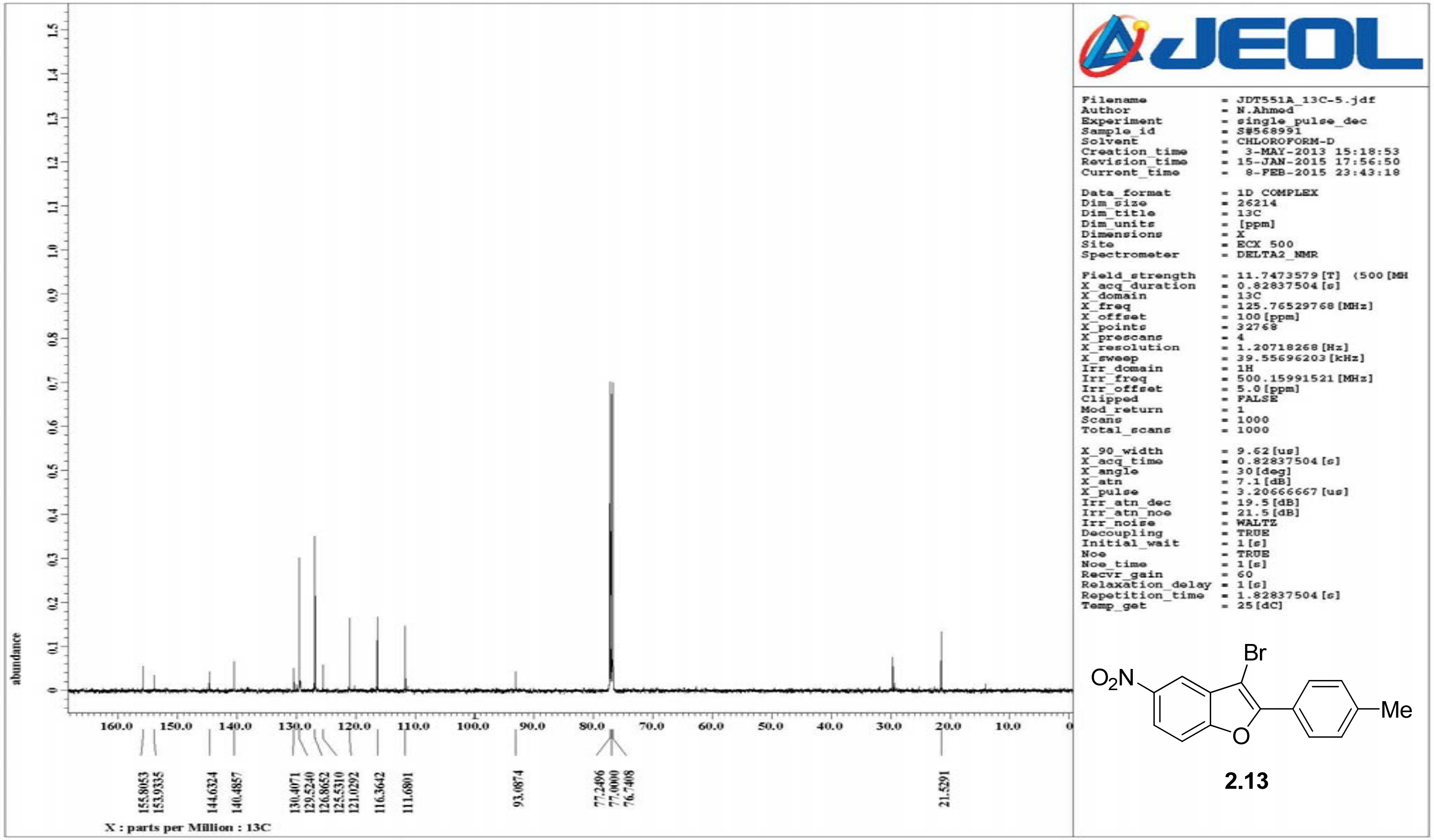

125 MHz ^{13}C NMR spectrum of compound **2.13**

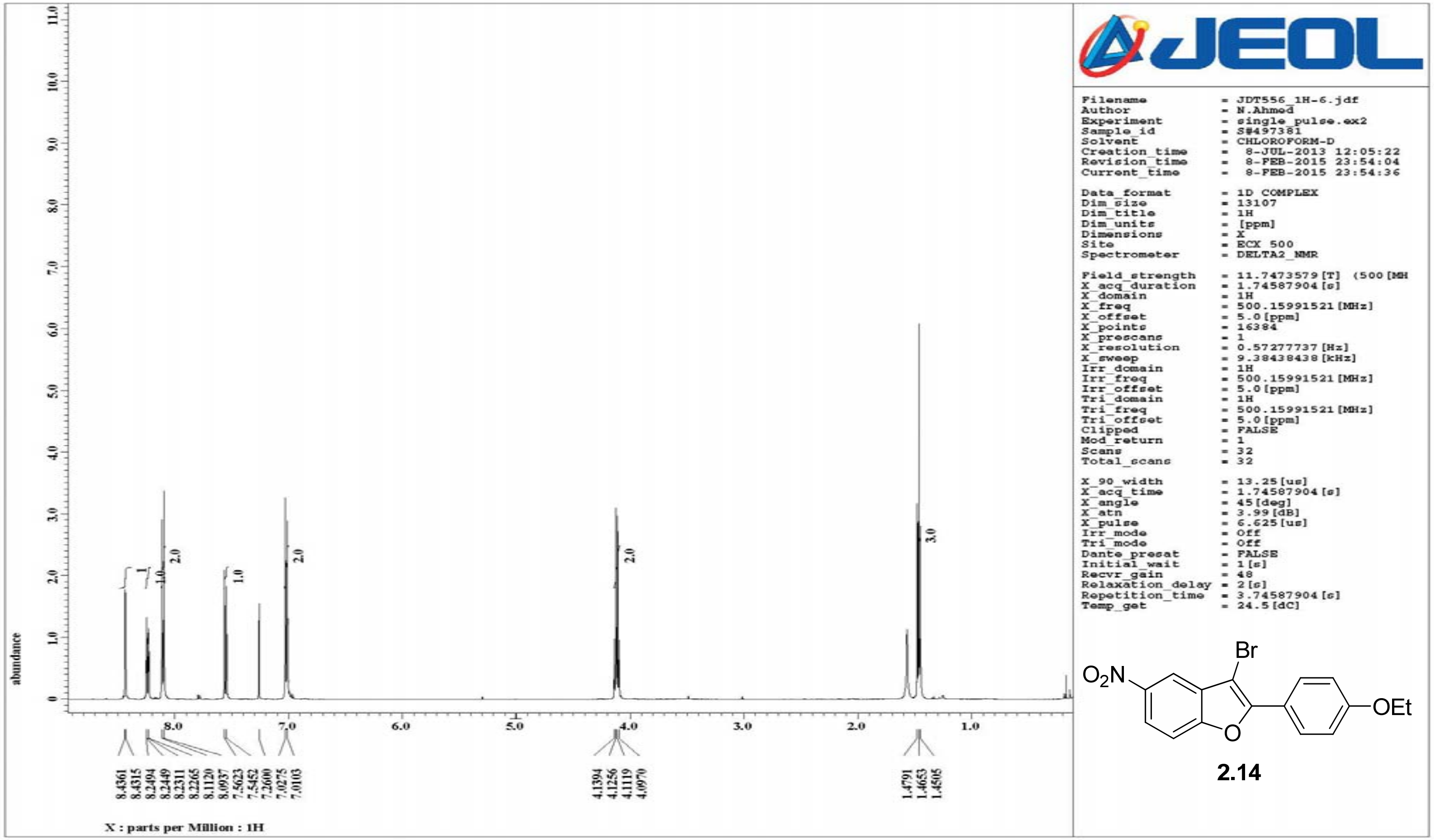

500 MHz ^{1}H NMR spectrum of compound **2.14**

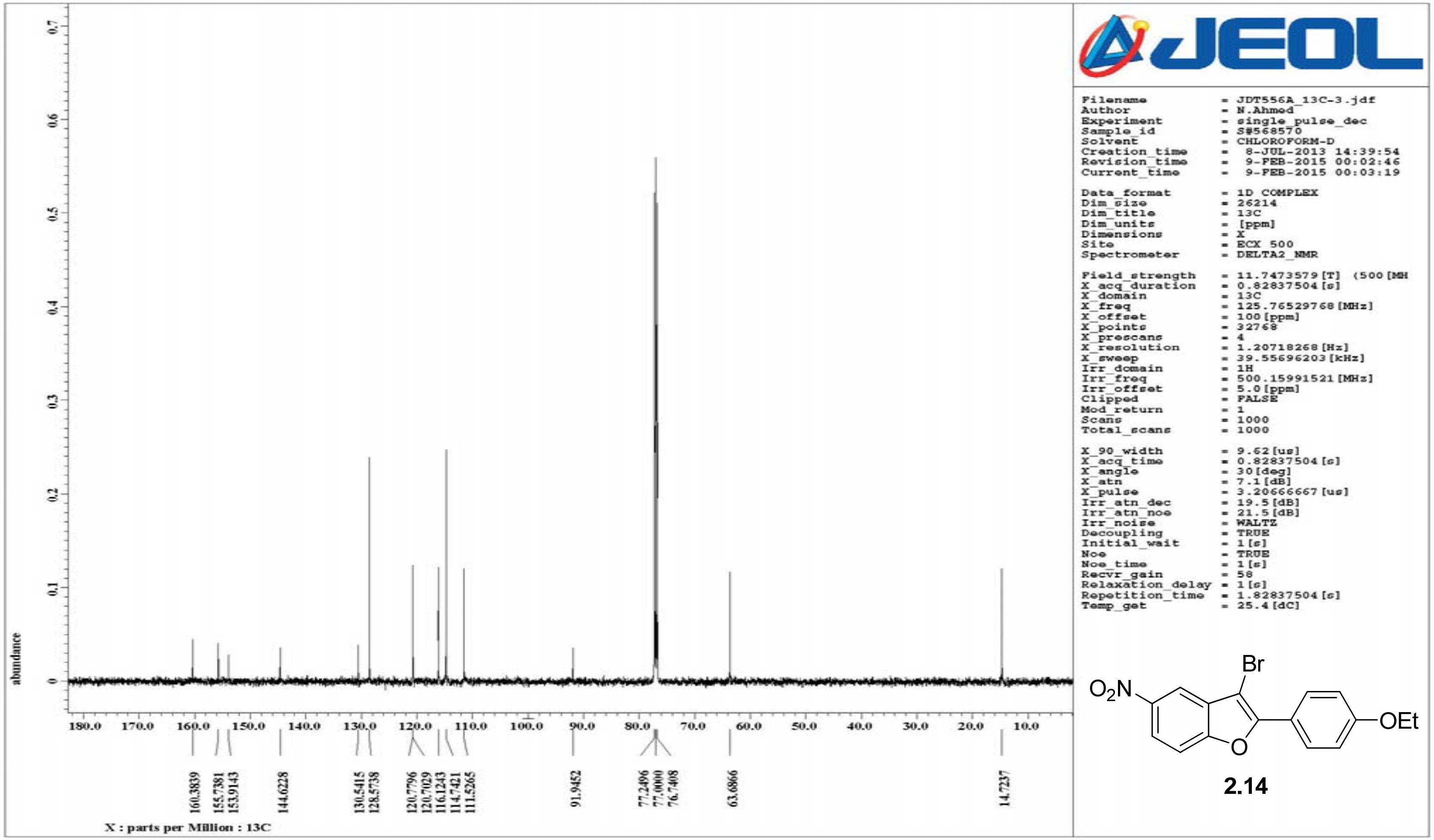

125 MHz ^{13}C NMR spectrum of compound **2.14**

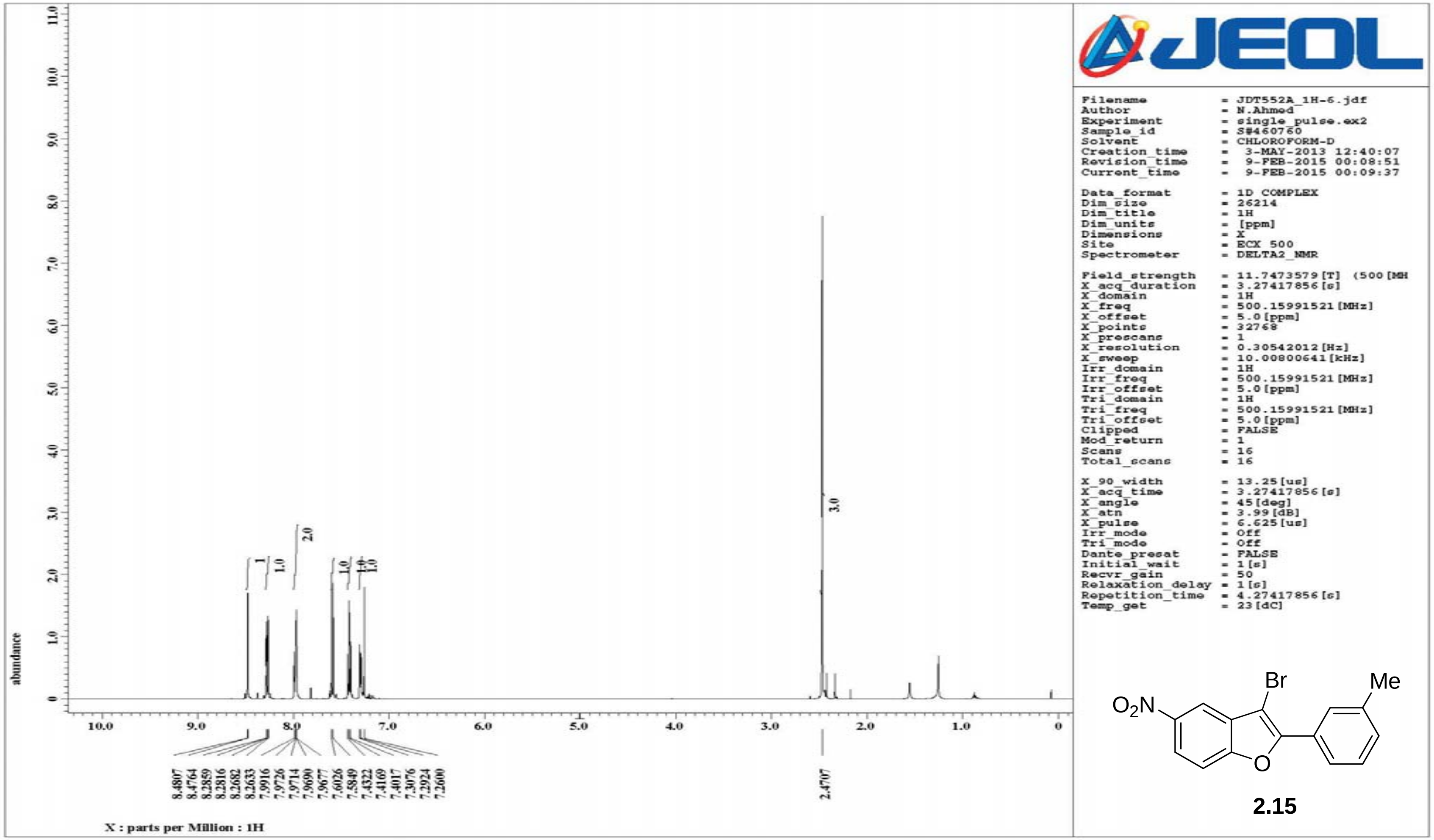

500 MHz ^{1}H NMR spectrum of compound **2.15**

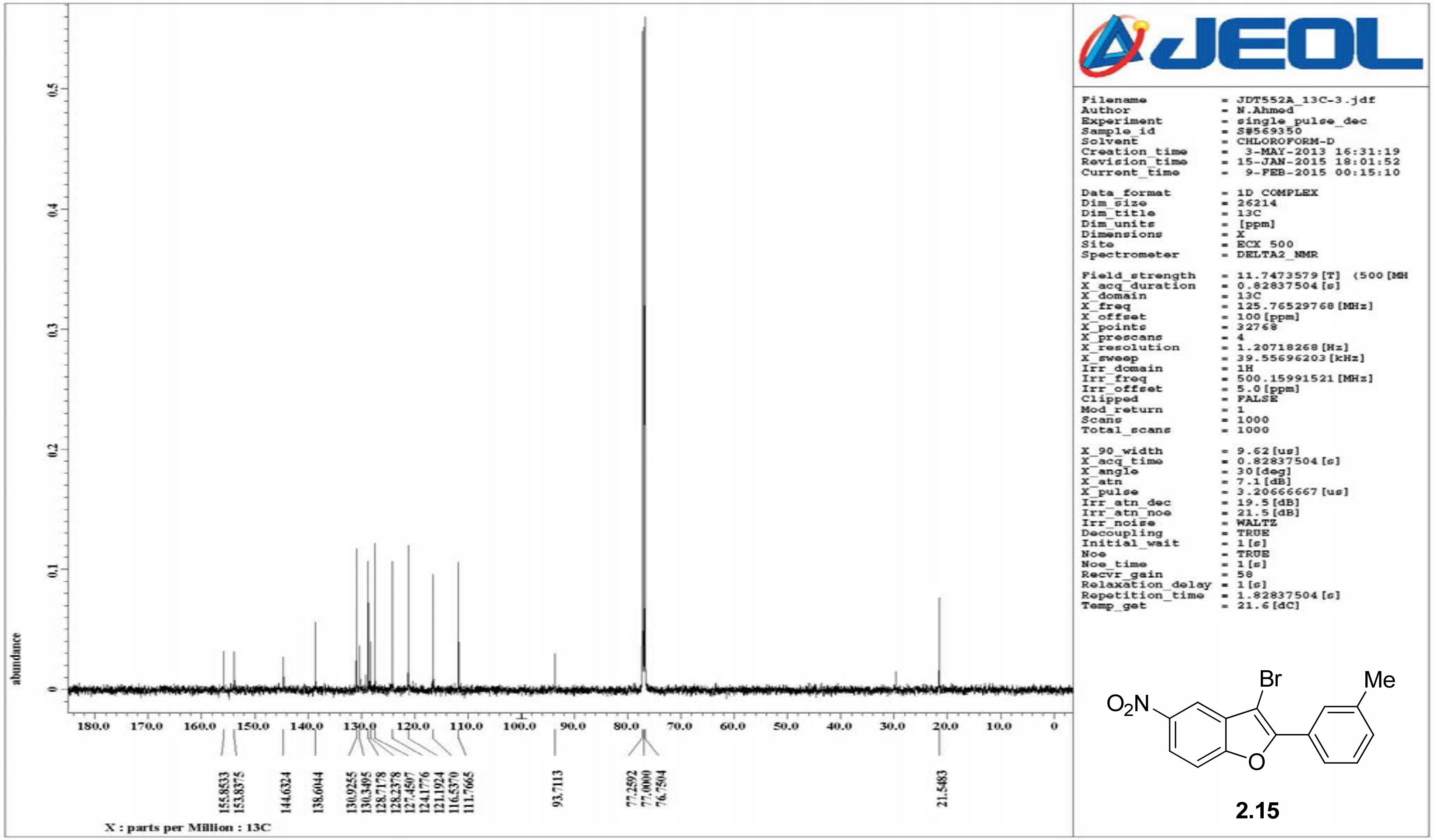

125 MHz ^{13}C NMR spectrum of compound **2.15**

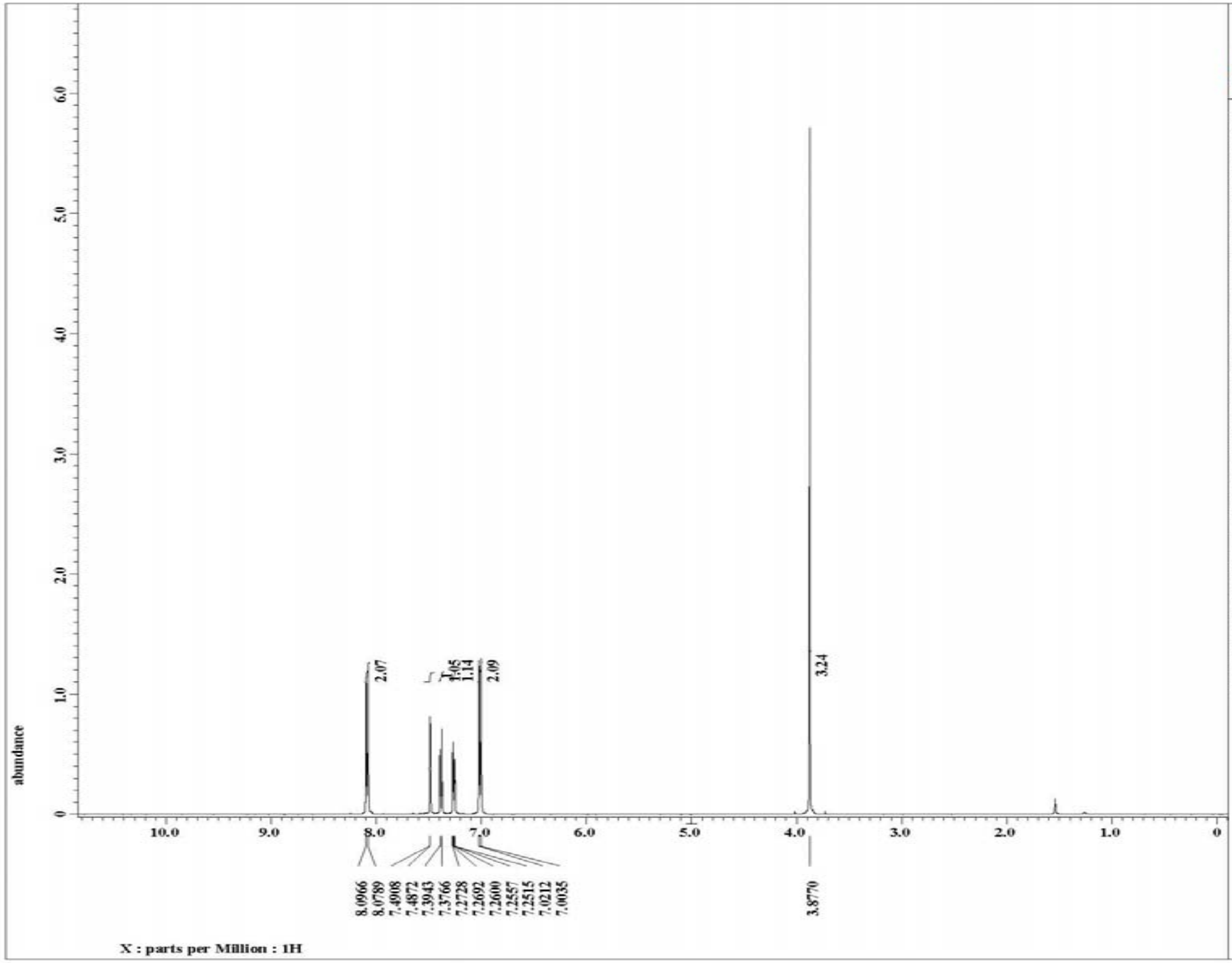

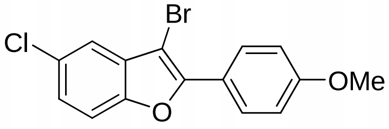

2.16

500 MHz ^{1}H NMR spectrum of compound **2.16**

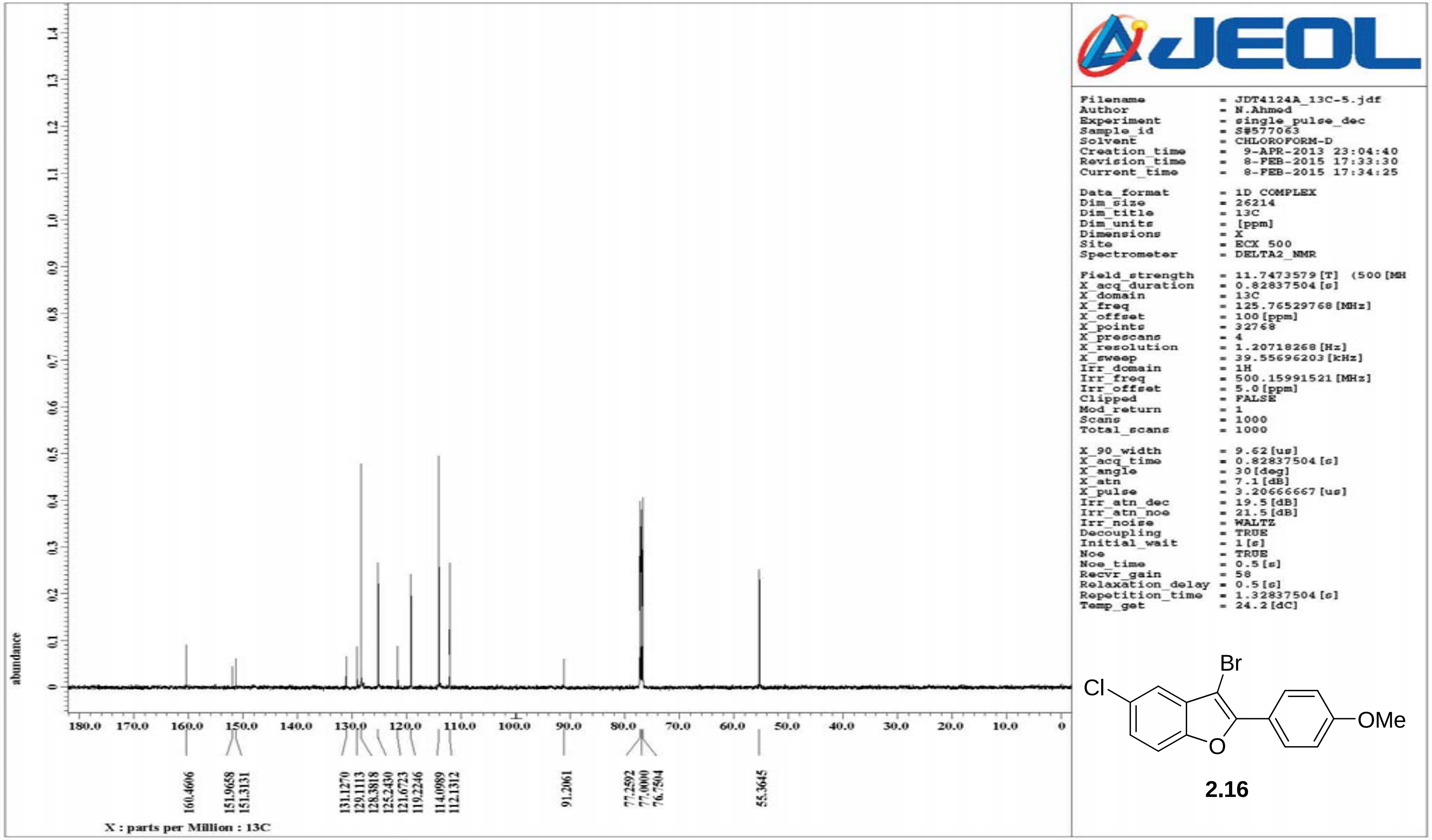

125 MHz ^{13}C NMR spectrum of compound **2.16**

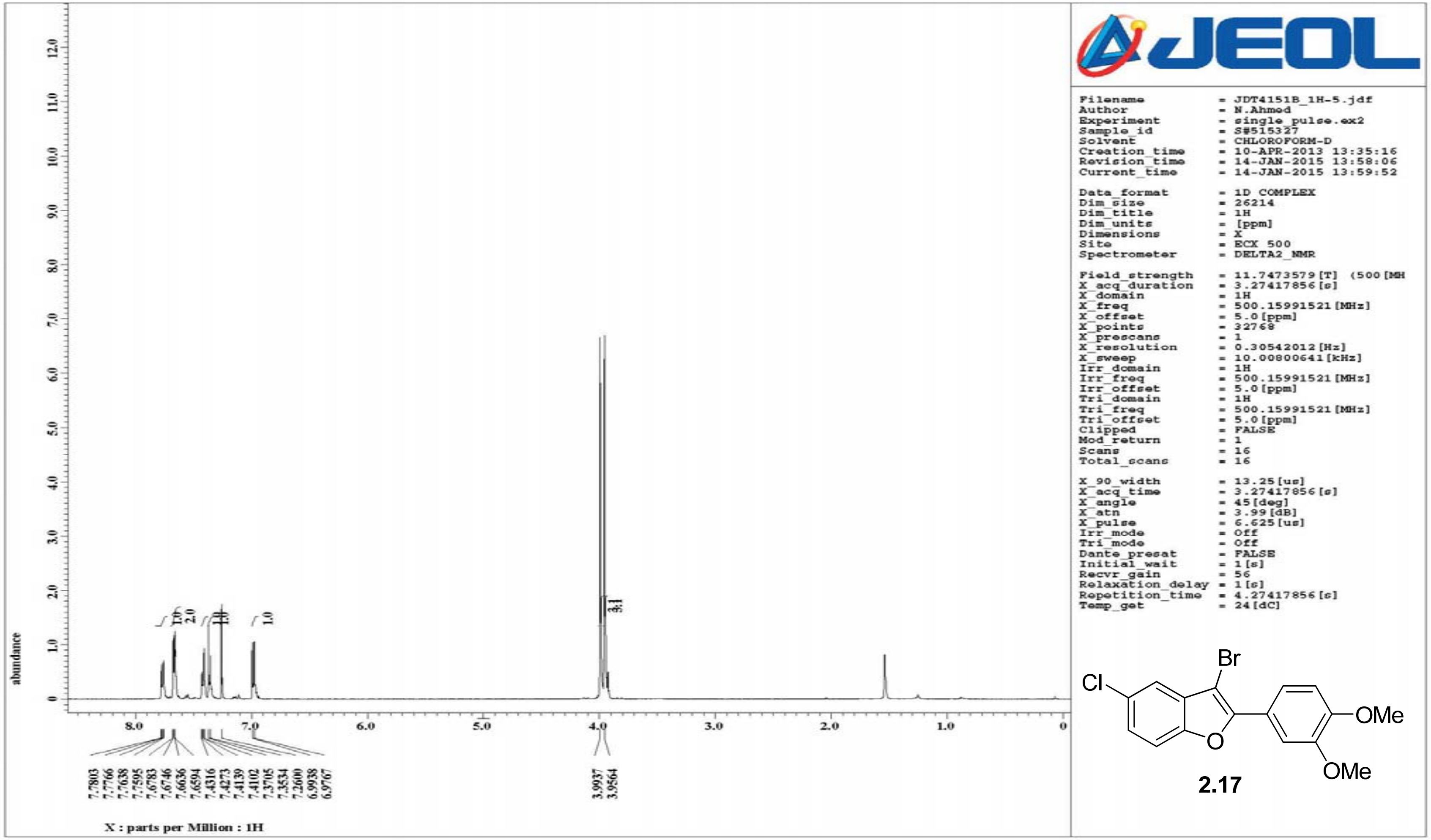

500 MHz ^{1}H NMR spectrum of compound **2.17**

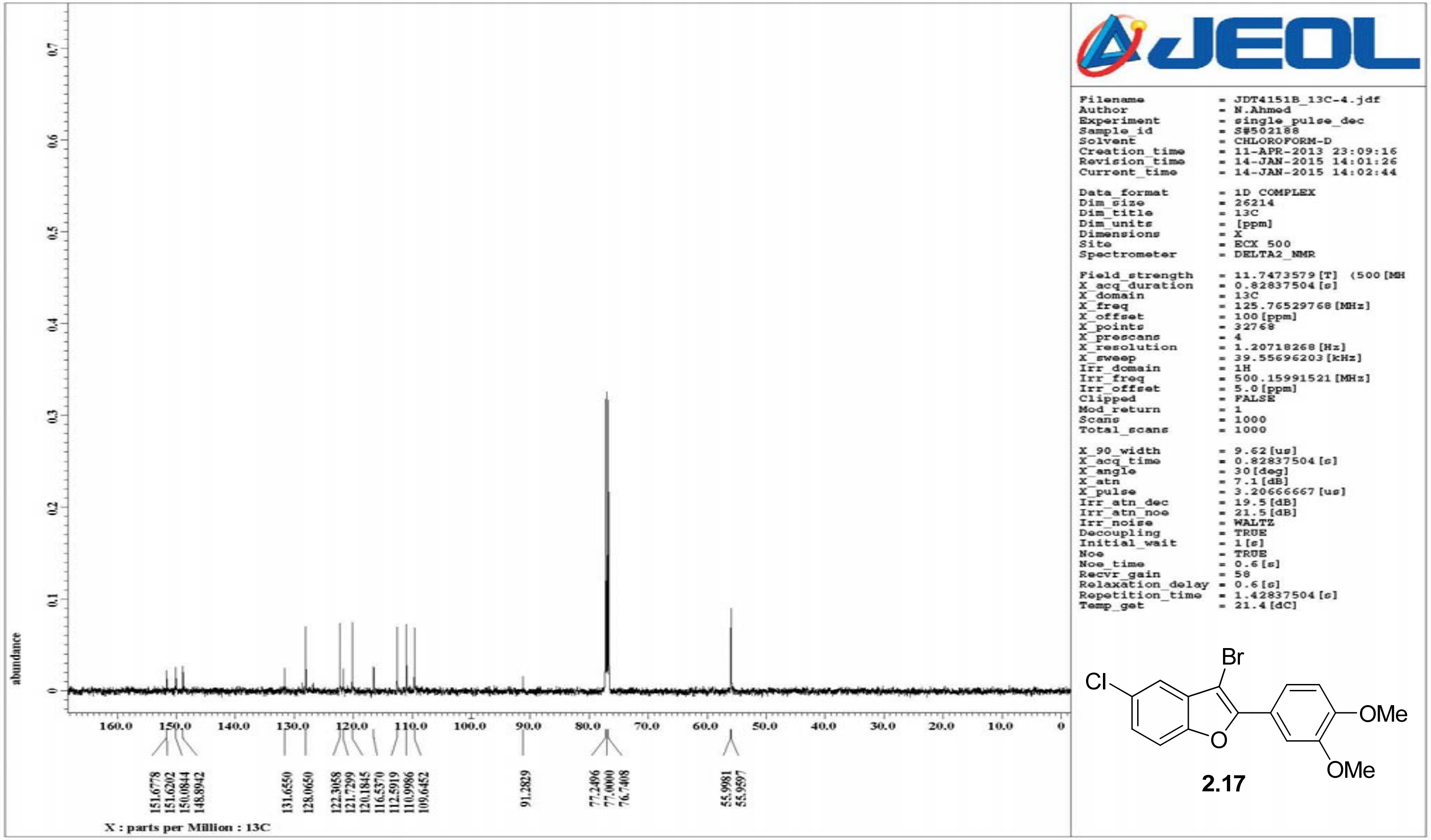

125 MHz ^{13}C NMR spectrum of compound **2.17**

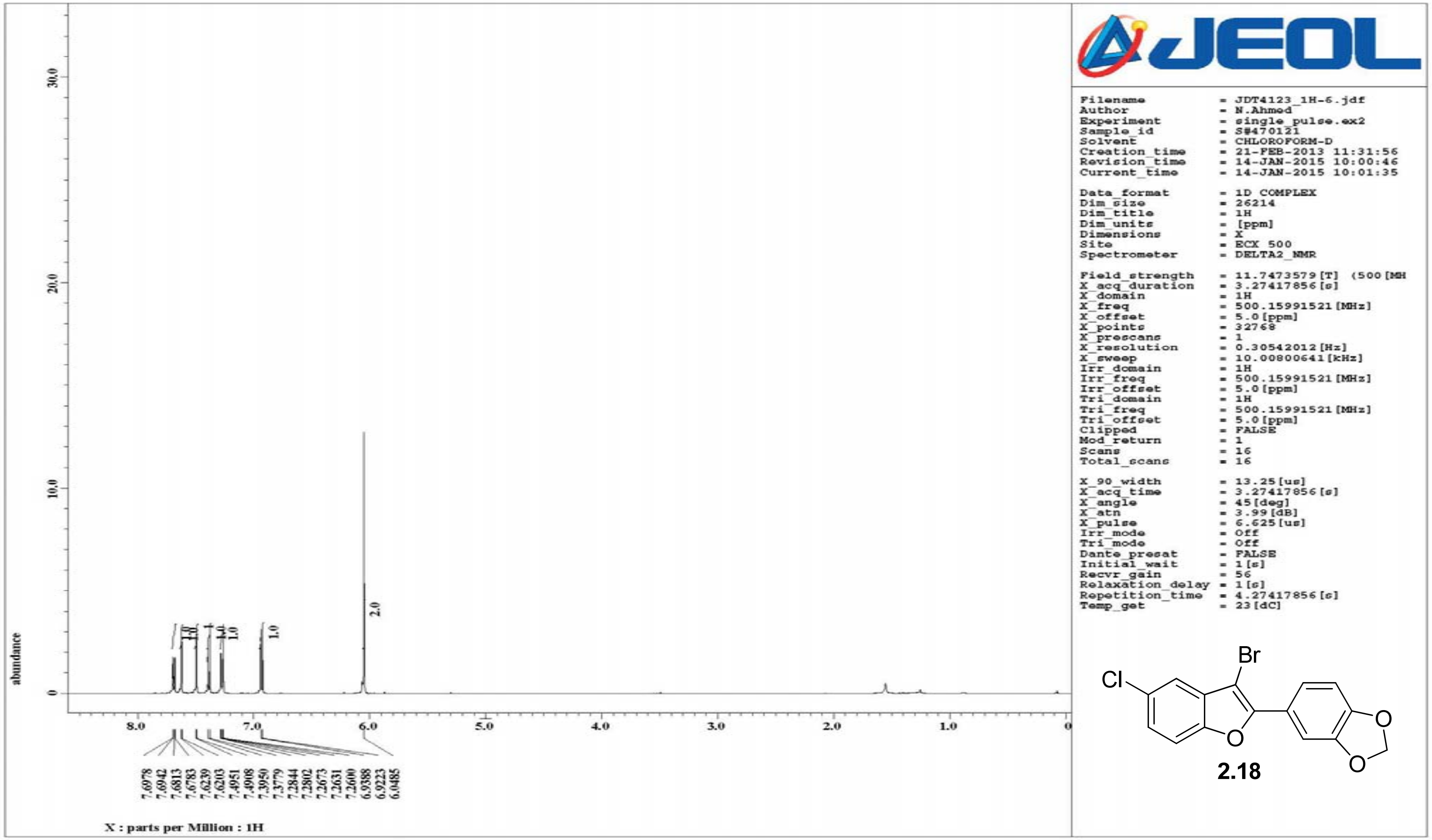

500 MHz ^{1}H NMR spectrum of compound **2.18**

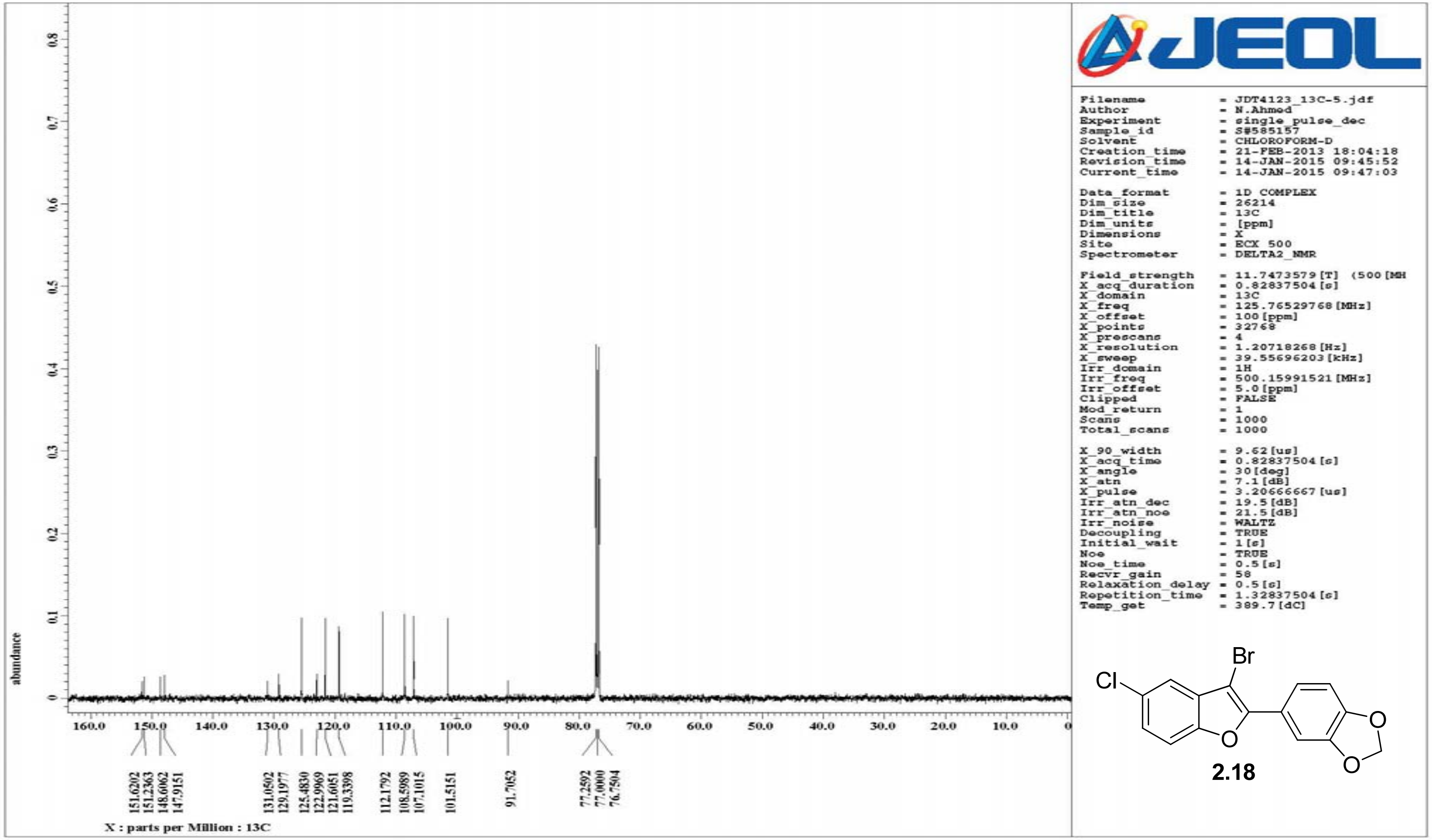

125 MHz ^{13}C NMR spectrum of compound **2.18**

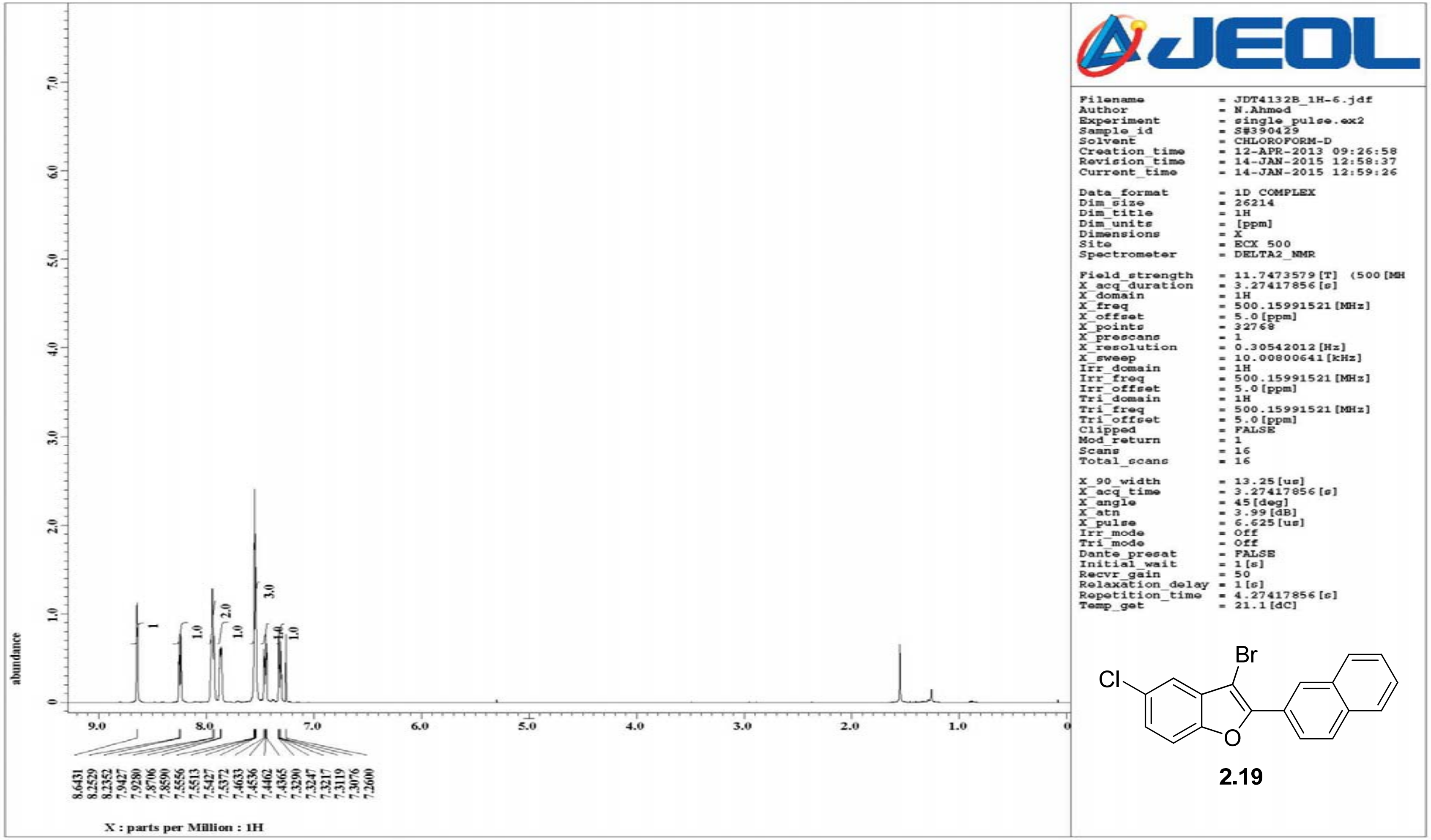

500 MHz ^{1}H NMR spectrum of compound **2.19**

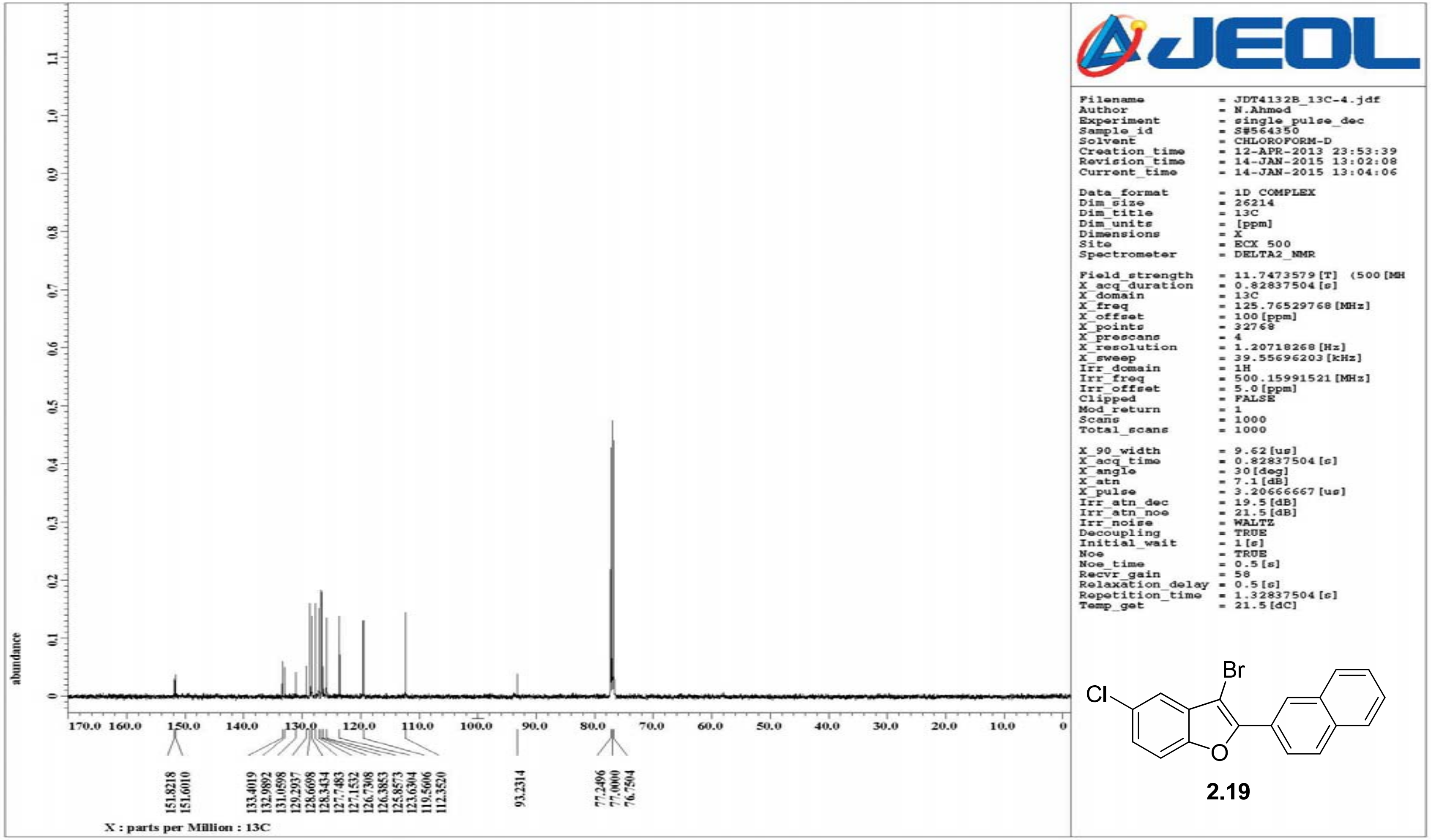

125 MHz ^{13}C NMR spectrum of compound **2.19**

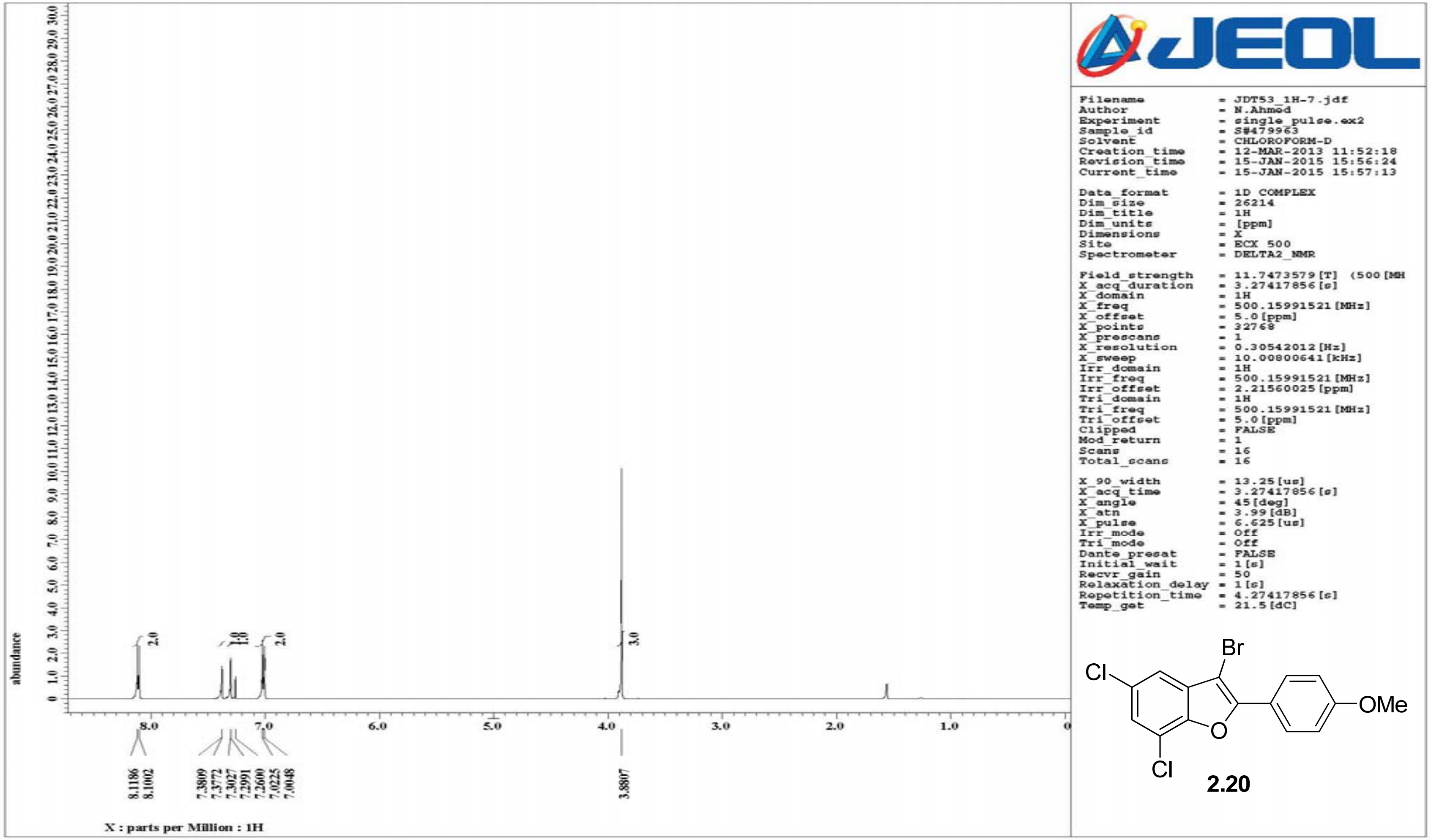

500 MHz ^{1}H NMR spectrum of compound **2.20**

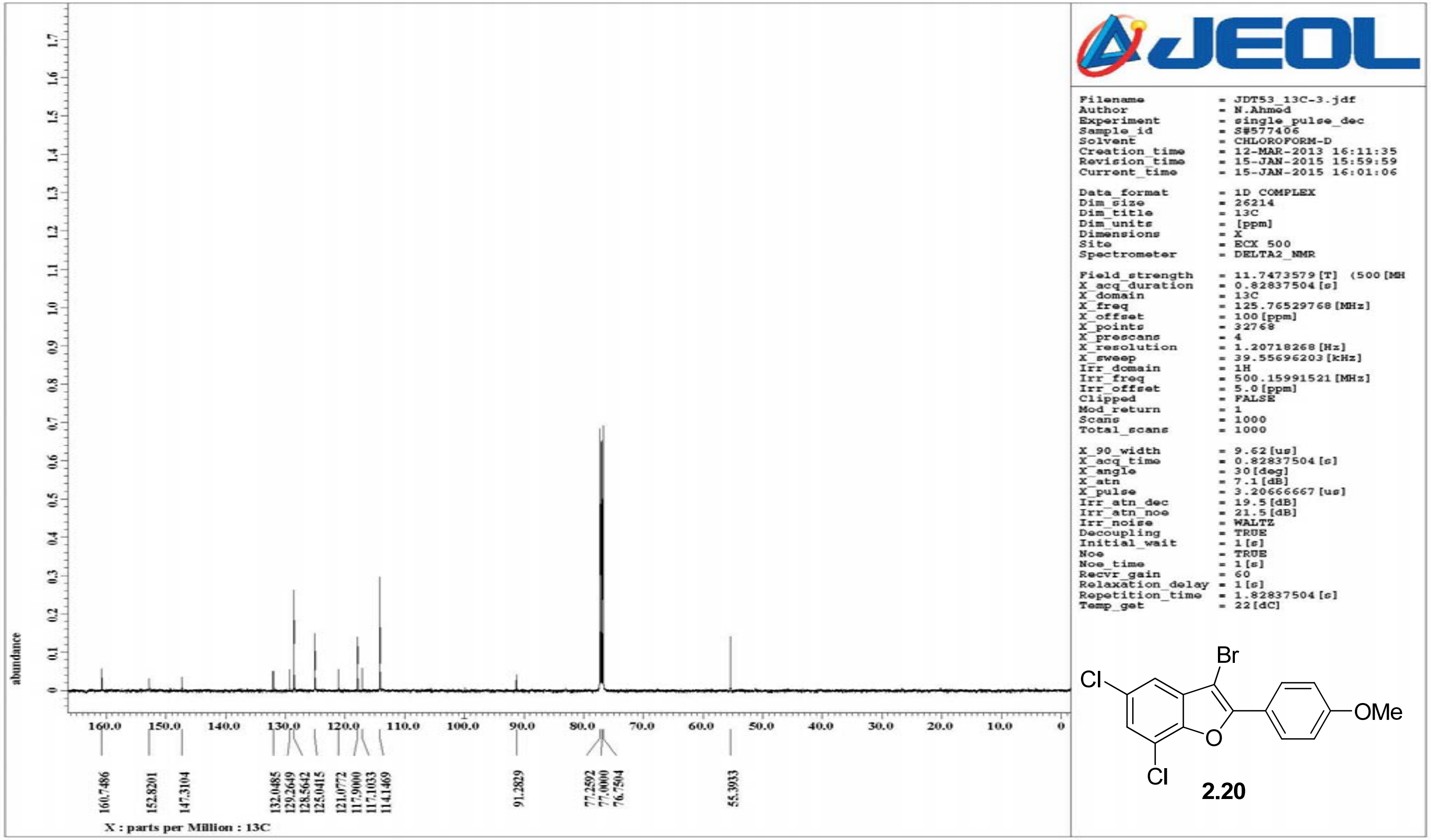

125 MHz ^{13}C NMR spectrum of compound **2.20**

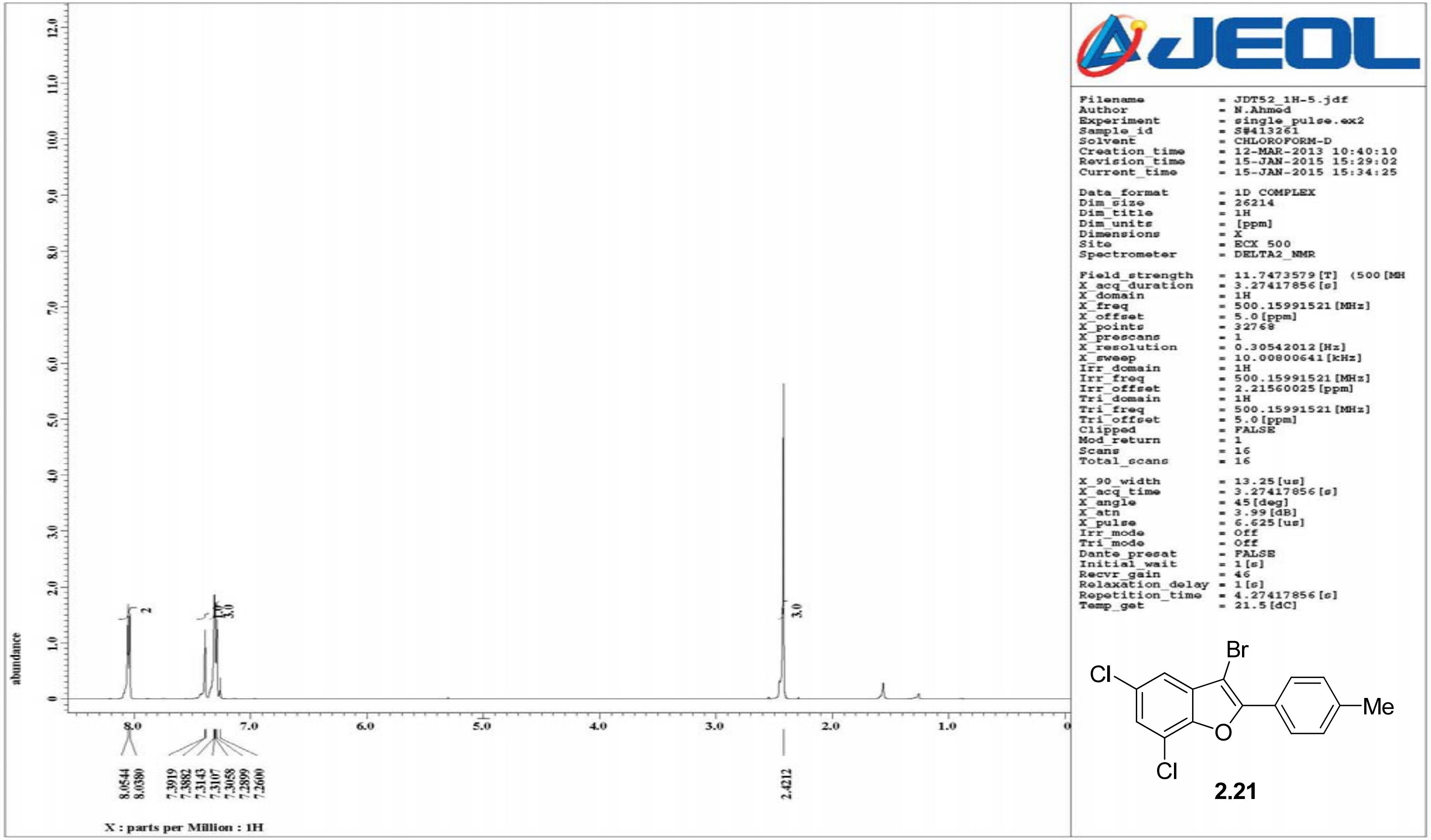

500 MHz ^{1}H NMR spectrum of compound **2.21**

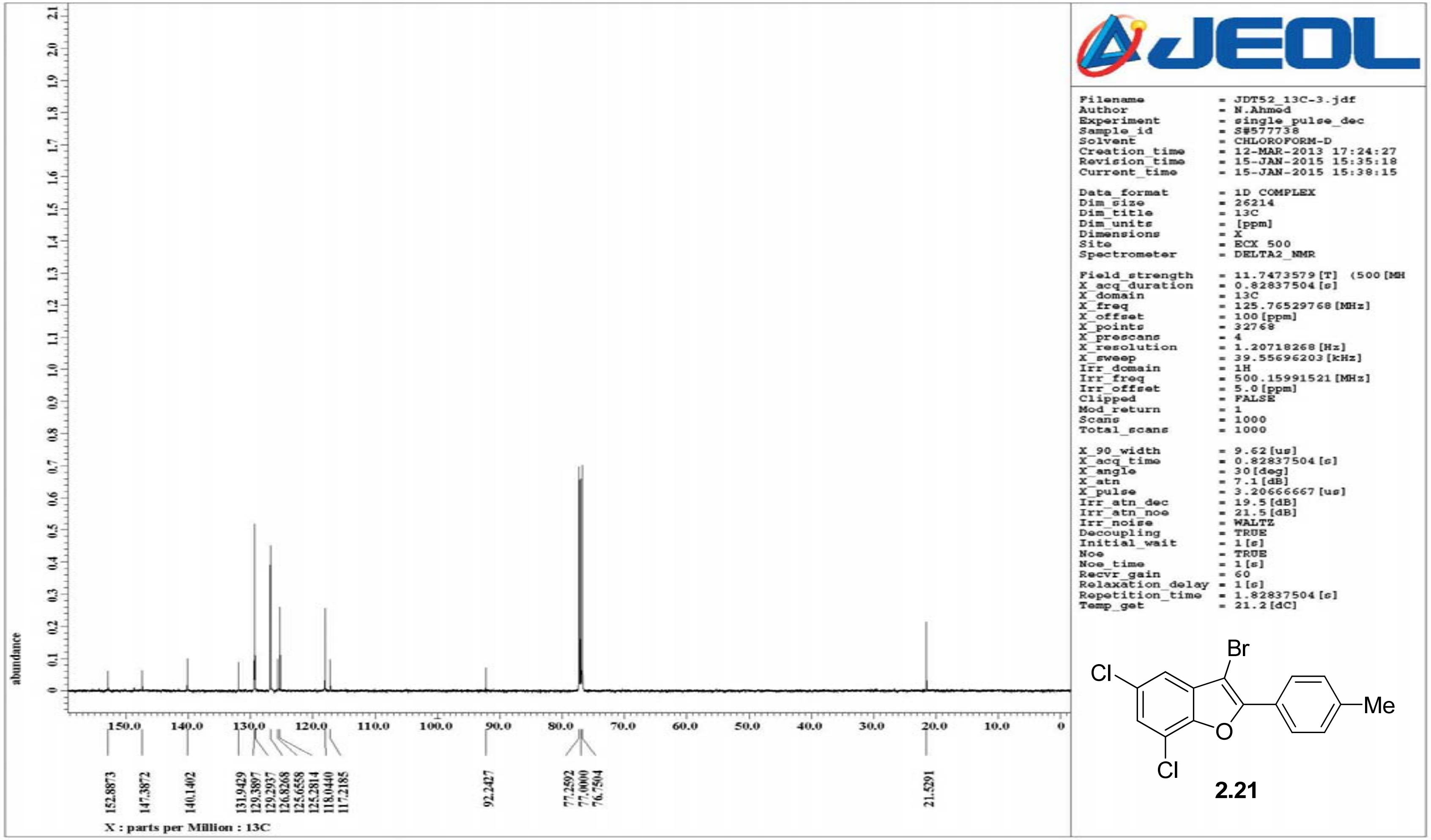

125 MHz ^{13}C NMR spectrum of compound **2.21**

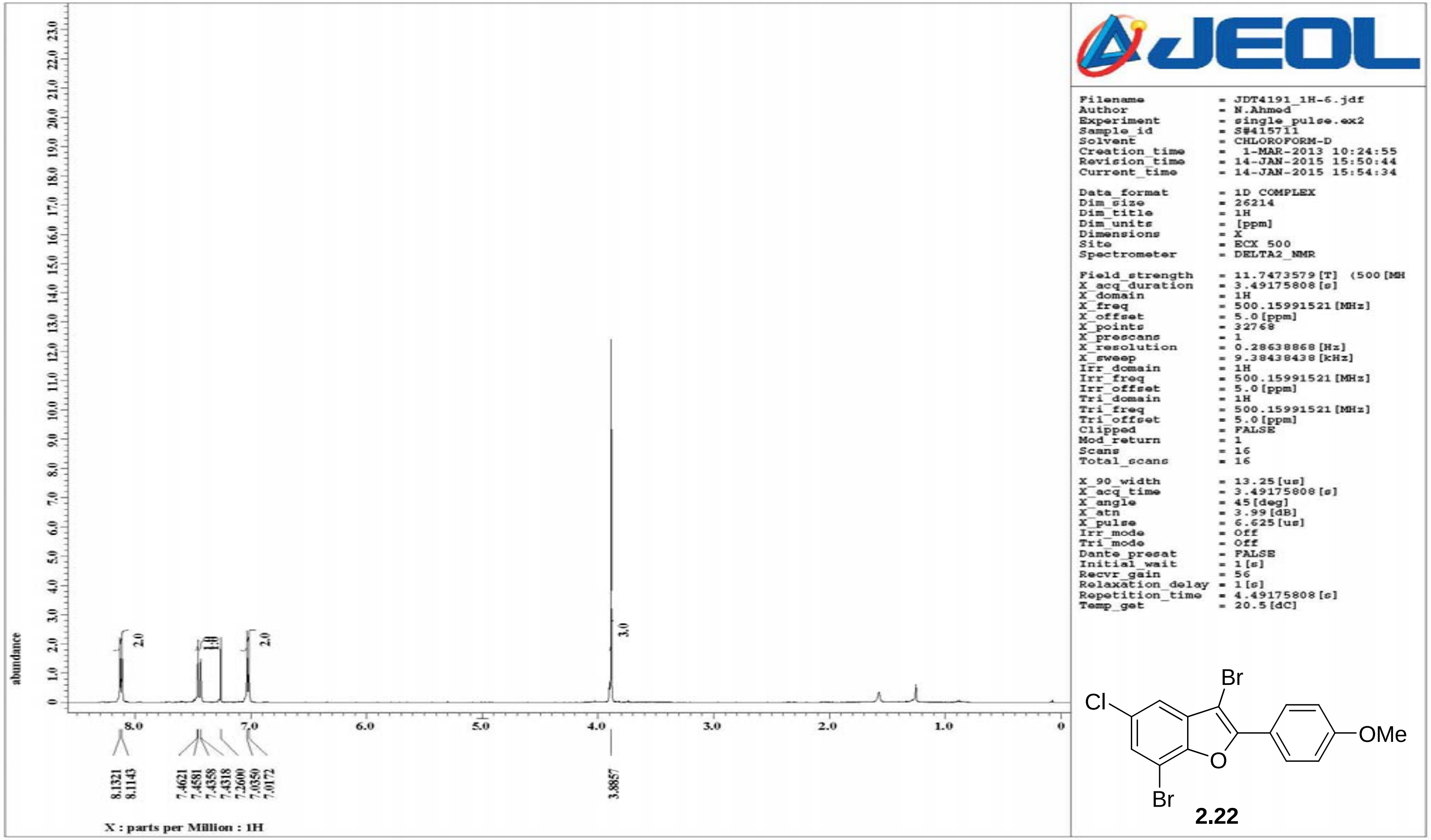

500 MHz ^{1}H NMR spectrum of compound **2.22**

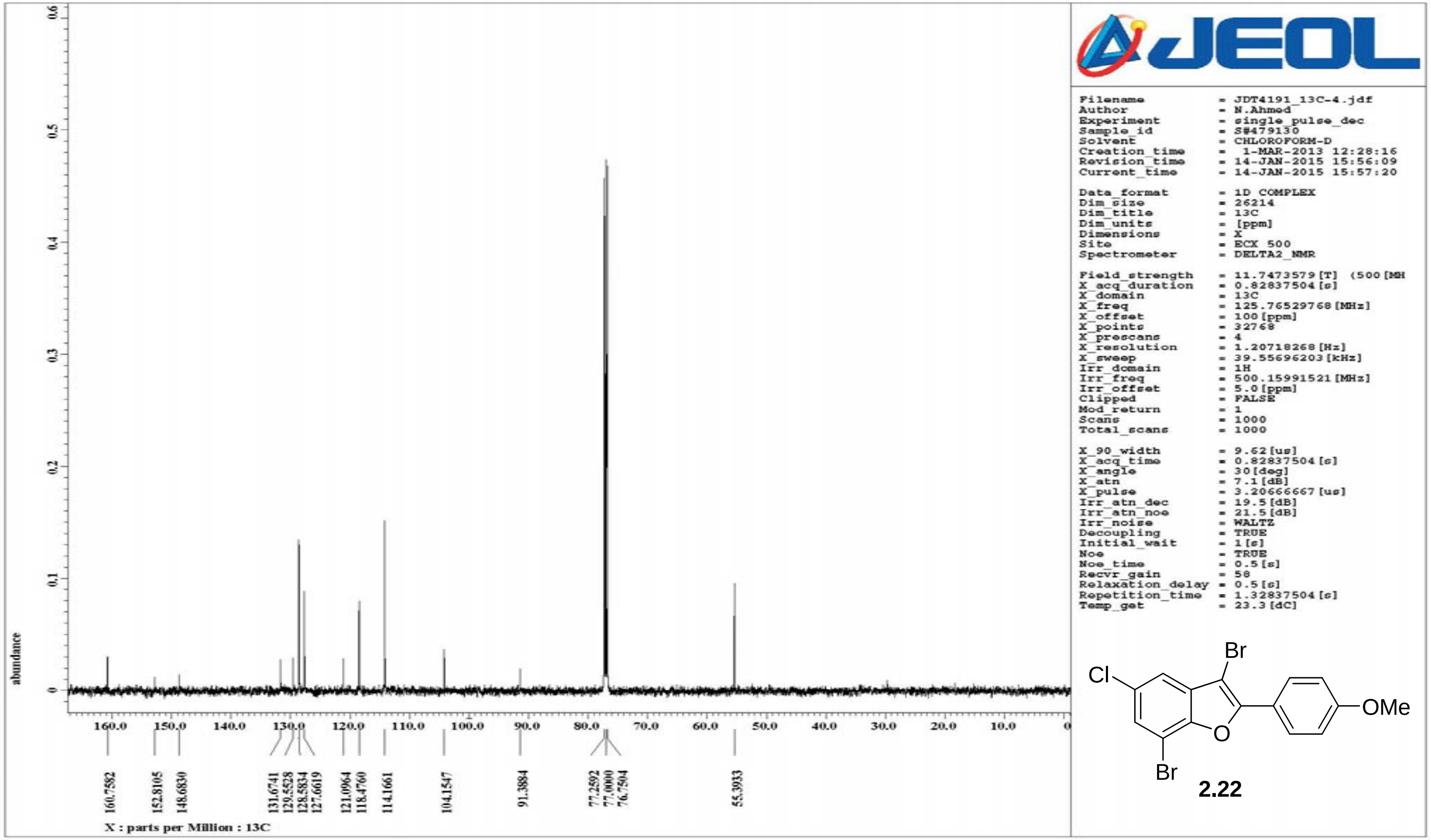

125 MHz ^{13}C NMR spectrum of compound **2.22**

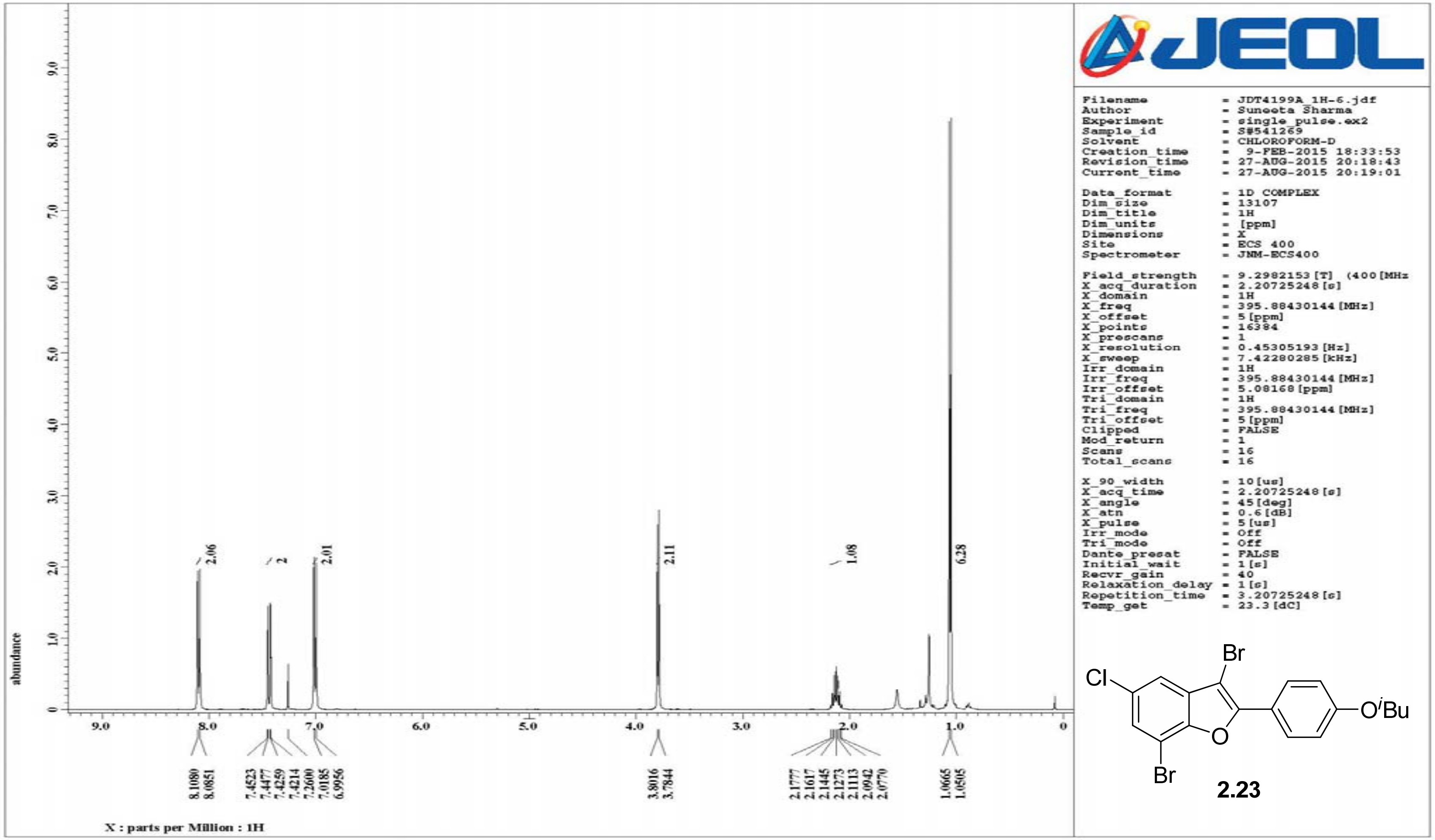

400 MHz ^{1}H NMR spectrum of compound **2.23**

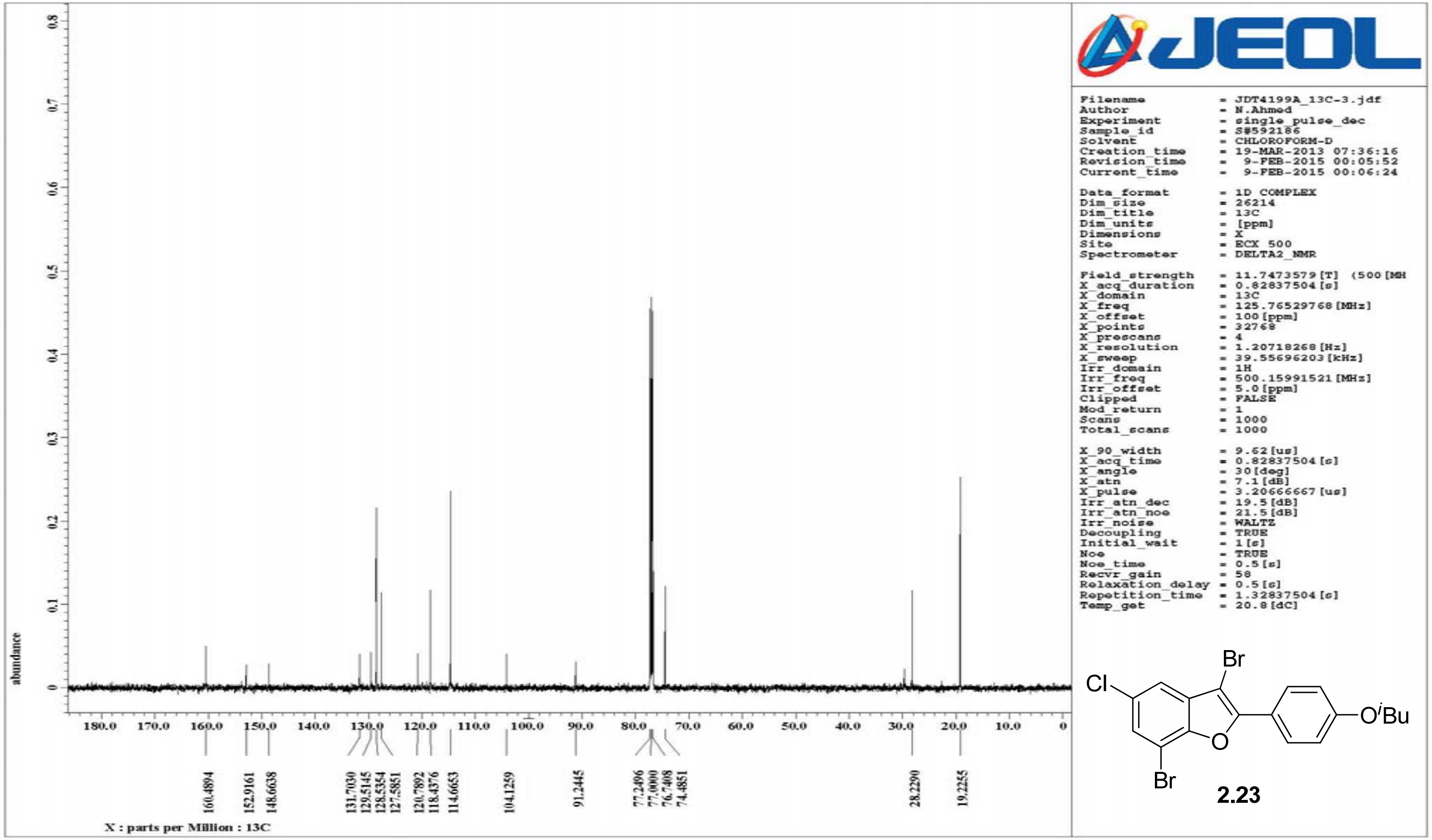

125 MHz ^{13}C NMR spectrum of compound **2.23**

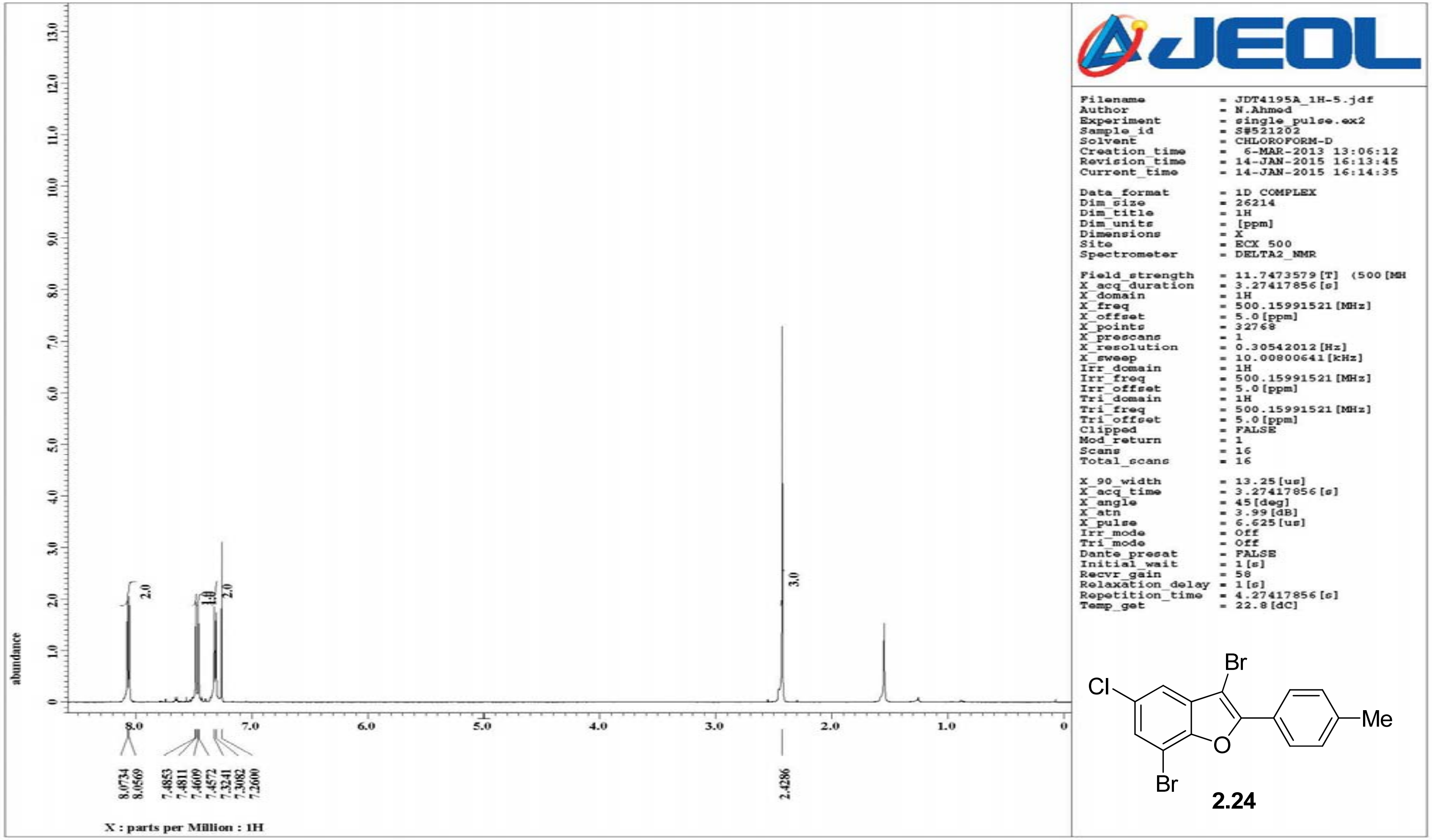

500 MHz ^{1}H NMR spectrum of compound **2.24**

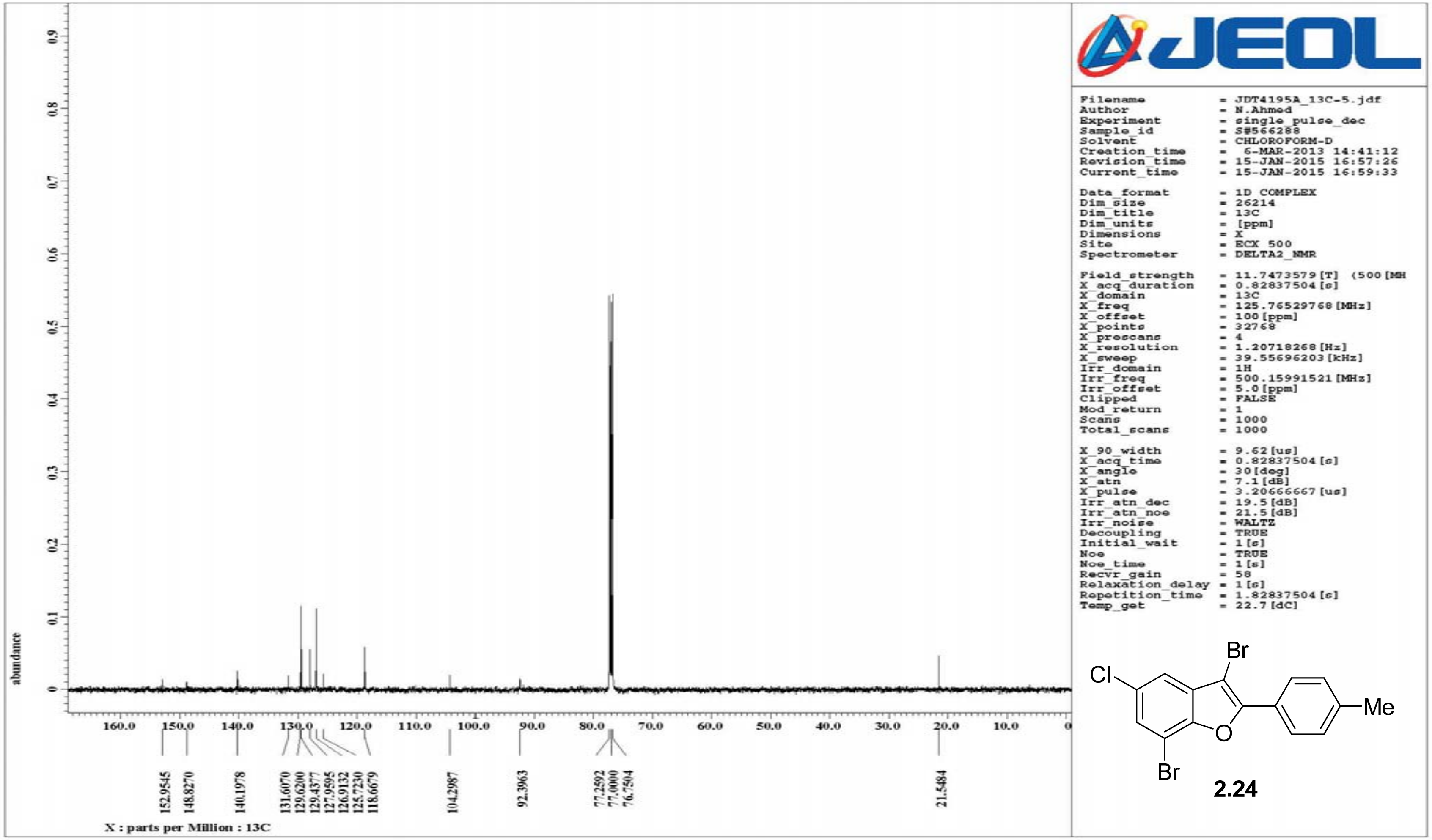

125 MHz ^{13}C NMR spectrum of compound **2.24**

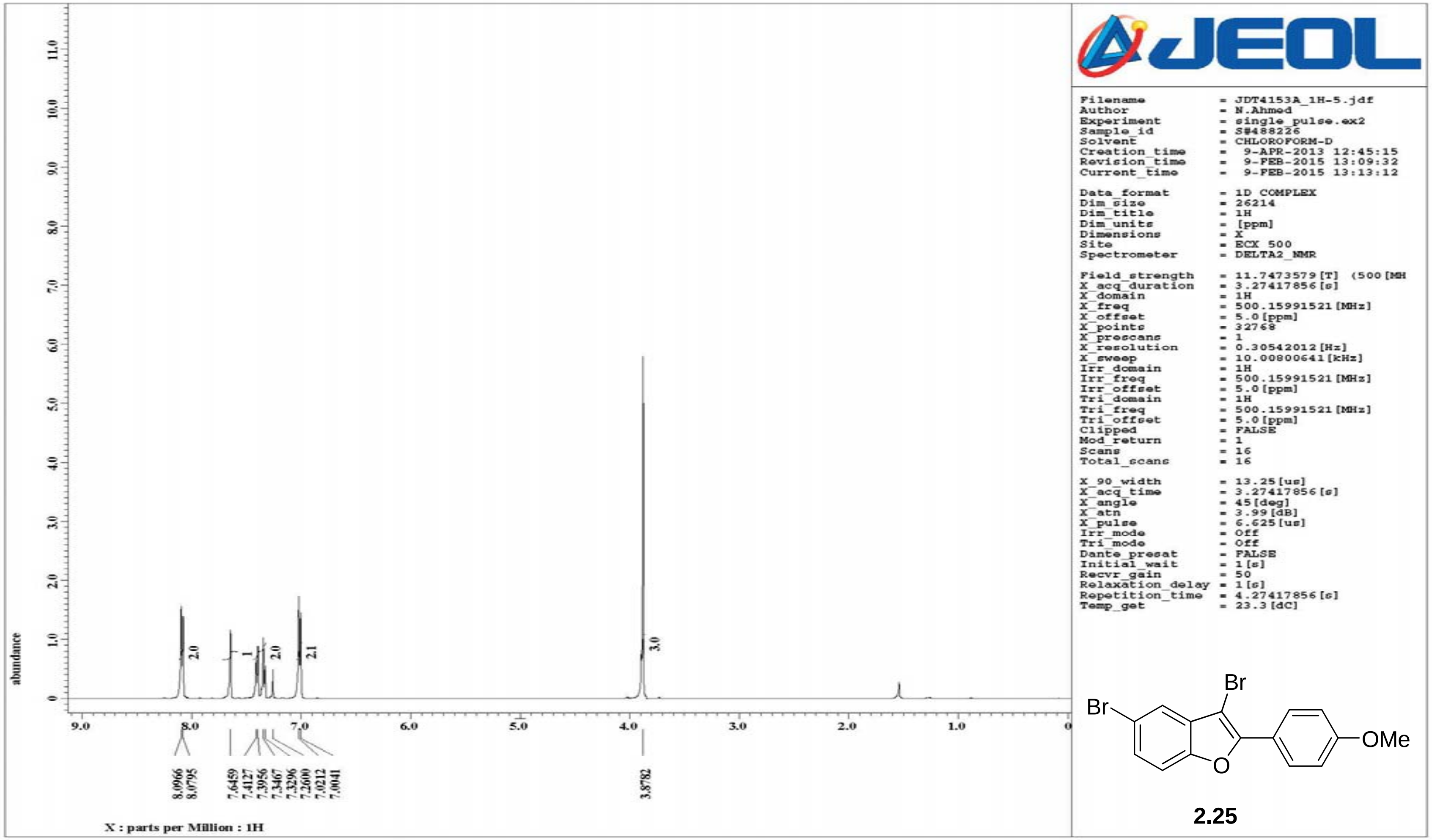

500 MHz ^{1}H NMR spectrum of compound **2.25**

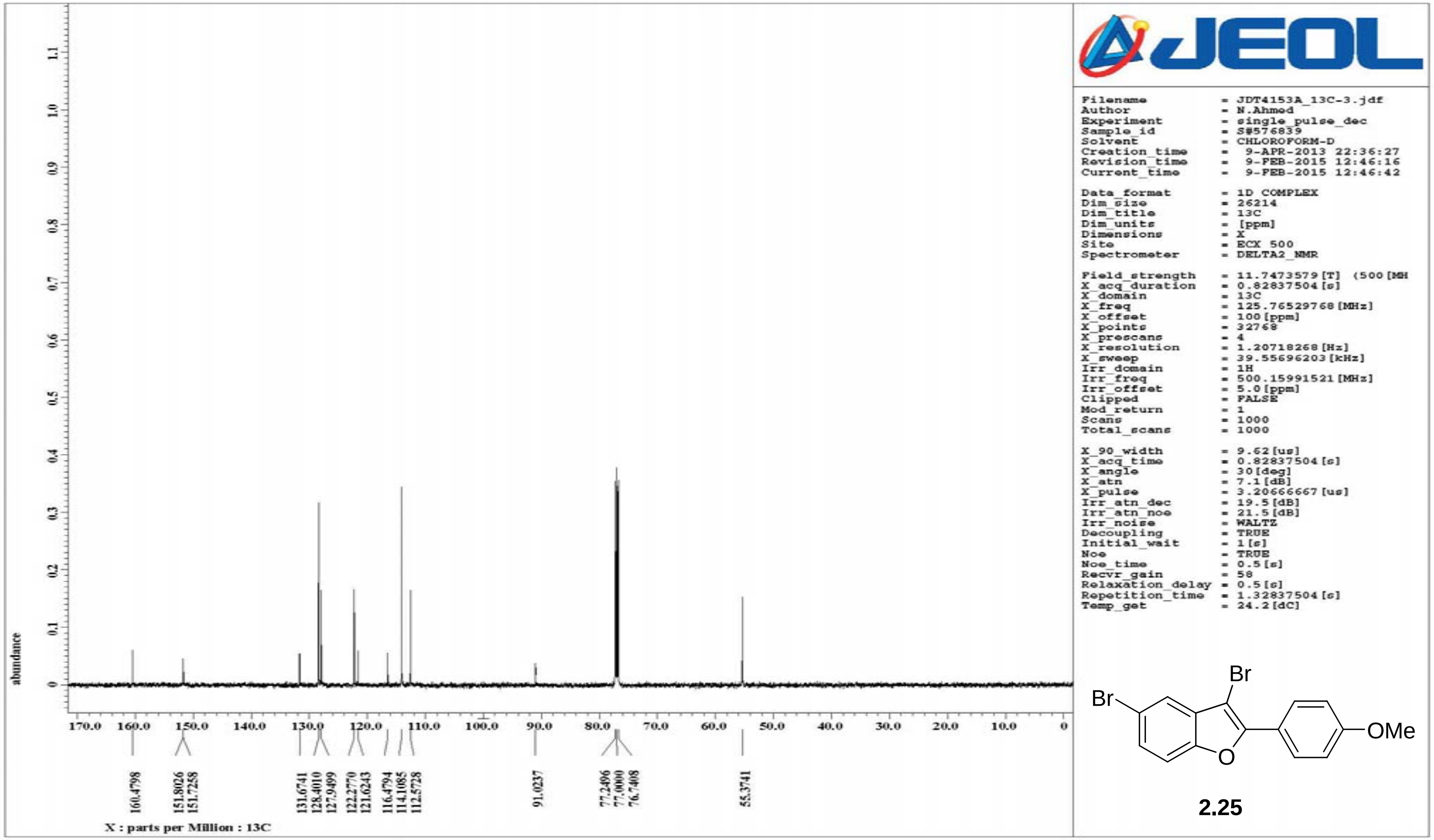

125 MHz ^{13}C NMR spectrum of compound **2.25**

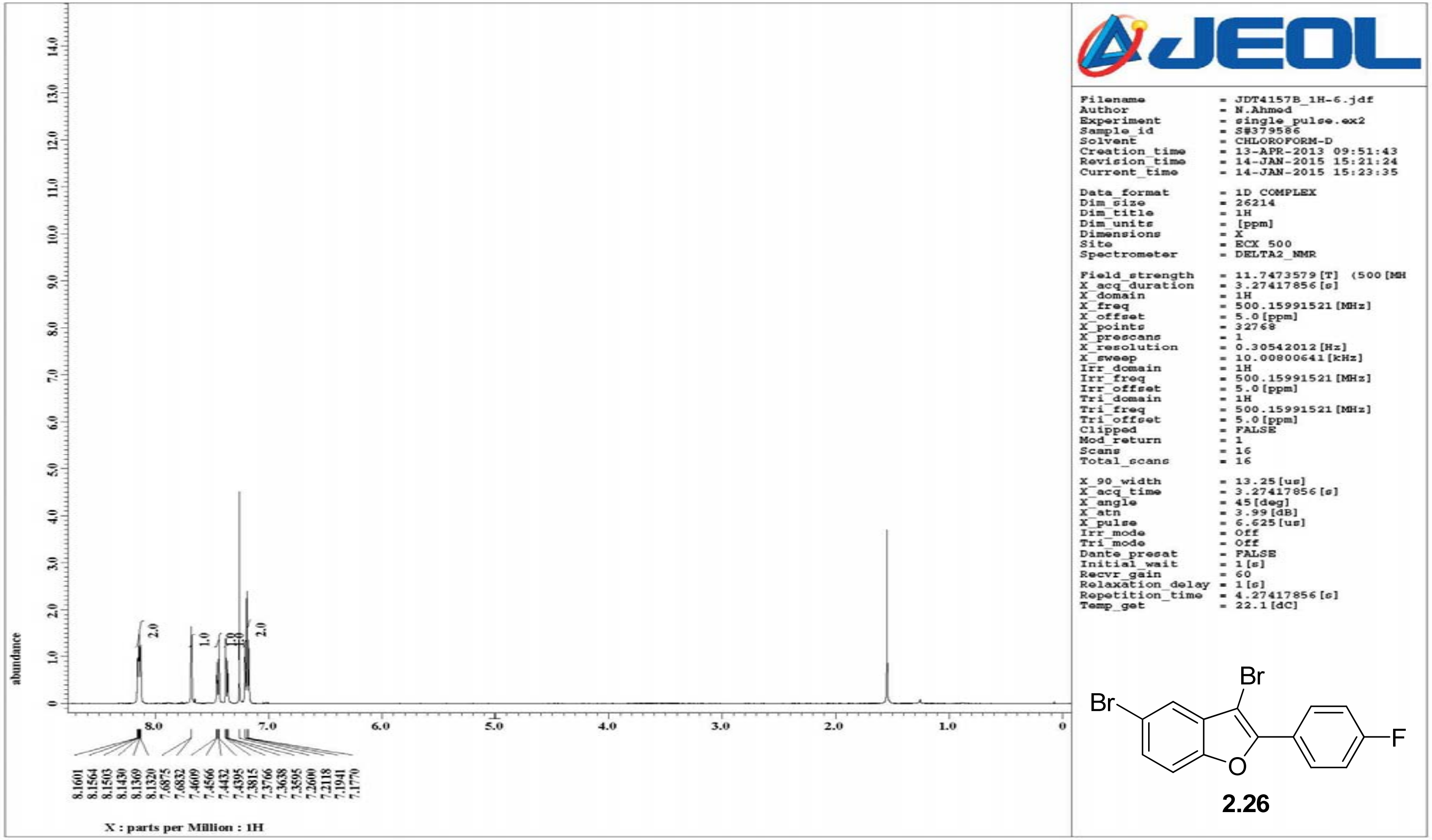

500 MHz ^{1}H NMR spectrum of compound **2.26**

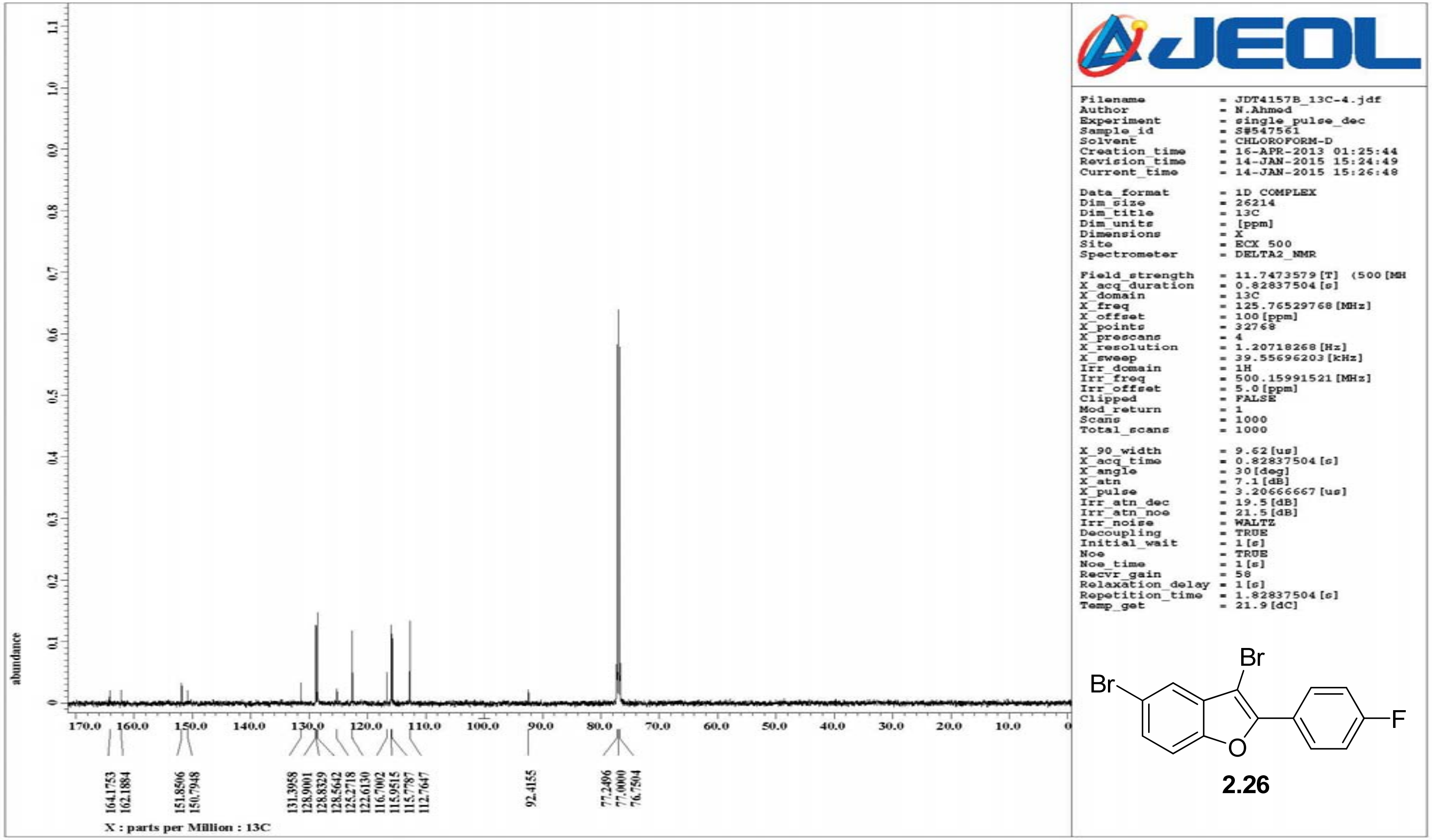

125 MHz ^{13}C NMR spectrum of compound **2.26**

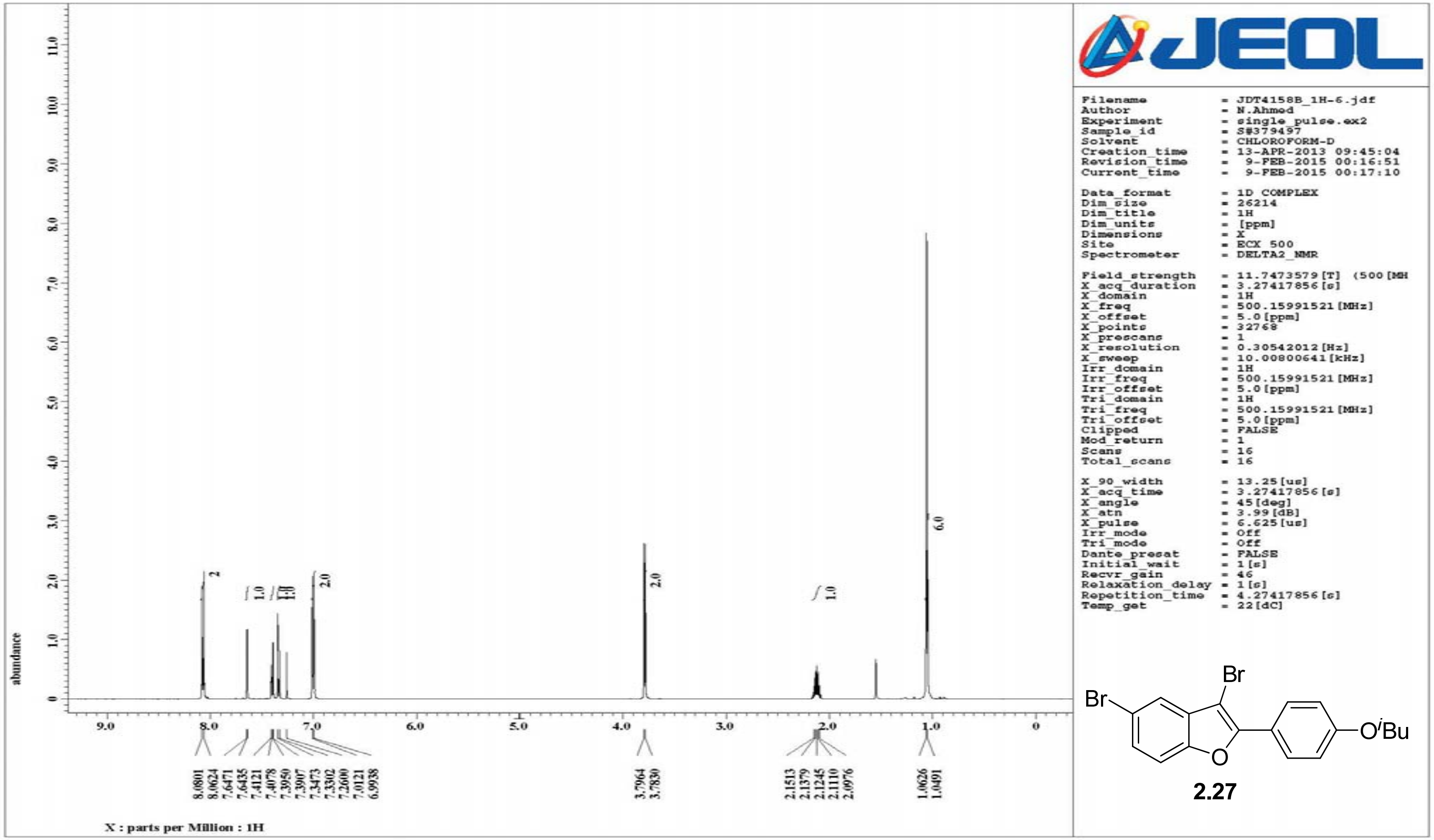

500 MHz ^{1}H NMR spectrum of compound **2.27**

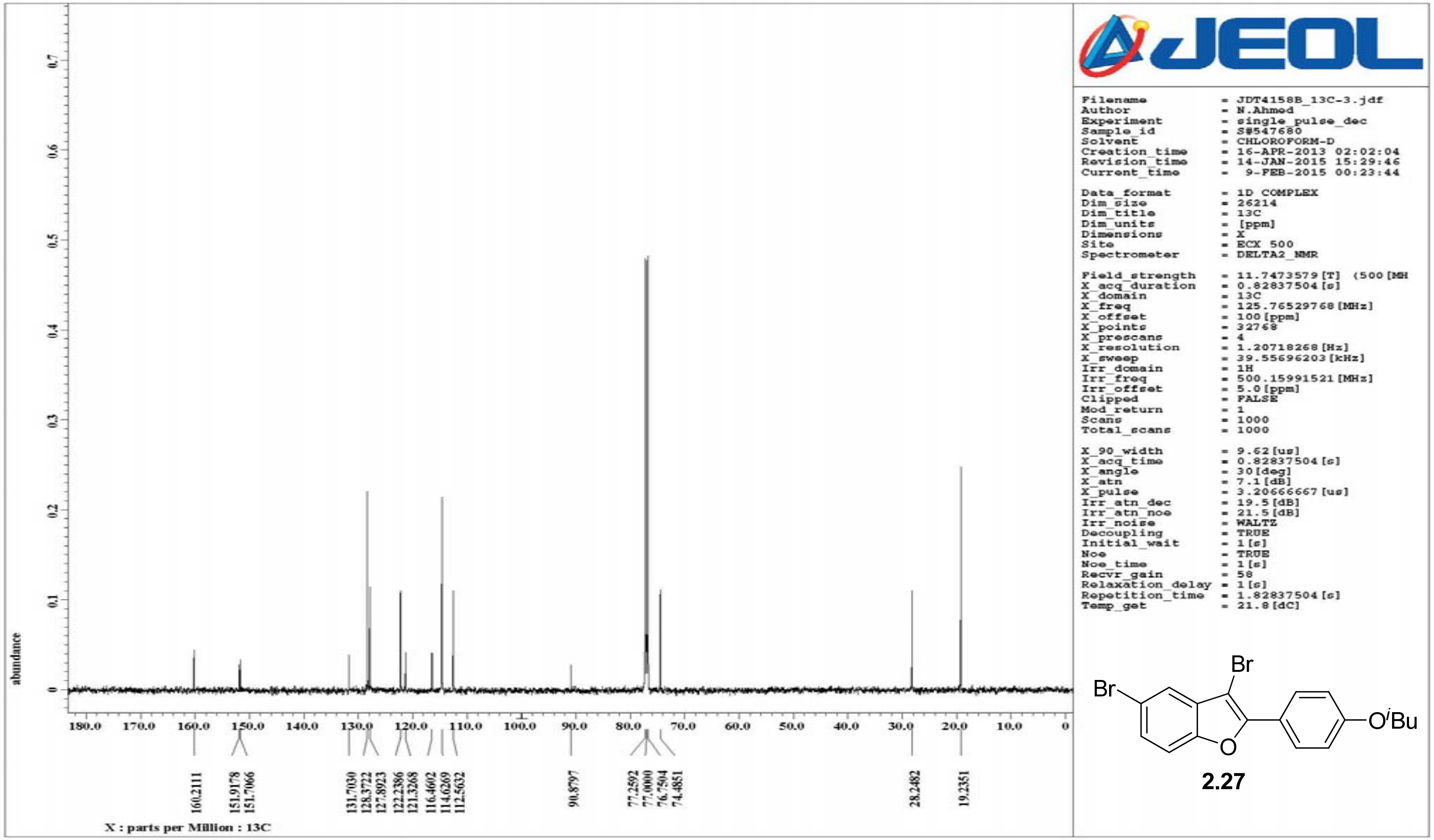

125 MHz ^{13}C NMR spectrum of compound **2.27**

3. ^{1}H, ^{13}C Spectra of Compounds (**3.1-3.7**):

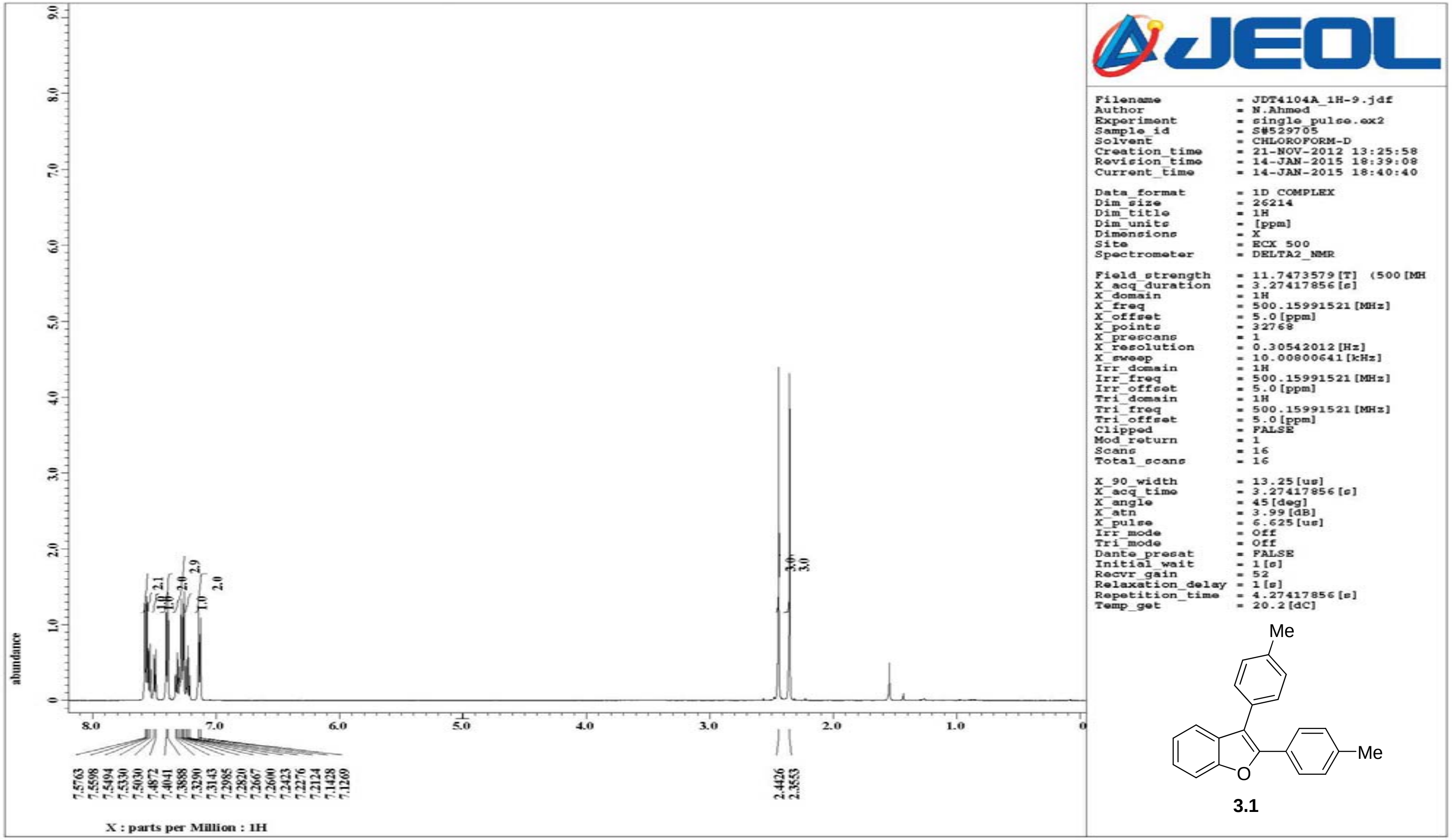

3.1

500 MHz ^{1}H NMR spectrum of compound **3.1**

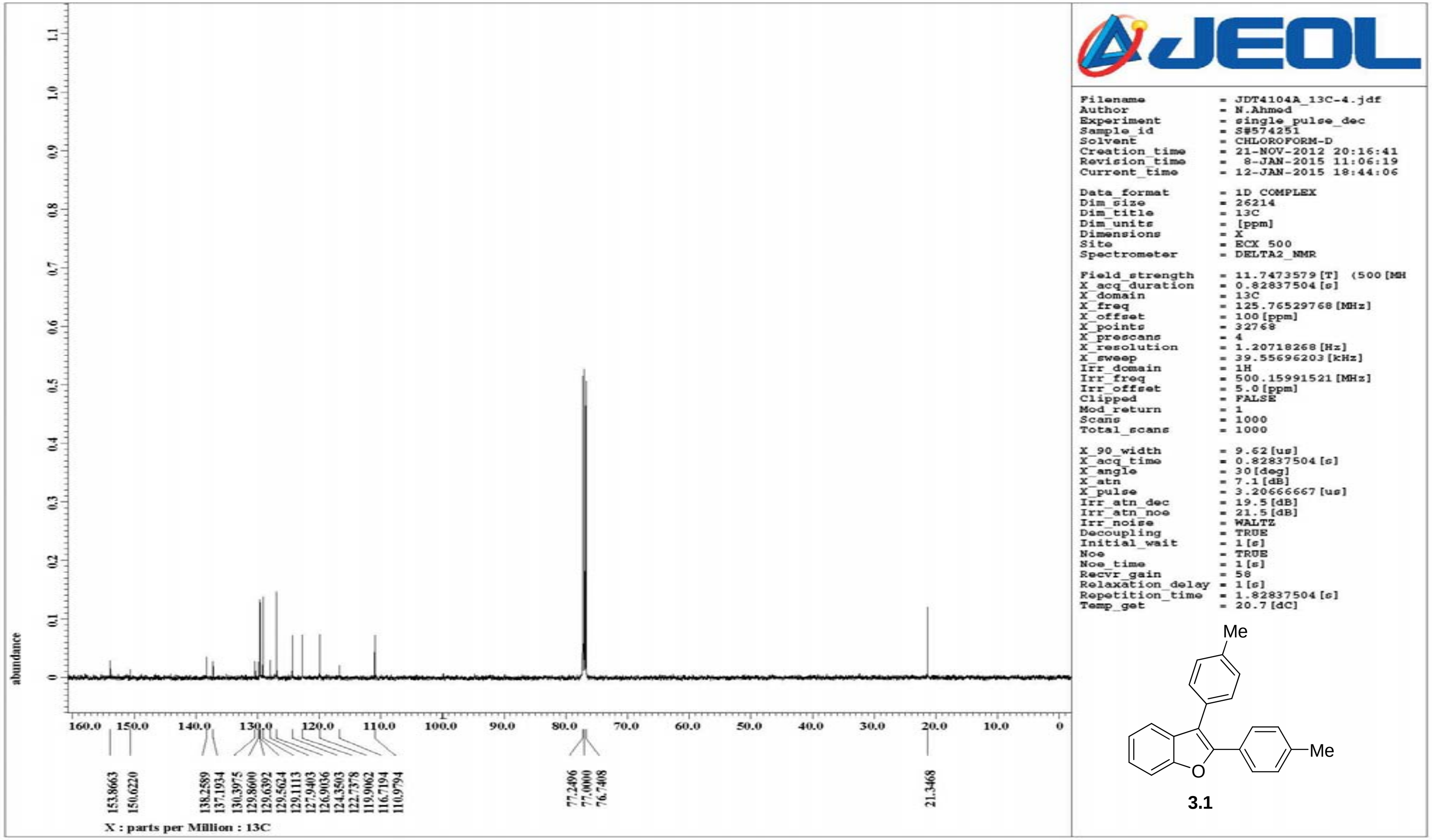

125 MHz ^{13}C NMR spectrum of compound **3.1**

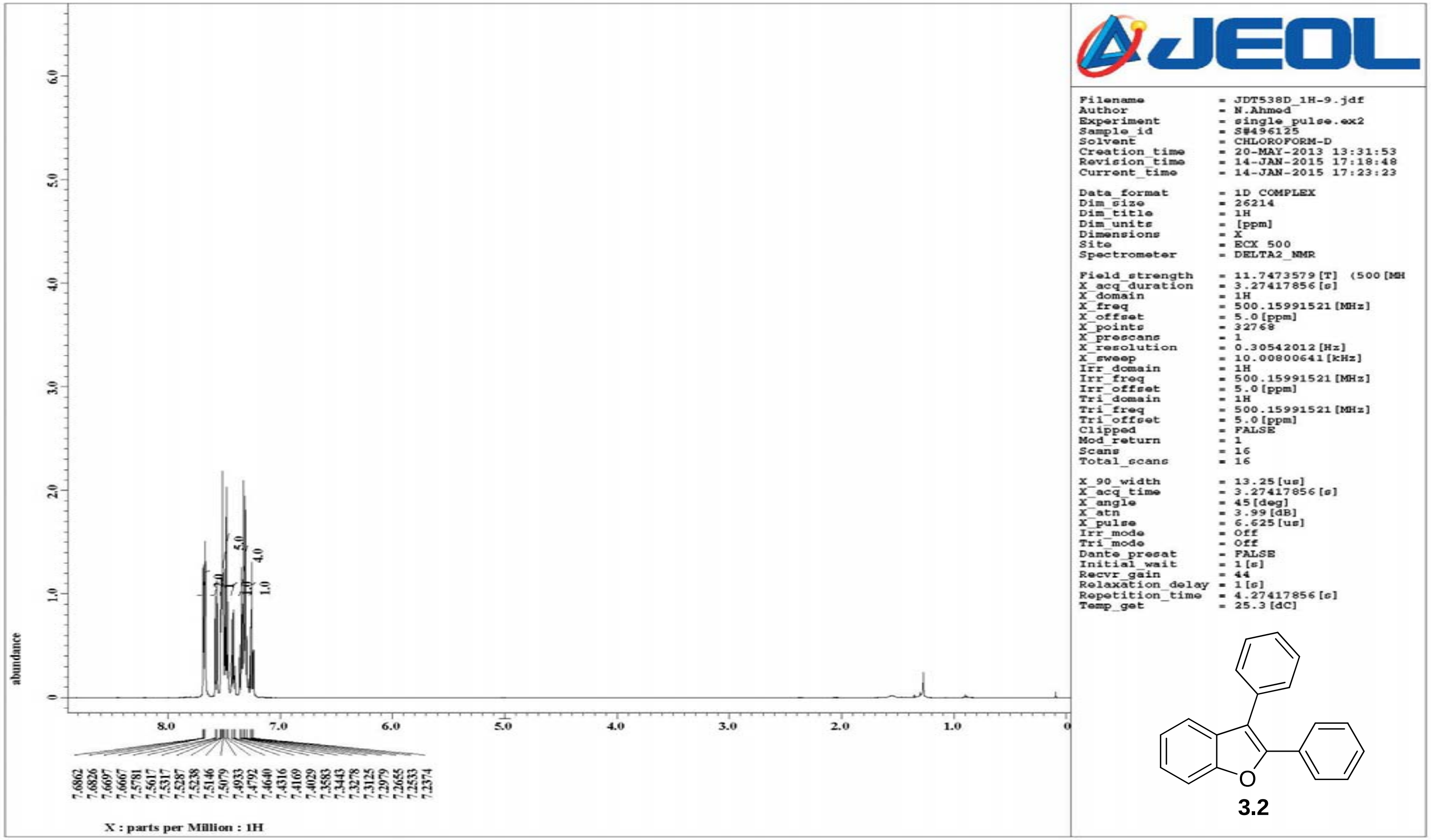

500 MHz ^{1}H NMR spectrum of compound **3.2**

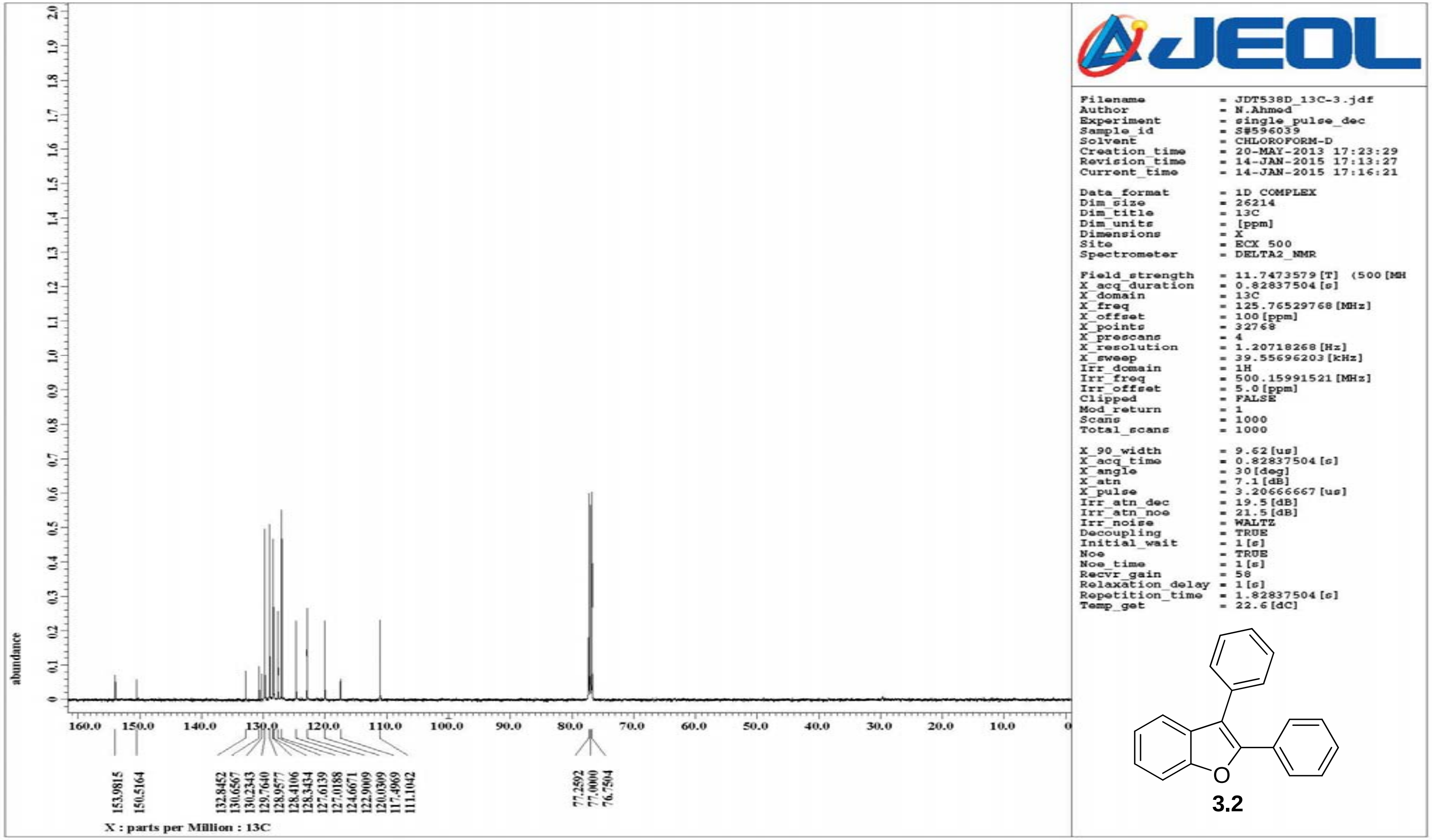

125 MHz ^{13}C NMR spectrum of compound **3.2**

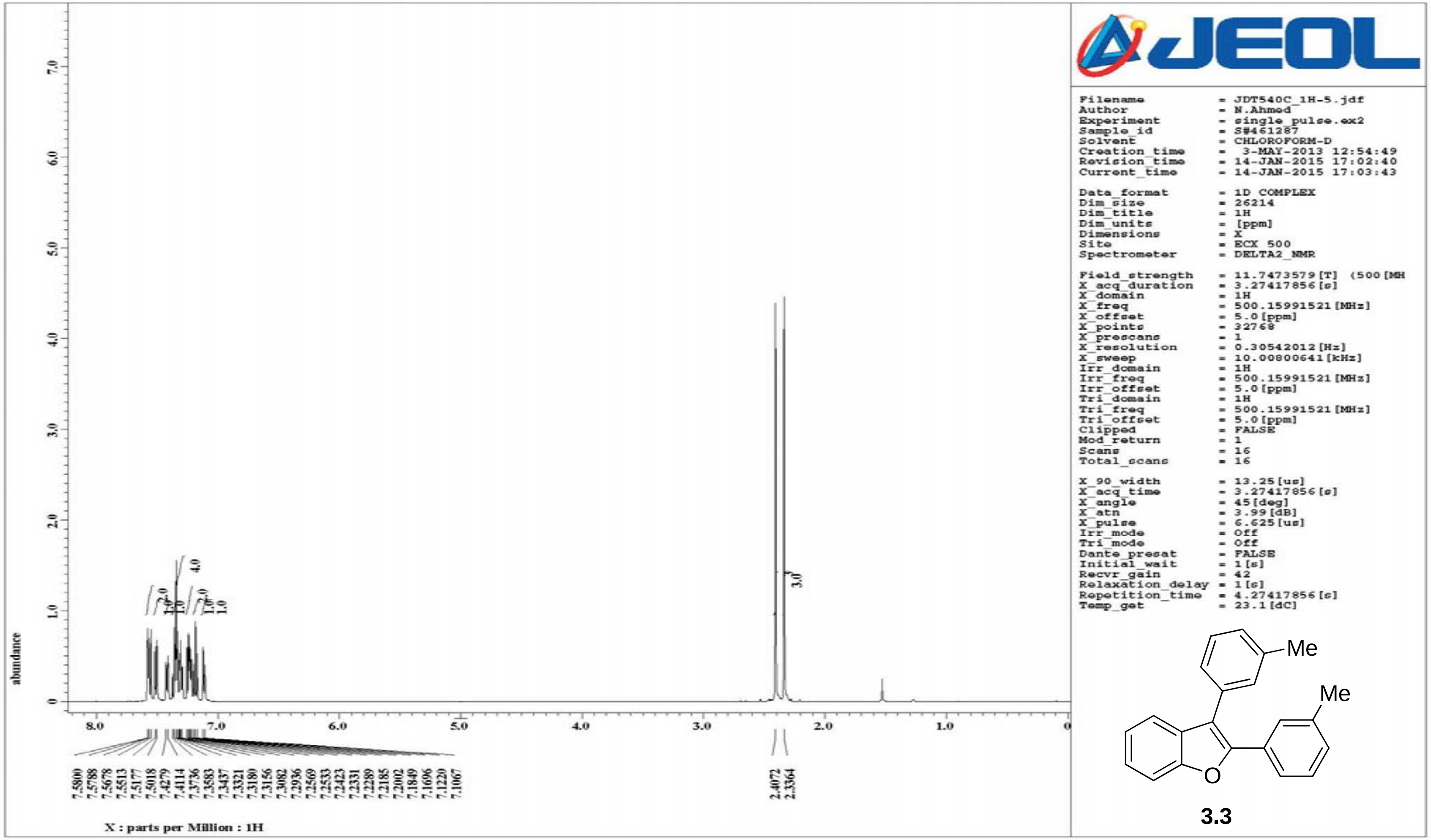

500 MHz ^{1}H NMR spectrum of compound **3.3**

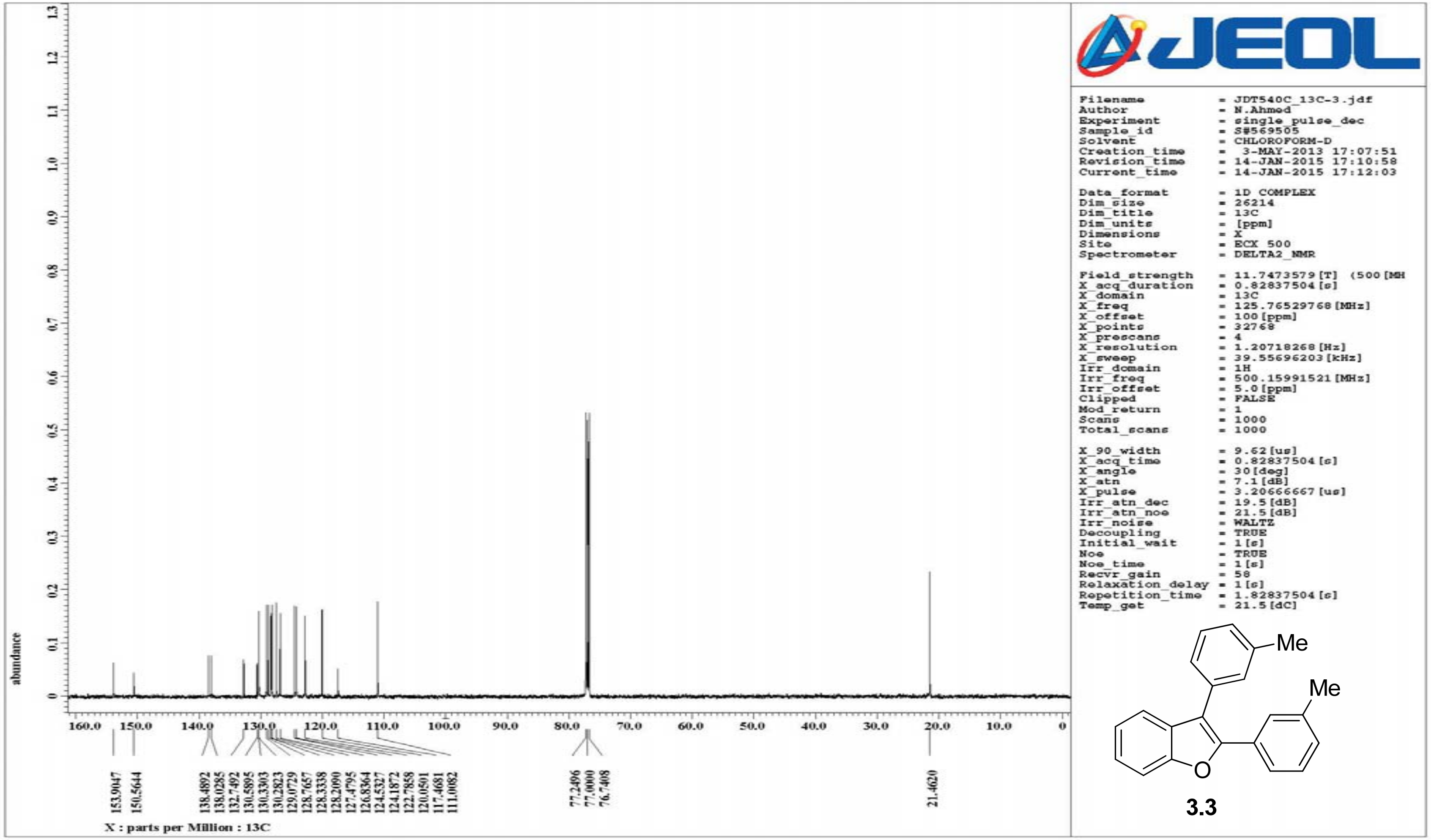

125 MHz ^{13}C NMR spectrum of compound **3.3**

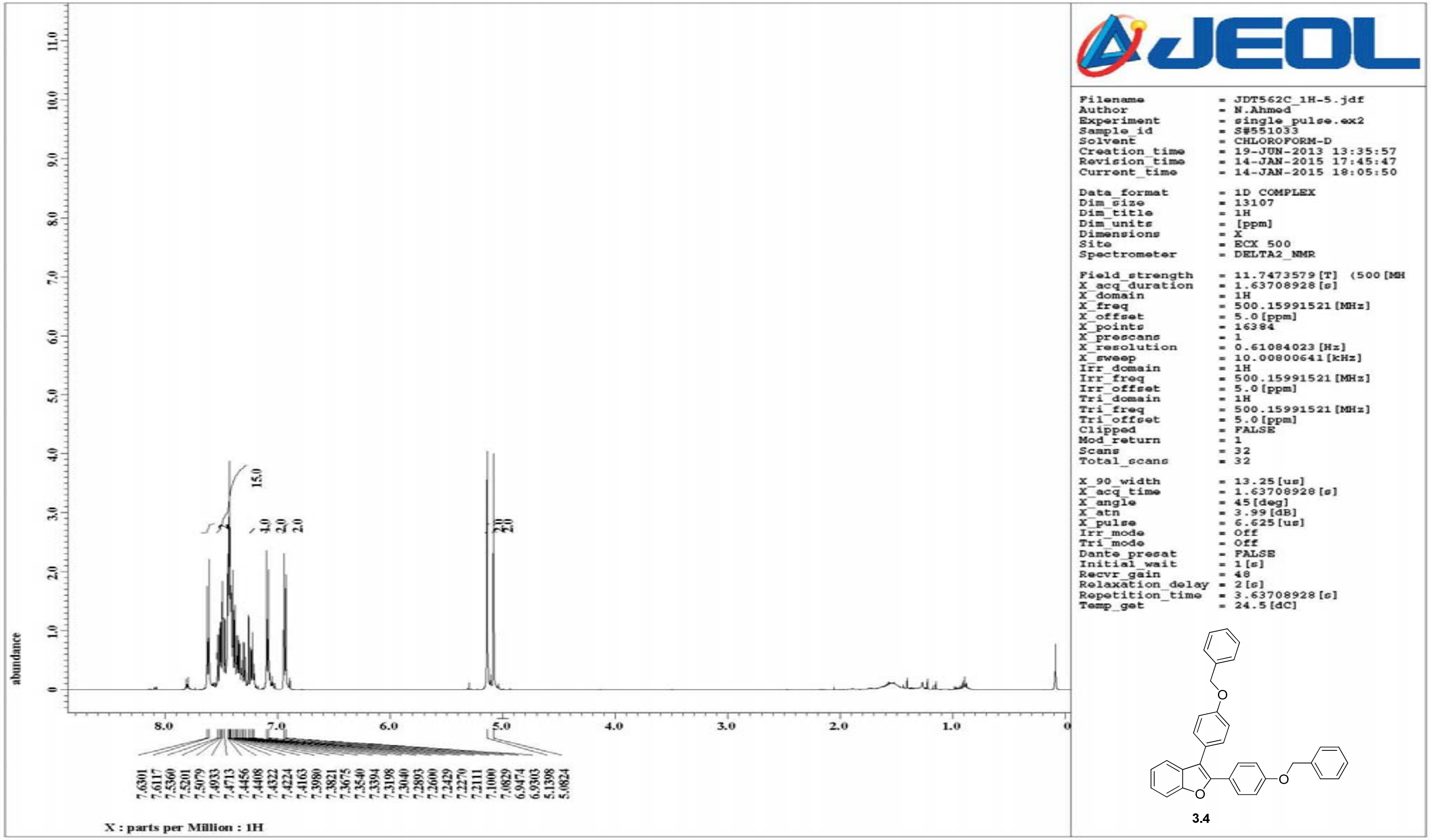

500 MHz ^{1}H NMR spectrum of compound **3.4**

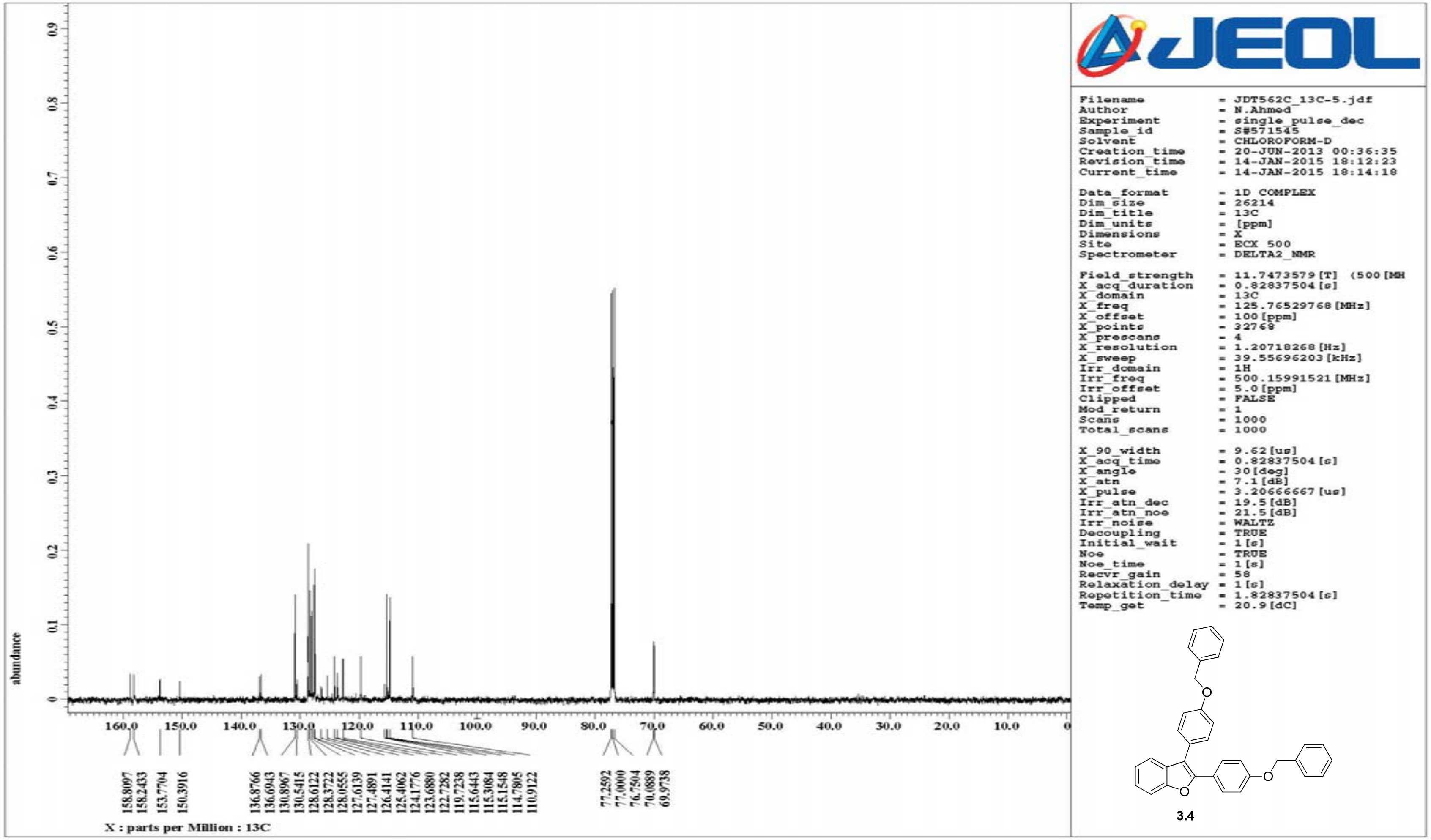

125 MHz ^{13}C NMR spectrum of compound **3.4**

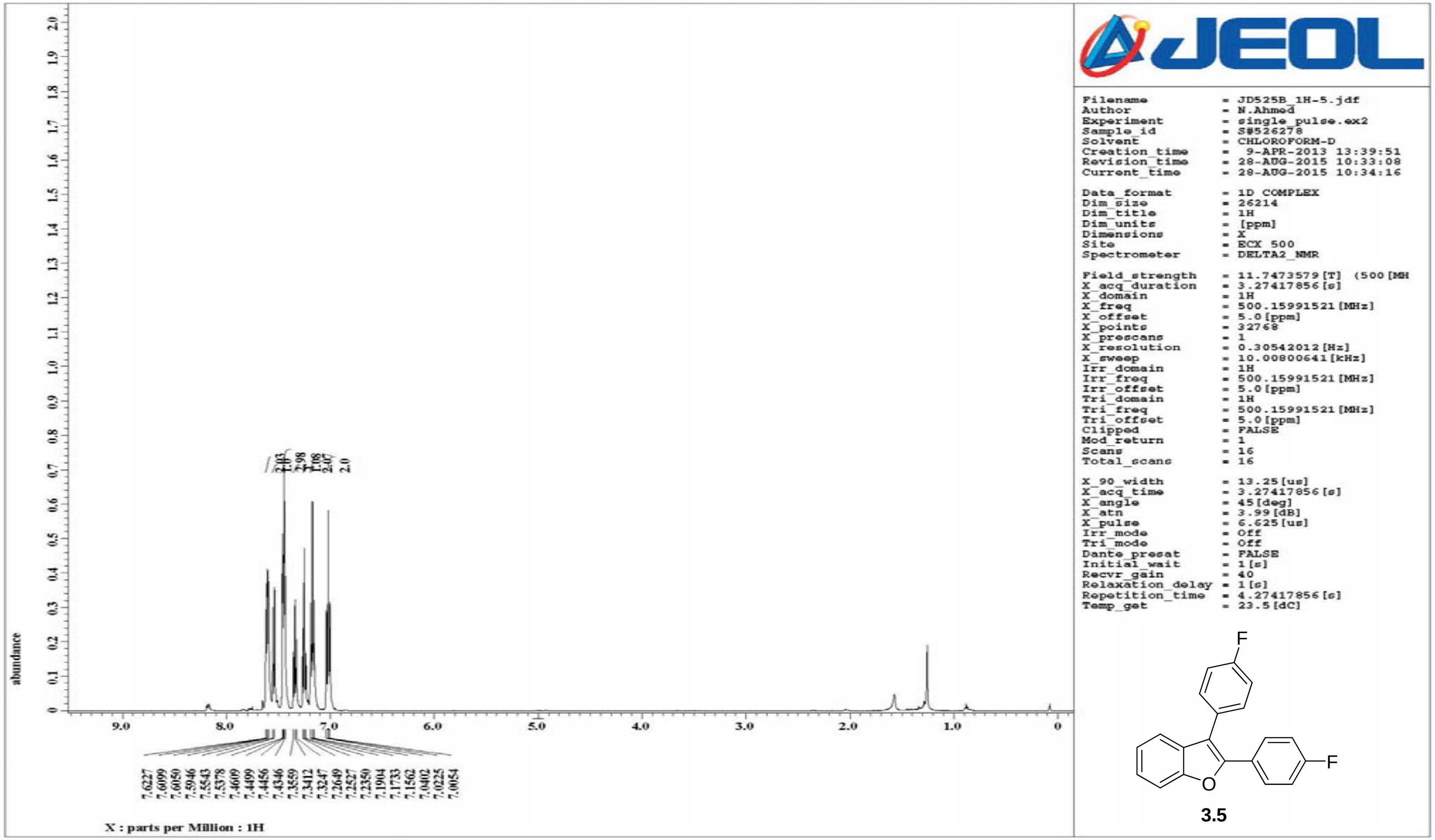

500 MHz ^{1}H NMR spectrum of compound **3.5**

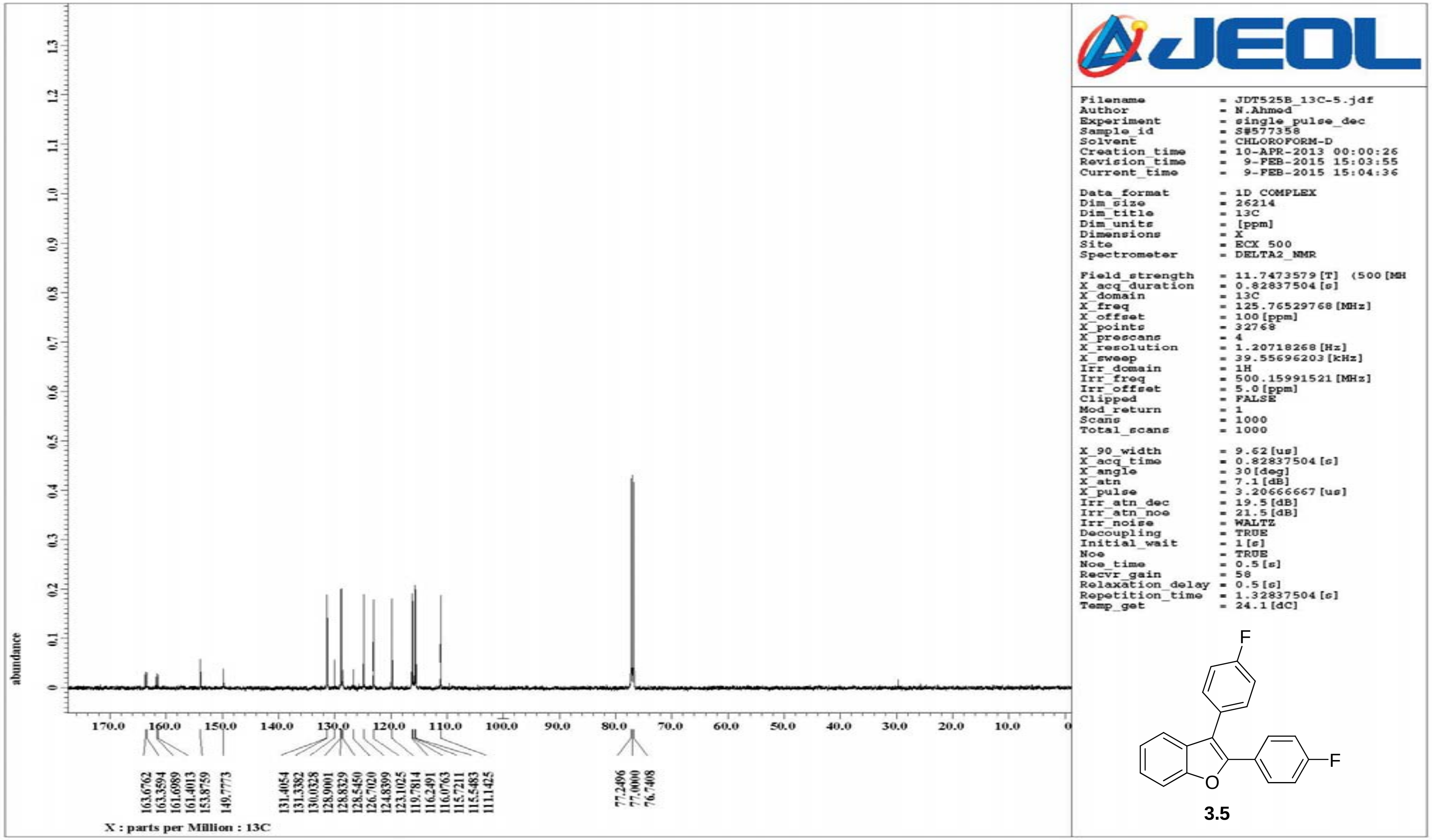

125 MHz ^{13}C NMR spectrum of compound **3.5**

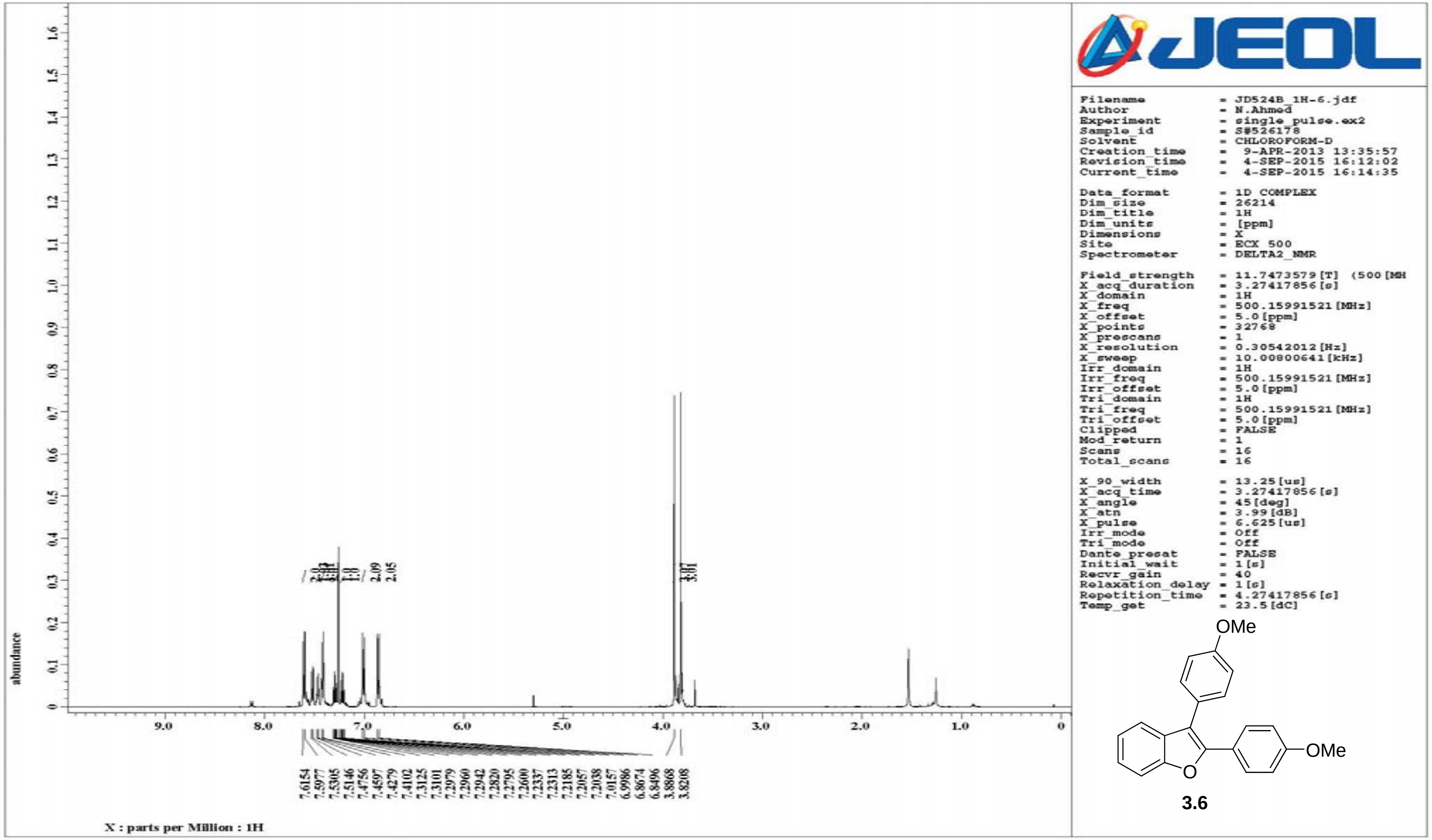

500 MHz ^{1}H NMR spectrum of compound **3.6**

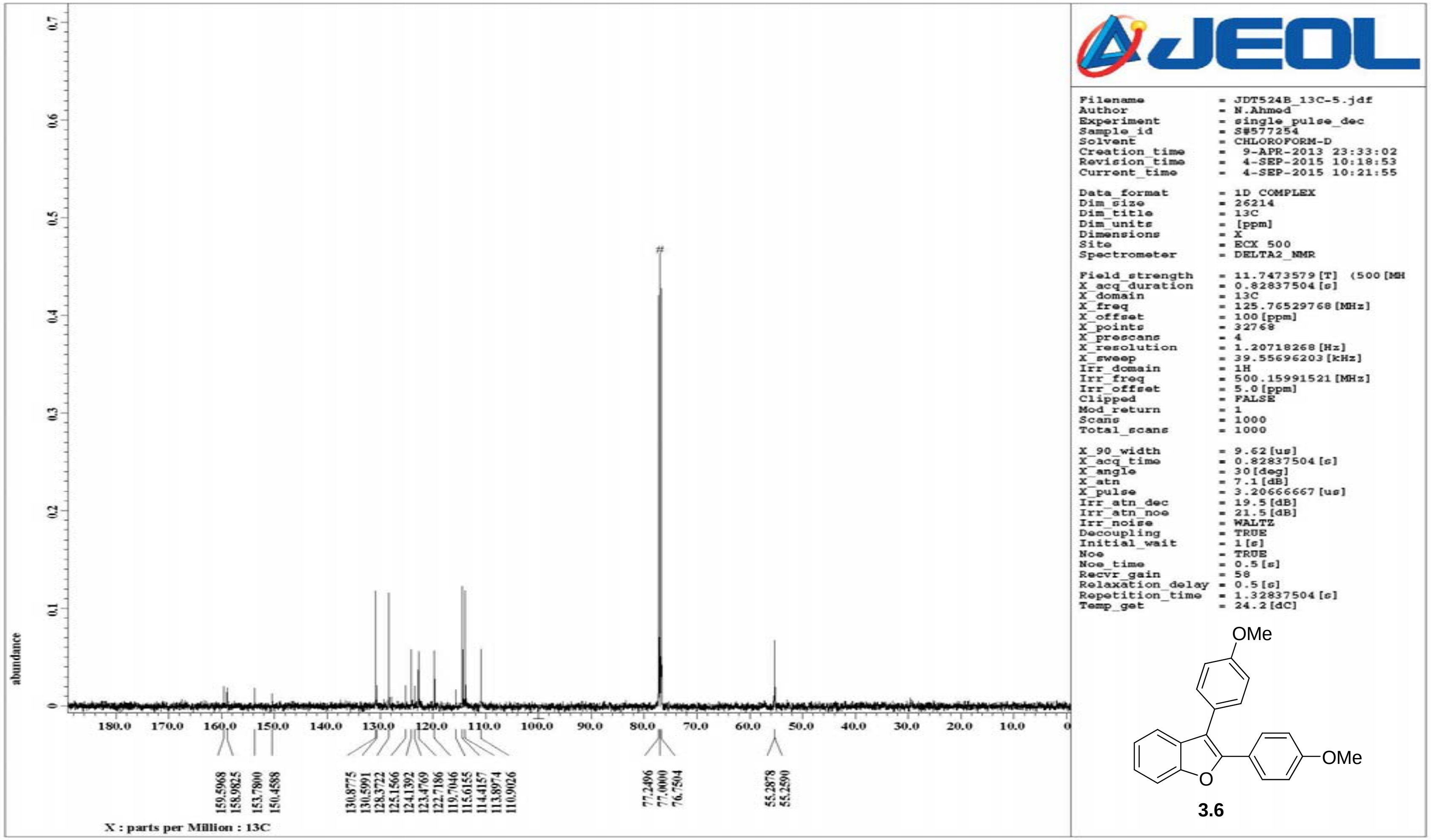

125 MHz ^{13}C NMR spectrum of compound **3.6**

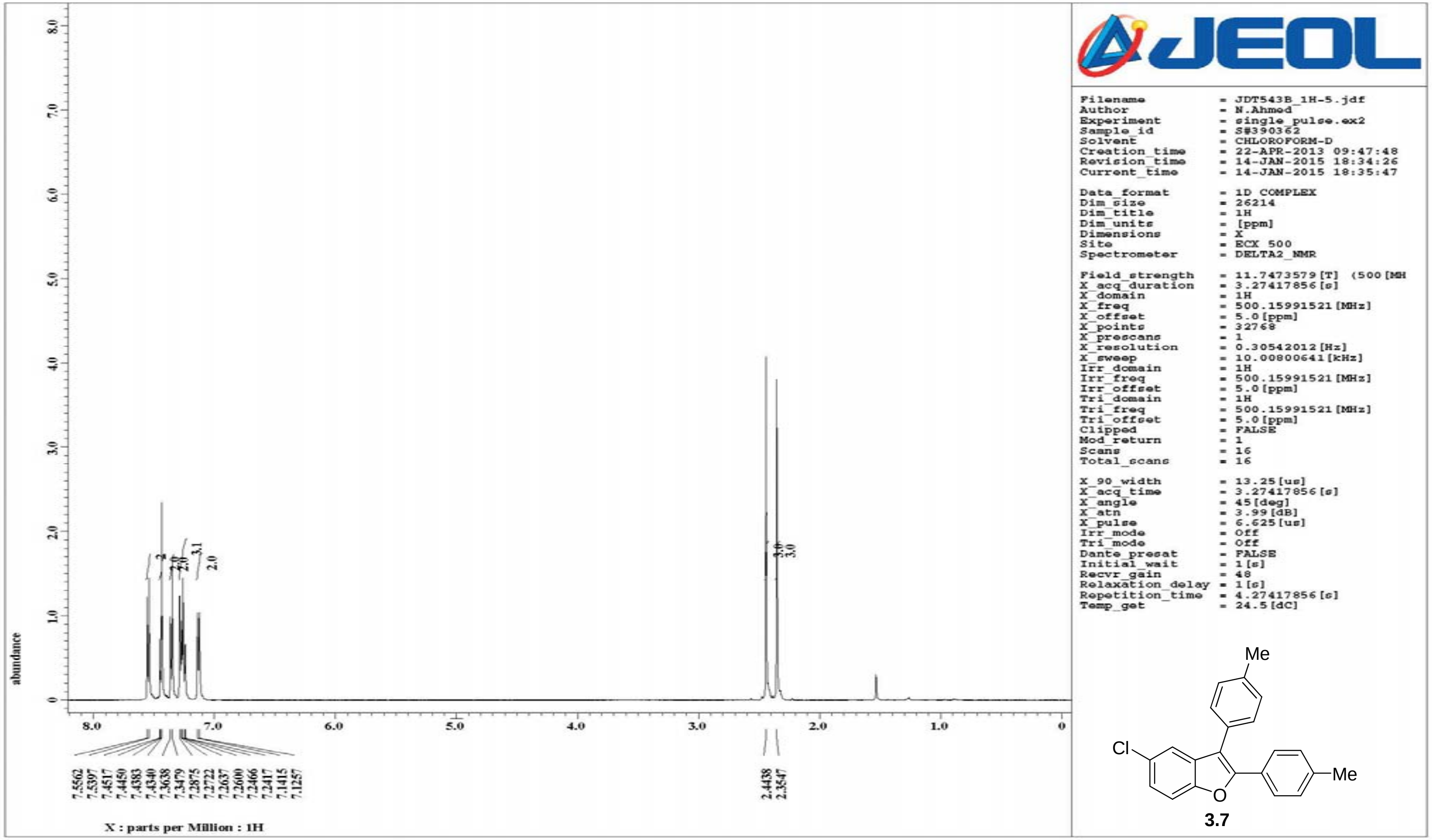

500 MHz ^{1}H NMR spectrum of compound **3.7**

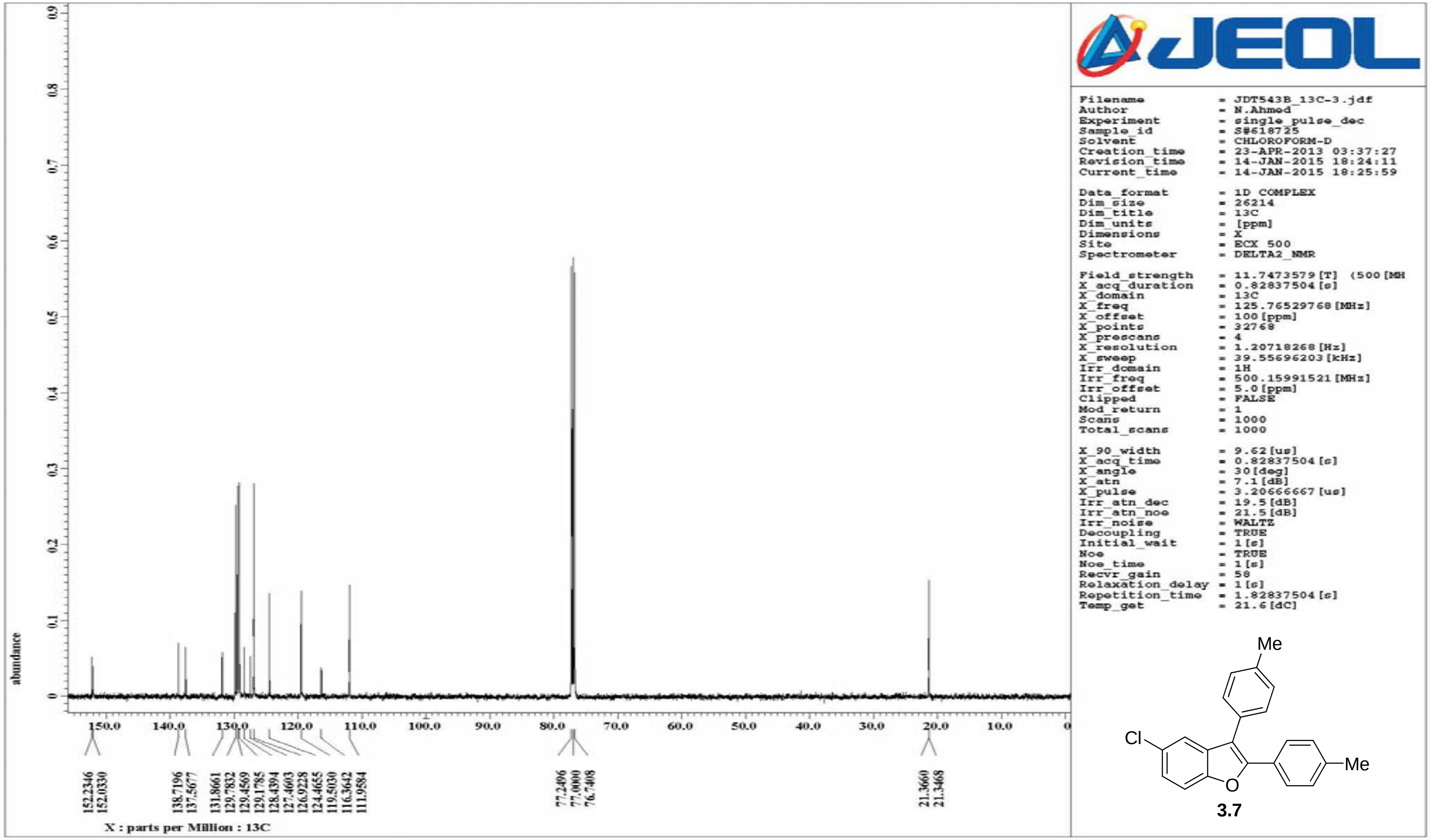

125 MHz ^{13}C NMR spectrum of compound **3.7**

4. ¹H, ¹³C Spectra of Compounds **(4.1-4.3):**

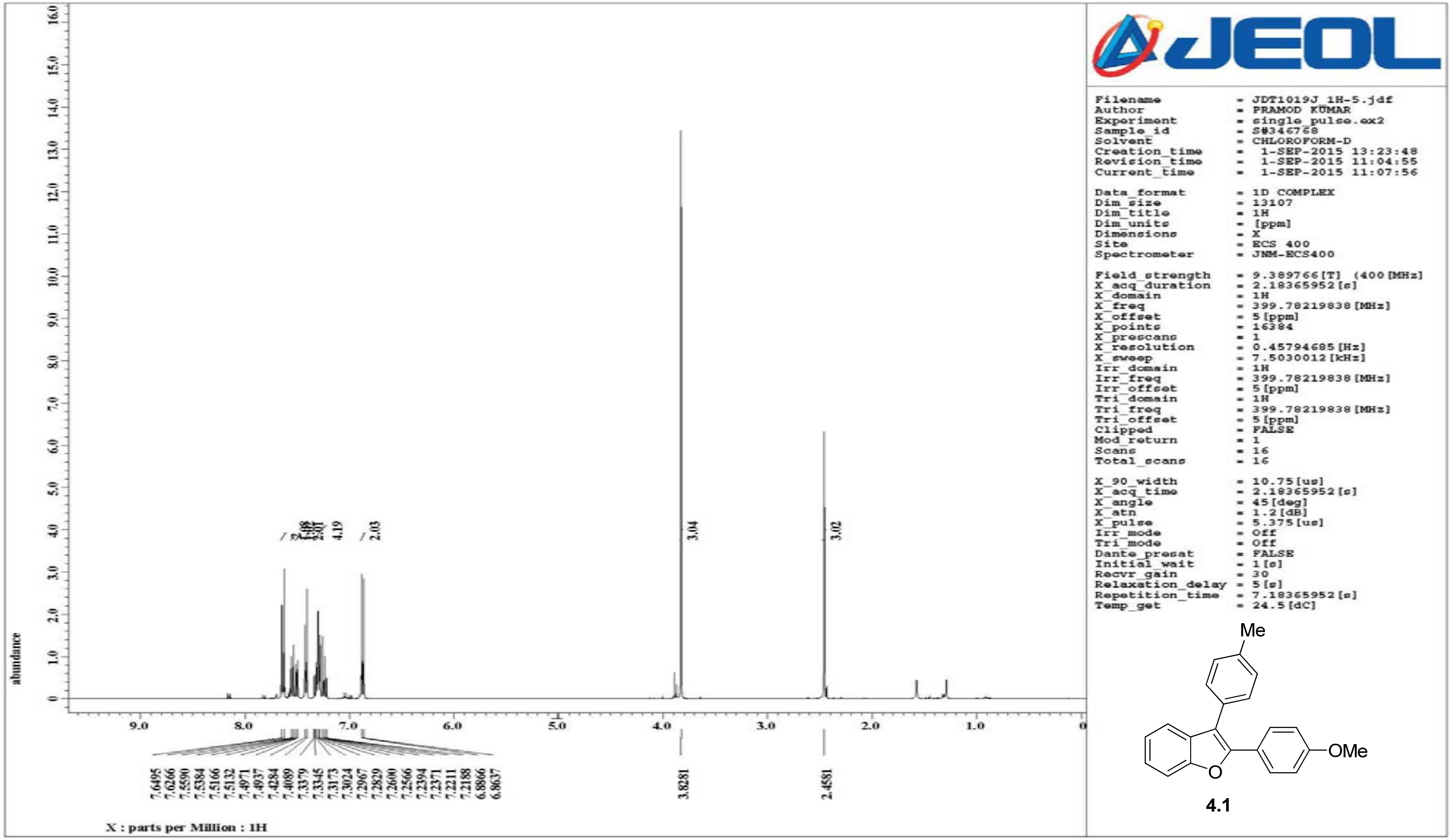

400 MHz ¹H NMR spectrum of compound **4.1**

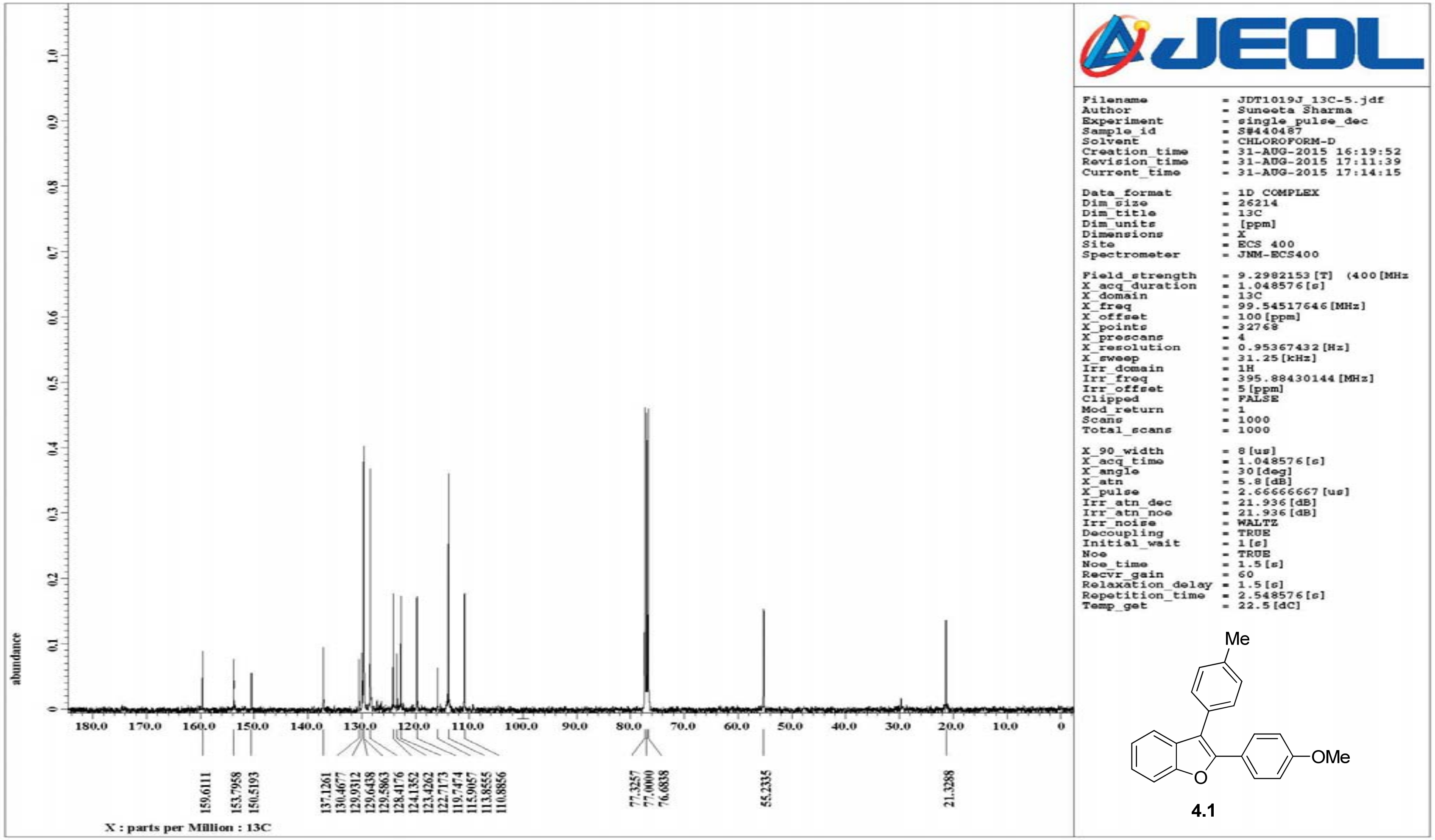

100 MHz ^{13}C NMR spectrum of compound **4.1**

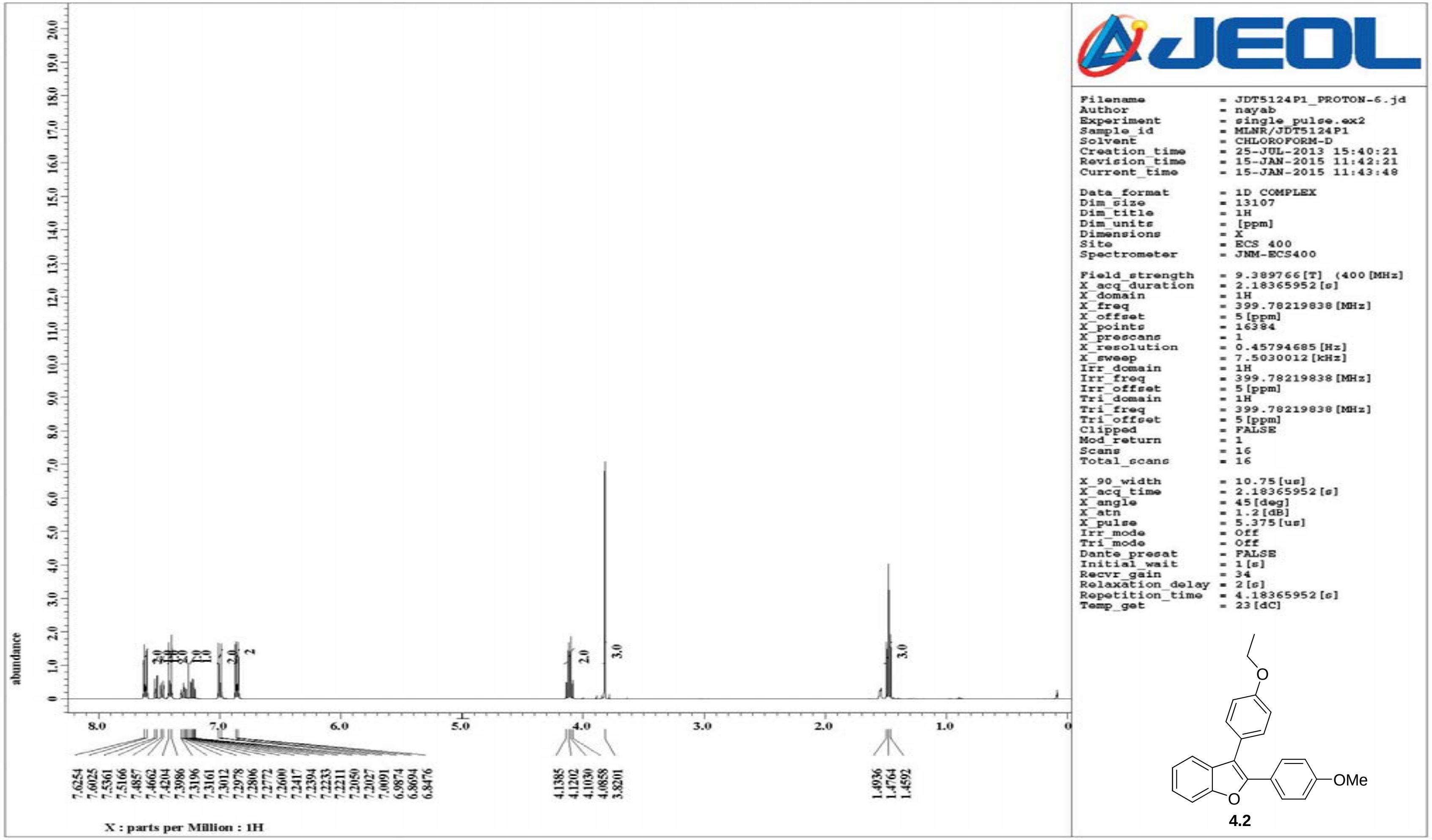

400 MHz ^{1}H NMR spectrum of compound **4.2**

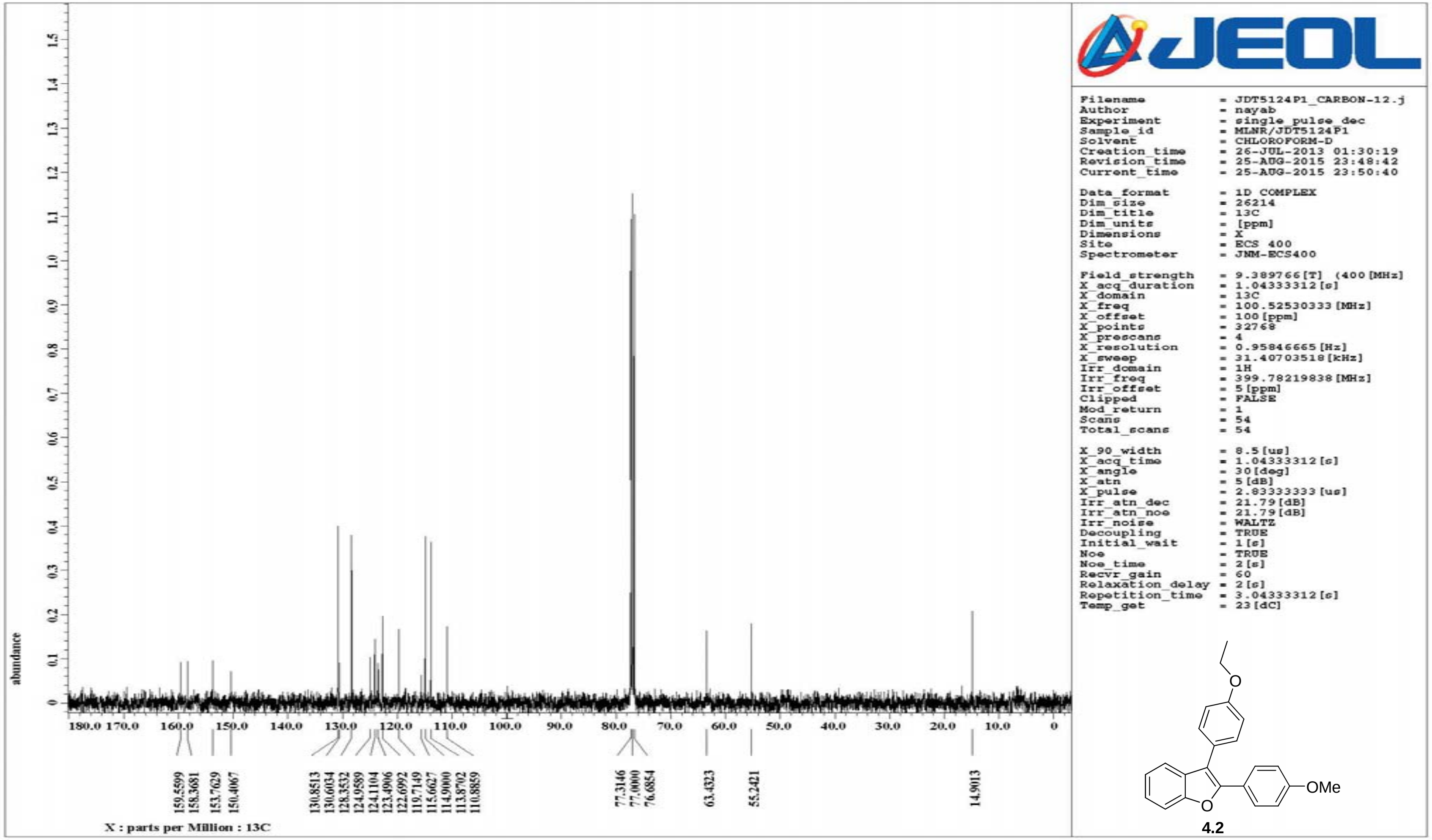

100 MHz ^{13}C NMR spectrum of compound **4.2**

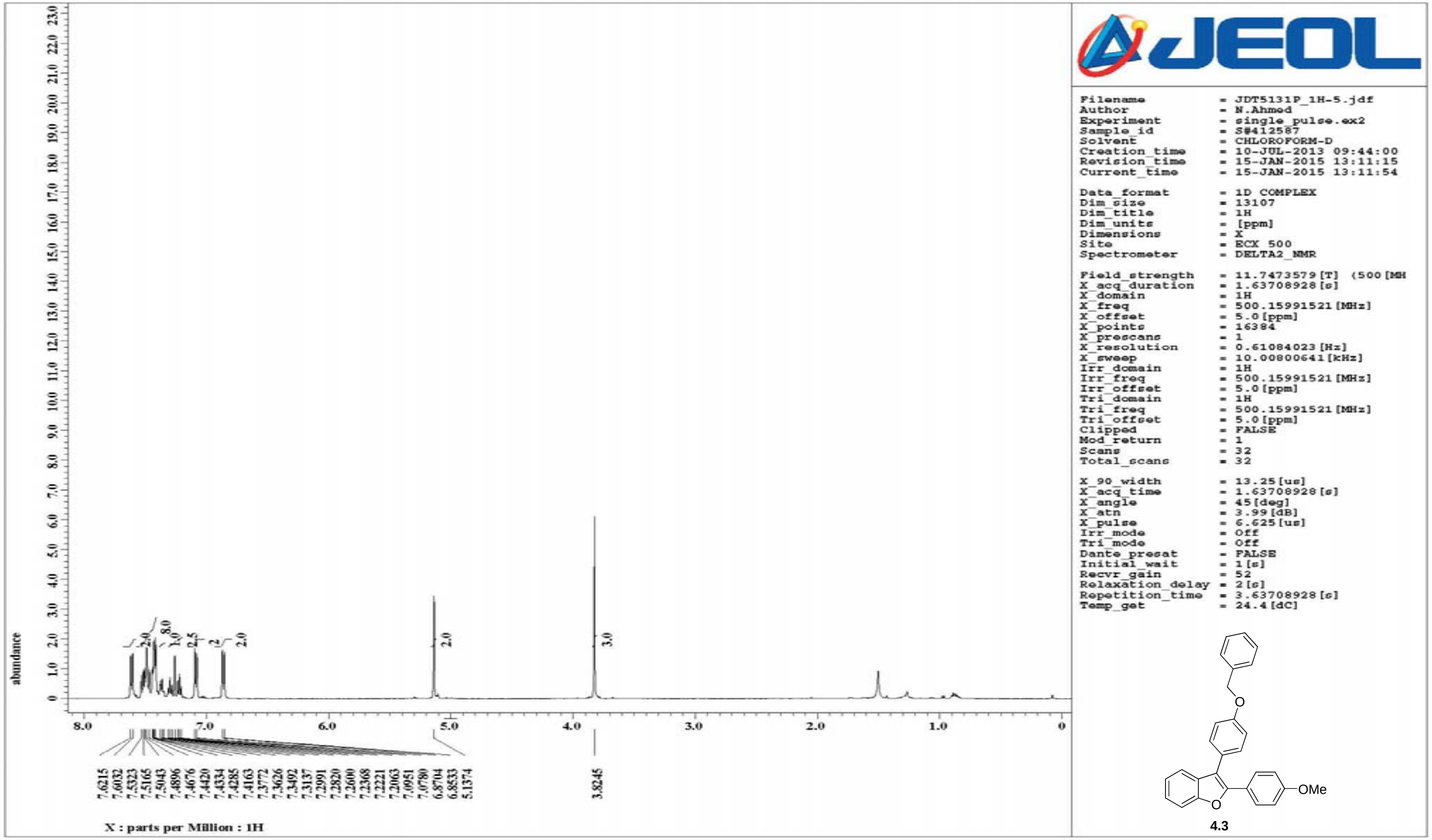

500 MHz ^{1}H NMR spectrum of compound **4.3**

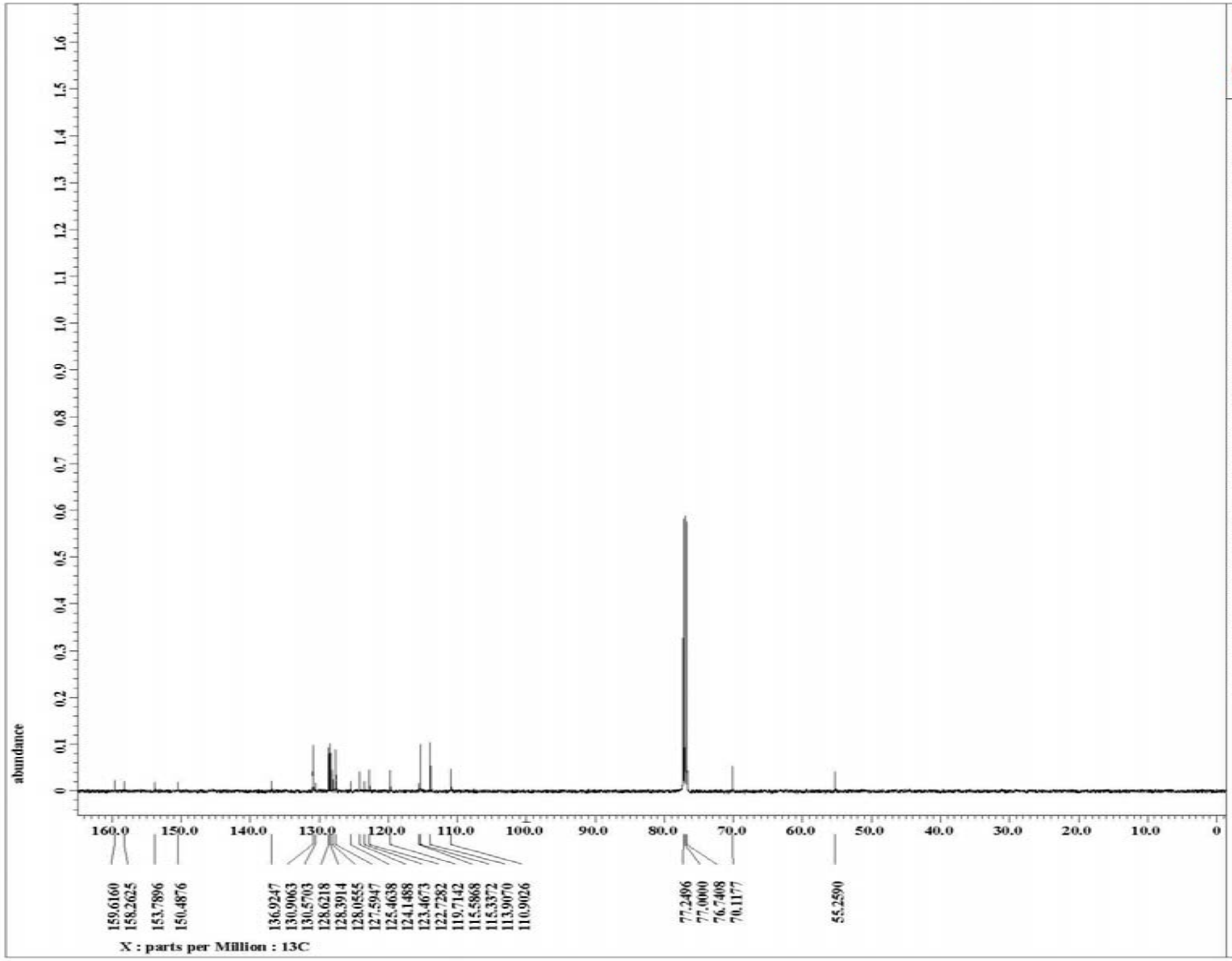

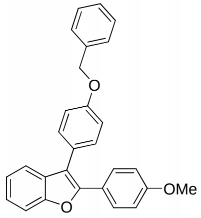

4.3

125 MHz ^{13}C NMR spectrum of compound **4.3**

5. ^{1}H, ^{13}C Spectra of Compounds **(5.1-5.4):**

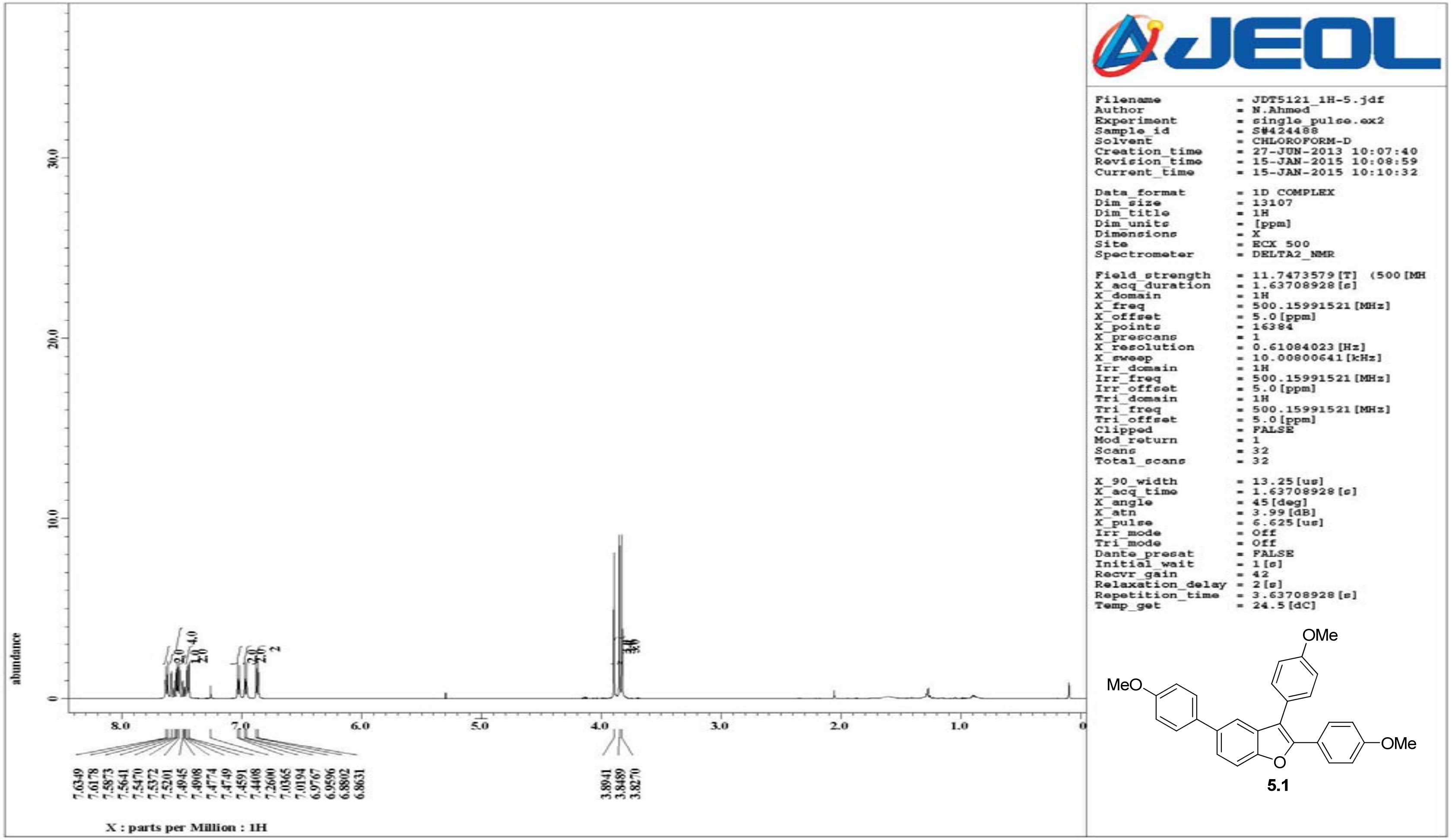

500 MHz ^{1}H NMR spectrum of compound **5.1**

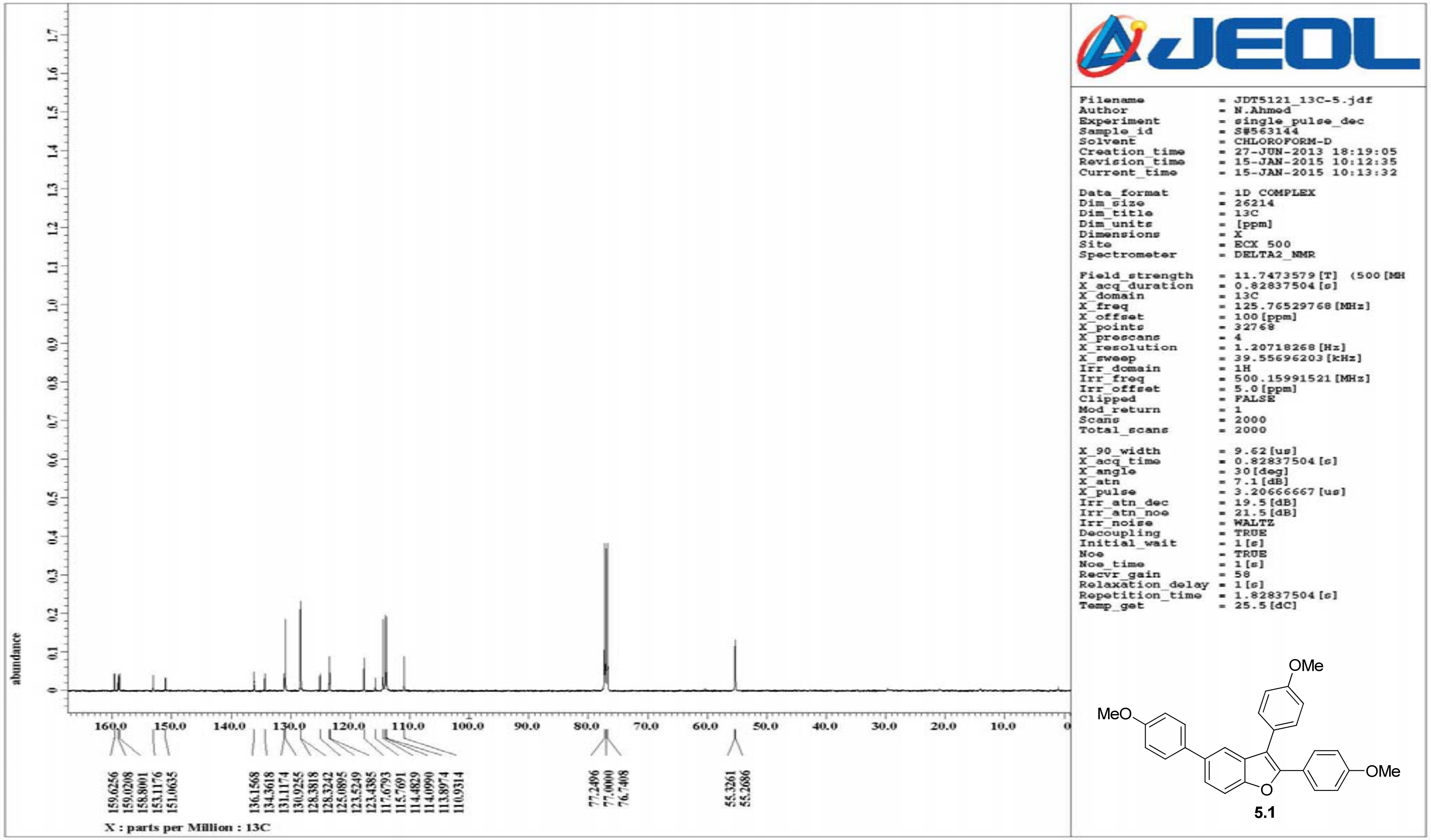

125 MHz ^{13}C NMR spectrum of compound **5.1**

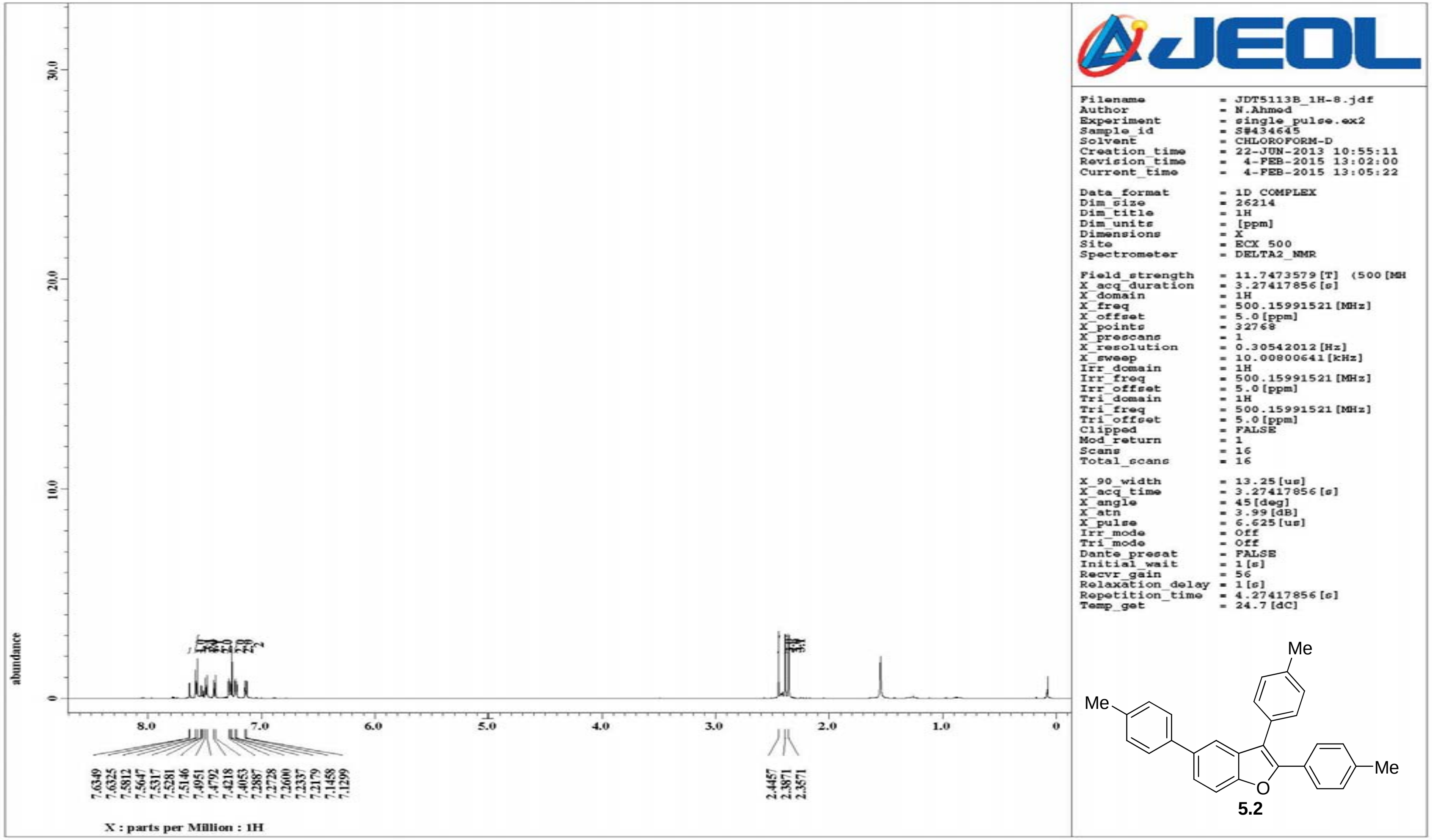

500 MHz ^{1}H NMR spectrum of compound **5.2**

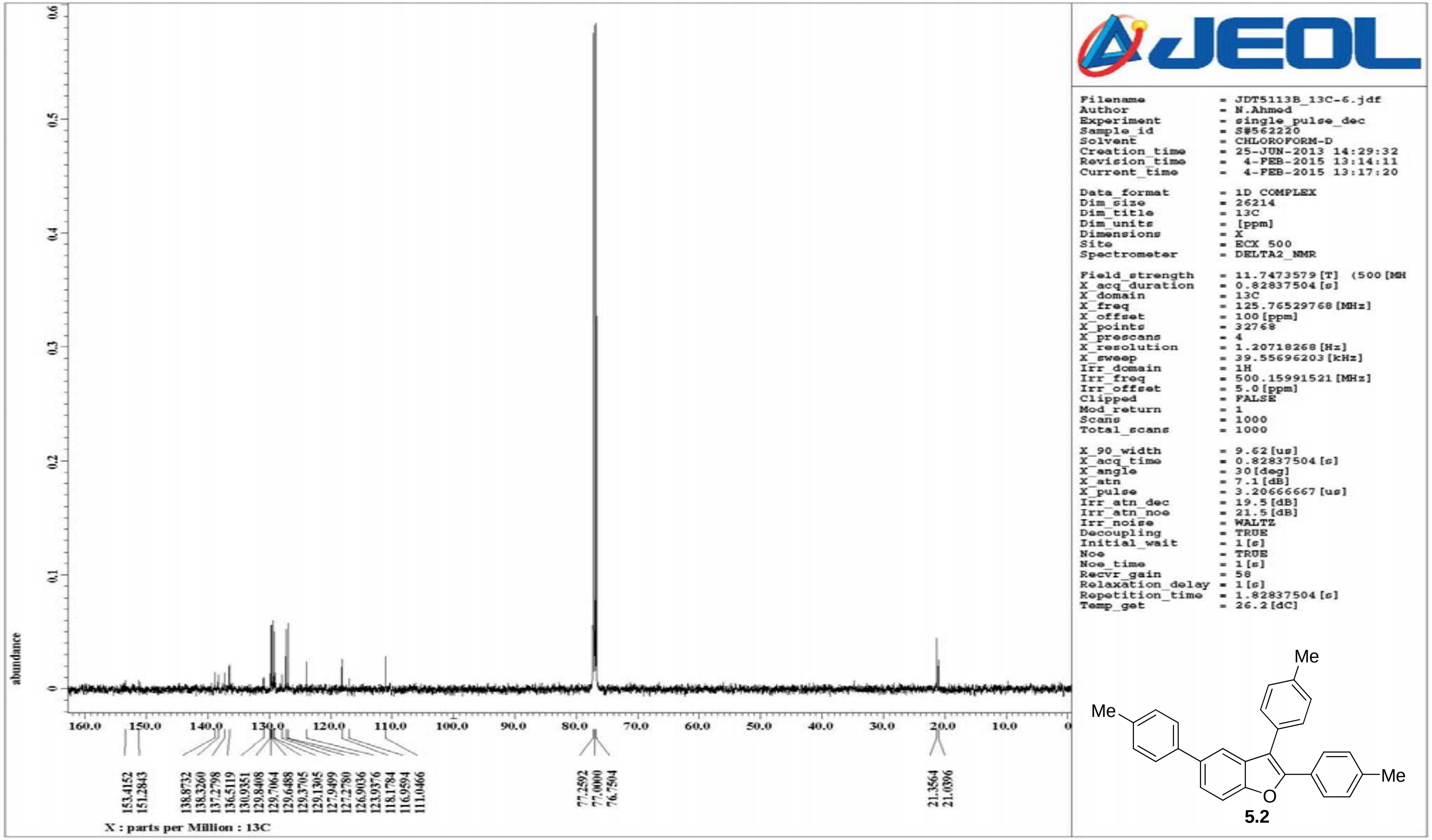

125 MHz ^{13}C NMR spectrum of compound **5.2**

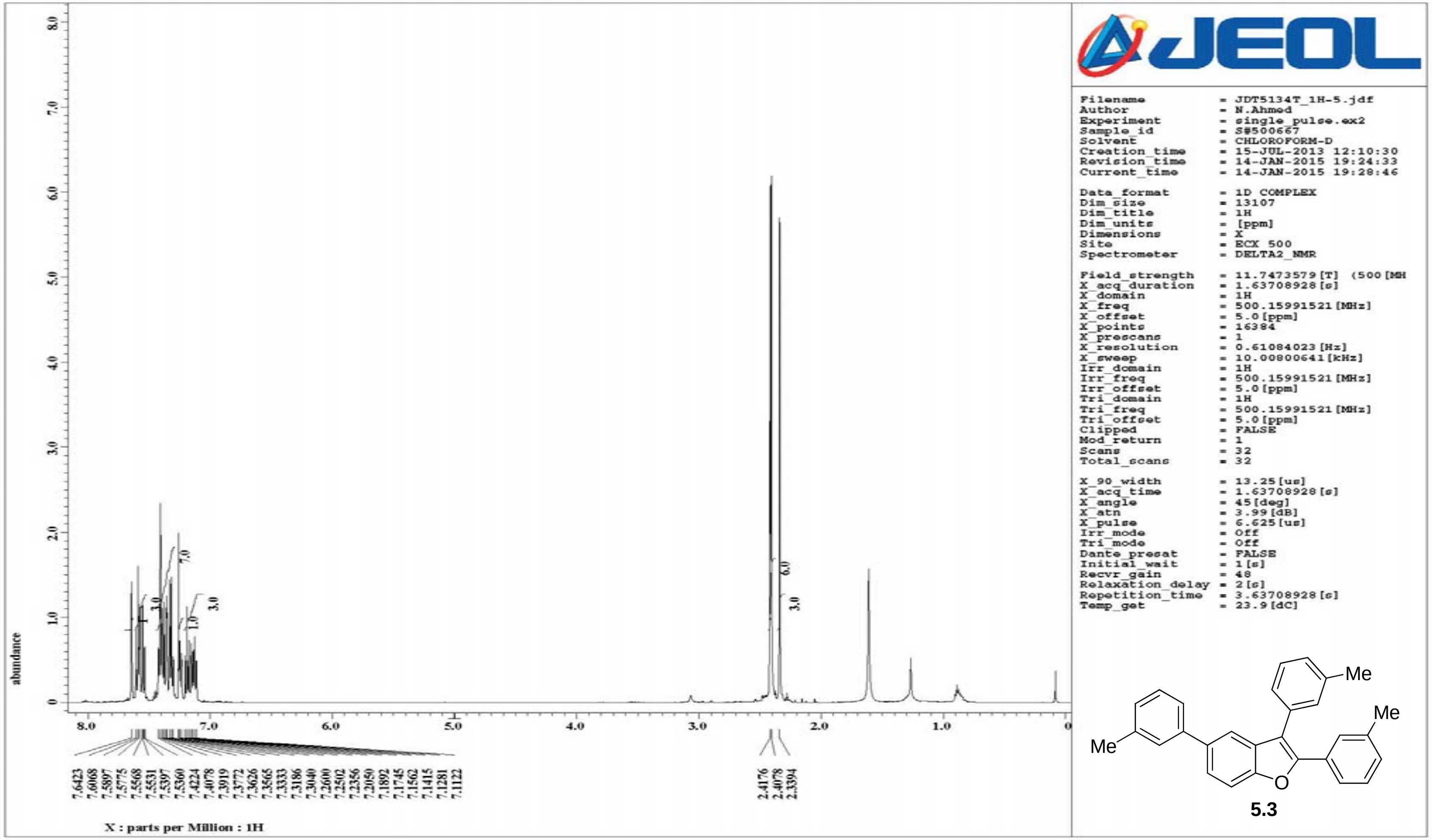

5.3

500 MHz ^{1}H NMR spectrum of compound **5.3**

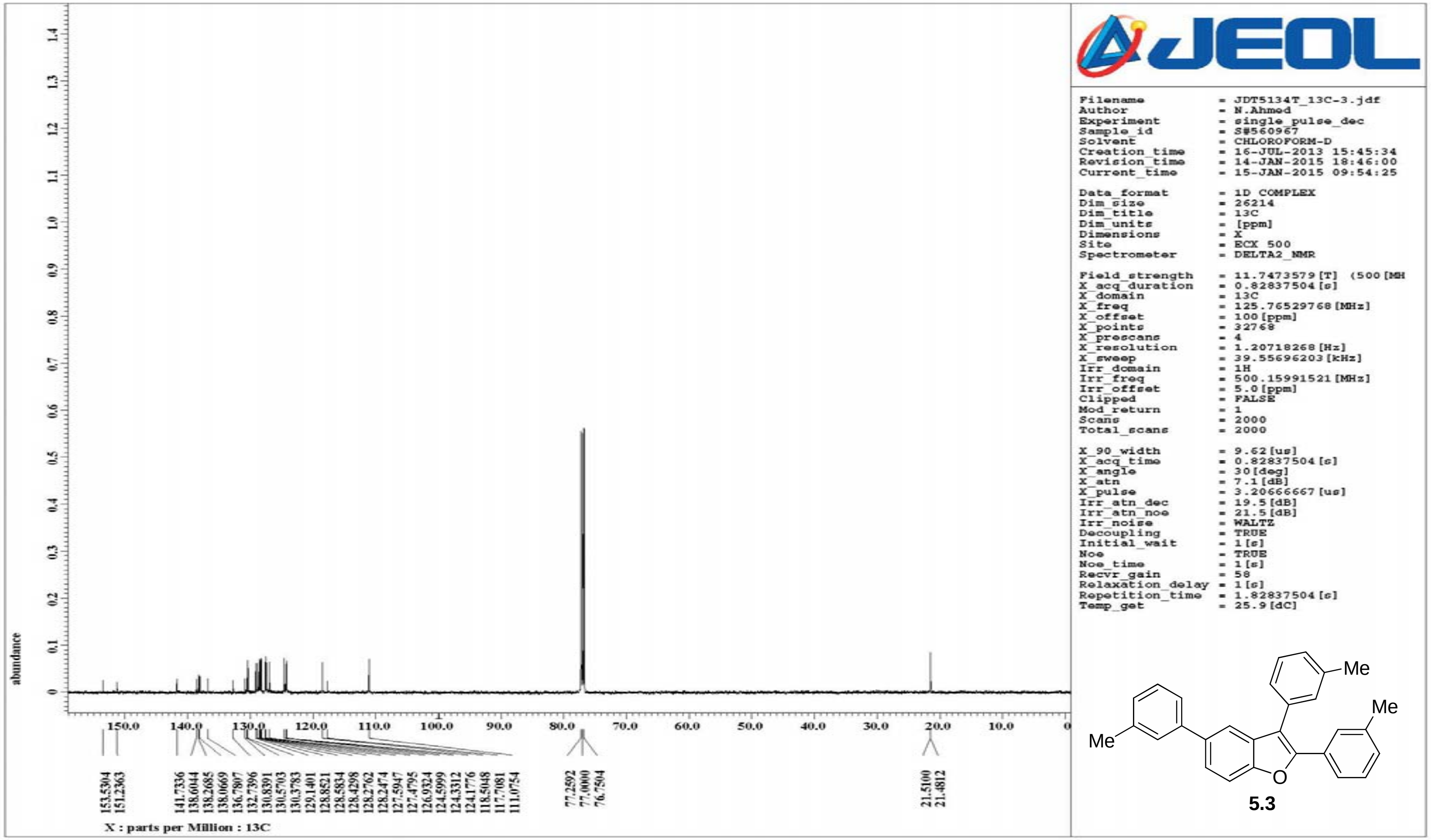

5.3

125 MHz ^{13}C NMR spectrum of compound **5.3**

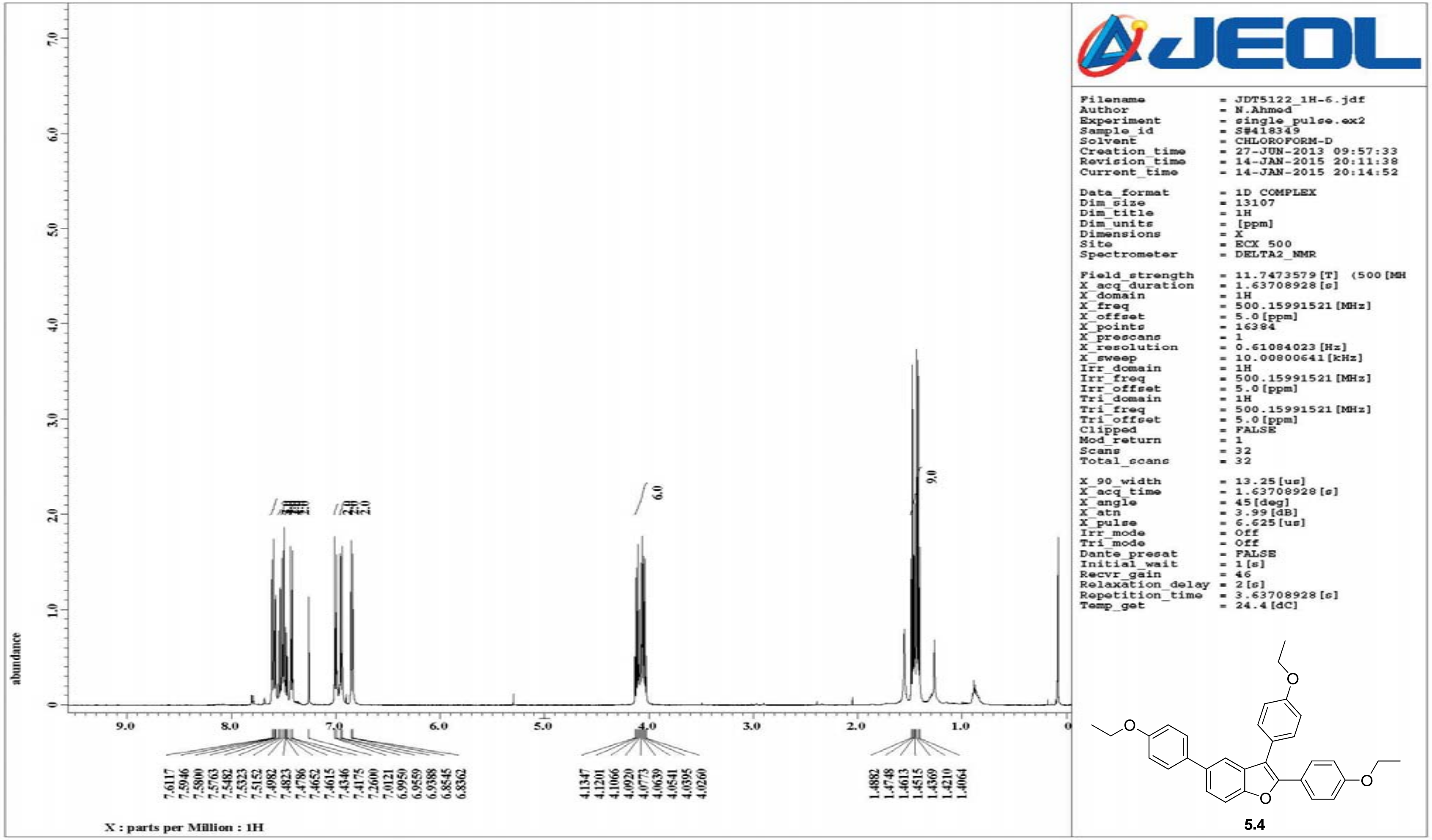

500 MHz ^{1}H NMR spectrum of compound **5.4**

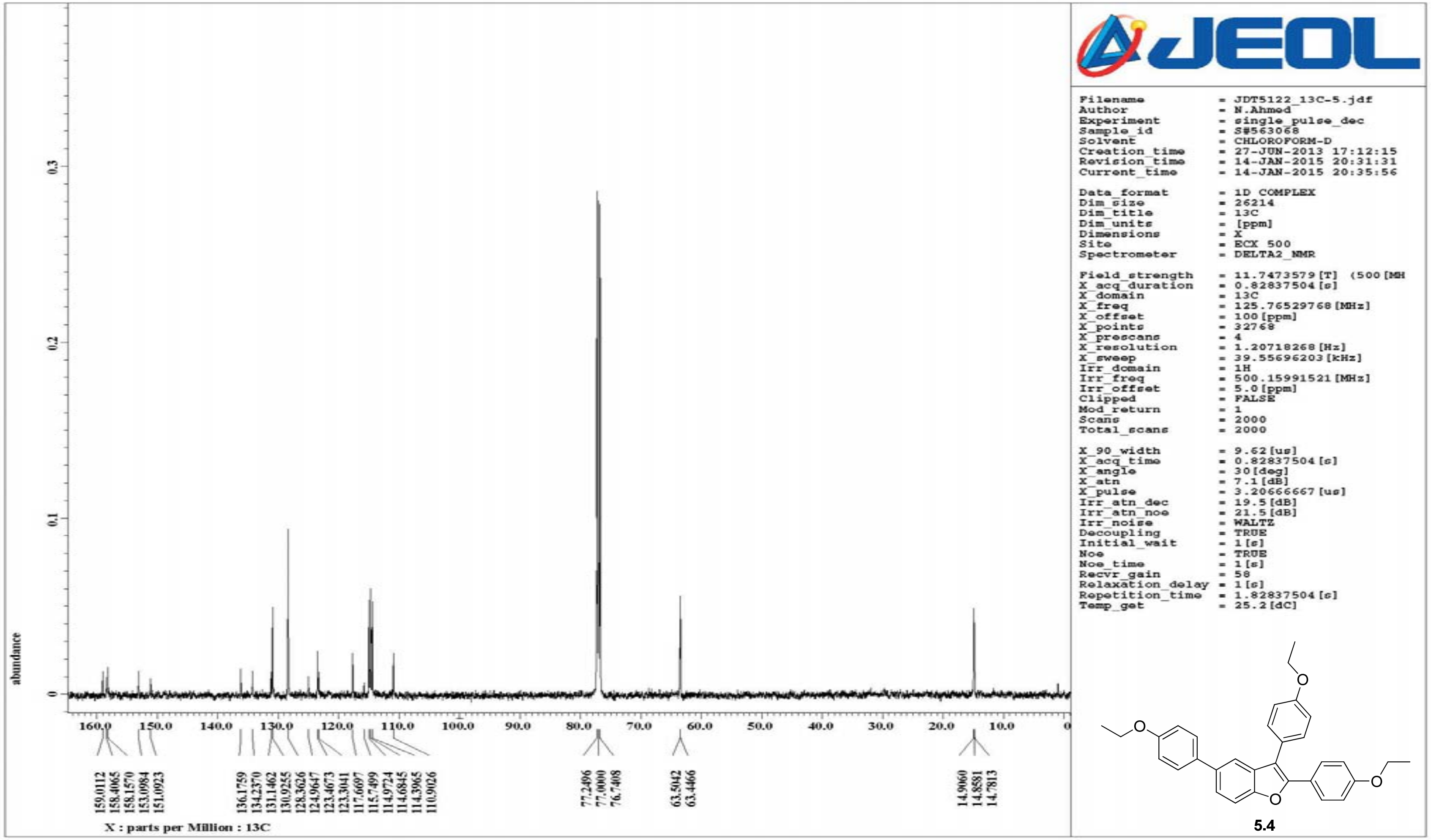

125 MHz ^{13}C NMR spectrum of compound **5.4**
